# The inter- and intra- generational transmission of family poverty and hardship (adversity): A prospective 30 year study

**DOI:** 10.1371/journal.pone.0190504

**Published:** 2018-01-23

**Authors:** Jake M. Najman, William Bor, Zohre Ahmadabadi, Gail M. Williams, Rosa Alati, Abdullah A. Mamun, James G. Scott, Alexandra M. Clavarino

**Affiliations:** 1 School of Public Health, Faculty of Medicine, the University of Queensland, Brisbane, Australia; 2 School of Social Science, the University of Queensland, Brisbane, Australia; 3 Mater Hospital, Brisbane and Faculty of Medicine University of Queensland, Brisbane, Australia; 4 UQ Centre for Clinical Research, Faculty of Medicine, the University of Queensland, Brisbane, Australia; 5 Metro North Mental Health Service, Royal Brisbane and Women’s Hospital, Brisbane, Australia; 6 School of Pharmacy, the University of Queensland, Brisbane, Australia; Centre Hospitalier Universitaire Vaudois, FRANCE

## Abstract

**Background:**

Children exposed to family poverty have been found to have higher morbidity and mortality rates, poorer mental health and cognitive outcomes and reduced life chances across a wide range of life domains. There is, however, very little known about the extent to which poverty is experienced by children over their early life course, particularly in community samples. This study tracks changes in family poverty and the main factors that predict family poverty (adverse life experiences) over a 30-year period since the birth of the study child.

**Methods:**

Data are from a prospective, longitudinal, birth cohort study conducted in Brisbane, Australia. Consecutive families were recruited at the mothers’ first obstetrical visit at one of two major obstetrical hospitals in Brisbane. Data are available for 2087 families with complete data at the 30-year follow-up. Poverty was measured using family income at each time point (adjusted for inflation).

**Findings:**

Poverty affects about 20% of families at any time point. It is common for families to move in and out of poverty, as their circumstances are affected by such adversities as unemployment and marital breakdown. Over the period of the study about half the families in the study experienced poverty on at least one occasion. Only a very small minority of families experienced persistent poverty over the 30-year duration of the study. Logistic regressions with time lag show that family poverty predicts subsequent adversities and adverse events predict subsequent poverty.

**Conclusions:**

Experiences of poverty and adversity are common and may vary greatly over the child’s early life course. In assessing the health consequences of poverty, it is important to distinguish the timing and chronicity of early life course experiences of poverty and adversity.

## Introduction

The adverse consequences of poverty are many and varied [1]. With occasional exceptions, persons who are economically disadvantaged are at increased risk of worse mental and physical health [2], and are more likely to have higher mortality rates for most diseases [3, 4]. Socioeconomically disadvantaged families have children who are more likely to have developmental delays [5, 6], as well as higher rates of morbidity [7–9] and mortality [10]. Children reared in economically disadvantaged families are not only more likely to have poorer health in childhood, but their health as adults also appears to be adversely affected, independent of their own economic standing in adulthood [11, 12].

There is also evidence that life stress and/or adversity are linked to poverty and may contribute to the poor health of the economically disadvantaged. Selye (1936) reported his findings describing the adverse biological consequences of rats exposed to stress in 1936 [13] and humans in 1956 [14]. More recently life adversities have been implicated as a cause of both poor mental [15] and physical health [16].

Despite the documented impact of poverty and adversity on child and subsequent adult health, there is little known about the pattern of child exposure to poverty and adversity over the early life course. Further, little is known about the early-life adversities experienced by children from socioeconomically disadvantaged families [17], and the extent to which these adversities persist after the child leaves home. There is a need to know whether both poverty and life adversities are intergenerationally transmitted.

There are three issues that we address in this study. Firstly, we describe experiences of poverty and life adversity in a sample of families repeatedly surveyed over a 30-year period after the birth of a child. Secondly, we examine patterns of change in experiences of family poverty and adversity over the early life course of this child. Thirdly, we consider the extent to which there is an intergenerational transmission of poverty and adversity. We report the findings from five data collections which track changes in family of origin income and life adversity, as these predict family income and life adversity for adult offspring.

### Poverty: Stability and change over the life course

Estimating the population prevalence of poverty involves judgements and criteria that may vary over time and across national boundaries. In Australia the poverty level is calculated on the basis of 50 per cent of median income and then adjusted for families of varying type and size [18]. Criteria for defining poverty differ for Australia, Europe and the USA [19, 20].

Efforts to specifically document the intra and intergenerational transmission of poverty in economically-developed countries are few. In any event past studies may not provide an accurate indication of what is happening in the current period. Prospective longitudinal studies of the intergenerational transmission of poverty may take 20–30 years to provide data. For example, the most detailed data on the intergenerational transmission of poverty comes from the Panel Study of Income Dynamics (PSID) which commenced in 1968 [21]. The Panel Study of Income Dynamics found that poverty is not a rare event, with one third of US children experiencing at least a year of living in poverty [22]. The PSID also found that most families living in poverty experienced temporary periods of poverty, moving out of poverty once their circumstances changed. Only some 5 per cent of children in this population sample experienced persistent poverty, defined as living in poverty for 10 years or more.

While the process of moving in and out of poverty has been documented in a few studies [23, 24] there is little known about the extent to which this process characterises different population groups and at different time points. Given that the available studies were initiated many decades ago, it is unclear whether the situation remains characteristic of the current period in economically developed countries, leading one reviewer to note that there is a “largely inconclusive evidence base surrounding life course and intergenerational poverty transmission” [23].

### An intergenerational transmission of poverty

Socioeconomic status in general, and poverty in particular, are associated with a wide number of structural (human capital) and behavioural or social (social capital) characteristics. Many of these characteristics may contribute to the persistence of intergenerational poverty. The structural factors involve income and assets, patterns of marriage and employment (or unemployment), levels of education and skill attainment. The behavioural and social factors include a wide range of behaviours (e.g. lifestyle, patterns of parenting), as well as aspirations and attitudes–for example, to having children, child rearing, and child care. To the extent that there is an intergenerational transmission of poverty, there is also likely to be the intergenerational transmission of the many related structural and behavioural characteristics associated with poverty.

While there are only a limited number of studies tracking the intergenerational transmission of poverty, their findings are broadly consistent. These studies confirm that children reared in poverty, are more likely to themselves experience poverty in adulthood.

### The causes of poverty: Long-term trends

There are three long-term trends contributing to increases in the proportion of the population living in poverty. If these trends continue then the proportion of populations living in poverty in developed economies can be expected to increase, irrespective of the capacity of some individuals to improve their social and economic circumstances. These trends involve changes in family composition, changes in the nature of labour markets, and the aging of the population.

In Australia the poor are largely comprised of three groups: single mothers, the under-employed or unemployed and (aged) pensioners [25].

For at least the last two decades the rate of marriage has been steadily declining[26, 27]. As a consequence of these changes there has been a large increase in the proportion of women who are not currently married to the father of their child. The rate of marital dissolution has been increasing since the 1970s, though more recent data suggests that the rate has plateaued with 40–50 per cent of marriages breaking down [27]. Between one in two and one in three children will live for at least for some period of the child’s early life with a single parent. Single parents, generally, receive an income below the poverty line. The current high rate of single parenthood will continue to substantially contribute to the overall rate of poverty.

From the perspective of levels of unemployment, much of the productive process has been globalised with the consequence that there is little work available for those with low education in many developed countries. While the Global Financial Crisis (GFC) of 2008 led to increased unemployment, the improvement in economic conditions subsequent to the GFC has not seen many of those positions restored [28]. It appears that employers have chosen technological advancements and the casualization of labour, rather than to re-employ their workforce. The availability of manual and unskilled work continues to decline, and economic growth largely benefits a more educated workforce. The proportion of elderly living on pensions continues to increase. This increase makes a modest contribution to those living in poverty in the Australian population.

While some factors contributing to poverty are increasing over time, there is little known about how these factors might impact over the early life course. This study examines changes in levels of poverty and life adversity, as these are experienced over the early life course.

## Hypotheses

1. There is a high level of persistence in family poverty across the child’s early life course and across generations.
2. There is a high level of persistence in life adversities (stressful life events) across the child’s early life course and across generations.
3. Poverty over the child’s early life course predicts subsequent adversities, but adversities do not predict subsequent poverty.

## Methods

Data are from a mother-child linked pre-birth cohort study. Recruitment of mothers commenced in January 1981 and continued until December 1983. Prospective mothers were recruited at their public hospital, obstetrical booking-in visit. About 50% of Australian women use public hospital obstetrical services for their pregnancies. Some 8556 consecutive prospective mothers were approached and invited to participate in the study. Almost all (8458) agreed. Study inclusion criteria required the mother give birth to a live singleton baby at the study hospital. Some 7223 mothers met those criteria. At the 21-year follow-up data were available on 3805 offspring (52.7 per cent retention) and 3754 mothers (55.6 per cent retention). At the 30-year follow-up 2900 offspring participated (see S1 Fig for a flow diagram). The analyses which follow are for 2087 respondents for whom we have complete data over the 30 years of the study. We have previously noted that those lost to follow-up are disproportionately the most economically disadvantaged persons in the study not only in economic terms, but also in their physical and mental health. While this pattern of loss to follow-up raises concerns about possible bias in findings, we have repeatedly tested these concerns and reported that efforts either to correct for bias or model its likely consequences suggest the findings are not misleading. While the distributions on key variables in those lost to follow-up are different from those who remain in the study, the estimates of association are very rarely affected [29].

### Measures

Family income was derived from the respondents’ self-report provided at recruitment. Families whose income was around or below the Australian poverty line were categorised as poor or living in poverty. S1 Table provides expanded details of the family income distributions at each follow-up. For most analyses we have dichotomised the data so that about 20 percent of the lowest income families are designated living in poverty. This categorization is a reflection of the National Poverty Line [25]. For some adjusted analyses we use concurrent income. In these instances the coding of the relevant income variables approximates the “poverty line income” at the time point when the data were collected. Family income is reported by the mother up to the 21-year follow-up and the offspring’s own family income is recorded at the 30-year follow-up.

Life adversity exposures are measured using a shortened form of the life events scale [30]. Life events scales comprise reports of salient events which may have substantial impacts on behaviour and mental health; for example the death of a close relative, marital breakdown or unemployment. Such events have been found to have good test–retest reliability and good concurrent validity based upon independent reports. The specific items in this scale varied somewhat from study phase to study phase (See S2 Table for a complete list of items used at each phase of the study and their related reliability coefficients). Maternal reports of life events (adversities) are provided at recruitment, and 5- and 14-year follow-ups. At the 21-year and 30-year follow-ups the life events scale comprises items reported by the offspring. While the list of items varies somewhat from time-to-time, previous studies, using a larger list of items, suggest that whatever list is used is likely to represent a subset of a much larger list of adverse life experiences commonly reported by families [31]. The distributions of life events (adversities) at each phase of the study are in the S3 Table.

Data are presented as adjusted for a number of potential confounders. These potential confounders (mother’s age, education and marital status) are self-reported and taken from the recruitment phase of the study.

## Results

Of the 2404 respondents with data at the 21-year follow-up, only 43 (1.8 per cent of sample) were recorded as living in poverty at all four previous phases of data collection. Of these 43, only 12 of the offspring were living in poverty at the 30 year follow-up. Of those offspring living in poverty at the 30-year follow-up (N = 471), 39.3 per cent had not experienced poverty at any of the previous data collections (to 21 years of age), while 36.1 per cent had repeated (2+) experiences of living in a family experiencing poverty. Some 49.1 per cent of offspring not in poverty at 30 years (N = 1933), had experienced poverty at a previous phase of data collection. The data confirms that about half of the families in this study experienced poverty at some stage during the child’s early years, and that a history of poverty is more common for those offspring who are currently living in poverty (Table 1).

**Table 1:** Offspring experiences of poverty and adversity over the first 30 Yrs.

Number times	Poverty (N = 2087)*	Adversity (N = 2087^)^**
	%	%
**0**	40.7	48.9
**1**	30.3	28.9
**2**	15.9	14.3
**3**	8.2	5.7
**9+**	4.8	2.1
**Total**	100	100

* Family Income is reported by mother at first clinic visit, 5, 14 and 21 years; at 30 years income is reported by offspring and is for self and partner.

** Adversity is reported by mother for family at first clinic visit, 5 and 14 years. Offspring reports own adversity at 21 and 30 years.

Rates of poverty are consistently very high for those women who are separated/widowed/divorced, and for those who describe themselves as single. Those who describe themselves as living together, that is with a partner but not married, appear to exhibit levels of poverty not greatly different from of the levels experienced by married persons. The employment status of the respondent and partner of the respondent also strongly predict poverty status, with those reporting they have no partner or they or their partner are unemployed, also reporting very high rates of poverty. Benefit (welfare recipient) data was only collected at first clinic visit (FCV) and at the 30-year follow-up. As anticipated those receiving welfare benefits are frequently living below the poverty line.

Table 2 presents the dichotomised form of the family income and adversity variables. To the extent possible we have created categorical variables in Table 2, which have similar distributions and are based upon similar criteria. It is noteworthy that adversities are experienced by a substantial proportion of the sample, with perhaps 15–20 per cent of the sample, at each phase, reporting they have experienced three or more adversities. For much of the sample of children (and their mothers), experiencing adverse life events is common.

**Table 2:** Distribution of family income and adversity at study follow-ups.

	Family Income*			Adversity **			
	N	Not Poor %	Poor %	N	Low (0–2) %	Borderline (3) %	High (4) %
**FCV**	1992	71.5	28.5	2072	86.1	8.5	5.5
**5 years**	1897	78.5	21.5	1944	82.1	11.3	6.6
**14 years**	2051	82.8	17.2	2087	82.6	9.1	8.3
**21 years**	1913	73.4	26.6	2187	77.8	11.2	11.0
**30 years**	2030	80.8	19.2	1998	86.2	7.4	6.5

* Family Income is reported by mother at first clinic visit, 5, 14 and 21 years; at 30 years income is reported by offspring and is for self and partner.

** Adversity is reported by mother for family at first clinic visit, 5 and 14 years. Offspring reports own adversity at 21 and 30 years; FCV: first clinic visit.

Table 3 provides the correlations for family income and adversity over the 30 years and across two generations. The correlations are clearly strongest for family income between the 5- and 21-year follow-ups. It is, however, notable that offspring’s family income is related to their family of origin incomes. These correlations are weak (but significant) associations. A similar though weaker pattern to the one observed for family incomes is also evidence of the persistence of life adversities across generations. Although adversities reported by mothers show evidence of persistence over the life course, these associations are weak (though statistically significant). It also appears that the strength of these associations may decline over time.

**Table 3:** Family income and life adversities across generations.

		Kendall’s Tau B				
		FCV	5 years	14 years	21 years	30 years
**Family Income across generations**	**FCV**	1.0	0.35**	0.26**	0.27**	0.11**
	**5 years**		1.0	0.45**	0.37**	0.17**
	**14 years**			1.0	0.56**	0.18**
	**21 years**				1.0	0.18**
	**30 years**					1.0
**Life adversities across generations**	**FCV**	1.0	0.20**	0.16**	0.09**	0.03
	**5 years**		1.0	0.26**	0.14**	0.07**
	**14 years**			1.0	0.13**	0.05*
	**21 years**				1.0	0.18**
	**30 years**					1.0

* p < .05.

**p < .01.

Mean correlation for family income over the early life course = 0.29; Mean Correlation for family adversity over the early life course = 0.13; Numbers vary slightly, N = 1876

In part A of Table 4 we examine the extent to which family income at the time of recruitment predicts life adversities over the family’s (child’s) subsequent life course. Family income at recruitment (FCV) predicts adversity at each subsequent follow-up, but adjustment for possible confounders (mother’s age, education and marital status) attenuates these associations and most associations are no longer statistically significant. The exception involves poverty predicting adversity when both are recorded at FCV. In Part B of Table 4 we use concurrent income to predict adversity reported at the same follow-up. All associations are strong and significant, with the exception of the 21-year follow-up where income is maternally-reported family income, but adversity is reported by the offspring. At the 21-year follow-up the association between family income from offspring reports of adverse life experiences is statistically significant but relatively weak. At the 30-year follow-up both income and adversity are reported by the offspring and the associations are stronger and consistent with those observed from maternal reports over the first three phases of data collection (FCV, 5 and 14 years).

**Table 4:** Family income predicting adversity (odds ratios, 95% confidence intervals).

		Part A: Family income at FCV		Part B: Family income at each phase	
	Ref = Higher	Unadjusted	Adjusted*	unadjusted	Adjusted*
		Lowest	Lowest	Lowest	Lowest
**Adversity at FCV**					
Low (n = 1783)	*1*	*1*	*1*	*1*	*1*
Borderline (n = 176)	*1*	**2.03 (1.46, 2.83)**	**1.71 (1.20, 2.44)**	**2.03 (1.46, 2.83)**	**1.71 (1.20, 2.44)**
High (n = 113)	*1*	**3.53 (2.38, 5.26)**	**2.64 (1.72, 4.07)**	**3.53 (2.38, 5.26)**	**2.64 (1.72, 4.07)**
**Adversity at 5 years**					
Low (n = 1596)	*1*	*1*	*1*	*1*	*1*
Borderline (n = 219)	*1*	**1.70 (1.26, 2.30)**	**1.55 (1.13, 2.12)**	**2.36 (1.72, 3.22)**	**2.26 (1.63, 3.13)**
High (n = 129)	*1*	**1.64 (1.11, 2.41)**	1.43 (.96, 2.17)	**3.59 (2.47, 5.21)**	**3.64 (2.44, 5.43)**
**Adversity at 14 years**					
Low (n = 1724)	*1*	*1*	*1*	*1*	*1*
Borderline (n = 189)	*1*	1.32 (.95, 1.83)	1.18 (.83, 1.66)	**3.83 (2.75, 5.34)**	**3.78 (2.69, 5.30)**
High (n = 173)	*1*	**1.87 (1.34, 2.59)**	1.38 (.96, 1.97)	**4.67 (3.33, 6.53)**	**4.25 (2.99, 6.05)**
**Adversity at 21 years**					
Low (n = 1623)	*1*	*1*	*1*	*1*	*1*
Borderline (n = 234)	*1*	1.20 (.88, 1.63)	1.07 (.77, 1.47)	1.33 (.98, 1.82)	1.26 (.91, 1.74)
High (n = 230)	*1*	**1.98 (1.48, 2.66)**	**1.56 (1.14, 2.14)**	**1.66 (1.21, 2.26)**	**1.49 (1.07, 2.07)**
**Adversity at 30 years**					
Low (n = 1722)	*1*	*1*	*1*	*1*	*1*
Borderline (n = 147)	*1*	1.21 (.84, 1.75)	1.05 (.71, 1.55)	**2.67 (1.84, 3.87)**	**2.55 (1.74, 3.74)**
High (n = 129)	*1*	**1.64 (1.12, 2.39)**	1.47 (.98, 2.19)	**3.88 (2.64, 5.68)**	**3.92 (2.65, 5.79)**

* Adjusted for sociodemographic variables of mother’s age, mother education, and marital status at first clinic visit; Odds ratios in bold are significantly different to those of the reference category (P < .05).

Table 5 examines the alternative causal sequences for the association between family poverty and experiences of adversity (with time lag). Low family income predicts subsequent levels of adversity for every unadjusted and adjusted estimate. These odds ratios suggest an association of moderate strength (less than 2x), but all associations are consistent and statistically significant. Further, adversity, at each phase of data collection, predicts subsequent phase family income. These associations are consistently strong (greater than 2x).

**Table 5:** Family income predicting adversity (ref = low) and adversity predicting family income (ref = higher income), with lag.

	odds ratios (95% Confidence Intervals)	
	Unadjusted	Adjusted*
Poverty FCV→ adversity 5 years (N = 1856)	**1.68 (1.31–2.16)**	**1.59 (1.22–2.06)**
Poverty 5 years→ adversity 14 years (N = 1897)	**1.93 (1.48–2.51)**	**1.71 (1.30–2.26)**
Poverty 14 years→ adversity 21 years (N = 2051)	**1.92 (1.49–2.47)**	**1.81 (1.39–2.35)**
Poverty 21 years→ adversity 30 years (N = 1833)	**1.91 (1.44–2.52)**	**1.81 (1.36–2.42)**
Adversity FCV→ Poverty 5 years (N = 1884)	**2.38 (1.80–3.17)**	**2.30 (1.71–3.10)**
Adversity 5 years→ Poverty 14 years (N = 1909)	**1.85 (1.40–2.45)**	**1.87 (1.41–2.50)**
Adversity 14 years→ Poverty 21 years (N = 1913)	**2.40 (1.88–3.08)**	**2.47 (1.91–3.19)**
Adversity 21 years→ Poverty 30 years (N = 2030)	**2.72 (2.14–3.46)**	**2.72 (2.12–3.49)**

* Adjusted for sociodemographic variables of mother’s age, mother education, and marital status at first clinic visit; Odds ratios in bold are significantly different to those of the reference category (P < .05).

## Discussion

Poverty is associated with a very wide range of diminished life prospects for those affected. While the adverse consequences of poverty are well understood, poverty itself is less well understood particularly its form and duration.

We report here the findings from a 30-year prospective study of poverty and adversity experiences of mothers and their children. Pregnant women were recruited early in pregnancy, and the experiences of the children of these mothers who were living in poverty over the first 30 years of life are described. Poverty was determined by reference to the Australian poverty line, with a similar standard used over the 30 years of follow-up.

Over the 30-year period (there are 5 data collections reported); we find very little evidence that there is a persistent “underclass”. Very few families experienced poverty at every phase of the data collection. Further, we find that about half of the children in this study have experienced poverty at some stage of their early growth and development. Those living in poverty at the 30-year follow-up are more likely to have experienced multiple episodes of poverty in their families of origin. However, the vast majority of those living in poverty at 30 years, experience childhood/adolescent poverty only somewhat more often than their 30 year old counterparts who were not poor at 30 years of age. Adverse life experiences were found to be strongly associated with poverty and there appears to be a causal link between these adverse life experiences and family poverty. With regard to life course experiences of adversity, children reared in a home characterised by higher levels of adversity are themselves only somewhat more likely to experience adversity when they are adults.

Our research points to a bidirectional relationship whereby low family income is associated with increased risk of adverse life events and the experience of adverse life events is associated with an increased risk of low family income. The latter causal pathway appears to be stronger than the former.

The finding that poverty at first clinic visit is a statistically significant but not strong predictor of subsequent adversity is important. It suggests that factors other than initial family poverty lead to subsequent adversity. The association between concurrent poverty and adversity suggests that such events as marital breakdown and unemployment may be the prime causes of concurrent poverty. The correlation of incomes over the phases of the life course is moderate to weak. This suggests that while income at one point in time predicts income at other points in time, the prediction is not a strong one and there appear to be substantial proportions in our sample whose relative income varies substantially within a generation over time as well as across generations. Arguably, the 30 years that have elapsed since the study started has been characterised by a widening range of life options (improved education, increased employment for women, a rapidly changing labour force) which have meant that women who experience periods of poverty (and their children) manifest a good level of economic mobility.

In this study those living in poverty were primarily single mothers, the unemployed and those receiving sickness or disability welfare benefits. In part, this reflects the adequacy (or more correctly inadequacy) of welfare benefits provided in the Australian context. However, this also suggests that single women who re-partner and those unemployed who obtain work, experience significant changes in their economic circumstances. Of course, repartnering may not be a permanent change and employment might be casual and episodic.

When considering the broad range of negative consequences of poverty, it may be important to know the stage of the life course and duration of time that an affected person may be exposed to poverty. It may also be important to distinguish the possible consequences of poverty, from the related levels of adversity experienced by that same person. There may be a need for different policies to address poverty and adversity. This will be even more the case in circumstances where life adversities (for example marital and employment problems) are the cause of the poverty.

When we consider the period of time that elapses from recruitment of the sample to the final follow-up, the associations (poverty correlated with poverty over time; adversity correlated with adversity over time) appear to decline in magnitude over time. Family income at FCV correlates with family income at the 5-year follow-up (Tau B = 0.35), but only B = 0.11 with income at the 30-year follow-up. Similarly, life adversity at FCV correlates with life adversities at the 5-year follow-up (Tau B = 0.20), but only Tau B = 0.03 (NS) with adversity at the 30-year follow-up. This suggests that adult children’s economic circumstances are only occasionally a reflection of the economic circumstances of their family of origin, and adversity in childhood is related to adversity in adulthood.

## Limitations

The findings need to be interpreted in the context of the limitations of the study. We only have complete data on about 2000 respondents. While it is the case that those lost to follow-up are disproportionately of low family income and experience higher levels of adversity, we have reason to believe the findings are not biased [32]. In any event the loss of those with lower incomes and higher levels of adversity should not reduce the strength of the associations we have observed [29].

There are also variable and often long gaps in time between phases of data collection. Where studies have more frequent follow-up the results might differ from ours. This should not affect the findings involving the simultaneous measurement of family income and adversity, but could impact on the prediction models.

It is, of course, possible that those who move in and out of poverty over their life course, experience changes in income which are of a modest magnitude. No longer being in poverty might mean a modest increase in income.

The list of life adversities varied between phases of the data collection. It is to be expected that life adversities to which people are subjected, will change at different stages of the life course. The adversities reported by the mother at the first three phases of the study are likely to have impacts on the child during sensitive periods of that child’s development, while the adversities recorded at the 21- and 30-year follow-ups are reported by offspring and, arguably, more directly experienced by offspring. In view of the high rate of adversities reported by both mothers and their offspring, we suggest that any list of adversities reflects a subset of a broader range of life events. Our measure of life adversities should not be interpreted in terms of the specific events that are listed, but rather considered as markers of a larger number of life changes that are likely stressful to individuals. From this perspective the specific, listed adversities are likely to be less important than the extent to which they reflect a life course influenced by repeated experiences of a wide range of life adversities.

## Conclusions

Poverty and adversity are commonly experienced by children during their life stages which involve growth and development. However, the findings also suggest that a relatively small proportion of children experience chronic and persistent poverty over their period of childhood and adolescence. For this population sample there is little evidence of an “underclass”. Rather we find evidence of substantial economic mobility both within a single generation and across generations.

Poverty and life adversities are strongly related, and the relationship appears to be bidirectional. Children reared in poverty-affected households experience a wide range of adversities. Households which have a history of adversities tend to more often also experience poverty. While poverty experienced earlier in the life course predicts subsequent poverty, many families appear to move in and out of poverty over the period the child is being reared. Interestingly poverty experienced early in the child’s life course does not independently predict adversity at later stages in the child’s life course.

In view of the evidence showing a strong association between poverty and child health and developmental outcomes, there is a need to know more about the pattern of exposure to poverty and its impact on subsequent child health and development. There is also a need to learn more about the role played by life adversities as these contribute to child health and development outcomes. A third gap in the knowledge base concerns the factors that link poverty and child outcomes. Family poverty is likely a predictor of a wide range of attitudes, beliefs and behaviours which themselves may impact on child outcomes. It is this causal sequence that needs to be understood. Programs and interventions intended to reduce poverty need to be better informed about the extent to which life adversities and poverty are related over the child’s early life course.

## Supporting information

S1 Fig Details of loss to follow up. (DOCX)

S1 Table Distribution of family income (per week) categories at each follow up. (DOCX)

S2 Table Reliability coefficients for adversity items at each follow up. (DOCX)

S3 Table Life event/adversity at each follow up. (DOCX)

S1 File Input data. (PDF)
