# Supplementary material for: The inter- and intra- generational transmission of family poverty and hardship (adversity): A prospective 30 year study
Source: PLoS One. 2018 Jan 23;13(1):e0190504. doi: 10.1371/journal.pone.0190504 (PMC5779648; doi:10.1371/journal.pone.0190504)
Supplement: S1 Fig — (DOCX) [file pone.0190504.s001.docx]

**S1 Fig. Details of loss to follow-up**

| **Phase of study (year)** | **Numbers (percent retained)** |
| --- | --- |

7223

5308 (73.5%)

5216 (72.2%)

3805 (52.7%)

2900 (40.0%)

5 Year Follow-up

(1986-1988)

Birth^*^

(1981-1984)

21 Year Follow-up

(2001-2004)

14 Year Follow-up

(1995-1997)

30 Year Follow-up

(2010-2014)

^*^Selection criteria: live singleton baby born at the study hospital – a small number of children who have deceased have not been removed from sample calculations.
