# Supplementary material for: The inter- and intra- generational transmission of family poverty and hardship (adversity): A prospective 30 year study
Source: PLoS One. 2018 Jan 23;13(1):e0190504. doi: 10.1371/journal.pone.0190504 (PMC5779648; doi:10.1371/journal.pone.0190504)
Supplement: S1 Table — (DOCX) [file pone.0190504.s002.docx]

**S1 Table. Distribution of family income (per week) categories at each follow up**

| **Categories** | **FCV ^a^** | **5 year**  **follow up ^b^** | **14 year follow up ^c^** | **21 year follow up ^d^** | **30 year follow up ^e^** |
| --- | --- | --- | --- | --- | --- |
| **$0 - 49** | 21 (1.1%) |  |  |  |  |
| **$50-99** | 72 (3.6%) | 7 (.4%) |  |  |  |
| **$100-199** | 474 (23.8%) | 128 (6.7%) | 28 (1.4%) |  |  |
| **$200-299** | 774 (38.9%) | 274 (14.4%) | 111 (5.4%) | 181 (9.5%) |  |
| **$300-399** | 373 (18.7%) | 434 (22.9%) | 213 (10.4%) | 149 (7.8%) |  |
| **$400-499** | 185 (9.3%) | 456 (24%) | 250 (12.2%) | 178 (9.3%) |  |
| **$500-599** | 93 (4.7%) | 313 (16.5%) | 308 (15%) | 167 (8.7%) |  |
| **$600-699** |  | 285 (15%) | 298 (14.5%) | 122 (6.4%) |  |
| **$700-799** |  |  | 843 (41.1%) | 172 (9.0%) | 265 (13.1%) |
| **$800-999** |  |  |  | 267 (14.0%) | 124 (6.1%) |
| **$1000-1499** |  |  |  | 435 (22.7%) | 426 (21.0%) |
| **$1500-1999** |  |  |  | 242 (12.7%) | 359 (17.7%) |
| **$2000-2499** |  |  |  |  | 306 (15.1%) |
| **$2500-2999** |  |  |  |  | 257 (12.7%) |
| **$3000+** |  |  |  |  | 293 (14.4%) |

^a^ Income below $ 200 per week= **lowest** (n=567; 28.5%); Higher income= **ref** (n= 1425; 71.5%)

**^b^** Income below $ 300 per week= **lowest** (n=409; 21.5%); Higher income= **ref** (n= 1488; 78.5%)

**^c^** Income below $ 400 per week= **lowest** (n=352; 17.2%); Higher income = **ref** (n= 1699; 82.8%)

**^d^** Income below $ 500 per week= **lowest** (n=508; 26.6%); Higher income = **ref** (n= 1405; 73.4%)

**^e^** Income below $ 1000 per week = **lowest** (n=389; 19.2%); Higher income = **ref** (n= 1641; 80.8%)

**Abbreviations**: FCV, first clinic visit
