# Supplementary material for: The inter- and intra- generational transmission of family poverty and hardship (adversity): A prospective 30 year study
Source: PLoS One. 2018 Jan 23;13(1):e0190504. doi: 10.1371/journal.pone.0190504 (PMC5779648; doi:10.1371/journal.pone.0190504)
Supplement: S2 Table — (DOCX) [file pone.0190504.s003.docx]

**S2 Table. Reliability Coefficients for Adversity Items at each follow up**

| **Items** | **Corrected Item-Total Correlation** |
| --- | --- |
| **FCV : Cronbach's Alpha = 0.51** |  |
| Someone close died or been seriously ill | .095 |
| Big problem with own health | .150 |
| Serious disagreements with partner | .295 |
| Serious financial problems | .420 |
| Partner major change in job | .253 |
| Mother major change in job situation | .163 |
| Serious problems with housing | .325 |
| Self or partner problem with law | .235 |
| **5-year follow up: Cronbach's Alpha = 0.45** | |
| Mother or partner major changes in job | .168 |
| Mother or partner trouble at work | .249 |
| Problems with the police | .211 |
| Death of close friend or partner | .092 |
| Changed partners since birth | .109 |
| Serious disagreements with partner | .312 |
| Serious problems with housing | .221 |
| Mother or partner treated for mental problems | .259 |
| **14-year follow up: Cronbach's Alpha = 0.58** | |
| Mother or partner lost job last five years | .223 |
| Mother or partner problems under police last five years | .281 |
| Marital breakdown last 5 years (divorce/separation/change partner) | .335 |
| Trouble finding somewhere to live | .217 |
| Mother or partner treated for mental problems last 5 years | .318 |
| Serious financial problems last 5 years | .373 |
| Mother or partner serious illness/injury last 5 years | .204 |
| Violence in marital relationship last 5 years | .379 |
| **21-year follow up: Cronbach's Alpha = 0.48** |  |
| Age child left home 16 years or less | .361 |
| Living in area with social problems | .221 |
| Victim of crime 2+ | .165 |
| Involved in traffic accidents 2+ (removed from scale) | .014 |
| Been hurt or beaten up | .194 |
| physical disease told by a doctor | .149 |
| mental disease told by a doctor | .223 |
| given warning by the police/been to court | .211 |
| Unemployment (= no job) | .161 |
| Living in hostel, Shed, Tent, Mobile home, Temporary dwelling, Boat | .103 |
| low education (incomplete secondary and less) | .272 |
| Single parent (raising a child + no partner) | .202 |
| Marital breakdown (separation, divorced, widow) | .075 |
| **31-year follow up: Cronbach's Alpha = 0.57** |  |
| Serious illness or injury to self/partner | .260 |
| Death of close family members/relatives/friends | .209 |
| Separation/Ended steady relationship | .309 |
| Serious problem with a close friend, neighbor, relative | .268 |
| Unemployment/sacked from job | .322 |
| Major financial crisis | .357 |
| Problems with police requiring court appearance | .336 |
| Something valuable lost or stolen | .255 |

**Abbreviations**: FCV, first clinic visit
