# Supplementary material for: The inter- and intra- generational transmission of family poverty and hardship (adversity): A prospective 30 year study
Source: PLoS One. 2018 Jan 23;13(1):e0190504. doi: 10.1371/journal.pone.0190504 (PMC5779648; doi:10.1371/journal.pone.0190504)
Supplement: S3 Table — (DOCX) [file pone.0190504.s004.docx]

**S3 Table. Life event/adversity at each follow up**

|  | **Life adversity at different phases (n (%))** | | | | |
| --- | --- | --- | --- | --- | --- |
|  | **FCV** | **5 years** | **14 years** | **21 years** | **30 years** |
| **Scores** |  |  |  |  |  |
| 0 | 852 (41.1%) | 431 (22.2%) | 787 (37.7) | 484 (23.2%) | 814 (40.7%) |
| 1 | 632 (30.5%) | 708 (36.4%) | 613 (29.4%) | 615 (29.5%) | 578 (28.9%) |
| 2 | 299 (14.4%) | 457 (23.5%) | 324 (15.5%) | 524 (25.1%) | 330 (16.5%) |
| 3 | 176 (8.5%) | 219 (11.3%) | 189 (9.1%) | 234 (11.2%) | 147 (7.4%) |
| 4 | 75 (3.6%) | 86 (4.4%) | 96 (4.6%) | 141 (6.8%) | 73 (3.7%) |
| 5 | 26 (1.3%) | 30 (1.5%) | 54 (2.6%) | 50 (2.4%) | 24 (1.2%) |
| 6 | 11 (.5%) | 8 (.4%) | 17 (.8%) | 22 (1.1%) | 15 (.8%) |
| 7 | 0 | 5 (.3%) | 5 (.2%) | 12 (.6%) | 7 (.4%) |
| 8 | 1 (.0%) | - | 1 (.0%) | 4 (.2%) | 10 (.5%) |
| 9 |  |  |  | 1 (.0%) |  |
| **Categories** |  |  |  |  |  |
| Low (0-2) | 1783 (86.1%) | 1596 (82.1%) | 1724 (82.6%) | 1623 (77.8%) | 1722 (86.2) |
| Borderline (3) | 176 (8.5%) | 219 (11.3%) | 189 (9.1%) | 234 (11.2%) | 147 (7.4%) |
| High (4+) | 113 (5.5%) | 129 (6.6%) | 173 (8.3%) | 230 (11.0%) | 129 (6.5%) |
| **Total** | **2087** | **2087** | **2087** | **2087** | **2087** |
