# Supplementary material for: The inter- and intra- generational transmission of family poverty and hardship (adversity): A prospective 30 year study
Source: PLoS One. 2018 Jan 23;13(1):e0190504. doi: 10.1371/journal.pone.0190504 (PMC5779648; doi:10.1371/journal.pone.0190504)
Supplement: S1 File — (PDF) [file pone.0190504.s005.pdf]

Supplementary file: input data

Data Dictionary

Variable name	Label	Value label
Age.m	Age mother at FCV	1 13-19<br>2 20-35<br>3 35+
Edu.m	Maternal education at FCV	1 Incomplete high school<br>2 Complete high school<br>3 Post-high
Marital.m	Mother's Marital status at FCV	1 Single<br>2 Living together<br>3 Married<br>4 Sep-div-wid
A.fcv.s	Adversity at FCV (mother report): scale	Ranged from 0 to 8
A.5.s	Adversity at 5 years (mother report): scale	
A.14.s	Adversity at 14 years (mother report): scale	
A.21.s	Adversity at 21 years (Child report): scale	
A.30.s	Adversity at 30 years (Child report): scale	
A.fcv.gr	Adversity at FCV (mother report): groups	1 low (0-2)<br>2 borderline (3)<br>3 high (4+)
A.5.gr	Adversity at 5 years (mother report): groups	
A.14.gr	Adversity at 14 years (mother report): groups	
A.21.gr	Adversity at 21 years (Child report): groups	
A.30.gr	Adversity at 30 years (Child report): groups	
d.in.fcv	Detailed family income at FCV	1 0 - $49 pw<br>2 $50-99<br>3 $100-199<br>4 $200-299<br>5 $300-399<br>6 $400-499<br>7 $500+

d.in.5	Detailed family income at 5 years	1<br>2<br>3<br>4<br>5<br>6<br>7	$0-99<br>$100-199<br>$200-299<br>$300-399<br>$400-499<br>$500-599<br>$600+
d.in.14	Detailed family income at 14 years	1<br>2<br>3<br>4<br>5<br>6<br>7	0-$199 pw<br>$200-299<br>$300-399<br>$400-499<br>$500-599<br>$600-699<br>$700 or more
d.in.21	Detailed family income (mother report) at 21 years	1<br>2<br>3<br>4<br>5<br>6<br>7<br>8<br>9	0-$299 per wk<br>$300-$399 per wk<br>$400-$499 per wk<br>$500-$599 per wk<br>$600-$699 per wk<br>$700-$799 per wk<br>$800-$999 per wk<br>$1,000-$1,499 per wk<br>$1,500 or more per wk
d.in.30	Detailed family income (child report) at 30 years	1<br>2<br>3<br>4<br>5<br>6<br>7	$700-799<br>$800-999<br>$1000-1499<br>$1500-1999<br>$2000-2499<br>$2500-2999<br>$3000+
p.fcv	Poverty at FCV	0<br>1	higher<br>poor
p.5	Poverty at 5 years		
p.14	Poverty at 14 years		
p.21	Poverty at 21 years		
p.30	Poverty at 30 years		

age.m	edu.m	marital.r	A.fcv.s	A.5.s	A.14.s	A.21.s	A.30.s	A.fcv.gr	A.5.gr	A.14.gr	A.21.gr	A.30.gr	d.in.fcv	d.in.5	d.in.14	d.in.21	d.in.30	p.fcv	p.5	p.14	p.21	p.30
3	2	3	1	2	3	1	1	1	1	2	1	1	4	2	1	1	3	0	1	1	1	0
2	1	3	0	1	2	4		1	1	1	3		4	5	6	8		0	0	0	0	
2	3	2	1					1					6					0				
2	2	3	2	0	0	0	2	1	1	1	1	1	4	4	6	7	6	0	0	0	0	0
2	3	3	0	1	1	0	1	1	1	1	1	1	6	7	7		5	0	0	0		0
2	2	3	0	0	1	2	0	1	1	1	1	1	6	5	4	8	6	0	0	0	0	0
2	3	3	3		2	0		2		1	1		5		7	8		0		0	0	
2	2	3	0	0	0	5		1	1	1	3		4	4	5	2		0	0	0	1	
2	2	3	1					1					4					0				
1	2	1	0	1	0	1		1	1	1	1		4	4	7	9		0	0	0	0	
1	2	2	0	0	0	2		1	1	1	1		3	3	5			1	1	0		
2	2	3	3					2														
2	2	3	0	1	1	2	0	1	1	1	1	1	3	5	2	7	5	1	0	1	0	0
2	2	3	1	2	2			1	1	1			3	4	2	2		1	0	1	1	
1	2	2	3	2	4	0	0	2	1	3	1	1	3	3	4		5	1	1	0		0
1	1	1	0	0	0	1		1	1	1	1			1	3	3			1	1	1	
2	2	3	3	2		2		2	1		1		3	3		7		1	1		0	
1	1	2	2	1		1		1	1		1		3	3		6		1	1		0	
2	2	3	0	1	0			1	1	1			3	4	4	8		1	0	0	0	
1	2	3	0	3				1	2				4	2				0	1			
2	1	3	1					1					3					1				
2	2	3	3	3			1	2	2			1	4	4		8	4	0	0		0	0
2	3	3	1	1				1	1				5	3				0	1			
2	2	3	1					1					3					1				
2	1	3	1	2	0	1		1	1	1	1		4	5	7	7		0	0	0	0	
2	2	3	1	1		0		1	1		1		3	5		8		1	0		0	
2	1	3	1	0		1		1	1		1		3	5		1		1	0		1	
1	2	3	2	5	4	4		1	3	3	3		3	3	4	2		1	1	0	1	
2	1	3	0	4	3	3		1	3	2	2		6	6	7	5		0	0	0	0	
2	2	3	1	1	0	0		1	1	1	1		3	6	7	5		1	0	0	0	
2	2	3	1			3	0	1			2	1	3			7	1	1			0	1
1	2	3	4		4		6	3		3		3	3		6		1	1		0		1
1	3	4	0					1					2					1				
2	2	3	0	4	3	5		1	3	2	3		4	6	4			0	0	0		
2	3	3	0	2	2	3	1	1	1	1	2	1	5	6	7	8	3	0	0	0	0	0
2	2	3	4					3					3					1				
2	2	3	2	2	0	5		1	1	1	3		5	5	7	8		0	0	0	0	
2	2	3	1	1	2			1	1	1			5	5	3			0	0	1		
2	1	3	1	1	3	2	2	1	1	2	1	1	3	4	3	8	4	1	0	1	0	0
2	2	3	2	2	2	1	1	1	1	1	1	1	3	3	4	8	2	1	1	0	0	1

2	1	3	2	4	0			1	3	1			3	2	2			1	1	1		
2	1	3	4	0	0	0	2	3	1	1	1	1	3	2	2	1	3	1	1	1	1	0
2	2	3	1	0	3	2	3	1	1	2	1	2	3	2	6	2	2	1	1	0	1	1
2	2	2	1	3	2	5		1	2	1	3		6	4	6	2		0	0	0	1	
2	1	3	2	3	3	3	0	1	2	2	2	1	3	3	2	6		1	1	1	0	
2	2	3	0	1	0	1	2	1	1	1	1	1	3	6	7	8	3	1	0	0	0	0
2	2	3	0	3	0	6		1	2	1	3		4	5	5	1		0	0	0	1	
2	3	3	1	1	4			1	1	3			4	3	3		7	0	1	1		0
2	3	3	0	1	0		2	1	1	1		1	7	5	7	9	7	0	0	0	0	0
2	1	3	1	5				1	3				4	4				0	0			
3	3	3	0	2				1	1				4	4				0	0			
2	1	3	1	2	1	2		1	1	1	1		4	4	3	3		0	0	1	1	
1	2	3	0	1	1		2	1	1	1		1	3	3	4	1	7	1	1	0	1	0
2	2	3	1	0	2			1	1	1			3	5	6	1		1	0	0	1	
2	2	3	2					1					4					0				
2	2	3	2	2		3		1	1		2		3	3				1	1			
2	2	3	0	3	2		1	1	2	1		1	4	5	5		5	0	0	0		0
2	2	3	1	4		1		1	3		1		4	6		7		0	0		0	
2	2	3	2	1	0	1		1	1	1	1		3	4	6	6		1	0	0	0	
1	1	3	1	1	1	2		1	1	1	1		6	2	7			0	1	0		
2	3	3	2	2	2			1	1	1			4	6	7	1		0	0	0	1	
3	2	3	3	2				2	1				4	6				0	0			
2	2	3	0	1	0			1	1	1			4	4	6	4		0	0	0	0	
2	3	3	0	0	0	0	0	1	1	1	1	1	5	4	5	6	4	0	0	0	0	0
2	2	3	0	2	2	0	0	1	1	1	1	1	4	4	7	5	4	0	0	0	0	0
2	2	2	3	1	4	7		2	1	3	3		6	4	7			0	0	0		
1	2	3	0	2	2	3	0	1	1	1	2	1	5	5	4	3	4	0	0	0	1	0
2	2	3	1	2	2	3	0	1	1	1	2	1	6	7	7	8	7	0	0	0	0	0
2	2	3	0					1					4					0				
2	2	3	1	2	0	2	3	1	1	1	1	2	5	6	7	9	3	0	0	0	0	0
2	2	2	0					1					4					0				
2	3	3	0	1	0	1		1	1	1	1		5	4	7	8		0	0	0	0	
1	2	3	4	3	1	4		3	2	1	3		7	7	7	8		0	0	0	0	
2	3	3	0	0	0	2	1	1	1	1	1	1	4	4	4	9	5	0	0	0	0	0
1	2	1	2					1					1			7		1			0	
2	2	1	4	4				3	3				2	3		5		1	1		0	
2	2	3	0	0	2	1		1	1	1	1		5	4	7	7		0	0	0	0	
2	2	2	1	2	0	1	1	1	1	1	1	1	3	6	6	4	3	1	0	0	0	0
2	3	3	0	1	2	2	0	1	1	1	1	1	4	4	5	6	7	0	0	0	0	0
2	2	3	2	0	0	2	0	1	1	1	1	1	4	3	7	7	7	0	1	0	0	0
2	2	3	3	3	1			2	2	1			4	4	5	2		0	0	0	1	

2	2	3	0	0	1	0	2	1	1	1	1	1	4	4	6		4	0	0	0		0
2	3	3	1	1	5	2	1	1	1	3	1	1	3	4	5	4	4	1	0	0	0	0
2	2	2	1	3	0	4		1	2	1	3		3	2	7	8		1	1	0	0	
2	1	3	0					1					3					1				
1	2	3	0					1					5					0				
2	2	3	0	0	0			1	1	1			3	6	7			1	0	0		
2	2		1	1	1	1		1	1	1	1		3	5	7	5		1	0	0	0	
3	1	3	4	1				3	1				4	3				0	1			
2	2	3	4	2				3	1				5	7				0	0			
2	1	3	1	1	0	4	3	1	1	1	3	2	4	4	5	8	5	0	0	0	0	0
2	2	3	0	0	0	3	0	1	1	1	2	1	3	4	5	5	6	1	0	0	0	0
2	3	3	2	5	3	1		1	3	2	1		4	6	7	9		0	0	0	0	
2	3	3	0	0	0	3		1	1	1	2		3	4	7	8		1	0	0	0	
2	2	3	0	1	0		1	1	1	1		1	4	4	5	3	4	0	0	0	1	0
2	2	3	1	1	0	1		1	1	1	1		4	4	5	7		0	0	0	0	
2	3	3	2	4	2	2	1	1	3	1	1	1	5	2	3	7	7	0	1	1	0	0
3	3	3	2					1					3					1				
2	3	3	0	1	0	1	0	1	1	1	1	1	5	5	6	7	3	0	0	0	0	0
2	1	3	0	1	1		3	1	1	1		2	4	5	4		3	0	0	0		0
1	1	2	2	2	4	3	0	1	1	3	2	1	3	4	7	8	4	1	0	0	0	0
1	1	2	0					1					5					0				
2	2	3	0	2	6			1	1	3			4		7			0		0		
2	1	2	3	2				2	1				3	3				1	1			
2	2	3	0					1					3					1				
1	2	3	0		2	4		1		1	3		4		3	7		0		1	0	
1	2	3	2	2	4	0	0	1	1	3	1	1	4		2	1	4	0		1	1	0
2	3	3	0	0	1	0	1	1	1	1	1	1	4	4	4	4	4	0	0	0	0	0
2	1	3	0	1				1	1				4	3				0	1			
2	2	3	1	2	0	4		1	1	1	3		4	5	6	7		0	0	0	0	
1	1	1	1	2				1	1				1	2				1	1			
2	2	3	1	1	0	2		1	1	1	1		4	4	4	9	4	0	0	0	0	0
2	2	3	0	1	0	3		1	1	1	2			4	5	2			0	0	1	
2	2	3	0	1	1	2		1	1	1	1			5	5	3			0	0	1	
1	1	2	2	1	2	3		1	1	1	2		4	3	2			0	1	1		
2	2	3	1					1					4			9		0			0	
2	2	3	4	2	2	1	2	3	1	1	1	1	4		5		5	0		0		0
2	2	2	1	1	3	1		1	1	2	1		3	2				1	1			
2	1	3	1	1	3	3	1	1	1	2	2	1	4	5	7	6	7	0	0	0	0	0
2	1	3	1					1					3					1				
1	2	1	2					1					1					1				
2	2	3	0		0	0	0	1		1	1	1	4		7	8	5	0		0	0	0

2	2	1	2					1					2					1				
2	2	3	1	3	0	2	1	1	2	1	1	1	4	7	7	9	1	0	0	0	0	1
2	1	3	0		1			1		1			3			1		1			1	
2	1	3	0	0	1	0	0	1	1	1	1	1	4	4	4	2	6	0	0	0	1	0
2	1	3	0					1					3					1				
2	3	3	0	2	0	2		1	1	1	1		3	6			4	1	0			0
2	2	3	2	0	1	2	1	1	1	1	1	1	6	6	4	1	3	0	0	0	1	0
3	2	3	1		1	2		1		1	1		5		4			0		0		
3	1	3	0	1	1	0	1	1	1	1	1	1	5	2	1	1	4	0	1	1	1	0
2	2	3	7	3	2	3		3	2	1	2			2	7	2			1	0	1	
3	3	3	1	0	2	2	1	1	1	1	1	1	4	5	5	6	3	0	0	0	0	0
1	2	1	4	3	4			3	2	3			4	4	4	7		0	0	0	0	
1	3	1	2	0	0	1		1	1	1	1		3	3	7	9	7	1	1	0	0	0
1	2	2	4	0	3	2	3	3	1	2	1	2	4	4	3	2	4	0	0	1	1	0
1	1	3	3	2	1	3	2	2	1	1	2	1	3	3	5	4	4	1	1	0	0	0
2	2	3	0					1					3					1				
1	2	2	1					1					1					1				
1	3	2	0	5	3	1	0	1	3	2	1	1	4	7	7	7	7	0	0	0	0	0
2	1	3	1	4	2	2	1	1	3	1	1	1	3	2	2	3	6	1	1	1	1	0
2	2	3	3	2				2	1				3	4				1	0			
1	2	3	1		1	5		1		1	3		4		2	5		0		1	0	
2	2	3	1			2		1			1		4			7		0			0	
1	2	3	1	3	0	4		1	2	1	3		3	3	6			1	1	0		
1	2	3	1	3	2	4	0	1	2	1	3	1	3	2	2	4	3	1	1	1	0	0
2	2	3	2	0	0	2		1	1	1	1		4	4	6	4		0	0	0	0	
2	3	4	2	2	3			1	1	2			2	2	2			1	1	1		
1	2	3	5	0	0			3	1	1			2	5	7			1	0	0		
2	1	3	2	1	1	3	1	1	1	1	2	1		2	3	1	5		1	1	1	0
2	2	3	0					1					3					1				
2	2	3	3	0	4			2	1	3			4	6	3	4		0	0	1	0	
1	2	3	3	0	6	1	2	2	1	3	1	1		3	5	2	7		1	0	1	0
2	1	3	1	0	1	3		1	1	1	2		4	4	6	7		0	0	0	0	
2	2	2	3	2	3	3		2	1	2	2		3	6	5			1	0	0		
2	3	3	0	4	3	4	1	1	3	2	3	1	3	2	4	9	3	1	1	0	0	0
2	3	3	0	1	0	2		1	1	1	1		5	5	7	8		0	0	0	0	
2	2	3	1	2	0	0	0	1	1	1	1	1	6	5	6	7	5	0	0	0	0	0
2	2	3	2	1	0	1	1	1	1	1	1	1	4	4	7	7	2	0	0	0	0	1
2	2	3	1					1					3					1				
2	2	3	2	0	0	1	2	1	1	1	1	1	6	7	7	9	4	0	0	0	0	0
2	2	3	1	1				1	1				4	4				0	0			
1	2	1	5	2	4	3	0	3	1	3	2	1	4	4	3	3	7	0	0	1	1	0

2	2	3	1	2	1	4	2	1	1	1	3	1	4	7	4	7	1	0	0	0	0	1
2	2	3	3					2					3					1				
1	1	1	2		2	3		1		1	2		2		7	2		1		0	1	
2	2	3	1	3	3	3	0	1	2	2	2	1	4	2	4	2	1	0	1	0	1	1
1	2	1	0					1					4					0				
2	2	1	4	0	2			3	1	1			1	2	3			1	1	1		
1	1	2	1	1	2	5		1	1	1	3		4	4	2	4		0	0	1	0	
2	1	3	0	1	1	2		1	1	1	1		3	3	4			1	1	0		
2	2	3	2	0	0	1		1	1	1	1		4	4	6	6		0	0	0	0	
2	1	3	0	0	0	1		1	1	1	1		3	3	3	7		1	1	1	0	
1	2	3	0		1	1	0	1		1	1	1	3		5	5	3	1		0	0	0
1	2	2	1					1					3					1				
2	3	3	0	1	0			1	1	1			4	7	7			0	0	0		
2	2	3	1	1	1	0	0	1	1	1	1	1	4	4	5	4	5	0	0	0	0	0
2	2	3	2	3	4			1	2	3			3	4	2			1	0	1		
2	2	3	0	2	0			1	1	1				4	2				0	1		
2	3	3	1	1	2	0	0	1	1	1	1	1	3	4	3	3	4	1	0	1	1	0
2	2	3	1	0	0	0	1	1	1	1	1	1	5	7	6	8	4	0	0	0	0	0
2	2	3	2		0	0	1	1		1	1	1	1		6	3	7	1		0	1	0
2	2	3	0	0	2	2	1	1	1	1	1	1	5	2	7	9	6	0	1	0	0	0
1	2	3	1		4	2		1		3	1		5		2			0		1		
2	2	3	0	2	0	0		1	1	1	1		4	4	7	9		0	0	0	0	
1	2	3	4	3	3	5		3	2	2	3		3	4	6	5		1	0	0	0	
2	2	3	0	3	2	2		1	2	1	1		4	6		3		0	0		1	
2	2	4	3	2	3			2	1	2			1	6	2	2		1	0	1	1	
2	1	3	2		0	2		1		1	1		3		3	4		1		1	0	
2	2	3	2	1		1		1	1		1		3	3		3		1	1		1	
2	2	3	1					1					3					1				
2	2	3	2	3	2	2	3	1	2	1	1	2	3	6	3	2	1	1	0	1	1	1
1	2	3	0	2				1	1				3	5				1	0			
1	3	3	3		1	1	2	2		1	1	1	3		7	8	1	1		0	0	1
2	2	3	1	0	1	0	0	1	1	1	1	1	3	3	4	4	3	1	1	0	0	0
3	1	3	0	1	1	3		1	1	1	2		4	4	5	5		0	0	0	0	
2	3	3	0	0				1	1				4	4				0	0			
2	3	3	1		1			1		1			5		7			0		0		
2	1	3	2	2	0	1		1	1	1	1		4	6	6	7		0	0	0	0	
2	1	3	1	0		3	1	1	1		2	1	4	4		3	6	0	0		1	0
2	2	3	1	2	0	5		1	1	1	3		6	7	7	9		0	0	0	0	
2	2	3	1	0	2			1	1	1			4	4	3			0	0	1		
2	2	1	4					3					3					1				
2	2	3	1	3	4	3		1	2	3	2		4	3	4	6		0	1	0	0	

2	2	3	3	2	2			2	1	1			3	3	2	4		1	1	1	0	
2	2	3	0	0	1	2	1	1	1	1	1	1	7	7	6		1	0	0	0		1
3	3	4	0			2	0	1			1	1	2			1	7	1			1	0
2	1	1	1	0		4		1	1		3		3	4				1	0			
2	1	3	1	5	3	1		1	3	2	1		4	5	7	7		0	0	0	0	
2	2	3	1	0	0	0	0	1	1	1	1	1	4	3	7	8	3	0	1	0	0	0
1	2	3	5					3					2					1				
2	1	3	2	1	6	1		1	1	3	1		3	5	7	1		1	0	0	1	
2	1	3	1	1	0	0	1	1	1	1	1	1	4	5	7	9	4	0	0	0	0	0
2	2	1	3	6				2	3					2					1			
1	1	3	2	0	2			1	1	1			3	4	4	8		1	0	0	0	
2	2	1	0	1	0			1	1	1				4	4				0	0		
2	3	1	1	0	0	2		1	1	1	1		3	1	1	1		1	1	1	1	
2	3	3	2	2	1	1	4	1	1	1	1	3	4	5	7	1	5	0	0	0	1	0
2	2	3	0					1					4					0				
2	2	3	0					1					4					0				
2	1	3	1	1	3	2	2	1	1	2	1	1	4	3		2	3	0	1		1	0
2	3	3	5		2	1		3		1	1		3		3	3		1		1	1	
2	1	4	0	0				1	1				2	2				1	1			
1	2	2	3	2	3	2	1	2	1	2	1	1	3	2	3	3	4	1	1	1	1	0
2	3	3	0	1	1	1		1	1	1	1		3	5	5	5		1	0	0	0	
2	1	3	0	2	0			1	1	1			5	4	5	8		0	0	0	0	
2	2	3	0	1	0	0		1	1	1	1		4	5	7	8		0	0	0	0	
2	1	3	1	1	1			1	1	1			3	2	2	1		1	1	1	1	
2	2	3	0	0	0	2	2	1	1	1	1	1	5	6	7	7	2	0	0	0	0	1
2	1	3	1	4	4	6		1	3	3	3		3	2	5	2		1	1	0	1	
2	3	3	1	0	0	1		1	1	1	1		5	5	7	9		0	0	0	0	
2	3	3	1	3	2	1	3	1	2	1	1	2	4	4	4	6	3	0	0	0	0	0
1	2	4	1					1					2					1				
2	1	3	2					1					2					1				
2	2	3	0	1	1	1	0	1	1	1	1	1	6	6	5	9	3	0	0	0	0	0
2	3	3	1	2	1	0	0	1	1	1	1	1	4	4	7	3	2	0	0	0	1	1
2	1	3	4	2	3	0		3	1	2	1		4	3	6			0	1	0		
2	1	3	0	2	1			1	1	1			3	4	4			1	0	0		
2	2	3	1	1	1			1	1	1			4	4	7			0	0	0		
2	1	3	0	2	0	1		1	1	1	1		4	3	3	2		0	1	1	1	
1	2	3	2	2	1			1	1	1			3	5	3			1	0	1		
2	2	3	2	1	1	2	0	1	1	1	1	1	4	4	5	8	1	0	0	0	0	1
2	3	3	2	4	2	3	3	1	3	1	2	2	4	4	2	3	5	0	0	1	1	0
1	2	3	0					1					4					0				
2	3	3	1	1				1	1				3	5				1	0			

2	2	3	0	1	0	1	2	1	1	1	1	1	3	4	7	8	3	1	0	0	0	0
2	2	3	0	0	2	1	0	1	1	1	1	1		7	6		3		0	0		0
2	1	3	1	2	1			1	1	1				7	7				0	0		
2	2	3	0	1	0	1	0	1	1	1	1	1	4	7	7	7	7	0	0	0	0	0
2	1	3	1	1				1	1				4	5				0	0			
2	1	3	3					2					4					0				
2	3	3	0	2	1	0	1	1	1	1	1	1		5	7	9	4		0	0	0	0
2	2	3	1	0	0	1	0	1	1	1	1	1	4	5	7	7	5	0	0	0	0	0
2	2	3	7	2	1	2		3	1	1	1			4	7	7			0	0	0	
2	2	3	0	4	2	1	0	1	3	1	1	1	3	6	5		4	1	0	0		0
2	2	3	0	0	1			1	1	1			5	5	7	8		0	0	0	0	
2	2	2	4					3					5					0				
2	2	3	0	3	7	2	0	1	2	3	1	1	4	4	4	8	4	0	0	0	0	0
2	1	3	2	1	1	3	1	1	1	1	2	1	3	5	7			1	0	0		
2	3	3	2	1	0	0	2	1	1	1	1	1	3	4	7	4	6	1	0	0	0	0
2	2	3	0	0	0	1	0	1	1	1	1	1	3	4	6	9	4	1	0	0	0	0
2	1	3	2	3	2	5	4	1	2	1	3	3	3	4	2	2	2	1	0	1	1	1
2	2	3	0	1	0	1	1	1	1	1	1	1	4	6	7	4	3	0	0	0	0	0
2	1	2	2	2	1	3	3	1	1	1	2	2	4	5	5	4	4	0	0	0	0	0
2	2	3	0	1	0	1	4	1	1	1	1	3	6	4	7	6	3	0	0	0	0	0
2	3	3	3	3	2			2	2	1			4	4	7			0	0	0		
2	3	3	1	2	2	1		1	1	1	1		4	6	5	4		0	0	0	0	
2	3	1	4	4	2	2		3	3	1	1		2	3	7	8		1	1	0	0	
1	2	1	2	1				1	1				6	7				0	0			
1	1	1	0					1					2					1				
2	1	3	3					2					4					0				
1	1	3	2		1	2		1		1	1		3		6	2		1		0	1	
2	2	3	0	1	1	1	1	1	1	1	1	1	4	6	3	4	4	0	0	1	0	0
1	2	3	0	1	0	0	3	1	1	1	1	2	5	7	6	9	6	0	0	0	0	0
1	1	2	5					3					2					1				
2	1	3	4	1				3	1				3	4				1	0			
2	2	3	1	2	2	4	0	1	1	1	3	1	3	4	7		6	1	0	0		0
1	3	1	4	2	1			3	1	1			4	6	7			0	0	0		
2	2	3	0	0				1	1				1	4				1	0			
1	2	3	0	0	1	0		1	1	1	1		3	3	4	3		1	1	0	1	
1	2	1	1	4	2			1	3	1			1	2	1			1	1	1		
2	2	3	1	4		1		1	3		1		4	2		8		0	1		0	
2	2	3	0	2	1	0	0	1	1	1	1	1	5	3	4	4	4	0	1	0	0	0
2	1	2	0		3	2		1		2	1				5	3				0	1	
2	2	3	0	1	1	1	0	1	1	1	1	1	3	6	7	8	7	1	0	0	0	0
2	2	2	2		0			1		1			3		7	5		1		0	0	

2	2	3	1	0	0	0	1	1	1	1	1	1	5	4	5	7	3	0	0	0	0	0
1	2	3	1	2	3	5		1	1	2	3		3	4	5	7		1	0	0	0	
1	2	2	0	1				1	1				1	3				1	1			
2	2	3	1	0	0	0	0	1	1	1	1	1	6	6	7	9	5	0	0	0	0	0
2	2	3	0	0	0	1	0	1	1	1	1	1	2	3	2	3	6	1	1	1	1	0
2	1	3	1	2				1	1				6	3				0	1			
2	2	3	1					1					3					1				
2	2	4	3					2					2					1				
3	1	3	1	0	0			1	1	1			6	2				0	1			
2	1	2	0					1					6					0				
2	2	1	3	0		2		2	1		1		2	2		1		1	1		1	
2	2	3	1	1			0	1	1			1	4	5		3	3	0	0		1	0
2	2	3	2	1	2			1	1	1			4	3	3			0	1	1		
1	2	3	2					1					4					0				
2	2	3	2	2	2	5		1	1	1	3		4	6	5	2		0	0	0	1	
2	3	3	4	2	5	3		3	1	3	2		3	2	4	2		1	1	0	1	
2	2	3	2					1					3					1				
2	2	3	0	0	1	3		1	1	1	2		3	4	4	4		1	0	0	0	
2	3	2	1					1					4					0				
2	2	3	1	5	2	3		1	3	1	2		4	3	5	6		0	1	0	0	
2	2	3	1	1	0	1	2	1	1	1	1	1	4	7	7	8	7	0	0	0	0	0
2	3	3	0	1	1			1	1	1			4	5	1			0	0	1		
2	2	2	2	1	1	0	0	1	1	1	1	1	3	2	2	7	4	1	1	1	0	0
3	2	3	0	2		7		1	1		3		3	4				1	0			
2	2	3	2	0	1		8	1	1	1		3	4	4	4		1	0	0	0		1
1	2	1	1					1					2					1				
2	3	3	3	0	1	2		2	1	1	1		4	4	6	7		0	0	0	0	
2	2	3	2	2	0	3		1	1	1	2		4	5	6	2		0	0	0	1	
2	2	3	1	3	1	2	0	1	2	1	1	1	5	5	7	8	6	0	0	0	0	0
2	2	3	0	3	0			1	2	1			5	5	6	8		0	0	0	0	
2	2	3	1	3	4	3	0	1	2	3	2	1	4	5	5	8	5	0	0	0	0	0
2	2	2	1	2		2		1	1		1		5	7		8		0	0		0	
2	2	3	0	1	0	0	1	1	1	1	1	1	5	7	7	7	2	0	0	0	0	1
2	2	3	0	1	2	1		1	1	1	1		5	4	5			0	0	0		
2	2	4	2	3	2	5		1	2	1	3		4	5	7	7		0	0	0	0	
2	2	3	0					1					4			3		0			1	
2	3	3	0	0	0	1	2	1	1	1	1	1	7	7	7	9	7	0	0	0	0	0
2	1	3	1	0	0	2	0	1	1	1	1	1	5	6	7	9	6	0	0	0	0	0
2	1	3	1	1	0	2		1	1	1	1		4	6	7	9		0	0	0	0	
2	2	3	0	0	1	0	0	1	1	1	1	1	3	3	6	8	4	1	1	0	0	0
2	2	3	0					1					6					0				

1	1	3	3					2					4					0				
2	1	3	0	1	1	2	2	1	1	1	1	1	4	5	3	7	3	0	0	1	0	0
2	2	3	0	0	1	1		1	1	1	1		4	4	7	8		0	0	0	0	
2	2	3	0	0	0	2		1	1	1	1		4	4	7	7		0	0	0	0	
1	3	1	5	3	4	4		3	2	3	3		2	6	6	4		1	0	0	0	
2	2	3	0	2	0	1	2	1	1	1	1	1	4	4	5	6	1	0	0	0	0	1
2	2	3	1					1					4					0				
2	2	3	0	2		3		1	1		2		4	5				0	0			
2	2	2	5	3		2		3	2		1		3	3		7		1	1		0	
2	2	4	4	1		1		3	1		1		4	7		7		0	0		0	
2	2	3	0	1	1			1	1	1			6	7	3			0	0	1		
2	2	3	0	2	1	2	0	1	1	1	1	1	3	4		6	5	1	0		0	0
1	1	2	5	4	2	1	0	3	3	1	1	1	4	5	4	1	4	0	0	0	1	0
1	2	3	1	2	0	3	3	1	1	1	2	2	3	4	4		5	1	0	0		0
2	1	3	3	2	0	1		2	1	1	1		3	5	7	6		1	0	0	0	
2	2	3	1	2	4	0	0	1	1	3	1	1	6	6	7	4	6	0	0	0	0	0
2	1	3	3	0				2	1				6	3		1		0	1		1	
2	2	3	1	2				1	1				5	4				0	0			
3	2	3	1	0	2	2	1	1	1	1	1	1	3	3	5	7	7	1	1	0	0	0
2	2	2	1					1					6					0				
2	3	3	0	2	1	0	0	1	1	1	1	1	3	3	5	7	6	1	1	0	0	0
2	3	3	0	0	1	1	0	1	1	1	1	1	6		4		4	0		0		0
2	2	3	1	1				1	1				4	2				0	1			
2	2	3	1	1				1	1				5	6				0	0			
1	2	1	4	5	2			3	3	1			3	2	5			1	1	0		
2	2	3	5		5			3		3			3		2			1		1		
2	1	3	1	2				1	1				4	4				0	0			
1	1	2	2	3	0	2		1	2	1	1			3	3	2			1	1	1	
2	3	3	0		0	2	3	1		1	1	2	3		7	9	4	1		0	0	0
1	2	1	2	4	3	7	0	1	3	2	3	1	3	4	4		5	1	0	0		0
2	3	3	2					1					3					1				
2	3	3	3	3				2	2					4					0			
2	3	3	1	0	1	1	0	1	1	1	1	1	5	5	6	7	5	0	0	0	0	0
1	2	3	2	1	2	7		1	1	1	3		3	7	2	4		1	0	1	0	
2	2	2	0					1					3					1				
1	2	3	1	2	0	0	0	1	1	1	1	1	6	6	7	8	6	0	0	0	0	0
2	1	3	3	1	2	3	1	2	1	1	2	1	3	3	5	7	3	1	1	0	0	0
2	2	3	5	4				3	3				4	3				0	1			
2	2	3	0	0	0	1		1	1	1	1		7	7	5			0	0	0		
2	1	3	2					1					3					1				
2	2	3	4			5		3			3		4			4		0			0	

2	2	3	3	1		0		2	1		1		3			1		1			1	
2	2	2	2					1					4					0				
2	2	3	1			1		1			1		4			9		0			0	
2	2	2	2	1	1	4		1	1	1	3		4	5	7	9		0	0	0	0	
2	2	3	0	1	0	2	1	1	1	1	1	1	4	5	7	9	6	0	0	0	0	0
2	2	3	2	1	2	2		1	1	1	1		4	5	4	8		0	0	0	0	
1	2	3	3		4	1		2		3	1		3		7	6		1		0	0	
2	2	3	2	4	0	1		1	3	1	1		4	3	4	4		0	1	0	0	
2	2	3	0	0				1	1				3	4				1	0			
2	2	3	3	1	2	3		2	1	1	2		1	3	2	1		1	1	1	1	
2	2	2	0	1	1			1	1	1			3	3	5			1	1	0		
2	1	3	2	1	2	1	3	1	1	1	1	2	3	3	4	2	3	1	1	0	1	0
2	2	3	0	1				1	1				3	3				1	1			
2	1	3	0	2		1		1	1		1		3	5		7		1	0		0	
2	2	3	1	0	0	1		1	1	1	1		3	4	2	1		1	0	1	1	
1	1	3	4	1	3	1		3	1	2	1		4	3	4			0	1	0		
1	2	2	4	1				3	1				4	3				0	1			
2	1	3	2	1	0	1	0	1	1	1	1	1	3	5	7	7	3	1	0	0	0	0
2	1	3	2	5	0		0	1	3	1		1	4	4	7	7	3	0	0	0	0	0
1	1	3	1					1					4			1		0			1	
2	2	3	2	3	1	0	1	1	2	1	1	1	3	3	7	5	3	1	1	0	0	0
1	2	1	0					1					3					1				
2	3	2	0			0	1	1			1	1	4			8	3	0			0	0
2	2	1	1					1														
2	2	2	0	1		3		1	1		2		3	3		2		1	1		1	
2	2	3	2	0	0	1		1	1	1	1		6	7	6	2		0	0	0	1	
2	3	3	2	3	0			1	2	1			4		6			0		0		
2	2	2	2					1					4					0				
2	2	3	0	4	4	0		1	3	3	1		3	6	2	8		1	0	1	0	
2	3	3	0					1					5					0				
1	2	2	3	1	0	3		2	1	1	2		3	6	5			1	0	0		
1	3	3	5	4		8		3	3		3		3	4				1	0			
2	2	3	1					1					4					0				
2	3	3	0	0				1	1				5	5				0	0			
2	2	3	0	0	0			1	1	1			4	4	4			0	0	0		
2	2	3	0	1	1	3		1	1	1	2		3	3	3	4		1	1	1	0	
2	2	1	1					1														
2	2	3	0	0	0			1	1	1			4	4	7	8		0	0	0	0	
1	1	1	3	0	1			2	1	1			2	2	6			1	1	0		
2	2	3	0	0	0			1	1	1			4	4	6			0	0	0		
2	1	3	3		1	5		2		1	3		4		4	3		0		0	1	

2	2	3	1	1	1	1	2	1	1	1	1	1	7	4	7	8	4	0	0	0	0	0
2	2	2	0	1	0	0	0	1	1	1	1	1	4	6	7	9	5	0	0	0	0	0
2	2	4	1	3	3	5		1	2	2	3		3	3	5	4		1	1	0	0	
1	1	2	3					2					2					1				
2	2	2	3	2	3	5	0	2	1	2	3	1	3	4	4	4	7	1	0	0	0	0
2	3	3	2					1					3					1				
2	3	3	0	2	0	2	0	1	1	1	1	1	3	5	7	8	7	1	0	0	0	0
2	2	3	0	0	0			1	1	1			4					0				
2	2	3	0	4				1	3				4	5				0	0			
2	2	3	2	2	2	3		1	1	1	2		5	5	4	1		0	0	0	1	
2	2	3	1	3	5	2	1	1	2	3	1	1	6	6	4	9	1	0	0	0	0	1
3	2	3	0	1	1	2	3	1	1	1	1	2	3		6	2	5	1		0	1	0
2	2	3	0	0	0			1	1	1			3	4	5	4		1	0	0	0	
3	2	3	0	1				1	1				3	6				1	0			
2	3	3	1	1	0	0		1	1	1	1		6					0				
2	2	3	4	2	8	3		3	1	3	2			4	3	1			0	1	1	
2	2	3	0	1	0	4		1	1	1	3		3	5	3	9	1	1	0	1	0	1
2	3	3	0	0	0	0		1	1	1	1		4	4	5	4		0	0	0	0	
3	2	4	1	0	1			1	1	1			3	4	2			1	0	1		
2	2	3	3	2				2	1				4	2				0	1			
2	2	3	0	0	0	2	2	1	1	1	1	1	6	5	7	7	2	0	0	0	0	1
2	2	3	3	3	2	3	0	2	2	1	2	1	4	5	7	8	3	0	0	0	0	0
2	2	3	0					1					4					0				
2	3	3	1	2	1			1	1	1			4	6	6			0	0	0		
2	1	2	1	1	0	1		1	1	1	1		4	5	7	4		0	0	0	0	
1	3	2	0	1	0	0	0	1	1	1	1	1	4	4	5	8	7	0	0	0	0	0
2	2	3	0	1	0	2		1	1	1	1		3	6	7	3		1	0	0	1	
2	2	3	0	0	0			1	1	1			4	3	4			0	1	0		
2	2	3	1	4	4			1	3	3			3	6	4	2		1	0	0	1	
1	2	3	2					1					3					1				
1	2	3	0	2	4	0	3	1	1	3	1	2	4	4	7	7	7	0	0	0	0	0
2	2	3	4	3				3	2				3	3				1	1			
2	3	3	1	2	0	1		1	1	1	1		5	4	5	7		0	0	0	0	
2	2	3	3		0			2		1			4		3			0		1		
1	1	3	4	3	5	2		3	2	3	1		2	2	3	8		1	1	1	0	
3	1	3	2	1	1	2		1	1	1	1		3	2	2			1	1	1		
2	2	2	4					3					4					0				
2	2	2	1	5	2	2	2	1	3	1	1	1		3	3	3	6		1	1	1	0
2	1	3	4	2		4	0	3	1		3	1	5			9	1	0			0	1
1	3	2	1	2	0	5		1	1	1	3		3	4	7	4		1	0	0	0	
2	3	3	1	0	1	5		1	1	1	3		4	5	3	4		0	0	1	0	

2	2	3	0	1	2	1	0	1	1	1	1	1	5	5	7	8	4	0	0	0	0	0
1	2	1	3	2	1	0	1	2	1	1	1	1	3	7	7	5	7	1	0	0	0	0
2	2	3	4	2				3	1				5	3				0	1			
2	2	3	3					2					3					1				
2	2	3	1	1	2	1	2	1	1	1	1	1	3	4	7	4	6	1	0	0	0	0
2	2	3	2	4	1	2		1	3	1	1		5	3	6	7		0	1	0	0	
2	2		5	1				3	1				2	2				1	1			
2	1	3	1	1	1	3		1	1	1	2		4	7	7	8		0	0	0	0	
2	2	3	3		4			2		3			4		2	3		0		1	1	
2	3	3	2	1	4	2		1	1	3	1		4	5	6	1		0	0	0	1	
1	2	3	2	4				1	3				4	5				0	0			
2	2	3	0					1					5					0				
2	2	3	1	0	1	1	1	1	1	1	1	1	4	3	2	4	7	0	1	1	0	0
2	2	3	8	1	0	2	2	3	1	1	1	1	4	5	7	8	2	0	0	0	0	1
1	1	3	1	1				1	1				5	3				0	1			
2	3	3	1	2	0	3		1	1	1	2		4	5	7	9		0	0	0	0	
2	1	3	0	0	1	0		1	1	1	1		3	4	4	4		1	0	0	0	
2	2	3	0	0	0	0	0	1	1	1	1	1	5	4	6	8	7	0	0	0	0	0
2	2	2	4	2				3	1				1	5				1	0			
2	2	3	0	0	0	1	1	1	1	1	1	1		4	6		4		0	0		0
2	2	3	0	0	0	2		1	1	1	1		4	5	7	7		0	0	0	0	
2	1	3	1	0	1	4		1	1	1	3		4		4	1		0		0	1	
2	2	3	1	0	1	2	2	1	1	1	1	1	5	4	7	8	3	0	0	0	0	0
2	3	3	3	2	3	0	2	2	1	2	1	1	4	4	5	7	3	0	0	0	0	0
1	2	3	3	1	0	1	0	2	1	1	1	1	4	4	4		7	0	0	0		0
3	3	3	1	3	1	3		1	2	1	2		4	7	6	6		0	0	0	0	
2	2	3	2		0	1		1		1	1		4		7	7		0		0	0	
2	2	1	4	1	0			3	1	1			3	2	7			1	1	0		
2	2	3	1	1	0			1	1	1			5	5	7			0	0	0		
1	2	3	0					1					4					0				
2	2	3	0	1	1	1	0	1	1	1	1	1	4	3	4	3	4	0	1	0	1	0
2	3	3	3	4	3	1	1	2	3	2	1	1	4	5	3	1	5	0	0	1	1	0
2	1	3	0	0	1	1	5	1	1	1	1	3	3	3	4	4	3	1	1	0	0	0
2	2	3	0	0	0	1	0	1	1	1	1	1	3	5	7	2	4	1	0	0	1	0
2	2	3	0	1	0	0		1	1	1	1		4	5	7			0	0	0		
1	1	1	1	0	5			1	1	3			4	3	5			0	1	0		
2	2	3	1	3	0	5		1	2	1	3		3	4	6			1	0	0		
2	3	3	1	1	1	0		1	1	1	1		6	7	7	9		0	0	0	0	
2	2	3	0	1	0	2	0	1	1	1	1	1	5	5	3	8	5	0	0	1	0	0
2	2	3	1	2	0	2	4	1	1	1	1	3	3	3	5	4	2	1	1	0	0	1
2	3	3	2	3	1	0	2	1	2	1	1	1	5	6	7	8	1	0	0	0	0	1

2	2	3	1	1	1			1	1	1			3	1	3	1		1	1	1	1	
2	2	3	2	0	2	1		1	1	1	1		4	5	3	5		0	0	1	0	
2	2	3	1	2				1	1				4	3				0	1			
2	2	3	0	0	0	0	0	1	1	1	1	1	4	3	5	8	5	0	1	0	0	0
2	2	3	1	2	0	0	0	1	1	1	1	1	4	6	7	8	4	0	0	0	0	0
2	2	3	4		2			3		1			3		4	7		1		0	0	
2	2	1	3	2	3			2	1	2			2	2	3			1	1	1		
2	3	3	0	4	1	0		1	3	1	1		4	7	7	8		0	0	0	0	
2	2	3	0	0	2	1		1	1	1	1											
1	2	3	1	4	0		0	1	3	1		1	4	5	7	8	3	0	0	0	0	0
1	2	3	1	3	0	2	2	1	2	1	1	1	4	4	7	8	5	0	0	0	0	0
1	2	3	5	5	2	0		3	3	1	1		4	4	4	4		0	0	0	0	
2	1	3	1	0	0	1	1	1	1	1	1	1	5	7	7	6	5	0	0	0	0	0
1	2	1	6	1	4	2	1	3	1	3	1	1		3	3	3	3		1	1	1	0
2	1	3	0	0				1	1				4	3				0	1			
2	2	3	0	0	3	0		1	1	2	1		4	5	7	4		0	0	0	0	
1	2	3	3	1	0	1	1	2	1	1	1	1	3	6	6	8	3	1	0	0	0	0
2	1	3	1	2	3			1	1	2			4	3	7	8		0	1	0	0	
2	2	3	1	0	0	2	0	1	1	1	1	1	4	4	5	6	4	0	0	0	0	0
2	2	2	1					1					5					0				
1	2	2	0	3	2			1	2	1			2	3	3			1	1	1		
2	2	2	3	2	0			2	1	1			2	3	4			1	1	0		
2	2	2	1	1	0	0	0	1	1	1	1	1	4	3	6	8	5	0	1	0	0	0
2	2	2	2	2	3	2		1	1	2	1		4	4	4	8		0	0	0	0	
2	1	3	2	4	0	2		1	3	1	1		6	6	7	8		0	0	0	0	
2	2	3	0	1	1	1		1	1	1	1		4	3	3	2		0	1	1	1	
2	2	3	1	0				1	1													
1	2	1	6		1	3	2	3		1	2	1	4		5	6	3	0		0	0	0
3	1	3	2	1	2			1	1	1			3	4	1			1	0	1		
2	1	3	0	1				1	1				3	3				1	1			
3	2	3	0					1					3					1				
1	3	2	1					1					3					1				
2	2		0	0	0			1	1	1			4	4	7			0	0	0		
2	2	3	0	0		1		1	1		1			3		3			1		1	
2	2	3	4					3					3					1				
2	2	2	1	0	0	3	0	1	1	1	2	1	2	4	6	8	5	1	0	0	0	0
2	3	3	0	2	1	3	1	1	1	1	2	1	4	4	2	8	4	0	0	1	0	0
2	3	3	2		0			1		1			4		7			0		0		
2	2	3	2	2	1	1		1	1	1	1		4	7	7	9		0	0	0	0	
2	2	3	0	1	4	2	0	1	1	3	1	1	5	6	3	8	2	0	0	1	0	1
2	2	3	1	0	0		0	1	1	1		1	4	5	7	9	3	0	0	0	0	0

2	3	3	0	1	2	0		1	1	1	1		4	3	6	7		0	1	0	0	
2	2	3	2	3	2			1	2	1			4		5			0		0		
2	2	3	1	1	2	0		1	1	1	1		4	5	7			0	0	0		
1	2	3	3					2					3					1				
2	3	3	2	2	1			1	1	1			5	7	7	9		0	0	0	0	
2	3	3	0	0	0	0	0	1	1	1	1	1	4	7	7	8	4	0	0	0	0	0
2	3	3	0	4	3			1	3	2			3	5	6			1	0	0		
2	1	3	1	2		1		1	1		1		3	4		1		1	0		1	
2	2	2	0					1					3					1				
2	3	3	2					1					5					0				
3	3	3	0	2		0		1	1		1		7	7				0	0			
2	2	3	0	0	1	0		1	1	1	1		3	5	7			1	0	0		
2	2	3	0		0			1		1			5		3			0		1		
2	3	2	2	1	3		7	1	1	2		3	4	4	4	5	5	0	0	0	0	0
2	2	3	2	1	2	4		1	1	1	3		4	4		8		0	0		0	
1		2	0					1					3					1				
2	2	3	1	2	0	6	2	1	1	1	3	1	4	5	7	8	4	0	0	0	0	0
2	3	3	1	0	0	1		1	1	1	1		4	4	7	8		0	0	0	0	
2	1	3	1	0	0	4		1	1	1	3		4	7	7	3		0	0	0	1	
2	3	3	0	1	1	0	0	1	1	1	1	1	4	6	7	8	6	0	0	0	0	0
1	2	3	1	2	3	2	0	1	1	2	1	1	5	5	3	3	1	0	0	1	1	1
2	2	3	0	0	2	1	0	1	1	1	1	1	4	5	3		4	0	0	1		0
2	3	1	4	3	0			3	2	1			1	1	7			1	1	0		
2	3	3	0	1	2	1	2	1	1	1	1	1	4	6	5	7	2	0	0	0	0	1
2	2	3	2	3	1	2	1	1	2	1	1	1	4	7	7	8	3	0	0	0	0	0
2	2	3	0	2		5		1	1		3		4	7		9		0	0		0	
2	2	3	0	1	1	3		1	1	1	2		5	5	7	4		0	0	0	0	
2	1	3	1	0	0	4		1	1	1	3		3	2	2	1		1	1	1	1	
2	3	3	4					3					5					0				
2	3	3	3	0	0	0	1	2	1	1	1	1	5	4	5	7	6	0	0	0	0	0
2	2	3	2	0	2	2	1	1	1	1	1	1	4	5	5	1	3	0	0	0	1	0
2	3	3	1	2	0	1	0	1	1	1	1	1	2	3	4	3	3	1	1	0	1	0
2	2	4	1	1	2			1	1	1			3	4	2	9		1	0	1	0	
2	3	3	0	1	2	2	1	1	1	1	1	1	6	4	5	7	3	0	0	0	0	0
2	1	2	2	1	0	5	0	1	1	1	3	1	3	2	2	2	4	1	1	1	1	0
2	2	3	2	4	0	2	0	1	3	1	1	1	4	2	5	7	3	0	1	0	0	0
2	2	3	1	2	1	3	1	1	1	1	2	1	6	6	7	8	7	0	0	0	0	0
1	1	2	0	0	1	3	2	1	1	1	2	1	4	4	3	7	1	0	0	1	0	1
2	2	2	2					1					3					1				
2	3	3	4	1	0			3	1	1				7	7				0	0		
2	2	3	0	0	1	2		1	1	1	1		4	4	1	4		0	0	1	0	

2	1	3	0					1														
1	2	2	1	2				1	1					2					1			
2	2	3	1	1	3	4	5	1	1	2	3	3	4		2	4	1	0		1	0	1
2	2	3	2	0	0	0	0	1	1	1	1	1	4	5	5	7	5	0	0	0	0	0
2	2	3	0	0	1	2		1	1	1	1		3	3	7	4		1	1	0	0	
2	2	3	6	2	1	3		3	1	1	2		4	7	7			0	0	0		
2	2	3	0					1					7					0				
2	2	3	2	1	4	1		1	1	3	1		4	3	7	6		0	1	0	0	
2	1	2	3					2					2					1				
2	3	3	2	1				1	1				3	3				1	1			
2	3	3	0	1	0	1	1	1	1	1	1	1	4	5	5	5	2	0	0	0	0	1
2	2	3	2	2	2			1	1	1			4	4	1			0	0	1		
2	3	3	3	3	1	0	0	2	2	1	1	1	3	7	7	9	5	1	0	0	0	0
2	2	3	2	1	1	1	0	1	1	1	1	1	4	4	7	8	4	0	0	0	0	0
2	2	3	2	2	3	2	1	1	1	2	1	1	3	5	6	8	7	1	0	0	0	0
2	2	3	0		1	0		1		1	1		5		6	7		0		0	0	
2	3	3	1	2	2	4	2	1	1	1	3	1	2	2	7	5	1	1	1	0	0	1
2	2	3	0	3	2	0		1	2	1	1		4	2	1			0	1	1		
2	2	3	1	0	1	0	1	1	1	1	1	1	4			4	6	0			0	0
1	2	3	1	2	1	9	2	1	1	1	3	1	3	5	6	4	4	1	0	0	0	0
2	2	2	3					2					5					0				
2	2	3	1	1	0	0		1	1	1	1		4	4	5	5		0	0	0	0	
2	2	3	1	1	0	4		1	1	1	3		3	4	7	7		1	0	0	0	
2	2	3	1	3	3	0	0	1	2	2	1	1	4	5	2	4	6	0	0	1	0	0
1	2	3	2					1					3					1				
2	3	3	1	4	1	5	4	1	3	1	3	3	2	2	3	1	1	1	1	1	1	1
3	2	3	0	0	0	2		1	1	1	1		4	3	3	2		0	1	1	1	
2	2	3	3	2	2	5		2	1	1	3		5	6	7	7		0	0	0	0	
2	3	3	0	0	0	2	0	1	1	1	1	1	7	3	7	9	5	0	1	0	0	0
2	3	3	1	3	1	1		1	2	1	1		4	4	6	8		0	0	0	0	
2	2	3	1	1	5			1	1	3			4	3	2			0	1	1		
2	2	3	1	2	1	0	2	1	1	1	1	1	4	5	3	7	5	0	0	1	0	0
2	1	1	3	3				2	2				1	3				1	1			
2	1	1	0	0	0	1		1	1	1	1		2	5	5	7		1	0	0	0	
2	3	3	1	3	0	1	1	1	2	1	1	1	6	4	7	9	5	0	0	0	0	0
2	2	3	0					1					3					1				
2	1	3	0		1	2	0	1		1	1	1	4		7	3	6	0		0	1	0
2	3	3	1		2	3		1		1	2		3		7	2		1		0	1	
2	2	3	2	1	1			1	1	1			3	5	3	3		1	0	1	1	
1	2	3	2	3	1	2	0	1	2	1	1	1	3	3	5	7	3	1	1	0	0	0
2	3	3	0	2	0	0	6	1	1	1	1	3	5	6	7	9	1	0	0	0	0	1

2	1	3	0	0	3	2	0	1	1	2	1	1	4	5	3	2	6	0	0	1	1	0
2	2	3	0		0			1		1			4		7			0		0		
2	2	3	0	0	0	2	0	1	1	1	1	1	4	5	7	9	7	0	0	0	0	0
2	2	3	1		1	3		1		1	2		4		2	2		0		1	1	
1	3	3	0	2	2			1	1	1			2	4	7			1	0	0		
2	2	3	2	1	2			1	1	1			7	3	5			0	1	0		
2	2	1	0	2	1	2	1	1	1	1	1	1	6	2	5	5	4	0	1	0	0	0
1	2	3	2	1	1	0	2	1	1	1	1	1	3	4	7	7	7	1	0	0	0	0
2	2	3	0	2	1	1		1	1	1	1		3	7	7	7		1	0	0	0	
2	3	3	3	0	1	1		2	1	1	1		4	4	5	6		0	0	0	0	
2	2	3	1	1	1	0	1	1	1	1	1	1	5	4	6	6	5	0	0	0	0	0
2	3	3	1	2	0	2	0	1	1	1	1	1	5	5	7	9	7	0	0	0	0	0
2	3	3	1	1	0	4	0	1	1	1	3	1	5	3	7	8	4	0	1	0	0	0
2	2	3	2	2	0	1	1	1	1	1	1	1	5	6	7	7	1	0	0	0	0	1
2	2	3	0	0	0	0	1	1	1	1	1	1	4	7	7	9	1	0	0	0	0	1
2	1	1	2					1					3					1				
2	1	3	0					1					3					1				
2	2	3	0	0	0	1	0	1	1	1	1	1	4	5	7	8	4	0	0	0	0	0
2	1		0	1	1	2		1	1	1	1		4	4	4	1		0	0	0	1	
2	2	3	0	1		1	2	1	1		1	1	4	3		1	3	0	1		1	0
2	1	3	3	1				2	1				4	4				0	0			
1	1	1	4	1	0			3	1	1			2	5	7			1	0	0		
2	3	3	0	0	0	1	3	1	1	1	1	2	4	4	6	7	3	0	0	0	0	0
2	3	3	0	0	0	1	0	1	1	1	1	1	5	6	7	5	3	0	0	0	0	0
2	2	3	3	3	0	2		2	2	1	1		4	6	7	1		0	0	0	1	
2	1	3	0	1	1	1		1	1	1	1		3	4	3	1		1	0	1	1	
2	2	3	0	1	1	1	1	1	1	1	1	1	4	5	7	9	6	0	0	0	0	0
1	2	2	4					3														
2		3		0	2	1	2		1	1	1	1		4	6	8	2		0	0	0	1
2	2	3	0		0	1	0	1		1	1	1	7		3	7	4	0		1	0	0
2	1	2	0					1					4					0				
2	2	4	4	0				3	1				3	3				1	1			
2	2	2	3	2	1	0	0	2	1	1	1	1	5	4	2	4	7	0	0	1	0	0
2	2	3	1	3	1	0		1	2	1	1		3	4	5	5		1	0	0	0	
2	1	3	0	1	0	1		1	1	1	1		3	3	3	3	4	1	1	1	1	0
2	2	3	4	2	2			3	1	1			4	3	3			0	1	1		
2	2	1	0	2	1	0	0	1	1	1	1	1	7	5	7	9	2	0	0	0	0	1
2	2	3	0	1	0	0	0	1	1	1	1	1	4	4	7	9	6	0	0	0	0	0
2	3	3	0	1	2			1	1	1			4	4	7			0	0	0		
1	2	2	2	0	0			1	1	1			2	5	7			1	0	0		
2	2	3	0	2	2			1	1	1			3	3	6	8		1	1	0	0	

2	2	3	0	0	6			1	1	3			5	4	2	1	1	0	0	1	1	1
1	2	1	1	2	3	4		1	1	2	3		4	3	3	3		0	1	1	1	
2	2	3	1	3	1	1	0	1	2	1	1	1	3	3	2	1	3	1	1	1	1	0
2	1	3	0	0	1	2	2	1	1	1	1	1	5	7	3	7	1	0	0	1	0	1
2	3	3	0	2	1	1	1	1	1	1	1	1	4	5	6	5	7	0	0	0	0	0
2	2	3	0	0	0			1	1	1			4	6	5			0	0	0		
1	2	3	0	2	1	0	0	1	1	1	1	1	4	7	3	8	7	0	0	1	0	0
2	2	3	0		1	0		1		1	1		4		5			0		0		
2	2	3		0	0	1	0		1	1	1	1	3	6	7	6	4	1	0	0	0	0
2	2	1	3	3	4	2		2	2	3	1		3	3	3	1		1	1	1	1	
2	2	3	0	1	0	2	5	1	1	1	1	3	4	4	6	7		0	0	0	0	
2	2	3	3					2					4					0				
2	2		5					3					3					1				
2	2	2	0	2	3			1	1	2			4	4	2	4		0	0	1	0	
2	2	3	1	3	2	4		1	2	1	3		3	3	5	2		1	1	0	1	
1	2	3	1					1					4					0				
3	2	3	0					1					4					0				
2	1	2	2					1														
2	1	3	3	4	1	3	1	2	3	1	2	1	4	4	7	5	2	0	0	0	0	1
1	1	2	3					2					3					1				
2	1	3	0	1	2	1	1	1	1	1	1	1	4	5	6	1	4	0	0	0	1	0
2	2	3	0	0	0			1	1	1			4	5	7			0	0	0		
2	1	3	0	0				1	1				3	2				1	1			
1	2	3	1	1		1		1	1		1		3					1				
2	1	3	3	5	4			2	3	3			4	2	6			0	1	0		
2	2	2	1		2	1	1	1		1	1	1	4		7	6	7	0		0	0	0
2	2	3	0	1	0	3		1	1	1	2		5	6	6	8		0	0	0	0	
2	3	3	0	0		1		1	1		1		5	5		8	5	0	0		0	0
2	2	3	0	1	0	1	0	1	1	1	1	1	4	5	6	7	7	0	0	0	0	0
1		1	3		2			2		1			3		4			1		0		
2	2	3	2	1	2	1	2	1	1	1	1	1	4	5	7		3	0	0	0		0
1	1	1	2					1					3					1				
2	2	3	3	1	5	1	2	2	1	3	1	1	3	4	3	2	3	1	0	1	1	0
3	1	3	1	2	0	3		1	1	1	2		2	2	4	1		1	1	0	1	
2	1	3	1	0	1	4		1	1	1	3		3	3	1	1		1	1	1	1	
1	2	3	0	2	0	5	3	1	1	1	3	2	4	3	7	9	1	0	1	0	0	1
1	2	3	1	0	5			1	1	3			4	4	6	4		0	0	0	0	
1	3	2	1	1				1	1				3	4				1	0			
2	2	3	5	5	5	2	0	3	3	3	1	1		5	6	8	5		0	0	0	0
2	2	4	2	1		3	4	1	1		2	3	5	2		7	2	0	1		0	1
2	1	2	1	4	5	4	7	1	3	3	3	3	3	4	4	2		1	0	0	1	

1	1	1	1	0	1			1	1	1			1	3	6			1	1	0		
2	3	3	1	0	1			1	1	1			3	4	3	1		1	0	1	1	
2	1	3	2					1					3					1				
2	1	3	0		3	4		1		2	3		4		2	1	3	0		1	1	0
2	2	3	1	2	0	0	0	1	1	1	1	1	4	5	7	9	7	0	0	0	0	0
2	3	3	0			5	4	1			3	3	3			4	1	1			0	1
2	2	3	0		1	1	0	1		1	1	1	4		7	8	4	0		0	0	0
2	2	3	2	1	1	2		1	1	1	1		4	7	1	2		0	0	1	1	
2	3	3	1	6	1	2	1	1	3	1	1	1	3	2	1	1	1	1	1	1	1	1
2	2	3	3	1	3	3	1	2	1	2	2	1	3	3	4	2	6	1	1	0	1	0
2	2	2	3					2														
2	2	3	1	0	0	2	2	1	1	1	1	1	3	3	7	9	7	1	1	0	0	0
2	2	3	3	3	3			2	2	2			3	4	4			1	0	0		
2	2	3	5	3	2	2	5	3	2	1	1	3	3	3	5	2	1	1	1	0	1	1
2	2	3	0	1	1	1	0	1	1	1	1	1	5	7	7		5	0	0	0		0
1	2	2	0					1					1					1				
2	2	3	1	2	0	1		1	1	1	1		3	3	5			1	1	0		
1	2	3	1	0		3	0	1	1		2	1	4	4		9	5	0	0		0	0
2	2	3	3					2					3					1				
2	2	3	0	2	0	1	3	1	1	1	1	2	4	5	7	8	4	0	0	0	0	0
1	2	2	2	2	2	3		1	1	1	2		3	5	7	9		1	0	0	0	
2	3	2	1					1					4					0				
2	2	3	3	3	1	3		2	2	1	2		4	7		8		0	0		0	
1	2	3	1	1	1	0	0	1	1	1	1	1	3	4	6	9	5	1	0	0	0	0
1	2	3	4	0	0	2		3	1	1	1		2	5	6			1	0	0		
2	2	3	0	3	0	1	0	1	2	1	1	1	4	4	7	9	4	0	0	0	0	0
2	1	3	0		1	2		1		1	1		3		5	6		1		0	0	
2	2	3	2	3	6	0		1	2	3	1		3	2	3	1		1	1	1	1	
2	2	2	0					1					3					1				
1	1	1	2		0	2	0	1		1	1	1	1		2	1	3	1		1	1	0
2	2	3	2					1					3					1				
3	2	3	3	4	3	4		2	3	2	3		5	6	6	1		0	0	0	1	
2	2	3	2	2	1	4	2	1	1	1	3	1	4	4		6	4	0	0		0	0
3	2	3	0	1	2	3		1	1	1	2		5	6	7			0	0	0		
2	2	3	0	1	0	2		1	1	1	1		4	4	4	5		0	0	0	0	
3	2	3	2	1	2	0		1	1	1	1		4	6	6	3		0	0	0	1	
2	2		3					2					3					1				
2	3	3	1	0	0	0	1	1	1	1	1	1	5	7	7	9	7	0	0	0	0	0
2	1	3	0		3	3		1		2	2		3		3	3		1		1	1	
2	2	3	0	0	0			1	1	1			6	6	7	9		0	0	0	0	
2	2	3	0	1	1			1	1	1			5	5	3			0	0	1		

2	2	3	0	0	0	0	0	1	1	1	1	1	3	4	5	3	4	1	0	0	1	0
2	2	2	1	2	2	3		1	1	1	2		4	4	7	9		0	0	0	0	
2	2	3	0	1	2			1	1	1			4	5	7			0	0	0		
2	2	3	1	2	2		1	1	1	1		1	3	5	6		4	1	0	0		0
2	2	3	1	0	0			1	1	1			5	4	5			0	0	0		
2	2	1	2					1					3					1				
2	2	3	0	0	0	3		1	1	1	2		4	6	5	7		0	0	0	0	
2	2	3	1	2	0	1		1	1	1	1		4	4	6	6		0	0	0	0	
2	3	3	0	1	1	0	1	1	1	1	1	1	4	4	7	9	7	0	0	0	0	0
2	2	2	2					1					3					1				
2	3	3	0	2	5	1	2	1	1	3	1	1	4	7	7	8	1	0	0	0	0	1
2	1	3	2	2	4	4		1	1	3	3		4	5	7	1		0	0	0	1	
2	3	3	0	1	0	0	0	1	1	1	1	1	3	4	7	2	7	1	0	0	1	0
1	3	3	2		2	3		1		1	2		4		7	9		0		0	0	
2	2	3	0	1	1	0		1	1	1	1		4	5	5	8		0	0	0	0	
2	2	3	1					1					4					0				
2	3	3	1	3	0	2	1	1	2	1	1	1	5	4	5	1		0	0	0	1	
2	2	3	0	0	0	3	1	1	1	1	2	1	3	3	4	4	4	1	1	0	0	0
2	3	3	0	2	0	2	0	1	1	1	1	1	4	4	7	7	3	0	0	0	0	0
2	2	2	1		0			1		1			5		5			0		0		
2	3	3	0	1	0			1	1	1			4			9		0			0	
2	2	3	2	1	4	4		1	1	3	3		5		2	1		0		1	1	
2	1	1	0	0	1	3		1	1	1	2		3	1	7	7		1	1	0	0	
2	2	3	0	1	1			1	1	1				4	7				0	0		
2	1	3	2	1	1	2	2	1	1	1	1	1	4	2	2	2	1	0	1	1	1	1
2	2	3	4	5	1			3	3	1			4	5	2			0	0	1		
2	3	3	0	0	2	5	0	1	1	1	3	1	4	4	4	8	4	0	0	0	0	0
2	1	3	1		1			1		1			3					1				
2	3	3	1			0		1			1		4					0				
1	3	1	2	2	1	6		1	1	1	3		6	2	6	4		0	1	0	0	
2	1	3	4	1	3	1		3	1	2	1		3	4	3	3		1	0	1	1	
2	1	3	0	0	0	1		1	1	1	1		4	7	3			0	0	1		
2	2	3	0	0	2	1	2	1	1	1	1	1	3	5	3	1	3	1	0	1	1	0
3	1	3	1	2	0	0	2	1	1	1	1	1	4	5	4	2	4	0	0	0	1	0
2	1	3	2	1	2	2	0	1	1	1	1	1	3	4	6	5	5	1	0	0	0	0
2	2	2	0		0	0		1		1	1				6					0		
1	1	2	0	1	0	2		1	1	1	1		3	2	7	5		1	1	0	0	
2	2	3	1	2	3	5		1	1	2	3		3	4	4	5		1	0	0	0	
2	2	3	1	3	0			1	2	1			4	5	3			0	0	1		
2	3	3	1	2	1	0		1	1	1	1		3	3	5	3		1	1	0	1	
2	2	3	3	2	5	0	2	2	1	3	1	1	4	2	2	1	4	0	1	1	1	0

3	2	3	4		1	2	2	3		1	1	1	2		4	3	3	1		0	1	0
3	2	3	1	2	3			1	1	2			6	4	3			0	0	1		
2	3	2	2	2	4	1	2	1	1	3	1	1	5	4	4		3	0	0	0		0
2	1	3	1	1	1	2		1	1	1	1		4	4	5			0	0	0		
2	2	3	0	3	2	1		1	2	1	1		4	4	6	6		0	0	0	0	
2	2	3	1		0	0		1		1	1		4		7	8	6	0		0	0	0
2	2	3	1	1	1	2	0	1	1	1	1	1	4	7	7	8	4	0	0	0	0	0
2	3	3	0	4	0	2		1	3	1	1		4	5	6	7		0	0	0	0	
2	2	3	0	2	1			1	1	1			4	6	7			0	0	0		
1	1	2	1	1	2	1		1	1	1	1		2	3	2	1		1	1	1	1	
1	1	3	3					2					3					1				
3	2	3	0	2	0	1	0	1	1	1	1	1	2	3	4	8	7	1	1	0	0	0
2	2	3	1					1					3					1				
1	3	3	0	1	1	3	1	1	1	1	2	1	4	4	4			0	0	0		
2	1	3	1	1	1	1		1	1	1	1		3	6	7	8	5	1	0	0	0	0
2	1	3	1	1	0	0		1	1	1	1		3	5	7	8		1	0	0	0	
2	2	2	2					1					4					0				
2	1	3	2	1	1	5		1	1	1	3		3	3	3	6		1	1	1	0	
1	2	2	2		2			1		1			2		4			1		0		
1	2	1	1		2	4		1		1	3		4		3			0		1		
2	3	2	0	3	0	0	1	1	2	1	1	1	6	3	7	9		0	1	0	0	
2	3	3	2	3	0	1	2	1	2	1	1	1	4	6	7	9	7	0	0	0	0	0
2	1	3	1	1	1	1		1	1	1	1		4	5	5	5		0	0	0	0	
2	3	3	2					1					4					0				
2	2	3	0	2	1			1	1	1			3	5	2	3		1	0	1	1	
2	2	3	0	0	3	1	1	1	1	2	1	1	4	4	3	2	1	0	0	1	1	1
1	2	3	1					1					3					1				
2	2	3	1		0	0		1		1	1		3		5	1		1		0	1	
2	3	3	5	1	0	2		3	1	1	1		3	5	7	3		1	0	0	1	
2	3	3	0					1					5					0				
1	2	3	2	3	3	2		1	2	2	1			3	7	9			1	0	0	
1	3	3	5					3					4					0				
3	3	2	1	1				1	1					5					0			
2	2	2	2	2	5		0	1	1	3		1	4	6	4	7	5	0	0	0	0	0
2	2	3	3					2					2					1				
2	2	3	1	1	4	0	0	1	1	3	1	1	3	4	3	1	4	1	0	1	1	0
3	2	3	0	1	1	1	0	1	1	1	1	1	5	4	7	6	3	0	0	0	0	0
3	1	3	0	2	0	0	1	1	1	1	1	1	3	4	4	2	3	1	0	0	1	0
2	1	3	4			2	1	3			1	1	3			3	4	1			1	0
2	3	3	0	1	0	2	1	1	1	1	1	1	4	7	7	9	7	0	0	0	0	0
2	2	3	2	1	1	3	1	1	1	1	2	1	6	5	5	7	6	0	0	0	0	0

1	2	3	2					1					3					1				
2	2	2	0					1					4					0				
2	1	3	5	3	2	5		3	2	1	3		2	6	3	8		1	0	1	0	
2	3	3	0	1	1	1	1	1	1	1	1	1	6	2	2	3	6	0	1	1	1	0
1	1	2	1					1					3					1				
3	2	3	0	0	0	1	0	1	1	1	1	1	3	3	3	1	3	1	1	1	1	0
2	2	3	1	1	0	0		1	1	1	1		3	4	5	4		1	0	0	0	
2	2	3	0					1					4					0				
2	2	3	0	1	1	0	1	1	1	1	1	1	5	5	7	9	6	0	0	0	0	0
1	2	1	2	1	0	4		1	1	1	3		3	3	5	7		1	1	0	0	
2	2	3	4	1				3	1				3	5				1	0			
1	2	3	1	0	0	2	0	1	1	1	1	1	3	6	7	3	7	1	0	0	1	0
2	1	3	0	1	0	0	1	1	1	1	1	1		4	7		6		0	0		0
2	1	1	5		3	8		3		2	3				2	1				1	1	
2	2	3	0	3	1	1	1	1	2	1	1	1	4	5	5	4	7	0	0	0	0	0
2	2	3	2	1	0	1		1	1	1	1		4	4	6	8		0	0	0	0	
2	3	3	1	3	1	0	0	1	2	1	1	1	4	5	7	8	5	0	0	0	0	0
2	2	2	0		4	4		1		3	3		3		5	4		1		0	0	
2	2	3	3		0	1	1	2		1	1	1	3		6	7	4	1		0	0	0
2	2	4	4	2	2	4		3	1	1	3		2	2	2	3		1	1	1	1	
2	3	3	0	0	0	3	1	1	1	1	2	1	4	5	6	8	5	0	0	0	0	0
1	1	3	1	2	2	6	4	1	1	1	3	3	4	5	6	8	1	0	0	0	0	1
2	2	3	1	1	0	1		1	1	1	1		4	6	7	8		0	0	0	0	
2	2	3	3	3	0	4		2	2	1	3		4	4	6	8		0	0	0	0	
2	3	3	3	1	2	2		2	1	1	1			4	5	6			0	0	0	
2	1	4	2	1	0	1		1	1	1	1		2	2	1	2		1	1	1	1	
2	2	3	0		1			1		1			4		5			0		0		
2	2	3	0	1	0	3		1	1	1	2		3	3	6	8		1	1	0	0	
2	2	3	0	3	0	3	0	1	2	1	2	1	4	1	2	3		0	1	1	1	
2	2	3	1		0	3	0	1		1	2	1	4		7	3	3	0		0	1	0
3	1	3	3	3	5	0		2	2	3	1		4	1	3	1		0	1	1	1	
2	3	3	1	0	1	2		1	1	1	1		3	6	7			1	0	0		
2	1	3	0	1				1	1				3	4				1	0			
2	2	3	0					1					7					0				
2	2	3	1	1	1		1	1	1	1		1	4	3	3		4	0	1	1		0
2	2	3	1	1	3	3	0	1	1	2	2	1	3	4	3	1	6	1	0	1	1	0
1	2	2	4	3	3			3	2	2			5	2	4			0	1	0		
1	2	3	4		3		2	3		2		1	3		4	5		1		0	0	
2	2	3	0	1	1	2		1	1	1	1		4	6	7			0	0	0		
1	2	2	1					1					3					1				
2	1	2	0	0	0			1	1	1			3	3	3			1	1	1		

2	2	3	1	2	0	4		1	1	1	3		4	5	6	6		0	0	0	0	
2	1	3	0		2	0		1		1	1		5			1		0			1	
2	2	3	0		1	2	1	1		1	1	1	4		5		1	0		0		1
2	3	3	0	1	3	2		1	1	2	1		4	3	3	1		0	1	1	1	
1	2	4	3	0		3		2	1		2		4	4				0	0			
2	2	3	0	1	0	0	0	1	1	1	1	1	3	7	7	9	4	1	0	0	0	0
2	3	3	0		3	0		1		2	1		3		7	7		1		0	0	
2	3	3	3					2					4					0				
2	2	3	5					3														
2	2	3	1					1					3					1				
2	2	3	1	3	3	3	7	1	2	2	2	3	4	2	2	1	3	0	1	1	1	0
2	2	3	0	0	0	1	1	1	1	1	1	1		4	5		6		0	0		0
2	3	3	2	3	2	2	2	1	2	1	1	1	4	7	6	3	3	0	0	0	1	0
2	3	3	1	5	3	2	4	1	3	2	1	3	5	4	5	3	3	0	0	0	1	0
2	2	3	2	1	0	1	1	1	1	1	1	1	4	6	7		7	0	0	0		0
2	2	3	3	2	0			2	1	1			3	3	5			1	1	0		
2	2	3	3	2		3		2	1		2		3	3		6		1	1		0	
2	2	3	1					1					4					0				
2	2	3	2	1	0	2	0	1	1	1	1	1	4	4	6	8	1	0	0	0	0	1
2	2	3	1	2	2	2	1	1	1	1	1	1	3	3	4		5	1	1	0		0
2	2	3	1	2	3	0		1	1	2	1		4	4	4	8		0	0	0	0	
2	2	3	1	1	1			1	1	1			4	4	5			0	0	0		
2	3	3	4	3		2	2	3	2		1	1	5	4		7	3	0	0		0	0
2	1	3	0	3				1	2													
2	2	3	0	3				1	2				5	7				0	0			
2	3	3	0	0	1	0	1	1	1	1	1	1	6	4	6	1	3	0	0	0	1	0
2	2	3	2	1	1	4		1	1	1	3		5	6	7	8		0	0	0	0	
2	2	3	0	0	0			1	1	1			7	5	7			0	0	0		
2	2	3	1	0	1	0		1	1	1	1		6	5	6	8		0	0	0	0	
2	3	3	0	0	0	2	0	1	1	1	1	1	5	6	7	9	6	0	0	0	0	0
1	1	4	0	1	1			1	1	1			5	6				0	0			
2	2	3	0	0	0			1	1	1			4	3	7	2		0	1	0	1	
2	1	3	1	3	1	3	1	1	2	1	2	1	4	3	3	1	6	0	1	1	1	0
2	2	2	1	1	1	0	1	1	1	1	1	1	5	3	7	8	7	0	1	0	0	0
1	1	1	3	3	5	4		2	2	3	3		2	2	2	3		1	1	1	1	
2	3	1	4		3			3		2			3		5	9		1		0	0	
2	2	2	2	4	3			1	3	2			4	2	5	1		0	1	0	1	
2	2	3	0	0	0	1		1	1	1	1		5	3	6			0	1	0		
2	2	3	0		0		0	1		1		1	3		7	8	7	1		0	0	0
3	1	3	0	0	0	1	1	1	1	1	1	1	4	5	4	1	1	0	0	0	1	1
2	2	3	1	2	1	2	3	1	1	1	1	2	3	3	6	8	5	1	1	0	0	0

2	2	3	0	2	0			1	1	1			5	4	7			0	0	0		
2	2	3	0	1	1	1		1	1	1	1		4			1		0			1	
1	2	1	2	1	1	1		1	1	1	1		2	5	7	1		1	0	0	1	
2	2	3	0	1	2	1		1	1	1	1		5	4	3	7		0	0	1	0	
2	2	3	2	6	3	5	1	1	3	2	3	1	4	3	4	8	3	0	1	0	0	0
2	2	3	3	5		0		2	3		1		3	4		1		1	0		1	
2	3	3	1	2	0	3	4	1	1	1	2	3	3	6	7	9	7	1	0	0	0	0
1	2	2	2	7		5		1	3		3		3	2		3		1	1		1	
2	1	3	1	2	3	1		1	1	2	1		4	3	2			0	1	1		
1	1	2	1	1	1			1	1	1			3	2	2	3		1	1	1	1	
2	2	3	1					1					4					0				
1	2	3	4					3					2					1				
2	1	3	3	0	1			2	1	1			3		3			1		1		
2	2	1	4	3	3	3	2	3	2	2	2	1	1	2	3	4	5	1	1	1	0	0
1	2	2	4	0	0	4	2	3	1	1	3	1	5	4	7		4	0	0	0		0
2	2	3	3	1	1	0	1	2	1	1	1	1	4	2	5	8	4	0	1	0	0	0
2	2	3	0	1	0	5	3	1	1	1	3	2	6	6	7	8	1	0	0	0	0	1
3	2	3	0		3		1	1		2		1					1					1
2	3	3	1	1	3	1	3	1	1	2	1	2	3	4	3	3	3	1	0	1	1	0
2	2	4	2					1					2					1				
1	2	3	0	1	0	0		1	1	1	1		3		5	8	3	1		0	0	0
1	3	3	0	1	0	3		1	1	1	2		4	6	5	8	1	0	0	0	0	1
2	2	2	0					1					3					1				
1	2	3	0		0	4	0	1		1	3	1	4		7	9	3	0		0	0	0
2	2	3	0	0	0	0	2	1	1	1	1	1	3	4	4	3	1	1	0	0	1	1
2	1	2	3					2					2					1				
1	1	3	2	2	5	6	0	1	1	3	3	1	3	5	7	8		1	0	0	0	
2	2	3	2	2	1	2		1	1	1	1		4	4	5	2		0	0	0	1	
2	2	2	4	1	2		1	3	1	1		1	3	3	2	6	6	1	1	1	0	0
1	1	2	1					1					3			5		1			0	
2	3	3	0	3	0	2	2	1	2	1	1	1	4	5	6	6		0	0	0	0	
2	2	3	0	4	3			1	3	2			4	6	6	8		0	0	0	0	
2	2	3	1	2	3	4		1	1	2	3		4	4	7	8		0	0	0	0	
2	2	3	0	0	0	0		1	1	1	1		4	5	6	8		0	0	0	0	
2	1	3	1	1	3	2		1	1	2	1		3		4	1		1		0	1	
1	2		5					3					2					1				
2	2	3	1	0				1	1				5	5				0	0			
2	2	3	0	0	1	0	4	1	1	1	1	3	3	5	3	3	5	1	0	1	1	0
2	2	3	0	0	1		0	1	1	1		1	5	6	7	1	4	0	0	0	1	0
2	2	3	2	1	0			1	1	1			4	6	7			0	0	0		
2	2	3	1		4	2		1		3	1		4		6	9		0		0	0	

2	2	3	1	1	1	3		1	1	1	2		4	4	5	7		0	0	0	0	
1	2	1	2	1				1	1				3	5				1	0			
2	2	3	0	0	0	1	4	1	1	1	1	3	4	5	7	7	4	0	0	0	0	0
2	2	3	1	3	2			1	2	1			4	4	3	5		0	0	1	0	
2	3	3	1	3	0	1		1	2	1	1		4	7	7	9		0	0	0	0	
1	1	1	1	1	1			1	1	1			4	4	3	2		0	0	1	1	
2	2	3	0	1	2	1		1	1	1	1		3	3	3	2		1	1	1	1	
2	2	3	0	1	3	0	1	1	1	2	1	1	4	5	6	6	4	0	0	0	0	0
1	1	3	4	2		5		3	1		3		3	2				1	1			
2	3	3	0	1	0	0		1	1	1	1		6	7	7	9		0	0	0	0	
2	3	3	0	1	0	2		1	1	1	1		4	5	7	8		0	0	0	0	
1	2	3	2	0	4	1	2	1	1	3	1	1	2	4	7	8	4	1	0	0	0	0
2	3	3	0	1	0	0	1	1	1	1	1	1	5	5	5	7	4	0	0	0	0	0
2	1	2	1	1		6		1	1		3		3	3		1		1	1		1	
2	2	3	0	2	3	1	2	1	1	2	1	1	3	4	7	7	2	1	0	0	0	1
2	2	3	0	2				1	1				5	4				0	0			
1	1	2	2					1					4					0				
1	1	2	0	1	2			1	1	1			3	5	6			1	0	0		
2	2	3	1	1	0	3	1	1	1	1	2	1	4	4	7	8	3	0	0	0	0	0
2	3	3	5	2				3	1				3	4				1	0			
2	2	3	0	1	0			1	1	1			5	5	5	2		0	0	0	1	
2	2	3	3		4			2		3			3		4			1		0		
2	2	3	0	1	0			1	1	1			4	3	7	7		0	1	0	0	
1	2	3	1	1	4	0	0	1	1	3	1	1	6	6	7	7	5	0	0	0	0	0
2	2	2	3	4	0	3	0	2	3	1	2	1	3	3	7	5	2	1	1	0	0	1
2	2	3	0	1	3	1	0	1	1	2	1	1	3	4	4	8	4	1	0	0	0	0
1	3	1	0	2	0	0	2	1	1	1	1	1	3	4	7	6	3	1	0	0	0	0
2	2	3	1	1	1	3	2	1	1	1	2	1	5	1	6	8	5	0	1	0	0	0
2	1	3	0	2	0	3	3	1	1	1	2	2	5	4	5	3	3	0	0	0	1	0
2	2	3	1	1				1	1				4	4				0	0			
2	2	3	0	4	3	1	1	1	3	2	1	1	3	2	1	4	5	1	1	1	0	0
2	2	3	3					2					5					0				
2	3	3	0	1	0	1		1	1	1	1		4	6	6			0	0	0		
2	2	3	0	0	0		0	1	1	1		1	5	6	7	8	5	0	0	0	0	0
2	1	3	2	1				1	1				3	3				1	1			
2	2	3	0					1					7					0				
2	1	3	1	3	1	2	1	1	2	1	1	1	3	5	3	3	7	1	0	1	1	0
2	2	2	2	0	0	2	0	1	1	1	1	1		4	4	1	1		0	0	1	1
2	3	3	0	1	0	0	1	1	1	1	1	1	4	4	7	5	7	0	0	0	0	0
2	2	2	0	3	0			1	2	1			4	3	6	7		0	1	0	0	
2	2	3	0	1	0	1	2	1	1	1	1	1	3	5	6	6	7	1	0	0	0	0

2	3	1	4	2	6	2		3	1	3	1		3	5	5	7		1	0	0	0	
3	3	3	1	1	1	0	0	1	1	1	1	1	5	6	7	7	3	0	0	0	0	0
2	2	3	0	0	1	1		1	1	1	1		3	4	4	5		1	0	0	0	
2	2	4	1	3	4	6	3	1	2	3	3	2	3	2	6	1	1	1	1	0	1	1
2	1	3		3	3	3			2	2	2		3	4	5	6		1	0	0	0	
2	2	3	0	0	2	3	1	1	1	1	2	1	5	4	3	4	4	0	0	1	0	0
2	2	3	0	1	0	0	1	1	1	1	1	1	4	5	7	8	6	0	0	0	0	0
2	2	3	0	0	1	1	1	1	1	1	1	1	5	5	7	7	4	0	0	0	0	0
2	2	2	1		0	6		1		1	3		5		7	3		0		0	1	
2	2	3	1	2	2	0	3	1	1	1	1	2	3	3	4	2	1	1	1	0	1	1
2	2	3	1	1	2	2	2	1	1	1	1	1	3	1	6	8	3	1	1	0	0	0
2	2	1	4	2	0	3	1	3	1	1	2	1	1	2	1	1	1	1	1	1	1	1
3	1	3	0					1					3					1				
2	2	3	0	1	1	1	0	1	1	1	1	1	5	5	6	8	6	0	0	0	0	0
2	2	3	1					1					4					0				
2	3	3	2	4	5	4	4	1	3	3	3	3	3	2	2	1	1	1	1	1	1	1
2	2	3	3	2	2	4		2	1	1	3		4	4	4	2		0	0	0	1	
2	2	1	0	1	3			1	1	2			5	4	3	2		0	0	1	1	
2	2	3	0	1	2	2	0	1	1	1	1	1	4	5	7	8	6	0	0	0	0	0
1	2	3	2					1					3					1				
2	2	3	1	1	0	3	1	1	1	1	2	1	3	5	6	8	3	1	0	0	0	0
2	1	2	0	1	1	0	0	1	1	1	1	1	4		2	4	5	0		1	0	0
1	2	2	1		0			1		1			3		7			1		0		
2	2	3	0	0	0	0		1	1	1	1		4	5	7	8		0	0	0	0	
2	2	3	0	1	1	0	1	1	1	1	1	1	5	7	2	9	7	0	0	1	0	0
2	2	3	0	1	2	1	2	1	1	1	1	1	4	6	6	4		0	0	0	0	
2	2	4	3	1				2	1				2	3		6		1	1		0	
2	2	1	4					3					2					1				
2	3	3	2	2	2	2		1	1	1	1		4	3		1		0	1		1	
2	3	3	1	0				1	1				2	7				1	0			
1	1	3	3	1	1	2		2	1	1	1		3	5	7	1		1	0	0	1	
2	2	3	1	2	2	0	1	1	1	1	1	1	4	5	6	9	7	0	0	0	0	0
2	2	3	3	2	3	2		2	1	2	1			3	2				1	1		
2	2	3	3	2	2	2	0	2	1	1	1	1	4	4	4	8	5	0	0	0	0	0
2	2	3	1	1	1	1	2	1	1	1	1	1		5	5	7	5		0	0	0	0
2	2	1	1	1	3	1	2	1	1	2	1	1	4	2	5	1	4	0	1	0	1	0
2	2	3	2	0	1	1	1	1	1	1	1	1	3	3	3	1	3	1	1	1	1	0
2	2	3	1	3	2			1	2	1			5	3	3			0	1	1		
2	2	3	0	1	0	2		1	1	1	1		4	4	4	5		0	0	0	0	
2	1	3	0		1	4	1	1		1	3	1	4		5	3	3	0		0	1	0
2	2	3	0	1	1	1	0	1	1	1	1	1	3	4	5	5	6	1	0	0	0	0

1	2	1	0		1			1		1			2		4			1		0		
1	2	1	2	2	5	4	1	1	1	3	3	1		2	4	8	6		1	0	0	0
2	2	3	1	1	3	3	0	1	1	2	2	1	4	7	5	8	2	0	0	0	0	1
2	2	3	0	2	1	1	0	1	1	1	1	1	4	5	7	8	7	0	0	0	0	0
1	2	1	2	2	7	2		1	1	3	1		2	3	3			1	1	1		
2	2	2	4	1	3	1	0	3	1	2	1	1	3	3	7	9	3	1	1	0	0	0
2	3	2	3	1				2	1				4	4				0	0			
2	2	3	0	2	4	1	4	1	1	3	1	3	4	5	5	3	4	0	0	0	1	0
2	2	2	1	4	0	1	3	1	3	1	1	2	5	7	7	9	6	0	0	0	0	0
1	2	2	4	3				3	2				3	7		9	5	1	0		0	0
2	2	3	1					1					4					0				
2	3	3	0	2	0			1	1	1			4	4	4			0	0	0		
1	1	1	2		3			1		2			2		2			1		1		
2	1	3	3	1	0	4	0	2	1	1	3	1		4	7		1		0	0		1
2	2	3	0	1	0			1	1	1			3	3	3	2		1	1	1	1	
2	3	2	7					3					3					1				
1	2	3	0	1	2			1	1	1			4	4	4			0	0	0		
2	1	3	0	2	0	1		1	1	1	1		4	4	7	6		0	0	0	0	
2	2	3	1					1					2					1				
1	2	3	0	0	0	0	1	1	1	1	1	1	4	4	6	8	4	0	0	0	0	0
2	1	3	1	1	0	1		1	1	1	1		3	4	5	4		1	0	0	0	
2	1	3	0	1	1	1		1	1	1	1		4	4	4	2		0	0	0	1	
1	2	3	3	0	0	2	1	2	1	1	1	1	1	3	4	3	4	1	1	0	1	0
3	1	3	3	0	1			2	1	1			4	4	3	2		0	0	1	1	
2	2	3	1	0	0	0		1	1	1	1		5	7	7	9		0	0	0	0	
1	2	1	2	2	2			1	1	1			4	5	6			0	0	0		
2	3	1	3		1	2		2		1	1				7	8				0	0	
2	1	3	0	1	0	0		1	1	1	1		1	3	5	4		1	1	0	0	
2	2	3	0	0	0	1		1	1	1	1		4	5	5	6		0	0	0	0	
1	2	1	0	0	2	1	1	1	1	1	1	1		2	2	3	6		1	1	1	0
2	1	3	2	0	0	2	0	1	1	1	1	1	3	3	3	7	1	1	1	1	0	1
1	3	3	2	3	3	1	1	1	2	2	1	1	4	2	5	5	3	0	1	0	0	0
2	1	3	0					1					4					0				
2	3	3	1	1	0	1	1	1	1	1	1	1	2	3	6	7	7	1	1	0	0	0
2	2	3	4	3	4	2		3	2	3	1		3	5	4	5		1	0	0	0	
2	3	3	0	1	0	1	1	1	1	1	1	1	3	4	5	6	1	1	0	0	0	1
2	2	3	3					2					4					0				
2	3	3	1	2	2	3	1	1	1	1	2	1	4	5	3	8	7	0	0	1	0	0
2	2	3	0		1			1		1			3		6			1		0		
2	3	3	1			3		1			2		2			2		1			1	
2	2	3	0	0	0	1		1	1	1	1		5	6	7	8		0	0	0	0	

2	2	2	2	0				1	1				3	3				1	1			
1	2	1	2					1					5					0				
2	2	1	4			6	0	3			3	1	2				1	1				1
2	3	2	0				1	1				1	3			7	3	1			0	0
2	1	3	1					1					4					0				
2	1	3	0	1	0	1		1	1	1	1		3	4	5	6		1	0	0	0	
2	2	2	2	1				1	1				4	3				0	1			
2	2	3	0	2	3	0	0	1	1	2	1	1	3	5	3	7	4	1	0	1	0	0
1	2	2	1	0	0	1		1	1	1	1			3	4	4			1	0	0	
2	2	1	3		4			2		3			3		4	2		1		0	1	
2	1	3	2	1	1	2	0	1	1	1	1	1	4	5	6	6	4	0	0	0	0	0
2	2	3	1	3	0	2	0	1	2	1	1	1	4	4	5	1	3	0	0	0	1	0
2	2	3	0	0	0	1		1	1	1	1		5	5	3			0	0	1		
2	2	3	0	1	1	1		1	1	1	1		5	3	7	4		0	1	0	0	
2	2	3	0					1					3					1				
2	1	3	0	0	0			1	1	1			2	5	2			1	0	1		
2	2	3	0	2	1	1	3	1	1	1	1	2	3	3	5	3	4	1	1	0	1	0
2	2	3	0	0	0	0	0	1	1	1	1	1	5		7		7	0		0		0
2	2	3	0	1	1	0	2	1	1	1	1	1	4	5		7	6	0	0		0	0
2	1	3	2		1	3		1		1	2		4		2	1		0		1	1	
2	2	3	2					1					4					0				
1	3	3	2	2	2	1	1	1	1	1	1	1	4	4	6	8	6	0	0	0	0	0
2	2	3	0	0	1	3		1	1	1	2		5	3	4	2		0	1	0	1	
2	1	3	1	3	0	1		1	2	1	1		3	3	3	2		1	1	1	1	
2	1	3	1	3	0	2		1	2	1	1		6	7	5	6		0	0	0	0	
2	3	3	0	1		1		1	1		1		4	6				0	0			
2	2	3	0	1	0	0	0	1	1	1	1	1	4	4	5	5	1	0	0	0	0	1
3	2	3	2	1	0	0		1	1	1	1		4	5	7	7		0	0	0	0	
2	2	3	0	0	2			1	1	1			4	3	3	2		0	1	1	1	
1	2	3	2	0	1			1	1	1			4	2	7	8		0	1	0	0	
1	2	3	0	1	1	2	1	1	1	1	1	1	4	4	5	2	3	0	0	0	1	0
2	2	3	0	1	0	1	0	1	1	1	1	1	5	7	7	9	7	0	0	0	0	0
3	1	3	0	1	0	0	0	1	1	1	1	1	4	6	5	3	4	0	0	0	1	0
2	3	3	0	0	1	3	0	1	1	1	2	1	3	4	7		7	1	0	0		0
2	2	3	0	0	0	1	2	1	1	1	1	1	3	5	6	7	6	1	0	0	0	0
2	3	3	0	2	0	2	3	1	1	1	1	2	4	5	7	8	6	0	0	0	0	0
2	1	3	4	2	1	3		3	1	1	2		3	4	7	7		1	0	0	0	
2	2	3	1	2	0	0	3	1	1	1	1	2	5	6	6	8	4	0	0	0	0	0
2	2	3	1		0	1		1		1	1		4		7	3		0		0	1	
2	2	3	1	1	0	2	2	1	1	1	1	1		6	7		3		0	0		0
1	2	3	0	1	0	1		1	1	1	1		5	5	7			0	0	0		

2	2	3	0	0	0	0	1	1	1	1	1	1	6	7	7	8	7	0	0	0	0	0
2	2	3	0	0	4			1	1	3			3	6	3	8	7	1	0	1	0	0
2	3	3	5	1	3	2		3	1	2	1		6	2	7	9		0	1	0	0	
2	1	3	0	2	1			1	1	1			3	3	5			1	1	0		
2	3	3	2		1			1		1			2		7	6		1		0	0	
2	2	3	0	1	0			1	1	1			4	5	7	4		0	0	0	0	
2	2	3	0	2	0	2	1	1	1	1	1	1	3	3	6	8	3	1	1	0	0	0
2	2	3	3	1				2	1				3	4				1	0			
2	2	3	3	2	0	0		2	1	1	1		5	4	7	8		0	0	0	0	
2	2	3	0	3	1			1	2	1			4	4				0	0			
2	1	3	0	0	0	3	1	1	1	1	2	1	4	4	4	8	4	0	0	0	0	0
1	3	1	4					3														
2	3	3	2	0	1	0		1	1	1	1		4	5	7	8		0	0	0	0	
2	2	1	3	0	1	1	0	2	1	1	1	1	7	4	4	7	1	0	0	0	0	1
2	2	3	3	3	1	3	4	2	2	1	2	3	4	3	6	2	2	0	1	0	1	1
1	1	1	1	0	2	1		1	1	1	1		2	7	5			1	0	0		
2	3	3	3					2					3					1				
2	1	3	0	2		3	0	1	1		2	1	3	2		8	1	1	1		0	1
2	2	3	0	1	0	2	4	1	1	1	1	3	4	5	7	8	1	0	0	0	0	1
2	1	3	1					1					3					1				
2	2	3	0	2	0	2	1	1	1	1	1	1	5	7	7	8	1	0	0	0	0	1
2	1	3	0	1	1	1	0	1	1	1	1	1	3	5	7	3	2	1	0	0	1	1
2	3	2	1	1	0	2	3	1	1	1	1	2	3	2	4	8	4	1	1	0	0	0
2	2	3	1	1	2	3		1	1	1	2		2	2	1	1		1	1	1	1	
2	2	3	0	0	0	3		1	1	1	2		3	4	5	4		1	0	0	0	
2	2	3	0	3	4			1	2	3			3	5	2			1	0	1		
2	3	1	2					1					2					1				
2	1	3	2		0			1		1			4					0				
2	2	3	1					1					4					0				
2	2	2	3					2					4					0				
2	3	3	0	2	2	3	1	1	1	1	2	1	5	6	7	7	5	0	0	0	0	0
2	3	3	1	1	1	1	1	1	1	1	1	1	5	4	4		5	0	0	0		0
2	2	3	0	1	0	2		1	1	1	1		3	3	4			1	1	0		
2	3	3	4	1	1	1		3	1	1	1		4	4	5	3		0	0	0	1	
2	2	3	0					1					3					1				
2	3	2	2					1					4					0				
1	2	3	3	7	1	4	3	2	3	1	3	2	3	3	5	8	4	1	1	0	0	0
2	2	3	0	0	1	2	0	1	1	1	1	1	5	6	2	8	4	0	0	1	0	0
2	2	3	1	0	1	3	1	1	1	1	2	1	3	4	6	8	5	1	0	0	0	0
2	2	2	0	2	0	4		1	1	1	3		5	4	4	3		0	0	0	1	
3	1	3	0					1					4					0				

2	1	3	0	0	0	0		1	1	1	1		3	4	6	1		1	0	0	1	
2	2	3	0	1	0			1	1	1			4	5	7			0	0	0		
2	2	3	4	1	0	1	2	3	1	1	1	1	4	3	5	3	6	0	1	0	1	0
2	2	2	1	4	3	1	0	1	3	2	1	1	4	4	7	8	3	0	0	0	0	0
2	2	3	1	1	3	2	2	1	1	2	1	1	4	5	4	8	3	0	0	0	0	0
3	2	3	1					1														
2	2	3	3	1	1	2	0	2	1	1	1	1	4	6	6	6	5	0	0	0	0	0
2	3	3	1					1					7					0				
3		3	1	1	2	6	3	1	1	1	3	2	6	4		2	2	0	0		1	1
2	2	3	1	2	4	3	0	1	1	3	2	1	4	5	3	1	1	0	0	1	1	1
2	2	3	1	2	1			1	1	1			3	4	5			1	0	0		
2	2	3	1	0	0	1	0	1	1	1	1	1	5	6	7	9	4	0	0	0	0	0
1	2	3	0	1	2			1	1	1			4		3			0		1		
3	3	3	1	0	1	0	0	1	1	1	1	1	4	5	5		3	0	0	0		0
2	2	3	1	1	0	0	1	1	1	1	1	1	3	7	6	4	5	1	0	0	0	0
2	3	3	0	0	0	0	0	1	1	1	1	1	4	7	7	8	3	0	0	0	0	0
2	2	3	1	1	0	2		1	1	1	1		6					0				
2	1	3	2					1					5					0				
2	2	3	2	2		0		1	1		1		4	4		7		0	0		0	
1	3	2	3		6	1	5	2		3	1	3	3		2	1	1	1		1	1	1
2	2	3	0		3	0	1	1		2	1	1	4		5	3	7	0		0	1	0
2	2	3	3	2	1	3	2	2	1	1	2	1		2	1	1	3		1	1	1	0
1	1	3	0	1	0	1		1	1	1	1		3	4	4	5		1	0	0	0	
2	1	1	2	2	5	3		1	1	3	2		2	5		1		1	0		1	
1	2		2					1					4					0				
2	2	4	2					1					3					1				
2	2	3	0	2	0			1	1	1			5		7	5		0		0	0	
2	3	3	0					1					5					0				
2	2	3	0	1	0	0		1	1	1	1		6	7	7	7		0	0	0	0	
1	2	1	2	1	3	3		1	1	2	2		4	4	3			0	0	1		
2	1	3	0	0		8		1	1		3		4	5				0	0			
2	1	3	3			5	5	2			3	3	4			1	1	0			1	1
2	2	3	1	0	1			1	1	1			4	2	7			0	1	0		
2	2	3	1	1	1	4		1	1	1	3		3	4	5	1		1	0	0	1	
2				0					1					4					0			
1	2	3	1					1					4					0				
1	2	3	2					1					5					0				
1	2	1	1	4	3	1	2	1	3	2	1	1	2	3	5	1	1	1	1	0	1	1
2	2	3	3					2					3					1				
2	2	3	3					2					3					1				
1	1	3	0	1	0			1	1	1			4	3	7			0	1	0		

1	2	3	1	2	3	0		1	1	2	1		4	7	7	9		0	0	0	0	
2	1	3	1	2	1	2	1	1	1	1	1	1	3	3	7	6	5	1	1	0	0	0
2	2	2	1	3	0	1	0	1	2	1	1	1	6	7	7	9	4	0	0	0	0	0
2		3	0	1	0	2	4	1	1	1	1	3	3	4	4	6	1	1	0	0	0	1
2	2	3	0	1	0	1		1	1	1	1		5	5	5	6		0	0	0	0	
2	3	3	2	0	1	1		1	1	1	1		5	7	7	9		0	0	0	0	
2		3	0	1	0	1		1	1	1	1			5	5	8			0	0	0	
1	1	1	2	5	2			1	3	1			2	2	1			1	1	1		
2	1	3	1	2	0	3		1	1	1	2		6	2	7	8		0	1	0	0	
2	2	3	0	0	0	1	0	1	1	1	1	1	4	4	4	4	6	0	0	0	0	0
2	3	3	0	0	1	1	0	1	1	1	1	1	4	5	6	8	4	0	0	0	0	0
1	2	1	5	1	4	1		3	1	3	1		2	4	2	2		1	0	1	1	
2	2	3	3	1	1			2	1	1			4	5	5	4		0	0	0	0	
2	3	1	0	0	0	2		1	1	1	1		5	2	2	2		0	1	1	1	
2	2	3	0	0	0			1	1	1			4	7	7			0	0	0		
1	2	2	3	3	3	4	0	2	2	2	3	1	2	3	4		7	1	1	0		0
2	2	3	1	0	0	8	2	1	1	1	3	1	3	3	3	2	2	1	1	1	1	1
2	1	2	1	0	1	2	3	1	1	1	1	2	4	5	7	5	3	0	0	0	0	0
2	2	3	1	2	0			1	1	1			4	4	4			0	0	0		
3	3	3	0	3	3	2		1	2	2	1		4	4	3	3		0	0	1	1	
2	1	3	0	1	1	2		1	1	1	1		4	4	4	3		0	0	0	1	
2	2	3	0					1					4					0				
2	2	3	0	2	0	3	3	1	1	1	2	2	3	6	7	8	7	1	0	0	0	0
2	2	3	0	2	2			1	1	1			3	3	4			1	1	0		
2	3	3	3	5	3	3	0	2	3	2	2	1	4	4	5	5	1	0	0	0	0	1
2	2	3	2	1	0	2	1	1	1	1	1	1	3	4	7	9	5	1	0	0	0	0
2	2	3	1	0	1	1	2	1	1	1	1	1	4	6	7	9	2	0	0	0	0	1
2	2	2	4					3					4					0				
2	2	1	2	3	4	4		1	2	3	3		3	3	3	2		1	1	1	1	
2	2	3	1		0			1		1			3		7			1		0		
2	1	3	0	0	0			1	1	1			3	5	6			1	0	0		
2	1	3	1					1					2					1				
2	3	3	1					1					5					0				
2	2	3	1	1	1	3	2	1	1	1	2	1	4	4	6	4	7	0	0	0	0	0
1	1	2	3					2					3					1				
2	2	3	1					1					4					0				
1	1	1	1				1	1				1	3				3	1				0
2	1	3	0					1					3					1				
2	2	2	2	1	2	3		1	1	1	2		3	6	3	2		1	0	1	1	
2	1	3	1	1	1			1	1	1			4	4	6	4		0	0	0	0	
1	1	3	4	4	3			3	3	2			2	4	7	4		1	0	0	0	

2	2	1	0	2	4	4		1	1	3	3		4	5	4	2		0	0	0	1	
2	3	3	4	3	1	3	1	3	2	1	2	1	2	7	7	4	2	1	0	0	0	1
2	2	3	2		2	4		1		1	3		3					1				
3	1	3	1	2	1	3	4	1	1	1	2	3	4	4	7	1	2	0	0	0	1	1
2	3	3	3			0		2			1		4			7		0			0	
2	2	3	1	0	1	0		1	1	1	1		3	2	3	4		1	1	1	0	
3	1	3	1	4				1	3				3	4	3	1		1	0	1	1	
2	3	3	1	2	1	2		1	1	1	1		4	4	7	6	6	0	0	0	0	0
2	2	2	1	4	2	3	1	1	3	1	2	1	4	6	7	7	1	0	0	0	0	1
2	3	2	1					1					4					0				
1	1	2	1	0	0			1	1	1			3	3	2			1	1	1		
2	1	3	0					1					5					0				
2	2	3	2	1	0	1	1	1	1	1	1	1	3	4	6	6	3	1	0	0	0	0
1	3	2	3					2					3					1				
1	2	2	1	2	4			1	1	3			4	4	4	2		0	0	0	1	
2	3	3	0					1					3					1				
2	2	3	0	0	0	0	2	1	1	1	1	1	4	7	7	7	6	0	0	0	0	0
2	1	3	1	1	1	1		1	1	1	1		4	4	5	3		0	0	0	1	
2	2	3	1	2	3	1	1	1	1	2	1	1	5	4	4	3	7	0	0	0	1	0
2	1	3	0	3	0	2		1	2	1	1		4	3	6	4		0	1	0	0	
1	2	3	5					3					6					0				
2	2	3	0	1	0	2		1	1	1	1		4	5	6	7		0	0	0	0	
2	2	3	1	0	1	0		1	1	1	1		5	6	6	8		0	0	0	0	
2	3	3	0	1	0	2		1	1	1	1		5	7	5			0	0	0		
2	3	3	0					1					4					0				
2	2	3	2	1	1	2	0	1	1	1	1	1	5		7	8	3	0		0	0	0
2	2	3	0	2	0	0	1	1	1	1	1	1	4	5	4	4	5	0	0	0	0	0
2	1	2	4	2	2			3	1	1			4	2	5			0	1	0		
3	3	3	1	1	1	0		1	1	1	1		4	5	2	1		0	0	1	1	
2	2	3	1	1	1			1	1	1			3	6	7			1	0	0		
2	1	3	1			0		1			1		4			2		0			1	
1	2	3	0	1	1	1	0	1	1	1	1	1	4	4	7	8	6	0	0	0	0	0
2	2	3	0	1	2	2	0	1	1	1	1	1	4	7	3	1	5	0	0	1	1	0
2	2	3	1	0				1	1				3	5				1	0			
2	2	3	0	2	4	0	0	1	1	3	1	1	5	5	1		3	0	0	1		0
2	2	3	3	1	1	5	5	2	1	1	3	3	3	6	7	5	4	1	0	0	0	0
2	3		1					1					3					1				
1	1	3	0	4	4	2		1	3	3	1		6	7	3	3		0	0	1	1	
2	1	4	2		1	4		1		1	3		4		2	1		0		1	1	
2	2	3	1	4	2	1	0	1	3	1	1	1	5	6	7	4	4	0	0	0	0	0
2	1	3	2	1	0	2		1	1	1	1		4	5	7	4		0	0	0	0	

1	2	1	4		0	2		3		1	1		3		3			1		1		
2	2	3	1	2	3		0	1	1	2		1	3	4	5	5	4	1	0	0	0	0
2	1	3	3					2					3					1				
2	2	1	2	1				1	1				3	3				1	1			
2	2	3	0					1					4					0				
1	3	1	2	1				1	1				5	2				0	1			
1	1	2	2	1	2			1	1	1			3	3	7	7		1	1	0	0	
2	3	3	5	2	0	1	0	3	1	1	1	1	1	3	5	8	7	1	1	0	0	0
2	1	3	1	3	3			1	2	2			5	5	4			0	0	0		
3	3	3	3	1	4			2	1	3			4	3	2			0	1	1		
2	2	3	0	0	0			1	1	1			3	4	7			1	0	0		
2	3	3	2					1					4					0				
2	2	3	1	4	4	1		1	3	3	1		4	6	2			0	0	1		
2	2	3	3	3	7	4	2	2	2	3	3	1		3	7	2	5		1	0	1	0
2	1	2	4	1	2	5	2	3	1	1	3	1	2	3	4	1	1	1	1	0	1	1
2	1	2	1					1					3					1				
2	2	3	1	2	0	1		1	1	1	1			5	7				0	0		
2	2	3	1	0	1	4		1	1	1	3		4	5		5		0	0		0	
2	2	3	2					1					5					0				
2	3	3	6	3	2	2	0	3	2	1	1	1	4	5	6	3	3	0	0	0	1	0
1	2		1	0	2			1	1	1				6	2				0	1		
2	2	3	2	2	1	2		1	1	1	1		4	5	6			0	0	0		
3	2	3	1	0				1	1				4					0				
1	1	1	5	4				3	3				2	3				1	1			
2	3	2	0	2				1	1				6	2		7		0	1		0	
2	2	2	0	0	0	0	0	1	1	1	1	1	3	2	4	1	4	1	1	0	1	0
1			1					1														
2	2	3	0	1	0	6		1	1	1	3		4	6	5	8		0	0	0	0	
2	2	3	1	0	0	2		1	1	1	1		3	4	7	7		1	0	0	0	
2	2	3	1	3	2	2		1	2	1	1		3	4	5			1	0	0		
2	1	3	1	0	0			1	1	1			3	3	2			1	1	1		
3	2		1	2	3			1	1	2			5					0				
2	2	3	0	1	0	0	0	1	1	1	1	1	5	6	7	7	7	0	0	0	0	0
2	2	3	0	3	0	3		1	2	1	2		3	6	6	7		1	0	0	0	
1	2	1	3	4	2			2	3	1			3	4	5			1	0	0		
2	2	3	2	1	1	0		1	1	1	1		3	2	3	1	5	1	1	1	1	0
2	2	2	0	0	0	0		1	1	1	1		6	4	5	2		0	0	0	1	
2	3	3	4	1				3	1				4	2				0	1			
2	3	2	1					1					3					1				
2	1	4	4					3					3					1				
2	2	3	3	1	2	1	1	2	1	1	1	1	3	4	3	9	4	1	0	1	0	0

2	2	3	0					1					4					0				
2	1	3	1	1	1	1		1	1	1	1		4	3	2	3		0	1	1	1	
2	1	2	1					1					2					1				
2	3	2	4	6	2	3	1	3	3	1	2	1	7	2	7	9	4	0	1	0	0	0
2	2	3	1	3	6	2	1	1	2	3	1	1	3	3	3	5	3	1	1	1	0	0
2	3	3	1		6			1		3			3		1			1		1		
2	1	3	0	0	1	7	0	1	1	1	3	1	3	3	5	3	1	1	1	0	1	1
2	3	3	1	0	0	2	1	1	1	1	1	1	4	3		2	3	0	1		1	0
1	2	2	1	0				1	1				3	2				1	1			
2	2	3	0	1	1	0	0	1	1	1	1	1	5	4	4		3	0	0	0		0
2	2	2	1	0				1	1				4	6				0	0			
1	1	2	0					1								8					0	
2	3	3	0	1	0	2	1	1	1	1	1	1	3	4	5	6	7	1	0	0	0	0
1	2	3	0	2	5	2	2	1	1	3	1	1	4	4	5	7	5	0	0	0	0	0
2	3	3	0	1	0	2	0	1	1	1	1	1	4	5			7	0	0			0
2	2	3	1					1					3					1				
2	2	3	1	2	0	2	3	1	1	1	1	2	5	6	6	7	1	0	0	0	0	1
1	1	1	0	1	1	4		1	1	1	3		2	3	3	1		1	1	1	1	
2	2	3	0	1	0		0	1	1	1		1	4	5	7		5	0	0	0		0
2	2	1	1	2	2	0	0	1	1	1	1	1		4	6	1	4		0	0	1	0
2	3	3	3	1	3	1		2	1	2	1		4	7	6	8		0	0	0	0	
2	3	2	3	2	1	2		2	1	1	1		3	3	2	2		1	1	1	1	
2	2	3	0	1	1	1		1	1	1	1		5	7	7	8		0	0	0	0	
2	3	3	1	0	1	0	0	1	1	1	1	1	4	7	7	8		0	0	0	0	
1	3	3	0	3	3	3		1	2	2	2			4	7	3			0	0	1	
2	3	3	2	2	1	1	0	1	1	1	1	1	3	5	5	7		1	0	0	0	
2	2	3	2	1	0	1	1	1	1	1	1	1	4	6	7			0	0	0		
3	2	2	1					1					5					0				
2	2	3	1		2	2	1	1		1	1	1	3		2	1	1	1		1	1	1
2	2	3	1					1					3					1				
2	3	2	5	2	5			3	1	3			2	2	5	7		1	1	0	0	
2	3	3	0	2	2			1	1	1			3	4	5			1	0	0		
1	1	2	2	3	4	1		1	2	3	1		4	6	3	8		0	0	1	0	
1	2	3	1	3	0			1	2	1			2	2	6			1	1	0		
2	2	3	1					1					6					0				
2	2	1	1	1	5			1	1	3				3	6				1	0		
1	2	3	0					1					5					0				
2	2	3	1	2	4	2	0	1	1	3	1	1	3	5	1		4	1	0	1		0
2	2	3	0					1					4					0				
2	2	3	0	0	2			1	1	1			1	4	7			1	0	0		
2	2	3	2	1	2	0		1	1	1	1		3	7	5	5	3	1	0	0	0	0

1	2	3	1		1			1		1			3		3	6		1		1	0	
1	1	1	0	1	1	2	0	1	1	1	1	1	3	2	5	3	5	1	1	0	1	0
2	2	3	0	1	0	1	1	1	1	1	1	1	6	7	6	4	5	0	0	0	0	0
2	2	1	1	0	2	2	0	1	1	1	1	1	3	3	5	3	1	1	1	0	1	1
1	1	3	0					1					3					1				
2	2	3	2	2	1	4	3	1	1	1	3	2	3	3	3	7	1	1	1	1	0	1
2	2	3	1	0	0	1		1	1	1	1		3		7	7		1		0	0	
1	2	1	2	2	5	6		1	1	3	3		4	5	3	9		0	0	1	0	
2	2	3	4	0	2	0	0	3	1	1	1	1	3	2	3	5	3	1	1	1	0	0
2	1	3	1	1	1	0	0	1	1	1	1	1	5	7	7	9	4	0	0	0	0	0
1	2	3	0	2	1	3	2	1	1	1	2	1	4	5	7	9	7	0	0	0	0	0
2	2	3	1					1					2					1				
2	2	3	0					1					4					0				
1	1	2	1	3	4	4	0	1	2	3	3	1	4	3	7	4	6	0	1	0	0	0
2	2	3	0	0	0	2		1	1	1	1		4	6	6	2		0	0	0	1	
1	2	3	0	1				1	1				4	3				0	1			
1	2	3	0	2	1	4	2	1	1	1	3	1		4	6	7	1		0	0	0	1
2	2	3	0	0	0	2	0	1	1	1	1	1	5	4	4	2	3	0	0	0	1	0
2	2	3	1	1	1	4		1	1	1	3		5	6	7	8		0	0	0	0	
2	2	3	1	1	2			1	1	1			3	5	7	1		1	0	0	1	
2	3	3	1	2	2			1	1	1			3	6	7			1	0	0		
3	1	3	1	0	6			1	1	3			5	7	7			0	0	0		
2	2	3	1	1	1	0	0	1	1	1	1	1	6	4	4	2	6	0	0	0	1	0
3	2	3	0		1			1		1					3					1		
3	1	3	2		2			1		1			4		1	1		0		1	1	
2	2	2	3	1	0	2		2	1	1	1		3	6	6	4		1	0	0	0	
1	2	1	3	1	2	1	0	2	1	1	1	1	5	7	7	8	6	0	0	0	0	0
1	2	2	1		0			1		1			5		6	1		0		0	1	
3	1	3	0	2	0			1	1	1			3	5	4	5		1	0	0	0	
1	2	4	4	2	6	5		3	1	3	3		2	5	6	2		1	0	0	1	
2	3	3	0	2	1	0	0	1	1	1	1	1	5	7	5	7	6	0	0	0	0	0
2	2	3	0	1	0			1	1	1			5	7	7			0	0	0		
2	2	3	2	0	0	1	0	1	1	1	1	1	5	6	7	9	7	0	0	0	0	0
2	3	2	0	3	0	1		1	2	1	1		5	4	5	3		0	0	0	1	
2	3	3	1		0			1		1												
2	2	3	4		2	2		3		1	1		3		7			1		0		
2	3	3	4	1	1	1		3	1	1	1		6	7	7	8		0	0	0	0	
2	1	1	1		0			1		1			2		5			1		0		
2	2	3	3					2					3					1				
2	2	1	0		2	2		1		1	1		6		6	4		0		0	0	
2	2	3	1	0	0	1	1	1	1	1	1	1	6	6	7	8	5	0	0	0	0	0

2	2	3	1	1	0			1	1	1			4	5	4			0	0	0		
2	2	3	0	1	1			1	1	1			3	3	7			1	1	0		
2	2	3	1	0	2	2	4	1	1	1	1	3		4	4	7	7		0	0	0	0
2	2	3	0					1					5					0				
2	1	3	0	3	4	2	0	1	2	3	1	1	3	3	2	1	3	1	1	1	1	0
2	2	3	0	1	3	0		1	1	2	1		3	3	6	7		1	1	0	0	
2	2	3	0	2	1	6	1	1	1	1	3	1	6	2	5	4	6	0	1	0	0	0
2	2	3	2		2	1	1	1		1	1	1	4		4	3	4	0		0	1	0
2	2	3	1	2	2	1		1	1	1	1		3	4	6	3		1	0	0	1	
1	2	2	3		1			2		1			1		4			1		0		
2	2	2	0	0	1	4		1	1	1	3		3	3	3	5		1	1	1	0	
2	3	3	1	2	1	0	4	1	1	1	1	3	5	5	6	9	6	0	0	0	0	0
2	1	3	1	1		5	2	1	1		3	1	4	2		4	2	0	1		0	1
2	2	3	1	3	0	2	0	1	2	1	1	1	3	4			3	1	0			0
2	2	3	0	3	1	2	3	1	2	1	1	2	4	3	7	1	5	0	1	0	1	0
2	2	3	0	0	2	2		1	1	1	1		4	4	2	2		0	0	1	1	
2	2	3	3	4	2	2		2	3	1	1		4	3	3	4		0	1	1	0	
2	2	3	0	0	0			1	1	1			4	4				0	0			
2	1	3	3	5	3	1	2	2	3	2	1	1	3		4	1	1	1		0	1	1
2	1	3	0					1					4					0				
2	2	3	1	2	0	3	2	1	1	1	2	1	4	4	7	6	7	0	0	0	0	0
2	2	3	0	0	2	3	0	1	1	1	2	1	4	5	5	6	3	0	0	0	0	0
1	1	3	3	5				2	3				4	2				0	1			
2	2	3	3	4	2	2	2	2	3	1	1	1		4	4	2	4		0	0	1	0
1	1	1	1	1	0	1		1	1	1	1		3	3	6	9		1	1	0	0	
2	1	3	2	2	2	2		1	1	1	1		3	6	7	9		1	0	0	0	
2	2	3	2	3	0	3	4	1	2	1	2	3	3	2	1	2	3	1	1	1	1	0
2	1	2	0	2	1	0		1	1	1	1		4	3	7	3		0	1	0	1	
2	2	4	2					1														
2	2	2	5					3					3					1				
2	3	3	1					1					5					0				
2	2	3	1	2	2	3	0	1	1	1	2	1	4	5	7	4	4	0	0	0	0	0
3	3	3	0	3	1	4		1	2	1	3		5	6	7	3		0	0	0	1	
2	2	3	1	0	1		2	1	1	1		1	5	6	5		5	0	0	0		0
2		3	3	1	1			2	1	1			5	3	4	1		0	1	0	1	
2	2	3	0	2	0	3	0	1	1	1	2	1	4	3	7	7	1	0	1	0	0	1
2	2	3	0	2	0	1	0	1	1	1	1	1	6	6	7	7	7	0	0	0	0	0
2	2	3	0	1	1	1	3	1	1	1	1	2	4	6	6	8	3	0	0	0	0	0
1	1	1	2				2	1				1	4				7	0				0
2	2	3	0					1					3					1				
2	2	3	2	0	1	6		1	1	1	3		2	4	5	7		1	0	0	0	

2	2	3	0	0	1	2		1	1	1	1		3	3	3	6		1	1	1	0	
2	2	3	1	2	2	4		1	1	1	3		3	4	4	1		1	0	0	1	
2	2	3	0	2	1	1	2	1	1	1	1	1	4	6	7	8	7	0	0	0	0	0
2	2	3	1	2	0	2		1	1	1	1		4	3	7	9		0	1	0	0	
2	2	3	1	1	1	1	3	1	1	1	1	2	3	4	4	3		1	0	0	1	
2	3	3	0	5	1	0	1	1	3	1	1	1	4	4	7		4	0	0	0		0
2	1	3	0	4	3	5		1	3	2	3			3	2	1			1	1	1	
2	3	3	0	5	2	1		1	3	1	1		5	2	3	6		0	1	1	0	
1	3	3	6			5		3			3		3			7		1			0	
2	2	2	1	3				1	2				3	2				1	1			
1	1	3	0	0	0	2		1	1	1	1		3	4	5	6		1	0	0	0	
2	2	3	0					1					4					0				
1	2	2	1	1	1	2		1	1	1	1		3	2	5	5		1	1	0	0	
2	1	3	0	2	4	2	1	1	1	3	1	1	5	6	2	3	3	0	0	1	1	0
1	2	1	3		7	2		2		3	1		3		6	3		1		0	1	
2	2	3	2	3	3	3		1	2	2	2		3	7	3	7		1	0	1	0	
2	2	3	0	0	0	0	0	1	1	1	1	1	5	6	7	8		0	0	0	0	
2	2	3	2					1					4					0				
2	2	3	0	1	0	0	1	1	1	1	1	1	4	6	7	7	5	0	0	0	0	0
2	3	3	0	1	0	0	0	1	1	1	1	1	7	7	7	9	7	0	0	0	0	0
2	2	3	1	2	2	0	1	1	1	1	1	1	3	6	7	7	6	1	0	0	0	0
2	2	3	1		1			1		1			4		7	5		0		0	0	
2	2	3	2	0	1	1	1	1	1	1	1	1	3	3	4	1	2	1	1	0	1	1
1	2	1	2		1	2		1		1	1		5		7	9		0		0	0	
2	2	3	2					1					3					1				
2	2	3	1	2	2	4		1	1	1	3		3	7	5	7	4	1	0	0	0	0
2	3	3	4	3	0			3	2	1			4	3	7			0	1	0		
1	1	1	0			5		1			3		3					1				
2	2	3	3	3	5	1		2	2	3	1		4	5	2			0	0	1		
2	2	3	1	2	0	5		1	1	1	3		4	7	6	8		0	0	0	0	
2	3	3	0	2	1	0		1	1	1	1		4	4	2	3		0	0	1	1	
2	1	3	0	2	0	0		1	1	1	1		4	4	5	1		0	0	0	1	
2	1	3	1	3	0	4	1	1	2	1	3	1	4	2	3	5	2	0	1	1	0	1
1	2	1	3	1	1	7		2	1	1	3			5	3	1			0	1	1	
2	2	3	0	0	0	1	0	1	1	1	1	1	5	4	4	1	3	0	0	0	1	0
2	1	3	1	1	2	0		1	1	1	1		4	4	4	3		0	0	0	1	
2	1	3	1					1					3					1				
2	2	3	0	1	0	2		1	1	1	1		2	4	7	4		1	0	0	0	
2	2	4	5	5	3	5		3	3	2	3		2	3	5	1		1	1	0	1	
2	2	3	1	0	3	3		1	1	2	2		4	4	5	7		0	0	0	0	
3	3	3	0	3	5	0		1	2	3	1		5	5	6	7		0	0	0	0	

2	2	3	1		1	3		1		1	2		5		6			0		0		
2	2	3	0	0	3	3		1	1	2	2		3	4	2			1	0	1		
2	2	2	0	1	2	1		1	1	1	1		3	3	3	3		1	1	1	1	
1	2	3	1	1				1	1				3	4				1	0			
2	1	3	0	1	1			1	1	1			4	3	3			0	1	1		
2	3	3	1	1	1	0		1	1	1	1		4	6	7	7		0	0	0	0	
2	2	1	0	2	0	1		1	1	1	1			3	7	7	6		1	0	0	0
2	2	3	2					1					3			3		1			1	
1	2	3	1			4		1			3		4					0				
1	1	1	0	1	1		0	1	1	1		1	3	2	7		4	1	1	0		0
2	2	3	1	2				1	1				4	3				0	1			
2	2	3	1					1					6					0				
2	2	3	1	0	1	1	1	1	1	1	1	1	4	5	5	7	7	0	0	0	0	0
2	2	3	1	1	3	2	4	1	1	2	1	3	5	5	3	4	4	0	0	1	0	0
2	3	3	5		6			3		3			4		2			0		1		
2	2	3	2	1	1	4	3	1	1	1	3	2	5	4	7	9	3	0	0	0	0	0
2	1	3	2	3	1	1		1	2	1	1		6	6	7	9		0	0	0	0	
2	2	3	0	2	2	1	0	1	1	1	1	1	4	5	3	9	7	0	0	1	0	0
2	2	3	1	0	0	0	1	1	1	1	1	1	3	6	6	1	3	1	0	0	1	0
1	2	3	1	4	3	2	1	1	3	2	1	1	4	3	7	4	7	0	1	0	0	0
1	2	3	0	1	4	6		1	1	3	3		3	5	7	5		1	0	0	0	
1	2	2	6			2	1	3			1	1	3			2	3	1			1	0
1	2	3	3		4	6		2		3	3		3		4	3		1		0	1	
2	2	3	1	1	1	1	1	1	1	1	1	1	7	7	7	9	7	0	0	0	0	0
2	2	3	2		0	3		1		1	2		3		4	7		1		0	0	
2	3	3	1		3	0	1	1		2	1	1	6		7		7	0		0		0
1	2	2	5	4	5	8		3	3	3	3		2	4	6	4		1	0	0	0	
2	1	3	0					1					4					0				
2	1	2	0	1	0			1	1	1			3	3	4	3		1	1	0	1	
2	2	3	0					1					4					0				
2	2	3	3	3	1	2	1	2	2	1	1	1	3	4	7	7	7	1	0	0	0	0
2	1	4	3		4	1		2		3	1		2		1		6	1		1		0
1	2	1	4	2		5	2	3	1		3	1	3	4		2	3	1	0		1	0
1	2	2	0					1					4					0				
2	1	2	2	1	2			1	1	1			4	2	4			0	1	0		
2	2	2	0	1	1	1	2	1	1	1	1	1	5	6	7	9	7	0	0	0	0	0
1	2	3	1	2	0	0	8	1	1	1	1	3	3	6	7	8	4	1	0	0	0	0
2	3	3	3	1	0	1	4	2	1	1	1	3	3	2	4	8	3	1	1	0	0	0
2	3	3	1	1	1	4	1	1	1	1	3	1	3	5	5	7	3	1	0	0	0	0
1	2	2	1	1				1	1					2					1			
2	2	2	4	1	2	1	1	3	1	1	1	1	3	2	2	4	3	1	1	1	0	0

2	2	3		0	5	3			1	3	2		4	4	6	3		0	0	0	1	
2	2	3	2	1				1	1													
2	2	3	0	1	0	1		1	1	1	1		3	4	7			1	0	0		
2	2	3	1	3	5	3	2	1	2	3	2	1	3	3	4	4	2	1	1	0	0	1
2	2	3	0	2	0	2		1	1	1	1		3	6	4			1	0	0		
2	2	3	2	1	0	2	3	1	1	1	1	2	4	5	7	7	4	0	0	0	0	0
2	1	3	1	5	2	8		1	3	1	3		4	2	2	2		0	1	1	1	
2	2	3	0	4	2	1		1	3	1	1		5	6	7	8		0	0	0	0	
2	2	3	2	6	3		0	1	3	2		1	4	2	3		1	0	1	1		1
2	2	3	1	0	0	3		1	1	1	2		4	7	6	5		0	0	0	0	
2	2	3	1					1					4					0				
2	2	3	0	1	0	1	2	1	1	1	1	1	4	6	7	9	2	0	0	0	0	1
2	2	3	0	3	0	5		1	2	1	3		3	3	3			1	1	1		
2	2	2	4	2	0	2		3	1	1	1		5	4	7	7		0	0	0	0	
2	1	3	1	2	0	2	0	1	1	1	1	1		5	7	8	1		0	0	0	1
2	2	2	1	1	0	3	0	1	1	1	2	1	4	2	7	7	5	0	1	0	0	0
1	2	2	2		1	1		1		1	1		3		4	3		1		0	1	
2	2	3	0	0	6			1	1	3			4	3	4			0	1	0		
2	1	2	4	2	1	5		3	1	1	3		3	3	4	4	4	1	1	0	0	0
2	2	3	3	2	2	6		2	1	1	3		2	3	2	1		1	1	1	1	
2	2	3	0	1	1	2	1	1	1	1	1	1	4	4	7	6	3	0	0	0	0	0
2	3	3	1	1	1	2	0	1	1	1	1	1	4	4			3	0	0			0
2	2	3	1	0				1	1				3					1				
2	2	3	1					1					5					0				
2	2	3	0	1				1	1				4	4				0	0			
1	2	1	1					1					2					1				
1	1	3	0	1	1	1	0	1	1	1	1	1	3	3	5	4	3	1	1	0	0	0
2	1	1	0					1					1					1				
2	3	3	1	2	1	3		1	1	1	2		4	7	7	7		0	0	0	0	
2	2	3	1	3	3			1	2	2			4	3	3			0	1	1		
2	2	2	1	1	1	5		1	1	1	3		5	6	7	3		0	0	0	1	
1	2	1	1					1					2					1				
2	2	3	4	1	0	3	0	3	1	1	2	1	4	2	4	2	1	0	1	0	1	1
2	2	3	1		1	3	0	1		1	2	1	3		6	2	4	1		0	1	0
2	2	3	1					1					4					0				
2	3	3	0	2	1	1	0	1	1	1	1	1	4	4	4		5	0	0	0		0
2	2	2	3		5	5		2		3	3		3		2	4		1		1	0	
2	2	3	0	1	4	4	1	1	1	3	3	1	4	4	2	1	1	0	0	1	1	1
2	2	3	1	0	2	0		1	1	1	1		3	3	3	1		1	1	1	1	
2	3	3	2	3				1	2				5	2				0	1			
2	3	3	0		2	1		1		1	1		4		1	2		0		1	1	

2	2	3	0		0	1	4	1		1	1	3	4		7	7	3	0		0	0	0
2	2	3	2					1					5					0				
2	1	3	0	1	3	1		1	1	2	1		4	3	3			0	1	1		
2	2	3	0					1					4					0				
2	2	2	2					1					4					0				
1	2	2	0	2				1	1				4	2				0	1			
2	2	3																				
2	2	3	1	0	2		1	1	1	1		1	4	5	4	3	3	0	0	0	1	0
2	2	2	1	0	0	1		1	1	1	1		7	3	7	9		0	1	0	0	
2	2	2	1	2	1	4	3	1	1	1	3	2	5	5	7	4	3	0	0	0	0	0
2	1	3	0	2				1	1				3	6				1	0			
2	2	3	3	1	0	0		2	1	1	1		4	6	7	9		0	0	0	0	
2	2	3	0	3	0			1	2	1			4	3	6	3		0	1	0	1	
2	3	3	0	2	1			1	1	1			4	6	6			0	0	0		
2	2	3	1	0	0			1	1	1			5	6	5			0	0	0		
1	2	1	0		2		0	1		1		1	1		3	5	1	1		1	0	1
2	2	3	0	1	0	2		1	1	1	1		5	6	7		7	0	0	0		0
2	3	3	0	2	0	1		1	1	1	1		7	7	7	9		0	0	0	0	
2	2	3	0					1					3					1				
2	2	3	3	2	0	0	2	2	1	1	1	1	3	3	4	4	2	1	1	0	0	1
1	1	3	2					1					3					1				
2	2	3	5	2	2	3		3	1	1	2		2	4	3	2		1	0	1	1	
3	2	3	0	3	0			1	2	1			3	3	5			1	1	0		
2	2	3	3	2	0	0		2	1	1	1		4	5	7	7		0	0	0	0	
2	2	3	0		1	3		1		1	2		3		5			1		0		
2	2	3	2	2	2			1	1	1			6		7			0		0		
2	2	3	0	2	1	1	1	1	1	1	1	1		6		8	4		0		0	0
2	2	3	2		0		1	1		1		1	6		7	9	6	0		0	0	0
2	3	3	3	2	1	1	0	2	1	1	1	1	3	7	6	6	2	1	0	0	0	1
2	3	3	2	2	0	1	0	1	1	1	1	1	3	5	6		4	1	0	0		0
2	1	3	2					1					4					0				
2	2	3	3	3	3	2	2	2	2	2	1	1	4	2	6	6	4	0	1	0	0	0
2	1	3	0		1	2		1		1	1		3		7	6		1		0	0	
2	2	3	1	1	1			1	1	1			4	5	7			0	0	0		
2	3	3	1	0	2	1	0	1	1	1	1	1	4	5	5	4	3	0	0	0	0	0
2	3	3	3					2					3					1				
2		2	4	3	1	2	1	3	2	1	1	1		5	1	1	1		0	1	1	1
2	2	3	1	0	0			1	1	1			5	4	7			0	0	0		
2	1	3	1	2	0	3	1	1	1	1	2	1	3	5	5	7	1	1	0	0	0	1
2	3	3	2	2	0	1		1	1	1	1		6	7	7	8		0	0	0	0	
2	2	3	0	3	0			1	2	1				5	7	8			0	0	0	

2	2	3	1		1	1	1	1		1	1	1	4		5	6	3	0		0	0	0
2	2	3	1	2	0	4		1	1	1	3		3	4	7	8		1	0	0	0	
1	2	1	1	0				1	1				2	4				1	0			
2	1	3	1	2	0	2	0	1	1	1	1	1	3	6	3	2	5	1	0	1	1	0
2	2	3	1	4	0	4	1	1	3	1	3	1	6	2	7	8	6	0	1	0	0	0
1	1	1	0	0	3			1	1	2			2		3			1		1		
2	1	3	1	2	3	1	1	1	1	2	1	1	5	6	7	7	6	0	0	0	0	0
2	1	3	0	2	1			1	1	1			4	3	3	2		0	1	1	1	
2	2	4	6	2	3	1	0	3	1	2	1	1	3	3	3	6	6	1	1	1	0	0
2	2	3	3	2	4	3		2	1	3	2		4	6	3	8		0	0	1	0	
1	1	3	2	5	3			1	3	2			3	3	5		1	1	1	0		1
2	2	3	2	3	1			1	2	1			6	7	6	8		0	0	0	0	
2	1	2	0	2				1	1				6	5				0	0			
2	2	3	0	0	2	2	3	1	1	1	1	2	7	6	7		3	0	0	0		0
2	2	3	2	3	0	2	0	1	2	1	1	1	6	6	6	7	4	0	0	0	0	0
2	3	3	4	1	1	4	2	3	1	1	3	1	3	4	5	7	4	1	0	0	0	0
2	1	3	0	0				1	1				3	4				1	0			
2	3	3	3					2					5					0				
2	2	2	1	5		4		1	3		3		7	3		6		0	1		0	
2	1	3	0	1	1			1	1	1			5	7	7			0	0	0		
2	1	3	0	1	1		0	1	1	1		1	4		5		4	0		0		0
2	2	3	1	0	0	2		1	1	1	1		3	3	4	5		1	1	0	0	
2	2	3	0	1	1	2	0	1	1	1	1	1	4	5	4	2	3	0	0	0	1	0
1	2	3	3	4	1	4	1	2	3	1	3	1	3	3	6	8	3	1	1	0	0	0
2	2	3	0	2	0	1	1	1	1	1	1	1	4	5	6	3	1	0	0	0	1	1
2	2	3	3	2	5	0	1	2	1	3	1	1	1	2	2	1	5	1	1	1	1	0
2	2	4	0		1			1		1			4		2			0		1		
2	2	3	5	5	3	0		3	3	2	1		5	3	5	6		0	1	0	0	
2	2	3	2	1	1	2	4	1	1	1	1	3	4	3	2	1	4	0	1	1	1	0
2	1	3	3	3	3			2	2	2			3	2	1	1		1	1	1	1	
2	3	3	0	2	1	2		1	1	1	1		4	3	3	2		0	1	1	1	
1	1	2	2	1	1			1	1	1			6	4	3	3		0	0	1	1	
3	3	3	3	1	0	2	0	2	1	1	1	1	5	7	7	9	5	0	0	0	0	0
2	2	3	0					1														
2	2	2	3	4	3			2	3	2			4	3	3	2		0	1	1	1	
2	2	3	0					1					3					1				
1	1	1	4	5				3	3				2	2				1	1			
1	1	1	2	1	2			1	1	1			3	3	5	1		1	1	0	1	
2	2	3	1					1					4					0				
2	3	3	0	2	1	1	1	1	1	1	1	1	5	5	7	7	7	0	0	0	0	0
1	2	3	1	1	1	1		1	1	1	1		4	4	7	8		0	0	0	0	

2	2	3	0	1	0	1	2	1	1	1	1	1	4	6	7	6	2	0	0	0	0	1
2	2	3	0					1														
2	2	3	1	2	0	2	0	1	1	1	1	1	5	4	7	8	4	0	0	0	0	0
1	3	3	3	4	3	3	4	2	3	2	2	3	4	4	7	8	3	0	0	0	0	0
2	2	1	6	1		0		3	1		1		2	2		7		1	1		0	
2	2	2	1		3			1		2			5		1			0		1		
2	2	3	1	0	0	1		1	1	1	1		4	4	4	4		0	0	0	0	
2	1	3	5		0	2		3		1	1		3		7	8		1		0	0	
2	2	3	0		1	1		1		1	1		6		3	3		0		1	1	
1	2	2	1		0			1		1			5		5			0		0		
2	3	3	2	1				1	1				5	5				0	0			
2	1	3	1	0				1	1					4					0			
2	3	3	1	2	5	5		1	1	3	3		5	3	2	1		0	1	1	1	
2	1	1	4					3					3					1				
2	2	4	1					1					2					1				
2	1	2	3	2				2	1				3	3				1	1			
1	2	2	2					1					3					1				
1	2	3	4	1	1	3		3	1	1	2		4	3	3	3		0	1	1	1	
1	2	1	1		4			1		3			7		5			0		0		
2	2	3	0	0	0	2	0	1	1	1	1	1	5	6	7	9	3	0	0	0	0	0
3	2	3	2	1	4	4		1	1	3	3		5	4	2	9		0	0	1	0	
2	2	3	0	0	1			1	1	1			6	5	7	7		0	0	0	0	
2	3	3	1					1					5					0				
2	2	3	0	2	1	1	0	1	1	1	1	1	5	3	6	6	4	0	1	0	0	0
2	2	3	1	3	0	2	2	1	2	1	1	1	4	5	5	7	4	0	0	0	0	0
2	1	3	0	2	0	1		1	1	1	1		3	4	6	2		1	0	0	1	
2	2	3	2		1	1		1		1	1		3		2	3		1		1	1	
1	3	3	0	1	1	4	1	1	1	1	3	1	3	3	6	8	1	1	1	0	0	1
1	2	3	0	0	0			1	1	1			3	2	4			1	1	0		
1	2	3	1	1	2	6		1	1	1	3		4	3	3	1		0	1	1	1	
2	1	1	2	1	0	2	1	1	1	1	1	1	2	2	4	4	6	1	1	0	0	0
2	1	3	1	1	0	0		1	1	1	1		3	2	5	1		1	1	0	1	
2	2	2	3					2					3					1				
2	1	2	0	0	0	3		1	1	1	2		4	4		6		0	0		0	
1	1	1	1	1				1	1				5	2				0	1			
3	1	3	3	1	3			2	1	2			4	3	2			0	1	1		
1	2	3	4	5	2	3		3	3	1	2		3	2	3	9		1	1	1	0	
2	2	3	4	1	2	2	0	3	1	1	1	1	4	4	6	3	5	0	0	0	1	0
2	2	3	2	3	3	8	3	1	2	2	3	2	4	3	3	7	1	0	1	1	0	1
2	2	3	2					1					4					0				
2	2	3	1	1	0	3		1	1	1	2		3	3	7			1	1	0		

2	2	2	1	3	2			1	2	1			5	3	2			0	1	1		
1	2	2	0	1	0	0		1	1	1	1		3	6	7	8		1	0	0	0	
2	3	3	3	3	2			2	2	1			4	3	1	4		0	1	1	0	
2	2	3	0	1	0	1		1	1	1	1		3	4	7			1	0	0		
2	2	3	2	1	1	2	1	1	1	1	1	1	4	4	6	7	4	0	0	0	0	0
2	2	3	2		3			1		2			4		2			0		1		
2	2	3	0	1	0	0		1	1	1	1		3	5	3	4		1	0	1	0	
2	2	3	0	0	1	3		1	1	1	2		4	5	1	5		0	0	1	0	
1	2	1	1	0	1	4	1	1	1	1	3	1	3	3	2	2	1	1	1	1	1	1
2	2	3	3	4	1	5	3	2	3	1	3	2	3	2	6	8	4	1	1	0	0	0
2	3	3	0	3	0	0	0	1	2	1	1	1	2	7	7	9	5	1	0	0	0	0
2	3	3	1	0	2	1		1	1	1	1		3	7	7	9		1	0	0	0	
3	2	4	1		1	1		1		1	1		3		4	2		1		0	1	
1	2	1	1	1	0	3	0	1	1	1	2	1	3	3	5	5	3	1	1	0	0	0
2	2	1	0					1					4					0				
1	2	3	4	1	4			3	1	3			3	7	5			1	0	0		
2	3	3	0	1	1	3		1	1	1	2		4	6	7	4		0	0	0	0	
2	2	3	0		0			1		1			4		7	9		0		0	0	
1	2		0	0	0	2		1	1	1	1		3	4	6			1	0	0		
2	2	3	2	1	4	6	6	1	1	3	3	3	3	3	2		1	1	1	1		1
2	2	3	1	0	0	0	2	1	1	1	1	1	4	3	6	2	4	0	1	0	1	0
2	2	2	3	0	2			2	1	1			4	4	3			0	0	1		
2	2	2	4					3					2					1				
1	2	1	0	2	4			1	1	3			3	5	6			1	0	0		
2	3	3	0					1					4					0				
2	2	3	2	1	0	2	0	1	1	1	1	1	3	4	3	3	1	1	0	1	1	1
2	2	3	1	1	0	0	0	1	1	1	1	1	5	6	6	9	5	0	0	0	0	0
2	2	3	0	0	1	2	0	1	1	1	1	1	5	7	7	8	4	0	0	0	0	0
2	2	2	3					2					3					1				
2	2	3	0					1					5					0				
1	2	2	3		1		1	2		1		1	4		7	8	5	0		0	0	0
1	1	2	0		3			1		2					7					0		
2	2	3	0	2	2	6		1	1	1	3		4	5	5	6	1	0	0	0	0	1
2	2	3	1	1	1	2	3	1	1	1	1	2	4	2	7	9	4	0	1	0	0	0
2	2	3	2		0			1		1			4		6			0		0		
1	2	3	4		3			3		2			2		2	7		1		1	0	
2	1	3	0	0	3	2	3	1	1	2	1	2	5	5	2	3	3	0	0	1	1	0
2	2	3	1	2	2	1		1	1	1	1		4	6	7	8		0	0	0	0	
2	2	3	0					1					4					0				
1	2	3	3	2	1	1	0	2	1	1	1	1	4	4	2	4	6	0	0	1	0	0
2	1	3	1					1					4					0				

2	2	3	3	1	3			2	1	2			3	5	4			1	0	0		
2	3	3	0	1	1			1	1	1			7	7	7			0	0	0		
1	1	3	2	2	0	2		1	1	1	1		4	5	5			0	0	0		
2	3	3	0	1	2	0	0	1	1	1	1	1	6	7	6		2	0	0	0		1
2	1	1	1	4	1			1	3	1			2	4	5			1	0	0		
2	2	3	2	1	3	3		1	1	2	2		5	4	6	6		0	0	0	0	
2	2	3	0	0	2			1	1	1			5	5	1			0	0	1		
2	2	2	5	1	2	4		3	1	1	3		4	6	3	1		0	0	1	1	
2	1	3	0	1	0			1	1	1			4	4	6			0	0	0		
2	2	3	1	1		1	2	1	1		1	1	5	4		6	6	0	0		0	0
2	2	3	0	0	0	4		1	1	1	3		6	5	7			0	0	0		
2	1	3	1	2	0	1	1	1	1	1	1	1	3	6	7	3	1	1	0	0	1	1
2	1	1	5					3					3					1				
2	2	3	0	1	0	1		1	1	1	1		6	4	7	8	6	0	0	0	0	0
2	2	4	2	4		4		1	3		3		4	3		2		0	1		1	
2	2	3	0	1	0	1	1	1	1	1	1	1	6	6	7	8	5	0	0	0	0	0
2	2	1	0	1	3	5		1	1	2	3		5	3	7			0	1	0		
2	2	3	0	1	2	1		1	1	1	1		4	5	4	5		0	0	0	0	
2	2	3	0		1	3	4	1		1	2	3	3		7	8	1	1		0	0	1
2	2	3	0	0	0	1	1	1	1	1	1	1	3	4	6	8	3	1	0	0	0	0
2	2	3	1	5	5	2	0	1	3	3	1	1	4	4	4	7	5	0	0	0	0	0
2	2	3	1	3	2		0	1	2	1		1	5	7	5		2	0	0	0		1
2	1	3	1	2	1	5		1	1	1	3		3	4	4			1	0	0		
1	1	1	2	4	5		1	1	3	3		1	6	4	4	8	4	0	0	0	0	0
2	1	3	2					1					3					1				
2	2	3	1	1	1	5		1	1	1	3		4	5	7	8	6	0	0	0	0	0
2	2	3	0	2	0	0	2	1	1	1	1	1	5	4	5	3	3	0	0	0	1	0
2	1	2	1	2	4	1		1	1	3	1		5	5	4	4		0	0	0	0	
2	2	3	2					1					6					0				
1	2	3	2					1					3					1				
2	2	3	0	0	1	0	0	1	1	1	1	1	4	4	7	7	3	0	0	0	0	0
2	1	3	0	0	0	3	0	1	1	1	2	1	3	5	6	6	1	1	0	0	0	1
2	2	3	2	2	4	3	0	1	1	3	2	1	3	4	7	5	6	1	0	0	0	0
1	1	2	2	4	4	4	2	1	3	3	3	1	4	2	2	7	3	0	1	1	0	0
1	2	3	1	3	1	2		1	2	1	1		4	2	3	2		0	1	1	1	
2	2	3	1	1	1	2		1	1	1	1		3	2	2	1		1	1	1	1	
2	2	3	0					1					5					0				
2	2	2	0	3	0	2	0	1	2	1	1	1	4	3	7	5	5	0	1	0	0	0
2	2	2	0	2	4			1	1	3			3	5	3			1	0	1		
1	2	3	2	1	0	2		1	1	1	1		3	7	7	1		1	0	0	1	
2	2	3	2	2			1	1	1			1	4	3		6	7	0	1		0	0

1	2	1	0	1	2	3		1	1	1	2		2	3	5	8		1	1	0	0	
1	1	3	2	6	7	3	0	1	3	3	2	1	5	2	5	7	6	0	1	0	0	0
2	2	3	0	1	0	2	0	1	1	1	1	1	4	5	7	9	6	0	0	0	0	0
2	2	3	2	1	4		1	1	1	3		1	4	3	4	4	1	0	1	0	0	1
1	1	2	1					1					4					0				
2	2	3	4	4	1	0		3	3	1	1		4	7	5	1		0	0	0	1	
2	1		0					1					3					1				
2	3	3	1	1	1	1	1	1	1	1	1	1	4	3	6	8	5	0	1	0	0	0
2	2	3	4					3					3					1				
2	2	3	0	1	1	1		1	1	1	1		5	6	4			0	0	0		
2	2	3	3	1	2	4		2	1	1	3		3	5	5	4		1	0	0	0	
2	1	3	2		2		6	1		1		3	4		3	4	1	0		1	0	1
2	3	3	1	1	0	1		1	1	1	1		5	5	7	8	7	0	0	0	0	0
1	3	1	2	1	1			1	1	1			3	5	3			1	0	1		
2	2	3	2	2	2	3	4	1	1	1	2	3	4	4	7	7	5	0	0	0	0	0
2	2	2	3					2					2					1				
2	2	3	0	0		1	1	1	1		1	1	5	6		9	6	0	0		0	0
1	3	3	2	5	2	7	3	1	3	1	3	2	3	4	7		3	1	0	0		0
2	1	3	0		2	4		1		1	3		3		4			1		0		
2	2	3	1					1														
1	2	3	2					1					3					1				
2	2	3	0	1	2	1	0	1	1	1	1	1	4	4	7	8	6	0	0	0	0	0
2	3	3	2	2	0	2	0	1	1	1	1	1	4	5	7	7	3	0	0	0	0	0
2	3	3	0	1	0	2	0	1	1	1	1	1	4	4	6	4	1	0	0	0	0	1
3	3	3	0	3	3	3		1	2	2	2		6	7	7	8		0	0	0	0	
1	2	3	3	2	3	3	0	2	1	2	2	1	7	4	5		6	0	0	0		0
1	1	3	0	2	0			1	1	1			3	3	5	4		1	1	0	0	
2	2	3	0	2	1			1	1	1			3	5	6	2		1	0	0	1	
1	2	3	1	0	1	3	1	1	1	1	2	1		4	5	5	6		0	0	0	0
2	1	3	1	3	6	2	0	1	2	3	1	1	4	5	6	7	2	0	0	0	0	1
1	2	3	2	3	3	2		1	2	2	1		3	3	6	3		1	1	0	1	
1	2	1	1		2			1		1					7					0		
1	1	1	0					1					1					1				
1	2	2	1	1		2		1	1		1			2		2			1		1	
2	2	3	1	1	0		0	1	1	1		1	3	4	3		6	1	0	1		0
2	2	1	1	0				1	1				3	2				1	1			
2	2	4		4					3				2	5				1	0			
2	2	3	1	2	0	3	3	1	1	1	2	2	4	3	4	2	1	0	1	0	1	1
1	2	2	1					1					7					0				
2	2	3	1	0	1	0	1	1	1	1	1	1	4	4	4	4	3	0	0	0	0	0
2	1	3	1	2	0	1	0	1	1	1	1	1	4	4	5	5	7	0	0	0	0	0

2	3	3	2	1	3	0	0	1	1	2	1	1	6	7	7	8	7	0	0	0	0	0
2	2	3	0	0	0	0	0	1	1	1	1	1	4	5	7	3	7	0	0	0	1	0
2	2	3	0					1					5					0				
2	2	3	2					1					3					1				
1	2	1	2					1														
1	1	3	0	0	0	2	3	1	1	1	1	2	4	7		8	3	0	0		0	0
2	1	2	1	1	1	6		1	1	1	3		3	7	2			1	0	1		
2	2	1	4					3					2					1				
2	2		2	2	1	4	0	1	1	1	3	1	4	6	4	9	4	0	0	0	0	0
1	3	3	3	4			3	2	3			2	3	3			3	1	1			0
2	2	3	0		1			1		1			4		5			0		0		
2	2	3	0	0	0	4		1	1	1	3		7	6	4	6		0	0	0	0	
2	2	3	0					1					3					1				
2	2	3	3					2					3					1				
1	2	2	5					3					4					0				
1	2	3	2	3	1	3		1	2	1	2		3	3	4	1		1	1	0	1	
2	2	2	0					1					4					0				
1	1	2	3					2					1					1				
1	2	2	3	3	3	2	3	2	2	2	1	2	2	5	3	2	3	1	0	1	1	0
2	1	3	0	1	5	1		1	1	3	1		3	4	2	4		1	0	1	0	
1	2	1	3					2					3					1				
2	2	3	2	1	0	1		1	1	1	1		3	5	7	8		1	0	0	0	
1	2	2	0					1					3					1				
2	3	2	0			3		1			2		4			4		0			0	
2	2	3	0		1	0		1		1	1		3		5	5		1		0	0	
2	2	3	1	1	1	7		1	1	1	3		4	4	6	5		0	0	0	0	
2	2	3	1	1	0	0	1	1	1	1	1	1			7	1	3			0	1	0
1	2	3	2		1	1	0	1		1	1	1	3		7	8	7	1		0	0	0
2	1	3	0	0	4	2	0	1	1	3	1	1	4	5	3	1	3	0	0	1	1	0
2	2	3	1	0	0	1		1	1	1	1		4	4	5	4		0	0	0	0	
2	1	3	0	0	0	1	2	1	1	1	1	1	5	6	7	5	3	0	0	0	0	0
2	1	3	5	4	1	0	2	3	3	1	1	1	2	7	5	6	4	1	0	0	0	0
2	1	3	0	2	3	1	4	1	1	2	1	3	3	4	5	6	5	1	0	0	0	0
3	2	2	0	0		3		1	1		2			2		3			1		1	
2	2	3	3	2				2	1				4	4				0	0			
1	2	2	2					1					3					1				
1	2	2	2					1					4					0				
1	1	3	4					3					2					1				
3	2	3	0	0	0	2		1	1	1	1		4	4	4	5		0	0	0	0	
2	2	3		2	0				1	1			6	4	4			0	0	0		
1	2	3	5					3					3					1				

2	2	3	0	1	1	2	1	1	1	1	1	1	3	6	7	9	4	1	0	0	0	0
1	2	1	1					1					3					1				
3		3	1	1	0	1	1	1	1	1	1	1	4	6	6	8	5	0	0	0	0	0
1	3	3	0	0	0			1	1	1			4	4	6	8		0	0	0	0	
2	3	3	3	2	2	2		2	1	1	1		1	4	7	4		1	0	0	0	
2	2	3	1	1	2	3		1	1	1	2		5	6	6	6		0	0	0	0	
2	2	3	0	0				1	1				3	7				1	0			
2	2	3	2					1					4					0				
2	3	3	2		0	1		1		1	1				5					0		
2	1	3	2	3	4	5	0	1	2	3	3	1	3	4	3	2	6	1	0	1	1	0
2	1	1	4		4			3		3			3		3			1		1		
2	2	3	1	1	1	2	1	1	1	1	1	1	4	6	2	1	2	0	0	1	1	1
2	2	2	2	2	1	3		1	1	1	2		4	4	2	3		0	0	1	1	
2	3	3	0	1	1	1	0	1	1	1	1	1	4	5	7	8	7	0	0	0	0	0
2	2	3	2	6	0			1	3	1			4	7	7	8		0	0	0	0	
1	2	3	1	1	2	1		1	1	1	1		5	3	2	5		0	1	1	0	
1	2	1	0	1	0	0	1	1	1	1	1	1	4	4	4		6	0	0	0		0
3	1	4	3	1	0	4	1	2	1	1	3	1		3	4	1	4		1	0	1	0
3	3	3	0	1	0	2		1	1	1	1		4	5	7	7		0	0	0	0	
1	1	3	2					1					3					1				
2	2	3	2	1	3	1	3	1	1	2	1	2	3	6	4	3	1	1	0	0	1	1
2	2	3	0	0	2	2		1	1	1	1		4	6	3	2		0	0	1	1	
3	3	2	2					1					3					1				
2	2	3	0	0	1			1	1	1			4	5	7			0	0	0		
2	1	3	1		5	1	3	1		3	1	2	4		5	4	3	0		0	0	0
2	3	3	0	1	1	0	1	1	1	1	1	1	3	5	5	8	4	1	0	0	0	0
1	2	1	2	4	2			1	3	1			5	2	5			0	1	0		
2	2	3	0			1		1			1		5			4		0			0	
1	2	3	3	3	2	5		2	2	1	3			4	5	3			0	0	1	
2	3	3	0	1	2			1	1	1			4	5	7			0	0	0		
2	2	3	1	0	0			1	1	1			4	4	6			0	0	0		
3	1	3	1	0	0	1	0	1	1	1	1	1	3	3	3	3	4	1	1	1	1	0
1	2	1	1	0	0			1	1	1			4	3	4			0	1	0		
2	3	3	2	1	1			1	1	1			3	5	2			1	0	1		
2	2	3	1	1	5	2		1	1	3	1		4	4	6	1		0	0	0	1	
2	3	3	3		1			2		1			4		7			0		0		
2	1	3	3	5	4	2		2	3	3	1		4	3	7	8		0	1	0	0	
2	3	3	1	4	1			1	3	1			3	6	7			1	0	0		
2	2	2	0	2	0			1	1	1			4	4	2			0	0	1		
2	2	3	0					1					3					1				
2	2	3	0		0	1	1	1		1	1	1	3		7	8	5	1		0	0	0

1	2	3	2	4	2	1		1	3	1	1		3	4	3	3		1	0	1	1	
2	1	2	0		6			1		3			6		4			0		0		
2	2		2	1	1	2	1	1	1	1	1	1	3	5	5	1	5	1	0	0	1	0
2	2	3	0					1														
2	2	2	3					2					2					1				
2	2	3	1					1					6					0				
2	1	3	1	5	6			1	3	3			4	4	3			0	0	1		
2	2	3	0					1					4					0				
1	2	2	4					3					3					1				
2	2	3	1	1	0	2	0	1	1	1	1	1	3	5	7		7	1	0	0		0
2	2	2	0					1					5					0				
2	2	3	5					3					1					1				
2	3	3	1		1	1	0	1		1	1	1	3		4	8	3	1		0	0	0
2	2	3	2	2	2	1		1	1	1	1		3	2	4	4		1	1	0	0	
2	3	1	5		1	1	2	3		1	1	1	2		5	9	5	1		0	0	0
2	2	3	1	0	0	1	0	1	1	1	1	1	4	6	7	4	1	0	0	0	0	1
2	2	1	0	1	0	4	2	1	1	1	3	1	4	2	5	6	1	0	1	0	0	1
2	2	3	0	3	1	1		1	2	1	1		3	3	2	1		1	1	1	1	
2	3	3	0	2		0		1	1		1		5	5		6		0	0		0	
1	2	1	0	1	0	3	1	1	1	1	2	1	5	6	7	8	1	0	0	0	0	1
2	2	3	2	1	4	1	2	1	1	3	1	1	3	7	5	5	1	1	0	0	0	1
1	2	3	0	3	0	2		1	2	1	1		4	3	5	6		0	1	0	0	
2	2	3	1		6	4		1		3	3		4		4	2		0		0	1	
1	2	2	0	1	0	0	0	1	1	1	1	1	3	5	7	8	4	1	0	0	0	0
2	3	3	0	1	0	3	2	1	1	1	2	1	7	4	7	9	3	0	0	0	0	0
1	2	2	0	2	0			1	1	1			6	3	7		4	0	1	0		0
2	2	3	0	2	1			1	1	1			3	4	5			1	0	0		
2	3	3	0	1	0	0	0	1	1	1	1	1	4	6	7	8	7	0	0	0	0	0
3	2	3	0	0	0	0	0	1	1	1	1	1	4	5	7	6	5	0	0	0	0	0
2	2	3	4	2	3	1	1	3	1	2	1	1	3	5	4	5	3	1	0	0	0	0
2	2	3	1	0	0	1		1	1	1	1		4		7	8		0		0	0	
2	2	3	1	1	0	0	0	1	1	1	1	1	5	5	5	9	2	0	0	0	0	1
2	2	2	1	0	2			1	1	1			3	4	3			1	0	1		
2	1	2	3					2					4					0				
2	1	2	1	1	1	5		1	1	1	3		3	2	2	2		1	1	1	1	
2	2	3	3	4	3			2	3	2				2	4				1	0		
2	1	3	0	3	4	3	3	1	2	3	2	2	4	3	2	4	5	0	1	1	0	0
2	2	3	2	3	1	2	3	1	2	1	1	2	4	2	3	5	1	0	1	1	0	1
2	2	3	0	1	1	1	2	1	1	1	1	1	3	5		2	3	1	0		1	0
2	3	3	0	2	0			1	1	1			4	7	7			0	0	0		
2	1	3	0	1				1	1				3	3				1	1			

2	2	3	0					1														
2	2	3	2	1	0	2	0	1	1	1	1	1	3	5	6	1	5	1	0	0	1	0
2	2	3		2	0	2			1	1	1		3	5	5	5		1	0	0	0	
2	2	2	3	4				2	3				3	3				1	1			
1	2	2	3		5	1	4	2		3	1	3	3		3	2	6	1		1	1	0
2	3	3	0	1	2			1	1	1			4	7	7			0	0	0		
2	2	3	3	3	2	3		2	2	1	2			3	4				1	0		
3	3	3	0	2	0	0	0	1	1	1	1	1	4	6	7	8	6	0	0	0	0	0
2	1	3	0	1	3	3	2	1	1	2	2	1	3	3	5	6	3	1	1	0	0	0
2	2	3	1	2	1	3		1	1	1	2		6	5	6	5		0	0	0	0	
1	2	2	1	3	2	0		1	2	1	1		3	4	6	8		1	0	0	0	
2	3	3	0	1	0			1	1	1			3	2	4			1	1	0		
2	3	3	2	1	1			1	1	1			4	3	6			0	1	0		
2	2	3	1	1	0	3		1	1	1	2		6	6	5	7		0	0	0	0	
2	2	3	0	1				1	1				3	2				1	1			
1	1	2	2	3	4	5	5	1	2	3	3	3	3	4	6	8	4	1	0	0	0	0
2	2	3	0	0	1			1	1	1			4	6	4	6		0	0	0	0	
2	2	1	4			2	2	3			1	1	1			1	1	1			1	1
2	2	2	0					1					3					1				
2	2	3	0	0	0	0	1	1	1	1	1	1	5	7	7	9	4	0	0	0	0	0
1	2	3	0					1					4					0				
1	2	3	0	1	1	4		1	1	1	3		3	2	4			1	1	0		
1	2	2	0					1					3					1				
2	2	4	1					1					5					0				
2	2	3	0					1					3					1				
1	1	2	3	2	2			2	1	1			2	3	5	1		1	1	0	1	
2	2	3	0	2	4	0		1	1	3	1		4	6	7		5	0	0	0		0
2	2	3	1	0	0	1		1	1	1	1		4	4	4	2		0	0	0	1	
2	2	3	2	1	2	5		1	1	1	3		4	5	5	5		0	0	0	0	
2	2	4	2	1	1	3		1	1	1	2		4	7	5	2		0	0	0	1	
1	2	3	1	0	1			1	1	1			3	3	5			1	1	0		
2	2	3	1	2	0			1	1	1			4	4	5			0	0	0		
2	1	2	0	0	0			1	1	1			4	6	5			0	0	0		
2	1	3	6					3					3					1				
2	2	3	2	2	1	1		1	1	1	1		4	4	6	7		0	0	0	0	
2	2	3	1	0		0	0	1	1		1	1	5	3			4	0	1			0
2	2	3	2	3	4			1	2	3			3	3	1	1		1	1	1	1	
2	2	3	1	3				1	2				3	4		2		1	0		1	
2	2	3	0	1	2			1	1	1			3	3	2			1	1	1		
2	2	2	1					1					4					0				
2	2	3	0	0	3	4	0	1	1	2	3	1	4		3	5	4	0		1	0	0

2	2	3	0	1	0	0		1	1	1	1		5	6	6	6		0	0	0	0	
2	3	3	2	1	0			1	1	1			5	5	7			0	0	0		
2	1	3	1	0	0			1	1	1			4	5	7			0	0	0		
2	1	3	0	1	1	2	3	1	1	1	1	2	3	4	5		3	1	0	0		0
2	1	2	1					1					5					0				
2	2	3	0	1	0	2		1	1	1	1		4	6	4	4		0	0	0	0	
2	2	3	0	1	0	3	0	1	1	1	2	1	4	6	6		4	0	0	0		0
1	2	1	1	0				1	1					7					0			
2	2	3	2	3	4	3	2	1	2	3	2	1	4	4	1	1	5	0	0	1	1	0
2	3	3	0	0	0	0	0	1	1	1	1	1	6	5	7	9	6	0	0	0	0	0
2	3	3	1	3	1	1		1	2	1	1		4	5	4	4		0	0	0	0	
2	2	3	1	5	0	2	0	1	3	1	1	1					3					0
1	3	3	2	1	0	1		1	1	1	1		3	4	7	7		1	0	0	0	
2	2	3	3	2	0			2	1	1			4	5	7		1	0	0	0		1
2	1	3	1	0				1	1				3	2				1	1			
2	2	1	1			1		1			1		3					1				
2	2	3	0	0	1	0		1	1	1	1			3	7	5			1	0	0	
1	1	3	0	1				1	1				4					0				
3	3	3	2	0	4	2	3	1	1	3	1	2	4	6	5	2	4	0	0	0	1	0
2	1	3	0	0	1	3		1	1	1	2		4	4	6	1		0	0	0	1	
1	1	1	4	2	1	4	0	3	1	1	3	1		6	6		7		0	0		0
2	2	3	0	0	0	1		1	1	1	1		4	4	7	7		0	0	0	0	
1	2	2	2					1														
2	1	2	0	0	2			1	1	1			4	4	1			0	0	1		
2	3	3	1					1					4			6		0			0	
1	2	3	0	0	1	4	3	1	1	1	3	2	3	4	5	3	3	1	0	0	1	0
2	1	3	0	1	0	0	1	1	1	1	1	1	3	4	3	2	1	1	0	1	1	1
1	3	3	2	0		2		1	1		1		3	3		1	3	1	1		1	0
3	1	3	1	1	2	0		1	1	1	1		3	5	6	1		1	0	0	1	
2	1	3	0	0	0			1	1	1			5	5	5			0	0	0		
2	2	3	3	3	1	1		2	2	1	1		4	2	1	9		0	1	1	0	
2	2	3	3					2					3					1				
2	2	4	6					3					3					1				
2	2	3	6	1	0	0		3	1	1	1		3	5	5	7		1	0	0	0	
2	3	3	1	3	1	1	1	1	2	1	1	1	5	2	7	9	6	0	1	0	0	0
2	2	3	0	2	1	3		1	1	1	2		4	2	4	2		0	1	0	1	
2	3	3	2		1	0		1		1	1		5		7			0		0		
2	3	3	0					1					1					1				
2	2	3	2			1		1			1		3			2		1			1	
1	2	2	4	3	4	3		3	2	3	2			7	7				0	0		
3	2	3	0	0	1	0		1	1	1	1		3	6	7	1	4	1	0	0	1	0

2	2	3	0	1	1	4	1	1	1	1	3	1	4	5	6	2	7	0	0	0	1	0
1	2	2	1					1														
2	2	3	4					3					4					0				
1	2	1	3					2					3					1				
2	3	3	3	1	1	1		2	1	1	1		5	6	6	9		0	0	0	0	
2	1	3	2	5	2			1	3	1			5	3	5			0	1	0		
2	2	3	1	1	3	2	3	1	1	2	1	2	4	4	7	4	1	0	0	0	0	1
2	2	3	2		5	2		1		3	1		3		6			1		0		
3	2	3	2	2	3	4	7	1	1	2	3	3	3	3	1	1	3	1	1	1	1	0
2	2	3	1	0	2	2	0	1	1	1	1	1	5	6	5	8	6	0	0	0	0	0
2	3	3	0					1					4					0				
2	2	3	3	1	0			2	1	1			5	7	7			0	0	0		
1	2	3	5	4	1	1	1	3	3	1	1	1	6	6	7	1	4	0	0	0	1	0
2	2	3	0	2	0	2	1	1	1	1	1	1	4	4	7	9	3	0	0	0	0	0
1	2	1	0	3	4	0		1	2	3	1		1	5	7	8		1	0	0	0	
2	2	3	0	2	2	0		1	1	1	1		3	4	6			1	0	0		
2	2	3	1					1					5					0				
1	2	4	5	3	0	0	1	3	2	1	1	1	3	3	7	9	5	1	1	0	0	0
2	2	4	0	2	0	2		1	1	1	1		3	5	6			1	0	0		
3	2	3	2	3				1	2				4	4				0	0			
2	2	2	2		0	1		1		1	1		3		7	8		1		0	0	
1	2	3	0			3		1			2		3			7		1			0	
2	2	3	0	2	0	1	1	1	1	1	1	1	4	5	4	2	3	0	0	0	1	0
1	2	3	0					1					3					1				
2	2	1	1	1	2			1	1	1			5	2	7			0	1	0		
2	3	3	1	0	1	0		1	1	1	1		3	3	3	3		1	1	1	1	
2	2	3	1	3	1	0		1	2	1	1		4	4	7	7		0	0	0	0	
2	2	3	2			2		1			1		5					0				
2	2	3	0	1	0			1	1	1			4	4				0	0			
2	2	3	3	3				2	2				4	2				0	1			
2	3	3	2	1	0	2	0	1	1	1	1	1	4	5	7	8	5	0	0	0	0	0
2	2	3	1	0	0	6		1	1	1	3		4	6	6	3		0	0	0	1	
3	2	3	0					1					5					0				
1	2	2	2					1					3					1				
2	1	3	0	1	0	0	0	1	1	1	1	1	5	4	6	5	1	0	0	0	0	1
2	3	3	1	2	4			1	1	3			4	4				0	0			
2	1	3	1	1				1	1				4	3				0	1			
2	1	2	2	1	1	7	5	1	1	1	3	3	2	4	4	4	1	1	0	0	0	1
2	3	3	0		2	6	2	1		1	3	1	4		4		1	0		0		1
2	2	3	0	1	3	4	1	1	1	2	3	1	4	4	3	7	3	0	0	1	0	0
1	2	2	1	0	0	2		1	1	1	1		4	4	5	9		0	0	0	0	

1	2	1	2	2	4	4	0	1	1	3	3	1	2	6	7	8	5	1	0	0	0	0
2	2	3	1	0	0	3		1	1	1	2		3	4	4			1	0	0		
2	3	2	2	2	1	1	3	1	1	1	1	2	2	2	3	3	5	1	1	1	1	0
2	1	3	0	3	1	1	0	1	2	1	1	1	4		3	2	6	0		1	1	0
2	3	3	1	2				1	1				3	3				1	1			
1	2	1	6					3					7					0				
1	3	2	6	5	4	1	2	3	3	3	1	1	3	2	7	6	4	1	1	0	0	0
2	3	1	1	2	2	4	3	1	1	1	3	2	7	4	5	4	3	0	0	0	0	0
2	1	3	1	1	2			1	1	1			3	3	1	1		1	1	1	1	
2	3	3	0	0	0			1	1	1			3	4	6			1	0	0		
1	2	3	0		2	4	0	1		1	3	1	3		5		6	1		0		0
2	2	3	1	1	2	1		1	1	1	1		4	5	7	8		0	0	0	0	
1	2	3	1	1	1	0		1	1	1	1		3	5	7	3	1	1	0	0	1	1
2	2	3	1	1	1	1	3	1	1	1	1	2	3	4	6	6	4	1	0	0	0	0
2	1	3		1					1				4	4				0	0			
1	1	1	2		1		0	1		1		1	2				4	1				0
3	2	3	1	2	1	3	1	1	1	1	2	1	4	5	7	1	7	0	0	0	1	0
1	2	1	2		1	4		1		1	3		3		5			1		0		
2	2	3	0	2	4	0	0	1	1	3	1	1	3	3	5	6	7	1	1	0	0	0
2	3	3	0	1	0	1		1	1	1	1		5	5	7	1		0	0	0	1	
2	2		2	1		3	2	1	1		2	1	3	3		1	2	1	1		1	1
2	2	3	0	2	1	0	0	1	1	1	1	1	4	4	5	6	7	0	0	0	0	0
2	2	2	1					1					2					1				
2	1	3	1	1	2	5	2	1	1	1	3	1	3	4	2	1	1	1	0	1	1	1
2	3	2	1					1														
2	3	3	0	0	0	0		1	1	1	1		5	6	7	9		0	0	0	0	
2	2	3	4	3	4	2	3	3	2	3	1	2	3	3	2	1	5	1	1	1	1	0
2	2	1	4	1	2	1		3	1	1	1		3	3	4	6		1	1	0	0	
1	2	4	1	5	2	1		1	3	1	1		4	4	6	5		0	0	0	0	
2	2	3	1	0	1		1	1	1	1		1	4	7	7		3	0	0	0		0
2	2	3	0	0	1			1	1	1			4	5	6	6		0	0	0	0	
2	2	3	1	2	1			1	1	1			5	7				0	0			
1	2	3	1	3	3	8	4	1	2	2	3	3	4	3	5	4	1	0	1	0	0	1
2	2	1	2		5	2	2	1		3	1	1	2		3	1	4	1		1	1	0
1	2	3	0	2	0			1	1	1				3					1			
1	2	3	1					1					4					0				
2		1	0					1					4					0				
2	2	1	2	0	1	2	3	1	1	1	1	2	2	5	7		4	1	0	0		0
2	2	3	2	0	6	1		1	1	3	1		4	3	1	1		0	1	1	1	
2	2	3	2	3				1	2				6	3				0	1			
2	1	3	0	1	0	2		1	1	1	1			4	7	8			0	0	0	

2	1	3	0	3	1	3		1	2	1	2		4	4	7	7		0	0	0	0	
2	2	3	0	0	0	1		1	1	1	1		4	3	5	6		0	1	0	0	
2	2	2	1	3	1	4		1	2	1	3		4	6	6			0	0	0		
2	3	2	2	1	0	3	0	1	1	1	2	1	3	7	7	7	1	1	0	0	0	1
2	2	3	0	1	0	2	4	1	1	1	1	3	4	7	7		5	0	0	0		0
2	2	3	2		3	1	0	1		2	1	1	6		6		7	0		0		0
2	2	3	1	5	5			1	3	3				2	5				1	0		
1	2	1	0	1	1			1	1	1			2	3				1	1			
2	2	3	0	1	0	1	3	1	1	1	1	2	4	4	6	7	7	0	0	0	0	0
2	3	3	0					1					4					0				
2	2	3	1	1	0	0	0	1	1	1	1	1	5	7	7	9	2	0	0	0	0	1
2	2	3	1	1	0	0		1	1	1	1		5	4	5			0	0	0		
2	2	3	1	1	1	3	3	1	1	1	2	2	2	2	3	3	1	1	1	1	1	1
1	2	2	1	2	2	3		1	1	1	2		3	2	3			1	1	1		
2	2	3	0	2	2	4		1	1	1	3		4	4	3	1		0	0	1	1	
2	3	3	0	1	0	0	0	1	1	1	1	1	3	4	4	4	3	1	0	0	0	0
2	2	3	0	1	0			1	1	1			5	5	7			0	0	0		
2	2	1	2					1					1					1				
1	1		3	4				2	3				2					1				
2	3	3	0	0	2	0	1	1	1	1	1	1	5	7	7	9	7	0	0	0	0	0
3	2	3	1	1	0	4	1	1	1	1	3	1	5	7	6	3	5	0	0	0	1	0
2	2	3	2	4	6	2		1	3	3	1		4	7	6	7		0	0	0	0	
2	2	3	0	1	0			1	1	1			4	4	7			0	0	0		
2	3	3	5	2	1	4		3	1	1	3		3	3	7			1	1	0		
2	2	3	1	4	2			1	3	1			3	2	5			1	1	0		
2	3	4	1					1					6					0				
2	1	3	1	0	3			1	1	2			3	6	7	2		1	0	0	1	
2	2	3	0	0	0	1	1	1	1	1	1	1	4	5	7	6	7	0	0	0	0	0
1	1	3	2	5	5	6	6	1	3	3	3	3	4	3	5	7	1	0	1	0	0	1
2	1	3	2	1	4	2	4	1	1	3	1	3	4	7	6	2	4	0	0	0	1	0
1	2	3	1					1					4					0				
2	1	3	0	2	1			1	1	1			4	5	7			0	0	0		
2	3	3	1	1	1			1	1	1			3	6	5	7		1	0	0	0	
2	2	3	0	4	1			1	3	1			4	3	7			0	1	0		
1	2	3	0	0				1	1				3	4				1	0			
2	1	1	4					3					1					1				
2	3	3	2	1	1	2	1	1	1	1	1	1	4	4	7	7	5	0	0	0	0	0
2	3	3	1	1	0	2	0	1	1	1	1	1	4	4	7	8	1	0	0	0	0	1
2	2	3	0					1					3					1				
2	1	3	1	1	0	4	5	1	1	1	3	3	4	6	7	3	2	0	0	0	1	1
1	2	3	3	1	0	4	2	2	1	1	3	1	4	2	3		5	0	1	1		0

1	2	3	1					1					4					0				
1	2	3	0	1	0	1		1	1	1	1		3	3	7	7		1	1	0	0	
1	2	3	4	3	5			3	2	3			3	3	7			1	1	0		
2	2	3	3	7	3	2	1	2	3	2	1	1		3	7	7	5		1	0	0	0
1	2	2	3	1	3	6		2	1	2	3		4	3	4	3		0	1	0	1	
2	2	3	0	1	0			1	1	1			4	2	4			0	1	0		
2	2	1	0	0				1	1				3	4				1	0			
2	3	3	1	4	0	3	2	1	3	1	2	1	6	7	7	9	6	0	0	0	0	0
2	1	3	1	2	3	2		1	1	2	1		4	5	3	1		0	0	1	1	
2	3	3	1		0			1		1			4		7			0		0		
2	2	1	2					1					4					0				
2	2	3	2	0				1	1					7					0			
2	3	3	0	2				1	1				4	6		9		0	0		0	
2	2	2	0		1	2	0	1		1	1	1	3		5	6	7	1		0	0	0
2	2	3	1	1	1			1	1	1			4	4	7			0	0	0		
2	2	3	2	0	0	0	0	1	1	1	1	1	5	6	7	9	5	0	0	0	0	0
1	3	3	2	5	2			1	3	1			3	3	7			1	1	0		
2	2	3	1		1	1		1		1	1		5		7	9		0		0	0	
2	2	3	0					1					3					1				
2	2	2	3					2					3					1				
2	1	3	0	0				1	1				3	2				1	1			
2	1	3	0	1	0			1	1	1			4	4	5			0	0	0		
2	2	3	0		3	2		1		2	1		3		7			1		0		
2	2	3	0	1	0	0	0	1	1	1	1	1	3	4	6	8	2	1	0	0	0	1
1	3	3	2	1	0	0	1	1	1	1	1	1	4	5	7	8	4	0	0	0	0	0
2	3	3	0	2	2	2	4	1	1	1	1	3	5	4	4	3	5	0	0	0	1	0
3	2	3	0	3	0	1	1	1	2	1	1	1	4	5	6	1	3	0	0	0	1	0
2	2			1	3	1			1	2	1		4	6	3	2		0	0	1	1	
2	3	3	2					1					4					0				
2	3	1	5	3	1	2	4	3	2	1	1	3	3	7	6	7	4	1	0	0	0	0
1	2	1	0					1					2					1				
2	2	4	3	0				2	1				3	2				1	1			
2	3	1	1		3			1		2			4		4			0		0		
3	3	4	2					1					2					1				
2	2	3	5			2	0	3			1	1	4			9	7	0			0	0
2	2	3	0	1	0	1	1	1	1	1	1	1	4	4	5	5	4	0	0	0	0	0
2	2	4	2	1	2	5		1	1	1	3		3	2	1	1		1	1	1	1	
2	2	3	3	5	4			2	3	3			3	4	3	3		1	0	1	1	
2	2	3	0	1	2		3	1	1	1		2	3	7	7	9	2	1	0	0	0	1
2	3	3	0	1				1	1				4	4				0	0			
2	2	3	1		0	3	0	1		1	2	1	4		4	3	5	0		0	1	0

2	2	3	1					1					4					0				
1	2	3	3	1		3	2	2	1		2	1	3	5			1	1	0			1
2	2	3	0	1	1	3		1	1	1	2		4	6	7	8		0	0	0	0	
2	1	3	1	2	2			1	1	1			4	5	6			0	0	0		
3	3	3	1		0	0		1		1	1		4		1	1		0		1	1	
2	2	3	1	1	0	0	1	1	1	1	1	1	3	3	5	5	6	1	1	0	0	0
2	2	3	3	0				2	1				4	4				0	0			
1	2	3	1					1					4					0				
2	2	3	1	2				1	1				6	7				0	0			
2	2	3	1	1	0	0	2	1	1	1	1	1	1	5	7	8	4	1	0	0	0	0
2	3	2	1	1	1			1	1	1			3	4	5	8		1	0	0	0	
2	2	1	4	0	0	2		3	1	1	1		2	5	6	7		1	0	0	0	
1	2	3	1	1	2			1	1	1			3	3	5			1	1	0		
2	2	3	3					2					6					0				
2	2	3	1		1		2	1		1		1	5		7		6	0		0		0
2	2	3	0	1				1	1				5	7				0	0			
2	2	1	1					1														
2	3	3	0	0	1	0	0	1	1	1	1	1	4	5	4		6	0	0	0		0
2	2	3	1	2	2	0	1	1	1	1	1	1	3	3	5	8	5	1	1	0	0	0
1	2	3	0					1					3					1				
2	2	3	1	2				1	1				3	4				1	0			
2	2	3	2		2	3	1	1		1	2	1	4		6	8	3	0		0	0	0
2	2	1	2	1	1	2	4	1	1	1	1	3	4	4	5	5	6	0	0	0	0	0
2	2	4	6	5	4			3	3	3			4	3	3	1		0	1	1	1	
2	1	3	1		2	1	0	1		1	1	1	4		5	4	4	0		0	0	0
2	2	3	2	1	4			1	1	3			4	5	1			0	0	1		
2	3	3	1	1	0	1	0	1	1	1	1	1	7	7	5	7	4	0	0	0	0	0
2	1	3	1	2	1	1	0	1	1	1	1	1	6	5	7	9	4	0	0	0	0	0
2	3	3	5	1				3	1				3	4				1	0			
1	2	3	3	1	3		1	2	1	2		1	2	4	4	6	5	1	0	0	0	0
2	1	3	4					3					4					0				
2	3	3	1	1	0	1	2	1	1	1	1	1	7	6	7	8	7	0	0	0	0	0
2	3	3	0	1	0			1	1	1			3	3	6			1	1	0		
2	2	3	0	1	2	1	0	1	1	1	1	1	4	7	7	8	5	0	0	0	0	0
2	2	3	0					1					4					0				
2	2	2	5					3					3					1				
2	1	2	1	1	1			1	1	1			4	5				0	0			
2	1	3	1	1	5			1	1	3			3	3	4			1	1	0		
2	2	3	3		2	4	2	2		1	3	1	3		5	6	1	1		0	0	1
2	2	3	0	0	1	0		1	1	1	1		3	6	5	6		1	0	0	0	
2	2	3	2	1	1	0	2	1	1	1	1	1	3	7	7	8	5	1	0	0	0	0

2	2	3	0	1	0	0	1	1	1	1	1	1	3	4	7	6	1	1	0	0	0	1
2	2	3	2	1	3	2	0	1	1	2	1	1	4	4	6	6	4	0	0	0	0	0
2	2	3	1	0	3			1	1	2			3	3	3			1	1	1		
2	1	2	0	2	1	1	0	1	1	1	1	1	5	3	7	2	4	0	1	0	1	0
2	2	3	0	6	1	2	1	1	3	1	1	1	4	4	4	2	7	0	0	0	1	0
2	3	3	0					1					3					1				
1	2	2	4	4				3	3				4					0				
2	3	3	0	0	0	2	0	1	1	1	1	1	6	5	6	8	4	0	0	0	0	0
2	2	2	0	1	0	2		1	1	1	1		4	3	5			0	1	0		
2	3	3	1	2	2	2		1	1	1	1		6	7	7	7		0	0	0	0	
1	2	3	1					1					4					0				
2	3	3	2	0	1	1	0	1	1	1	1	1	4	6	5	4	5	0	0	0	0	0
2	2	3	2	0	1	1	1	1	1	1	1	1	3	5	6	7	5	1	0	0	0	0
2	2	3	0	0		1	0	1	1		1	1	4	5		6	2	0	0		0	1
2	2	3	2	1	0	1	0	1	1	1	1	1	3	3	6	5	1	1	1	0	0	1
1	1	3	3					2					4					0				
2	2	3	0	4	1	1	0	1	3	1	1	1	3	3	7	8	7	1	1	0	0	0
2	2	3	1	0	0	0	0	1	1	1	1	1	5	6	7	9	5	0	0	0	0	0
1	2	3	0	1	0	0	1	1	1	1	1	1	4	5	7	8	3	0	0	0	0	0
2	2	3	1	0	0	3		1	1	1	2		4	5	7	8		0	0	0	0	
3	2	3	2	3	1	0	3	1	2	1	1	2	4	3	5	8	1	0	1	0	0	1
2	2	3	2	1	2	2		1	1	1	1		4	4	4	2		0	0	0	1	
2	3	3	0	1	1	1	2	1	1	1	1	1	4	3	7	5	4	0	1	0	0	0
2	2	3	0	0	2	4		1	1	1	3		6	7	5			0	0	0		
2	2	3	2	2				1	1				3	3				1	1			
2	2	3	1					1					5					0				
2	3	3	1	1	0	3	4	1	1	1	2	3		5	7		1		0	0		1
1	2	3	2	2	0			1	1	1			4	4	6			0	0	0		
2	2	3	1	4	3			1	3	2			4	6	3			0	0	1		
2	2	3	1	0	1			1	1	1				3	5				1	0		
2	2	3	3	4	4			2	3	3			3	6	5			1	0	0		
2	2	3	1	2	2			1	1	1			4	2	4			0	1	0		
1	1	3	0	1	1	6	8	1	1	1	3	3	3	4	6	8	1	1	0	0	0	1
1		1	1	1	3	3		1	1	2	2			3	4	1			1	0	1	
2	3	3	1	2	0	1	0	1	1	1	1	1	4	3	4	6	3	0	1	0	0	0
2	3	3	2	0	1	3		1	1	1	2		3	6	4	9		1	0	0	0	
2	2	3	2	2	2	2	1	1	1	1	1	1	5	5	2	2	3	0	0	1	1	0
1	2	3	1					1					4					0				
2	2	3	0	1	1	2		1	1	1	1		5	5	7	8		0	0	0	0	
2	2	3	3		1			2		1			3		4	3		1		0	1	
2	3	3	1	0	2	1	0	1	1	1	1	1	4	3	4	3	4	0	1	0	1	0

2	3	3	3	2	0	3	3	2	1	1	2	2	4	2	7	8	7	0	1	0	0	0
1	2	3	0	1	0			1	1	1				3	5				1	0		
1	2	3	0	1	0	1		1	1	1	1		4	5		7		0	0		0	
2	2	3	0	2	2	1		1	1	1	1		5	3	3	1		0	1	1	1	
2	1	3	4					3					3					1				
2	2	3	1	3	0	1	0	1	2	1	1	1	3	5	5	5	7	1	0	0	0	0
2	2	2	0	2	1	0	1	1	1	1	1	1	4	3	4		3	0	1	0		0
1	2	1	4	1	0	4		3	1	1	3		6	3	5	7		0	1	0	0	
1	3	3	0					1					4					0				
2	2	3	0					1					5					0				
2	2	3	1	2	4			1	1	3			4	4	7			0	0	0		
2	1	3	1	2	3			1	1	2			4	5	4	1		0	0	0	1	
2	3	3	0	1	0	3		1	1	1	2		7	7	7	9		0	0	0	0	
2	3	3	0					1					7					0				
2	1	3	3	1	0			2	1	1			5	3	3			0	1	1		
2	2	3	3					2					4					0				
1	2	2	4	2	1	2	3	3	1	1	1	2	4	4	5	8	3	0	0	0	0	0
2	3	3	1	2	0	2	1	1	1	1	1	1	7	6	7	9	5	0	0	0	0	0
2	2	3	2	4	5	5	0	1	3	3	3	1	3	3	6	6		1	1	0	0	
2	2	3	1	0	1	3	0	1	1	1	2	1	4	4	5	4	3	0	0	0	0	0
2	2	3	0		4	0		1		3	1		5		3	4		0		1	0	
2	3	3	2					1					1					1				
2	2	3	2					1					3					1				
2	2	3	1	4	1	8	4	1	3	1	3	3	4	2	3	1	1	0	1	1	1	1
2	2	3	1	1	0	0	1	1	1	1	1	1	4	6	7	8	7	0	0	0	0	0
2	2	3	0	0	0	0	1	1	1	1	1	1	4	5	7	4	5	0	0	0	0	0
2	1	2	2	1	4	2		1	1	3	1		4	3	3			0	1	1		
2	3	3	1	0	0	2	2	1	1	1	1	1	5	6	7	6	7	0	0	0	0	0
3	3	3	4	2	4			3	1	3			5	4	7	7		0	0	0	0	
2	2	3	1	6	1	3	4	1	3	1	2	3	4	2	7	8		0	1	0	0	
3	1	3	0	0	0			1	1	1			4	7				0	0			
1	2	3	0		2			1		1			4		6			0		0		
2	1	3	0					1					3					1				
2	2	3	2	0	1	2		1	1	1	1		4	4	6	8		0	0	0	0	
1	2	3	4		6			3		3			7		3			0		1		
2	3	3	3	2	2			2	1	1			4	5	7	8		0	0	0	0	
2	1	3	0	0	0			1	1	1			4	5	7	7		0	0	0	0	
2	2	3	2					1					3					1				
2	2	1	2	4	4	7		1	3	3	3		3	6	6	6		1	0	0	0	
2	2	3	1	1				1	1				4	4				0	0			
2	2	3	2		1			1		1			3		4	6		1		0	0	

2	2	3	1	1	0	1		1	1	1	1		4	5	5	7		0	0	0	0	
2	2	3	0	0	0	0		1	1	1	1		5	5	5			0	0	0		
2	3	3	3	2	1	1	0	2	1	1	1	1	4	7	7	7	4	0	0	0	0	0
2	2	2	3	3	3	3	0	2	2	2	2	1	3	4	4	3	1	1	0	0	1	1
1	2	3	1	3		7		1	2		3		1	3				1	1			
2	2	3	0	0	1		1	1	1	1		1	4	4	3		3	0	0	1		0
3	1	3	1	3	1	4	3	1	2	1	3	2	3	5	6	2	1	1	0	0	1	1
2	2	3	0	1	0	0	0	1	1	1	1	1	5	6	7	9	7	0	0	0	0	0
3	1	3	0	1	3	3		1	1	2	2		4	5	4			0	0	0		
2	3	3	1	2	2			1	1	1			3	3	2			1	1	1		
1	2	2	0	2	0	1	0	1	1	1	1	1	3	3	4	6	3	1	1	0	0	0
1	3	2	2	2				1	1				6	3				0	1			
2	2	3	0	2	0	1	0	1	1	1	1	1	4	4	6	8	4	0	0	0	0	0
1	2	1	0	4	0	2	1	1	3	1	1	1		3	4	4	4		1	0	0	0
1	3	3	0					1					3					1				
2	2	3	0	1	0	2	0	1	1	1	1	1	3	4	5	2	7	1	0	0	1	0
2	2	3	2		4	3	1	1		3	2	1	5		7	8	4	0		0	0	0
2	3	1	2	1	3	0	1	1	1	2	1	1	2	5	7	8	7	1	0	0	0	0
2	2	3	0	3	2			1	2	1			4	4	6	1		0	0	0	1	
2	2	3	2		1			1		1			5		7			0		0		
2	2	3	1					1					4					0				
2	1	3	0	1	0	1		1	1	1	1		4	3		2		0	1		1	
2	2	2	0	2	0	1	2	1	1	1	1	1	5	7	7	9	4	0	0	0	0	0
1	1	3	0					1					3					1				
1	2	3	3	2	1	3		2	1	1	2		3	2	6	8		1	1	0	0	
2	2	3	1	2	0	2	0	1	1	1	1	1	4	7	6	8	7	0	0	0	0	0
2	2	3	1	2	2	2	0	1	1	1	1	1	5	3		6		0	1		0	
2	2	3	0					1					3					1				
1	2	1	0	1				1	1				3	3				1	1			
2	2	3	0	1	2			1	1	1			4	6	7	8		0	0	0	0	
1	1	3	0	1	2			1	1	1			5	5	5			0	0	0		
1	2	1	4			0	1	3			1	1	7			7	7	0			0	0
2	2	3	2		2	3		1		1	2		6		4	2		0		0	1	
3	3	4	3	3				2	2				1	6				1	0			
2	2	3	1	1	0	1		1	1	1	1		4	5	5	5		0	0	0	0	
2	2	3	0	1	0	1	1	1	1	1	1	1	4	7	7	9	6	0	0	0	0	0
2	2	3	2	1	1	2		1	1	1	1		4	4	5	3		0	0	0	1	
2	2	3	0	1	0	2	0	1	1	1	1	1	3		5	4	5	1		0	0	0
1	2	1	3					2					1					1				
2	2	1	1		2			1		1			3		2			1		1		
2	3	3	0	1	1	2	0	1	1	1	1	1	5	6	7	9	7	0	0	0	0	0

2	2	3	0	2	0			1	1	1			3	5	6			1	0	0		
2	2	3	1	0	1	1		1	1	1	1		3	5	7	9		1	0	0	0	
2	2	3	0	1	0	2	1	1	1	1	1	1	6	3	6	8	6	0	1	0	0	0
2	2	3	0	0				1	1				4	5				0	0			
2	2	3	0	3	2	0	3	1	2	1	1	2	4		4	2	7	0		0	1	0
2	1	1	2			3	1	1			2	1	3			1	1	1			1	1
2	3	3	2	1	2	1	1	1	1	1	1	1	4	5	5		4	0	0	0		0
2	2	2	0	3	1	1		1	2	1	1		4	4	6	3		0	0	0	1	
2	2	4	4	1	3	2		3	1	2	1		2	2	4	5		1	1	0	0	
2	2	3	0	0	1	1		1	1	1	1		5	5	7	8		0	0	0	0	
2	2	3	1		1	4		1		1	3		6		7	5		0		0	0	
2	1	2	4					3					2					1				
2	2	3	5	3				3	2				4	4				0	0			
2	2	1	2	1	1	0	0	1	1	1	1	1	2	5	7	4	1	1	0	0	0	1
2	3	3	0			1	0	1			1	1	3			8	2	1			0	1
2	3	3	3	4	3	2	0	2	3	2	1	1	4	4	3	4	1	0	0	1	0	1
2	3	3	4	1	1		0	3	1	1		1	1	7	3	7	4	1	0	1	0	0
2	3	3	0	0	1	4		1	1	1	3		3	4	7	7		1	0	0	0	
2	2	3	0	2	1			1	1	1			5	6				0	0			
2	2	2	0	1	0	7		1	1	1	3		4	5	7	9	7	0	0	0	0	0
2	3	1	6	1	2	5		3	1	1	3			2	1	1			1	1	1	
1	1	1	3					2														
1	2	1	0	0	2	0		1	1	1	1		2	3	5	4		1	1	0	0	
2	2	3	0	1	3	1	1	1	1	2	1	1	4	5	3	1	4	0	0	1	1	0
2	2	3	0	2	1	1		1	1	1	1		3	4	6	6		1	0	0	0	
1	2	3	0	0	0			1	1	1			2	2	7			1	1	0		
2	2	2	2	0	0	0	1	1	1	1	1	1	5	6	7	9	5	0	0	0	0	0
2	2	3	0	0				1	1				3	4				1	0			
2	2	3	0	1	0	0	1	1	1	1	1	1	4	4	3	7	6	0	0	1	0	0
2	3	3	1	2	0	4	3	1	1	1	3	2	6	4	6	7		0	0	0	0	
1	2	3	2	5				1	3					2					1			
2	2	3	1	1	1	0	0	1	1	1	1	1	4	6	7	2	4	0	0	0	1	0
2	2	3	0		0	2		1		1	1		4		7	7		0		0	0	
3	2	3	2	3	2	3	2	1	2	1	2	1	3	3	2		1	1	1	1		1
2	3	3	0	1	0	0	0	1	1	1	1	1	4	4	4	6	4	0	0	0	0	0
1	1	3	0	3	4			1	2	3			4	5	2			0	0	1		
2	2	2	4					3					3					1				
2	3	3	0	2	0	2	1	1	1	1	1	1	4	6	7	9	7	0	0	0	0	0
1	2	1	3	3	1			2	2	1			2	3	5	2		1	1	0	1	
2	2	3	0	4	2			1	3	1			4	4	4			0	0	0		
2	2	3	0	1	2	1		1	1	1	1		5	5	7	8		0	0	0	0	

2	2	3	0					1					3					1				
2	2	1	3	7	4	7		2	3	3	3		4	2	2	6		0	1	1	0	
2	3	3	2	1	3	2	0	1	1	2	1	1	3	2	1	1	1	1	1	1	1	1
2	1	3	0	1	0	4		1	1	1	3		4	6	7	8		0	0	0	0	
1	1	2	3					2					2					1				
2	2	3	1	0	0		1	1	1	1		1	4	7	7		3	0	0	0		0
2	3	3	3	2	2	1	1	2	1	1	1	1	5	3	5	4	4	0	1	0	0	0
1	2	1	3	3	2	1	4	2	2	1	1	3	3	2	2	4	3	1	1	1	0	0
2	2	3	2	3	2			1	2	1			4	4	4	2		0	0	0	1	
2	1	1	3					2					2					1				
2	2	3	0	1	0	1		1	1	1	1		4	4	7	6		0	0	0	0	
1	1	3	5	0				3	1				4	4				0	0			
2	2	2	0		4	6		1		3	3		4		3	2		0		1	1	
3	2	3	3	1	3	2		2	1	2	1		4	7	2	9		0	0	1	0	
2	1	3	1		0			1		1			3		2			1		1		
2	3	3	3	3	3	4	1	2	2	2	3	1	3	5	3	1	4	1	0	1	1	0
2	3	3	3	1	3		0	2	1	2		1	4	6	7	8	4	0	0	0	0	0
2	2	2	3					2					5					0				
2	3	3	0	2	0	1		1	1	1	1		4	3	7	7		0	1	0	0	
2	2	3	0	2	2	2		1	1	1	1		4	7	7	7		0	0	0	0	
2	1	4	1	2	3			1	1	2			5	2	6	1		0	1	0	1	
1	2	1	1	2				1	1				3	4				1	0			
2	2	3	0		0	4		1		1	3		5		7	2		0		0	1	
2	2	2	0	0	2			1	1	1			5	2	1			0	1	1		
2	2	3	3	0	3	2	0	2	1	2	1	1	4	4	3	2	4	0	0	1	1	0
2	2	3	1	0	0	0	0	1	1	1	1	1	4	5	7	4	7	0	0	0	0	0
2	2	3	0	0	0	5	4	1	1	1	3	3	6	7	4	3	4	0	0	0	1	0
2	2	2	1	4	3			1	3	2			3	2	6			1	1	0		
2	2	3	2	3	2	2	0	1	2	1	1	1	3	4	7	8	3	1	0	0	0	0
2	2	3	1					1					3					1				
2	2	3	1	1	0	0	1	1	1	1	1	1	5	5	7	5	5	0	0	0	0	0
1	1	2	3	2	4	3	0	2	1	3	2	1	5	6	5	3	4	0	0	0	1	0
2	3	2	1	3				1	2				2	7				1	0			
2	1	3	4	1	2	2		3	1	1	1		3	4	5	1		1	0	0	1	
1	2	2	3		2	3	0	2		1	2	1	7		7	6	7	0		0	0	0
2	1	3	1	2	0	1		1	1	1	1		3	5	7	9		1	0	0	0	
2	2	2	0	1	0	1		1	1	1	1		3	6	6	7		1	0	0	0	
2	1	3	3	4	2			2	3	1			2	2	3			1	1	1		
1	2	3	1	2	1	0	0	1	1	1	1	1	5	4	7	2	6	0	0	0	1	0
2	2	3	0	3	3			1	2	2			3	4	7	7		1	0	0	0	
2	1	3	0	0				1	1				2	3				1	1			

2	2	3	2	2	0	3	0	1	1	1	2	1	4	3	3	1	1	0	1	1	1	1
2	1	3	1	1	3			1	1	2			4	4	2			0	0	1		
1	2	1	1	1				1	1					3					1			
2	1	3	2	3	2	1		1	2	1	1		3	3	6	3		1	1	0	1	
2	2	3	0	0	1	2		1	1	1	1		3	5	7	7		1	0	0	0	
1	2	2	1	1	2	4		1	1	1	3		2	5	2	7	4	1	0	1	0	0
3	1	3	4	0	6	2	2	3	1	3	1	1	4	2	3	1	3	0	1	1	1	0
2	2	3	2		0	1		1		1	1		5		7	8		0		0	0	
3	1	3	2	1	1	0		1	1	1	1		4	5	7	1		0	0	0	1	
2	3	3	7	3	2	0		3	2	1	1		5	4	7	8		0	0	0	0	
1	1	2	1			3	1	1			2	1	4				6	0				0
3	2	3	1	0	0	3	1	1	1	1	2	1	4	5	7	8	5	0	0	0	0	0
1	2	3	0	2		2	0	1	1		1	1	4	4		9	5	0	0		0	0
1	2	1	3	1	3	6	3	2	1	2	3	2	4	2	4		3	0	1	0		0
2	1		0			7		1			3		2			1		1			1	
2	1	4	2		1			1		1			3		6			1		0		
2	3	3	0	1				1	1													
2	3	3	2		5			1		3			4		3			0		1		
2	2	3	0	2	0			1	1	1			4	7	3			0	0	1		
1	2	3	4	3	1	3	6	3	2	1	2	3	3	3	4	1	1	1	1	0	1	1
2	2	3		1	1	1	2		1	1	1	1		6	7	9	4		0	0	0	0
2	2	4	1					1					2					1				
2	2	3	2	1	0	0		1	1	1	1		3	4	5			1	0	0		
2	2	1	3		0			2		1			4					0				
2	1	2	0					1					4					0				
2	2	3		1	0				1	1			4		4			0		0		
2	2	2	1	0	1	2		1	1	1	1		4	4	5	7	3	0	0	0	0	0
2	1	1	2		1	5		1		1	3				3	1	2			1	1	1
1	2	3	1	2				1	1				5	5				0	0			
2	3	2	5	4				3	3				4	5				0	0			
2	2	3	0	2	1	2	0	1	1	1	1	1		5	7	8	5		0	0	0	0
2	2	1	1	0	2			1	1	1			4	2	3			0	1	1		
2	2	3	2	2	1		2	1	1	1		1	3	3	7		3	1	1	0		0
2	3	3	1		2		1	1		1		1	5		3	5	3	0		1	0	0
2	2	3	0	1	0	5	2	1	1	1	3	1	6	7	6	7	3	0	0	0	0	0
2	2	3	1	1	0	2		1	1	1	1		4	5	5	7		0	0	0	0	
2	1	3	2	2	0	3		1	1	1	2		3	4		1		1	0		1	
2	3	3	1					1					4					0				
2	2	3	0	1	1			1	1	1			4	4	5			0	0	0		
2	1	3	4	1	4	4		3	1	3	3		4	4	6	1		0	0	0	1	
2	1	3	0	0	1	2		1	1	1	1		4	3	5	3		0	1	0	1	

2	2	3	0	1	0	3	0	1	1	1	2	1	4	5	7	9	5	0	0	0	0	0
2	2	3	1	2	1	2		1	1	1	1		4	5	7	6		0	0	0	0	
1	3	3	2		2	1	8	1		1	1	3	4		7		6	0		0		0
2	2	2	0		1	2	1	1		1	1	1	5		6	3	1	0		0	1	1
2	1	3	1					1					4					0				
2	3	1	2	5	6			1	3	3			3	2	3			1	1	1		
2	3	3	1	1	1			1	1	1			3	2	7			1	1	0		
2	2	3	2		2			1		1			3		3			1		1		
1	2		3	5	5	6	1	2	3	3	3	1	6	4	4		1	0	0	0		1
2	2	3	0		3	1		1		2	1		4		5			0		0		
2	2	3	1					1					4					0				
2	2	3	0	1	1	2		1	1	1	1		5		7			0		0		
2	3	1	0	1				1	1				3	5				1	0			
1	2	2	3					2					2					1				
2	2	3	0	1	1	1	1	1	1	1	1	1	5	3	6	6	3	0	1	0	0	0
2	2	3	0	1	0			1	1	1				4	6				0	0		
2	1	1	1	0	2	6	3	1	1	1	3	2	3	5	3	2	1	1	0	1	1	1
2	3	3	1	2	1	1	0	1	1	1	1	1	4	5	7	8	6	0	0	0	0	0
1	2	3	1	1	2	1	3	1	1	1	1	2	3	5	1	1	7	1	0	1	1	0
2	2	3	2	1	1	7	3	1	1	1	3	2	3	6	7		3	1	0	0		0
3	2	3	1	3	4	8		1	2	3	3		5	5	2	8		0	0	1	0	
1	2	3	0	1	0		2	1	1	1		1	5	4	7		3	0	0	0		0
2	2	1	2		5			1		3			2		2			1		1		
2	2	2	0	0	0	3		1	1	1	2		4	5	6	7		0	0	0	0	
1	2	1	3	1	0	2	5	2	1	1	1	3	7	5	7		5	0	0	0		0
2	2	3	2	5	1			1	3	1				2	2				1	1		
2	2	1	2					1					2					1				
1	2	2	4	0				3	1				3	2				1	1			
2	2	3	3	1	2	2		2	1	1	1		3	4	1	1	1	1	0	1	1	1
2	1	3	1	3		0	2	1	2		1	1	4	4		8	6	0	0		0	0
2	2	3	1	1	0	1		1	1	1	1		4	4	6	5		0	0	0	0	
2	2	3	2					1					4					0				
2	2	3	1	1	1	1	1	1	1	1	1	1	4	5	7	7		0	0	0	0	
2	1	3	0					1					4					0				
2	2	3	2	1	2			1	1	1			4	2	4			0	1	0		
1	1	3	4	4				3	3				3	4				1	0			
2	2	3	0	0	1	3	1	1	1	1	2	1	5	7	7	4	7	0	0	0	0	0
2	3	3	0	0	0			1	1	1			4	4	7	8		0	0	0	0	
2	3	3	2					1					6					0				
1	2	3	1	1	5	1	0	1	1	3	1	1	4	5	7	7	7	0	0	0	0	0
2	1	3	0	3	1	1	3	1	2	1	1	2		3	4	2			1	0	1	

2	2	3	2	0	1			1	1	1				3	6	2			1	0	1	
2	2	3	0	0	0	1	1	1	1	1	1	1	6	6	7		4	0	0	0		0
2	2	3	0		6	5		1		3	3		3		4	9		1		0	0	
2	2	3	2		2			1		1			3					1				
2	2	3	1	1	1	2	1	1	1	1	1	1	3	3	4	7	5	1	1	0	0	0
2	2	1	4		4	2		3		3	1		5		3	2		0		1	1	
2	1	1	4	2	2			3	1	1			2	4	7	8		1	0	0	0	
2	2	3	0	0	0	2		1	1	1	1		4	4	4			0	0	0		
2	1	3	1					1					3					1				
2	2	3	1	1	1	0	4	1	1	1	1	3	4	3	3	8	3	0	1	1	0	0
1	2	3	0	1	0			1	1	1			4	3	4			0	1	0		
2	2	3	2	1	3	2		1	1	2	1		4	7	7	6		0	0	0	0	
2	2	3	1					1					3					1				
1	1	1	2	5				1	3				5	2				0	1			
1	2	3	0	1	0	4		1	1	1	3		4	7	7			0	0	0		
2	2	3	1		1	2		1		1	1		5		7	8		0		0	0	
2	2	3	2	1	1	4	0	1	1	1	3	1	4	7	7	9	3	0	0	0	0	0
2	2	4	3		3	1		2		2	1		2		5			1		0		
1	3	3	3	2	2	3	0	2	1	1	2	1	4	7	4	3	5	0	0	0	1	0
2	1	3	0	0	0	1	0	1	1	1	1	1	3	3	6	7	1	1	1	0	0	1
2	1	2	1		2			1		1			5					0				
2	3	3	4	4	5	0	0	3	3	3	1	1	6	5	5	6	4	0	0	0	0	0
1	2	2	1	3		2	0	1	2		1	1	3	5		7	6	1	0		0	0
2	1	3	1	0	2	5		1	1	1	3		4	4	3	7		0	0	1	0	
2	3	3	0	2	1	2		1	1	1	1		3	4	5	1		1	0	0	1	
2	2	3	1	1				1	1				5					0				
1	2	2	0	3	3	1	0	1	2	2	1	1	2	5	5	7	2	1	0	0	0	1
2	3	3	1	0	0	4		1	1	1	3		2	5	3	8		1	0	1	0	
2	2	3	0	1	0	2		1	1	1	1		3	4	5	4		1	0	0	0	
2	3	3	2		2			1		1			6		4			0		0		
2	2	3	3					2					2					1				
2	3	3	2	2		0		1	1		1		3	5				1	0			
2	2	2	0	0				1	1				3	3				1	1			
2	2	3	1					1					1					1				
1	2	1	2					1					1					1				
3	3	3	1	3		3	1	1	2		2	1	5	6		4	3	0	0		0	0
1	3	2	2	1	2	1		1	1	1	1		3	5	6	7		1	0	0	0	
2	1	3	1	4	0	1		1	3	1	1		3	2	7	4		1	1	0	0	
1	1	1	0		2	8		1		1	3				4	2				0	1	
2	1	3	1	2	2	1	2	1	1	1	1	1	4	5	5	6	4	0	0	0	0	0
2	2	3	2	2				1	1				3	3				1	1			

2	3	3	0	3	0	0	0	1	2	1	1	1	6	4	5	8	6	0	0	0	0	0
2	2	2	1		0	1		1		1	1		6		4	1		0		0	1	
2	2	3	0	0	2		0	1	1	1		1	3	3	6		3	1	1	0		0
2	2	3	0	1				1	1				4	3				0	1			
2	3	3	1	2		1		1	1		1		4	3		6		0	1		0	
2	2	3	2	1	0	1	0	1	1	1	1	1	4	4	5	1	3	0	0	0	1	0
2	1	3	1	1	5	2	1	1	1	3	1	1	3	3	3	5	3	1	1	1	0	0
2	3	3	0	0	0	1	2	1	1	1	1	1	2	5	7	8	7	1	0	0	0	0
2	1	3	1			6		1			3		3					1				
2	2	3	3	2	0	1	0	2	1	1	1	1	3	5	6	3	4	1	0	0	1	0
2	3	3	2	2	1	2		1	1	1	1		5	7	7	8		0	0	0	0	
2	2	3	1		2	1	2	1		1	1	1	5		4	6	2	0		0	0	1
2	2	2	2					1					3					1				
2	2	3	2	2	2	2	0	1	1	1	1	1		2	7	8	3		1	0	0	0
2	2	3	0	0	1	0		1	1	1	1		3	6	6	6		1	0	0	0	
2	2	3	0	2	0			1	1	1			5	6	7			0	0	0		
1	1	1	1	0	2			1	1	1			3	2	4			1	1	0		
1	2	1	2	1	1	3		1	1	1	2		3		3	5	1	1		1	0	1
2	2	3	0	2	0	1		1	1	1	1		5	7	7	8		0	0	0	0	
2	2	3	1	3	1	0	0	1	2	1	1	1	4	4	5	7	7	0	0	0	0	0
2	2	3	2	0				1	1				3	4				1	0			
2	2	3	0	3	1	1		1	2	1	1		4	5	4	5		0	0	0	0	
2	1	3	1	0	2			1	1	1			4	3	3	1		0	1	1	1	
2	3	3	1	1	1			1	1	1			5		7			0		0		
2	2	3	0	2	0	0	0	1	1	1	1	1	3	4	3	3	5	1	0	1	1	0
2	2	3	0	1	1	4		1	1	1	3		5	7	7	6		0	0	0	0	
2	2	3	0	1	2			1	1	1			4	5	3			0	0	1		
2	2	3	2	4	0	2		1	3	1	1		3	3	4	1		1	1	0	1	
2	1	3	0	1	0	0	0	1	1	1	1	1	6	5	5		3	0	0	0		0
2	2	3	0	0	2	0		1	1	1	1		5	5	5			0	0	0		
2	3	3	0	2	1			1	1	1			6	7	7			0	0	0		
2	2	3	0	3	3	2		1	2	2	1		3	4	6			1	0	0		
2	3	3	0					1					5					0				
2	2	2	2	0	2			1	1	1			4	5	7			0	0	0		
2	2	3	4	3	1	1		3	2	1	1		7	1	2	4		0	1	1	0	
2	2	3	2	2	1			1	1	1			4	4	6			0	0	0		
2	3	3	2	2	3	2	0	1	1	2	1	1	3	7	7	7	4	1	0	0	0	0
2	2	3	1	0	2	1		1	1	1	1		7	6	7	8		0	0	0	0	
1	3	1	1	2	1		4	1	1	1		3	3	4	4		3	1	0	0		0
2	3	3	3	5				2	3				4					0				
2	1	3	0	0	0	1		1	1	1	1		5	6	5	3		0	0	0	1	

1	2	3	0	3	0	2		1	2	1	1		4	7	7	9		0	0	0	0	
2	2	3	5	1				3	1				4	2				0	1			
1	2	3	1	1	2			1	1	1			4	5	4	2		0	0	0	1	
2	3	3	1	2	0		1	1	1	1		1	5	7	7	9	3	0	0	0	0	0
1	2	3	4	1	0			3	1	1			3	6	7	8		1	0	0	0	
2	2	1	3	3				2	2					6					0			
2	2	3	3	3				2	2				5	4				0	0			
2	3	3	1	1	2			1	1	1			6	5	7			0	0	0		
2	1	3	0	2	0	1	1	1	1	1	1	1	4	7	5	6	3	0	0	0	0	0
2	3	1	1	2	2			1	1	1			3	3	7	4		1	1	0	0	
2	2	3	1	1				1	1				3					1				
1	2	2	1					1					2					1				
2	2	3	0	4		5	3	1	3		3	2	4	5		1	1	0	0		1	1
2	2	4	4					3					3					1				
2	2	3	1	1				1	1				4	4				0	0			
1	1	2	3		1			2		1			3		5			1		0		
2	2	3	0	0	1	6	3	1	1	1	3	2	3	4	3	4	1	1	0	1	0	1
2	2	3	1	1	0			1	1	1			5	3	7			0	1	0		
2	2	3	4					3					4					0				
2	2	3	0					1					5					0				
2	1	3	0			2	2	1			1	1	4			2	4	0			1	0
2	2	3	4	1	1		2	3	1	1		1	3	4	7	7	4	1	0	0	0	0
2	3	4	3	2	5	0	0	2	1	3	1	1	4	2	3	2	4	0	1	1	1	0
2	2	3	5	2	2		3	3	1	1		2	4	4	4	8	1	0	0	0	0	1
2	2	3	0	1	3		1	1	1	2		1	5	7	7	9	1	0	0	0	0	1
3	1	3	0	1	3	4		1	1	2	3		4	5	6			0	0	0		
2	2	3	0	0				1	1				3	3				1	1			
2	2	3	0	0		0		1	1		1		4	4				0	0			
2	2	2	0					1					4					0				
2	2	3	4	1	0	4	3	3	1	1	3	2	5	7	7	9	7	0	0	0	0	0
2	2	2	1		1	2	2	1		1	1	1	4		7	8		0		0	0	
2	2	3	0	0	0		0	1	1	1		1	4	4	7	8	3	0	0	0	0	0
2	2	2	4		2			3		1					6					0		
2	3	3	1					1					2					1				
1	2	1	1					1					2					1				
2	2	3	2		5	2	5	1		3	1	3	3		7	8	1	1		0	0	1
1	2	3	2	2	4	0		1	1	3	1		5	3	5	6		0	1	0	0	
3	2	3	2	0	1			1	1	1			5	5	7			0	0	0		
2	2	3	0	3	0			1	2	1			4	3	7	8		0	1	0	0	
2	2	3	1	1	0	2	0	1	1	1	1	1	4	5	7	3	1	0	0	0	1	1
1	2	3	2	3	1			1	2	1			3	2	7			1	1	0		

2	2	1	3	3	3	0		2	2	2	1		2	6	3			1	0	1		
1	2	1	1	3				1	2					6					0			
2	2	3	0					1					4					0				
2	2	3	1	1	0	2	0	1	1	1	1	1	5	4	7	8	7	0	0	0	0	0
1	2		1	1	2	1	8	1	1	1	1	3	3	4	4		4	1	0	0		0
2	1	3	0	0	0			1	1	1			3	3	3			1	1	1		
2	2	2	2	2	1	4	3	1	1	1	3	2	5	1	5	2	1	0	1	0	1	1
2	1	3	0					1					3					1				
2	2	3	1	1	0	0		1	1	1	1		4	5	7	9	4	0	0	0	0	0
2	2	3	4	3	2	1		3	2	1	1		4	2	3	4		0	1	1	0	
2	1	3	1					1					4					0				
2	3	3	0	0	0	2	0	1	1	1	1	1	4	6	4	1	6	0	0	0	1	0
2	2	3	0			7	2	1			3	1	3			2	3	1			1	0
3	2	3	0	3	4	3	0	1	2	3	2	1	4	4	7	1	3	0	0	0	1	0
2	2	3	4	0	0	5		3	1	1	3		3	3	5	6		1	1	0	0	
2	3	3	3	0	4	2	0	2	1	3	1	1	4	5	6	4	6	0	0	0	0	0
1	1	1	3	0				2	1				3					1				
2	2	3	3		0	2		2		1	1		7		6			0		0		
2	2	3	3	2	4			2	1	3			3	3	3	3		1	1	1	1	
1	2	1	1		4		3	1		3		2	3		3		4	1		1		0
3	3	3	0	0	6			1	1	3			4	4	1			0	0	1		
2	2	2	2	3	1	1		1	2	1	1		6	7	7			0	0	0		
2	2	3	1	2	0	2		1	1	1	1		4	3	4	6		0	1	0	0	
2	2	3	0	0	1	3		1	1	1	2		3	4	7	1		1	0	0	1	
2	2	3	5		1		1	3		1		1	2		5		1	1		0		1
2	1	3	2					1					3					1				
2	3	3	1	1		1	3	1	1		1	2	7	5		8	2	0	0		0	1
1	2	2	3					2					4					0				
2	1	3	0	2	0			1	1	1			1	3	2			1	1	1		
2	2	3	3					2					4					0				
2	1	3	0					1					3					1				
1	2	1	0					1					3					1				
2	3	1	5	0	0	3		3	1	1	2		3	3	6	7		1	1	0	0	
2	2	3	0	0	2	0	0	1	1	1	1	1	4	5	4	8	4	0	0	0	0	0
2	2	3	1		4	1	1	1		3	1	1	3		3	1	6	1		1	1	0
3	3	3	0	1	0	0	0	1	1	1	1	1	3	4	4	3	1	1	0	0	1	1
2	1	1	1		3	2	0	1		2	1	1	3		4	5	2	1		0	0	1
2	2	3	1	1	0	0	0	1	1	1	1	1	5	6	3	6	7	0	0	1	0	0
2	2	3	0	1	0	4	2	1	1	1	3	1	4	4	6	6	1	0	0	0	0	1
1	1	4	0	1	1	3	0	1	1	1	2	1	2	3	4	1	2	1	1	0	1	1
2	2	3	1	0	0			1	1	1			5	7	7			0	0	0		

2	3	3	0	2	0	0		1	1	1	1		4	3	5			0	1	0		
2	2	2	2	1	2			1	1	1			6	7	6			0	0	0		
1	1	3	5	2	1	2		3	1	1	1		3	2	4	4	3	1	1	0	0	0
1	1	2	3		2	2	0	2		1	1	1	2		3	4	4	1		1	0	0
2	2	3	1	0	2	0		1	1	1	1		6	3	4	2		0	1	0	1	
2	3	3	0	2	0	2	2	1	1	1	1	1	5	7	7	8	1	0	0	0	0	1
2	2	3	1	2	2	4		1	1	1	3		5	2	7	7		0	1	0	0	
2	3	3	2					1					3					1				
2	3	3	2	3	3	5	1	1	2	2	3	1	3	4	3	1	3	1	0	1	1	0
2	3	3	2	1	0	1	0	1	1	1	1	1	4	6	7	4	7	0	0	0	0	0
1	2	3	1	3	3	1	0	1	2	2	1	1	4	3	3	3	3	0	1	1	1	0
1	2	2	1	3	0			1	2	1			3	5	3			1	0	1		
2	3	2	2		1	3		1		1	2		4		7			0		0		
2	2	2	1	0				1	1													
2	2	3	0	1	0	0		1	1	1	1		6	6	7	9		0	0	0	0	
2	2	3	1	2	0	2	0	1	1	1	1	1	5	5	7	7	2	0	0	0	0	1
2	2	3	0	1	0	0	0	1	1	1	1	1	4	7	7	7	5	0	0	0	0	0
2	1	3	0					1					4					0				
2	2	3	0	1	2	2	0	1	1	1	1	1	4	3	3	2	1	0	1	1	1	1
2	2		0		1			1		1			6		2	1		0		1	1	
2	2	3	0	1	1			1	1	1			5	4	5	6		0	0	0	0	
2	2	3	0	1	0	0		1	1	1	1		5	6	7	6		0	0	0	0	
2	1	3	3		0	2		2		1	1		4		7			0		0		
1	2	2	0	1	3			1	1	2			2	5	3			1	0	1		
2	1	2	3					2					2					1				
2	2	3	1	3	0	2		1	2	1	1		4	5	5	1		0	0	0	1	
2	2	3	2	3				1	2				4	4				0	0			
2	3	4	4					3					6					0				
2	2	3	2	2	0			1	1	1			5	5	6			0	0	0		
2	2	4	1					1					2					1				
2	1	3	3	1	1	4		2	1	1	3		3	6	5	7		1	0	0	0	
2	2	3	4	0	1			3	1	1			2	3	4			1	1	0		
2	2	3	0	1	0			1	1	1			5	6	7			0	0	0		
2	2	3	0	2	2	0	0	1	1	1	1	1	4	3	5	3	3	0	1	0	1	0
2	2	3	1	2				1	1				6	6				0	0			
1	2	3	1	1	5	3		1	1	3	2			3	2				1	1		
2	2	3	0					1					3					1				
2	3	3	1	1	0	0	0	1	1	1	1	1	6	6	4	8	4	0	0	0	0	0
2	1	3	0	0	0	1	2	1	1	1	1	1	4	5	7	7	5	0	0	0	0	0
2	3	3	2	1	0	2	0	1	1	1	1	1	4	7	7	9	7	0	0	0	0	0
2	2	2	5	4				3	3				3	4				1	0			

2	3		1	1	1	2	1	1	1	1	1	1	5		6	1	3	0		0	1	0
3	2		0					1					4					0				
2	3	3	0	1	3			1	1	2			4	4	2	3		0	0	1	1	
2	2	3	0					1					3					1				
1	2	3	2	1	0	1		1	1	1	1		3	3	5	8		1	1	0	0	
1	2	3	2	0	1	1	0	1	1	1	1	1	5	5	7	8	4	0	0	0	0	0
2	3	3	2	1	0			1	1	1			4	3	3	7		0	1	1	0	
2	2	3	0	0	0	0		1	1	1	1		5	5	6			0	0	0		
3	3	2	0	2	3	1	0	1	1	2	1	1	6	6	6	3	1	0	0	0	1	1
2	2	3	1	1	1	1		1	1	1	1		5	6	3		4	0	0	1		0
2	2	3	0	0	0	0		1	1	1	1		6	4	6	7	6	0	0	0	0	0
2	1	3	1	1	2	0	4	1	1	1	1	3	4	4	2	3		0	0	1	1	
2	2	3	0	1				1	1				4	5				0	0			
1	2	3	0	0	0			1	1	1			3	4	7			1	0	0		
2	2	3	0	1	0	3	0	1	1	1	2	1	7	7	6	8	7	0	0	0	0	0
1	3	1	3	4	6	5	1	2	3	3	3	1	3	5	3	3	3	1	0	1	1	0
2	2	3	1		0			1		1			6		7	9		0		0	0	
2	3	3	0	3	1	2	0	1	2	1	1	1	4	3	5	6	3	0	1	0	0	0
2	2	3	0	0	1	1	0	1	1	1	1	1	5	6	7	8	5	0	0	0	0	0
2	3	3	1	1	0	1	2	1	1	1	1	1	3	4	7	8	4	1	0	0	0	0
1	2	4	1	1		4		1	1		3			4		1			0		1	
2	2	3	1	1	0	1		1	1	1	1		4	5	5	6		0	0	0	0	
2	2	4	2					1					2					1				
2	2	3	1	2	2	0	2	1	1	1	1	1	5	7	6	8	5	0	0	0	0	0
2	3	3	0					1					4					0				
2	1	3	1	2	1	1	2	1	1	1	1	1	5	6	7		5	0	0	0		0
2	2	3	1	1	2	6		1	1	1	3		4	1	6			0	1	0		
2	2	3	0	1	0	3		1	1	1	2		4	5	7	9		0	0	0	0	
1	2	3	2	1	1		1	1	1	1		1	3	3	4	3	3	1	1	0	1	0
2	3	3	0	2	4	0	3	1	1	3	1	2	4	3	5	9	3	0	1	0	0	0
2	3	3	1	1				1	1				5	5				0	0			
2	1	3	1	3	3			1	2	2			5	7	6			0	0	0		
2	2	3	0	1	0	0	2	1	1	1	1	1	4	4	7	6	5	0	0	0	0	0
2	2	2	2	1	1			1	1	1			4	4	4			0	0	0		
1	2	1	0	2	3	5		1	1	2	3		2	3	3	5		1	1	1	0	
2	3	3	0	2	4	2	0	1	1	3	1	1	4	7	5	3	5	0	0	0	1	0
1	2	3	0	3	0	0		1	2	1	1			5	7	8			0	0	0	
2	2	3	1					1					4					0				
2	3	3	1		3	2		1		2	1		5		4	6		0		0	0	
2	2	3	0	0	0	3	1	1	1	1	2	1	4	3	7	7	5	0	1	0	0	0
2	2	3	1					1					4					0				

1	2	2	1					1					2					1				
2	2	3	1	4	0	0		1	3	1	1		3	5	7			1	0	0		
2	2	3	1		2	2		1		1	1		3		7	1		1		0	1	
2	2	2	3	2	3			2	1	2			4		3	6		0		1	0	
2	2	3	0	3	4	4	2	1	2	3	3	1	4	4	7	4	1	0	0	0	0	1
2	2	3	3	2	0		2	2	1	1		1	3	4	6		3	1	0	0		0
2	2	3	0	2	1	1	2	1	1	1	1	1	4	3	2	1	4	0	1	1	1	0
3	1	3	0	0	0		3	1	1	1		2		4	7	1	1		0	0	1	1
2	3	3	6	3	0			3	2	1			3	5	7			1	0	0		
2	2	3	1	2	5			1	1	3			6	4	1	1		0	0	1	1	
2	2	3	1	0	1	1	0	1	1	1	1	1	4	4	5	4	4	0	0	0	0	0
2	1	3	1	0	1	2		1	1	1	1		3	7	7	6		1	0	0	0	
2	2	3	2	0				1	1				3	7				1	0			
2	2	3	1		0			1		1			4		7			0		0		
2	2	3	1	1	2	2		1	1	1	1		6	3	2	8		0	1	1	0	
2	2	3	1	4	4	1	2	1	3	3	1	1	6	6	5	8	6	0	0	0	0	0
2	3	3	0	2	3	5		1	1	2	3		4	4	2	9		0	0	1	0	
2	2	2	0	0	0	1	1	1	1	1	1	1	3			4	3	1			0	0
2	2	1	3					2														
1	2	3	0	2	2			1	1	1			4	4	4			0	0	0		
2	2	3	3		2	5		2		1	3		5		7	9		0		0	0	
2	2	3	0	2	1			1	1	1			4	3	5			0	1	0		
1	1	1	1	2	1	4	1	1	1	1	3	1	2	2	3	3	3	1	1	1	1	0
2	2		1		1			1		1			5		7			0		0		
2	2	4	1	3	2	3		1	2	1	2		2	2	3	4		1	1	1	0	
2	1	3	1	1	3			1	1	2			4	4	5			0	0	0		
2	2	3	1	0	1	1	2	1	1	1	1	1	6	5	7	8	5	0	0	0	0	0
2	2	1	2					1					2					1				
2	2	2	4					3					3					1				
2	3	3	3	1	1	0	1	2	1	1	1	1	5	5	7	7	4	0	0	0	0	0
2	2	3	3	5	4			2	3	3			5	5	7			0	0	0		
2	2	3	0	2	0			1	1	1			3	7	7			1	0	0		
2	2	3	2		1	0	1	1		1	1	1	5		7	7	6	0		0	0	0
2	3	3	3	0	1	2	0	2	1	1	1	1	4	5	7	5	1	0	0	0	0	1
2	2	3	1	0	1			1	1	1			4	4	5	7		0	0	0	0	
2	3	3	2	2	1	0	0	1	1	1	1	1	3		3	1	3	1		1	1	0
2	2	3	1	0	1	1	0	1	1	1	1	1	6	4	6	7	6	0	0	0	0	0
2	3	3	0	1	1	2		1	1	1	1		6	4	7	8		0	0	0	0	
1	1	3	2	2	0	3		1	1	1	2		4	4	6	7		0	0	0	0	
2	2	1	3	4	5			2	3	3			5	6	1	2		0	0	1	1	
1	2	3	1	4	4	3		1	3	3	2		3	2	2	1	1	1	1	1	1	1

2	2	3	0	0	0	2		1	1	1	1		1	5	7	7		1	0	0	0	
2	2	3	0	0	5	5	0	1	1	3	3	1	4	5	3		7	0	0	1		0
2	2	2	0	0	0	0	0	1	1	1	1	1	4	5	7	7	3	0	0	0	0	0
2	2	3	1	0	0	1	1	1	1	1	1	1	4	5	7	8	2	0	0	0	0	1
2	2	3	0	1				1	1				3	4				1	0			
2	3	3	1		4			1		3			7		7	4		0		0	0	
2	1	3	2					1					3					1				
2	2		2					1					4					0				
2	2	3	0					1					4					0				
2	1	3	1					1					6					0				
2	1	3	0	1	0	0	1	1	1	1	1	1	4	4	7	8	3	0	0	0	0	0
2	3	3	3	2	1	0	1	2	1	1	1	1	4	4	5	7	7	0	0	0	0	0
2	1	3	0	2	0	2		1	1	1	1		5	5	7	8		0	0	0	0	
3	3	3	1	1	1	2	0	1	1	1	1	1		4	7	8	3		0	0	0	0
2	1	3	0					1					3					1				
2	1	3	3					2					3					1				
2	3	3	1	1	2		1	1	1	1		1	4	5	4	5	1	0	0	0	0	1
2	2	3	2	0	0	5		1	1	1	3		4	6	5			0	0	0		
2	2	3	0	0	1			1	1	1			4	7	7			0	0	0		
2	2	3	2	2	4			1	1	3			4	4	4	3		0	0	0	1	
2	2	3	3					2														
2	1	3	0					1					4					0				
2	2	3	3					2					4					0				
2	2	3	4					3														
2	1	3	0	2	1	0	0	1	1	1	1	1	3	3	6	3	4	1	1	0	1	0
1	2	1	1	4	2	4	2	1	3	1	3	1	4	3	3	2	2	0	1	1	1	1
1	1	1	5	3	6			3	2	3			2	2	1			1	1	1		
2	2	3	1	3	1	1	0	1	2	1	1	1	4	2	2	6	6	0	1	1	0	0
2	2	3	0		1	1	0	1		1	1	1	4		7	8	2	0		0	0	1
2	2	3	0	0	1	2		1	1	1	1		4	5	7	8		0	0	0	0	
2	2	3	0	2	2	1		1	1	1	1		3	3	4	7		1	1	0	0	
1	1	2	0	1	1			1	1	1			4	6	3	2		0	0	1	1	
1	2	2	2					1					4					0				
2	1	3	3					2					1					1				
2	1	3	0		2			1		1			6		7			0		0		
1	2	3	3	1	0	1	1	2	1	1	1	1	5	6	7	8	4	0	0	0	0	0
2	2	3	1	0	0			1	1	1			3	6	7			1	0	0		
2	2	3	1	1	2	1		1	1	1	1		3	5	5	2		1	0	0	1	
2	1	3	1	0	1	1	2	1	1	1	1	1	5	6	7	8	1	0	0	0	0	1
2	3	3	1	1	2			1	1	1			4	5	7			0	0	0		
2	2	3	3		2			2		1			5		6			0		0		

2	1	2	2		2	4		1		1	3		3		3	1		1		1	1	
2	2	3	0	1	0	4		1	1	1	3		3	3	1			1	1	1		
3	2	3	0	0	0	2		1	1	1	1		5	4	6	4		0	0	0	0	
2	2	1	0					1														
2	1	3	1	4	2	0		1	3	1	1		3	5	3	1		1	0	1	1	
2	2		4		5			3		3			3		4			1		0		
2	3	3	1	2	2	2		1	1	1	1		6	6	7	8		0	0	0	0	
2	2	3	0	3	1	0		1	2	1	1		4	6	7			0	0	0		
2	1	3	2					1					3					1				
2	2	3	1					1					2					1				
1	1	3	4	1	1	2	1	3	1	1	1	1	2	3	2	1	6	1	1	1	1	0
2	2		1					1					3					1				
2	2	3	4	2	2	4	7	3	1	1	3	3	3	4	2	2	3	1	0	1	1	0
1	2	2	1					1					3					1				
2	3	3	4	4	2	1	1	3	3	1	1	1	3	7	7	9	7	1	0	0	0	0
2	1	3	0	0				1	1				3	3				1	1			
1	2	2	3	1	1	4	2	2	1	1	3	1	3	7	7	9	3	1	0	0	0	0
2	2	3	1	2	1	1	0	1	1	1	1	1	5	5	3	6	6	0	0	1	0	0
2	2	3	1	4	2	2		1	3	1	1		4	3	2	2		0	1	1	1	
1	2	3	1					1					3					1				
2	1	2	3	1	1			2	1	1			1	3	5			1	1	0		
2	1	3	3	2	7	4		2	1	3	3		3	4	7	1		1	0	0	1	
2	2	3	2	2				1	1				3	2		3		1	1		1	
2	1	3	0	1	0	0	2	1	1	1	1	1	5	5	6	2	6	0	0	0	1	0
1	1	2	2					1														
2	2	3	1	3	2	2		1	2	1	1		4	4	3	1		0	0	1	1	
2	2	3	1	2	3	2		1	1	2	1		5	6	3	1		0	0	1	1	
2	2	3	0	1	0	2		1	1	1	1		4	3	7	1		0	1	0	1	
1	2	3	0	2	2	2		1	1	1	1		5	6	7	1		0	0	0	1	
2	1	3	2	1	4			1	1	3			6	4	2			0	0	1		
2	2	3	0	2	2	2	3	1	1	1	1	2	4	4	3	1	5	0	0	1	1	0
1	2	3	3					2					6					0				
2	2	3	0	1	1		0	1	1	1		1	6	4	5	8	1	0	0	0	0	1
1	2	3	0		2			1		1			3		5			1		0		
2	2	3	5	2	3			3	1	2			3	4	3			1	0	1		
3	1	2	1					1					3					1				
2	2	3	0		3	2		1		2	1		3		5	8		1		0	0	
2	1	3	0	2	1	0	1	1	1	1	1	1	4	5	7	2	7	0	0	0	1	0
2	2	3	1	0	0	0	2	1	1	1	1	1	4	4	7	8	7	0	0	0	0	0
2	2	3	1	2	2	3		1	1	1	2		4	4	3	1		0	0	1	1	
2	2	3	1					1														

2	2	3	2					1														
2	3	3	1	0	0	1	2	1	1	1	1	1	7	5	7		3	0	0	0		0
3	1	3	0	1	1			1	1	1			5	4	5	1		0	0	0	1	
2	1	3	4			2	3	3			1	2	3				1	1				1
1	1	3	0	2	0	3		1	1	1	2		4	3	3			0	1	1		
2	1	3	3	3	1			2	2	1			4	6	7			0	0	0		
2	2	3	0	0	0	1		1	1	1	1		3	4	5	3	5	1	0	0	1	0
2	1	3	0		2			1		1			5		5			0		0		
2	2	3	2	1	1			1	1	1			3	2	2			1	1	1		
1	2		1			2		1			1		6					0				
2	2	3	1		3	0		1		2	1		4		5	2		0		0	1	
2	1	1	0	0	4	2	0	1	1	3	1	1		3	3	4	4		1	1	0	0
2	3	3	0		2			1		1			6		7	7		0		0	0	
2	3	3	1	0	1	0	1	1	1	1	1	1	4	7	7	8	5	0	0	0	0	0
2	2	3	1	0	0	3	1	1	1	1	2	1	4	5	7	2	5	0	0	0	1	0
1	3	1	5	5	1	1	0	3	3	1	1	1	2	2	4	3	4	1	1	0	1	0
2	1	3	0	1	1			1	1	1			4	3				0	1			
2	2	3	0	1	2	1		1	1	1	1		5	3	6	1		0	1	0	1	
2	1	3	1	2	0	2	2	1	1	1	1	1	3	5	7	6	7	1	0	0	0	0
2	2	3	3	3	2	2		2	2	1	1		6	6	7	7	4	0	0	0	0	0
2	2	3	3					2					5					0				
2	2	3	4	3	2		3	3	2	1		2	3	4	7	4	3	1	0	0	0	0
2	1	3	3		6	2		2		3	1		2		4			1		0		
1	1	2	0		3			1		2					3					1		
2	1	2	1	2	1	0	0	1	1	1	1	1	4	4	7	8	2	0	0	0	0	1
2	2		0	0				1	1					4					0			
2	2	3	3					2					5					0				
2	1	3	0	0	0			1	1	1			3	4	4			1	0	0		
2	3	3	1	0	0	2	0	1	1	1	1	1	4	7	7	9	3	0	0	0	0	0
2	2	3	1	1	1	0		1	1	1	1		4	6	7	3		0	0	0	1	
2	2	3	3		0		1	2		1		1	4		7	8	4	0		0	0	0
1	3	3	0	1	0	1		1	1	1	1		3	7	7	8		1	0	0	0	
2	2	3	1	1	0		0	1	1	1		1	3		7		7	1		0		0
2	2	1	3	2	2	2	0	2	1	1	1	1	2	4	4		7	1	0	0		0
1	1	3	2		0	1		1		1	1		4		5			0		0		
2		3	0					1					3					1				
2	2	3	0	2	0	3	2	1	1	1	2	1	4	4	5	3	5	0	0	0	1	0
1	2	3	2	3	3	2	2	1	2	2	1	1	3	4	6	4	3	1	0	0	0	0
3	1	3	0	1	1			1	1	1			3	3				1	1			
2	3	3	1	3	1		1	1	2	1		1	5	7	7	8	5	0	0	0	0	0
2	2	3	0					1					4					0				

2	1	3	1	2	1			1	1	1			4	4	7			0	0	0		
2	1	2	0	2	1			1	1	1			3	5	6			1	0	0		
2	2	3	2	0	0	1		1	1	1	1		4	5	7	8		0	0	0	0	
2	2	3	3	4	5		2	2	3	3		1	4	6	4	7	3	0	0	0	0	0
2	2	3	0					1					3					1				
2	1	4	1					1					2					1				
2	1	3	2	2	0	3		1	1	1	2		3	3	6	7		1	1	0	0	
2	1	3	2		2			1		1			3		4	3		1		0	1	
2	2	3	2		2	2		1		1	1		5		4			0		0		
2	1	3	0	0	0	1		1	1	1	1		4	4	7	5		0	0	0	0	
1		3		0	1				1	1				6	7				0	0		
1	1		1	1	0			1	1	1			2	5	7			1	0	0		
2	3	3	1	1	1	0	0	1	1	1	1	1	7	6	6	9	5	0	0	0	0	0
2	2	3	2		0	2	0	1		1	1	1	5		5	5	4	0		0	0	0
2	2	3	0	0	0	0	0	1	1	1	1	1	5	7	7	9	3	0	0	0	0	0
3	1	3	2	1	1	0		1	1	1	1		3	5	7	3		1	0	0	1	
2	3	3	2	1	0	0	0	1	1	1	1	1	3	6	5	8	3	1	0	0	0	0
3	3	3	2	0	2	1	1	1	1	1	1	1	4	4	7	7		0	0	0	0	
2	3	3	1	3	7	3		1	2	3	2		6	4	7	8	3	0	0	0	0	0
2	3	3	0	1	1		0	1	1	1		1	3	3	7		3	1	1	0		0
2	2	3	1	2	0	1	2	1	1	1	1	1	4		3	3	3	0		1	1	0
2	3	3	1	0	0	1	0	1	1	1	1	1		6	7	9	6		0	0	0	0
2	3	3	3		1			2		1			4		7			0		0		
2	2	3	1	1	0	1	0	1	1	1	1	1	4	5	2	7	3	0	0	1	0	0
3	3	3	2	3	0	1	0	1	2	1	1	1	4	7	7	9	7	0	0	0	0	0
2	2	1	0	1		0		1	1		1		7	6		6		0	0		0	
2	2	3	1	3	4			1	2	3			5	3	3			0	1	1		
2	2	2	2					1					4					0				
2	2	3	3	3	1	1	1	2	2	1	1	1	4	6	7	8	3	0	0	0	0	0
1	2	2	2	0				1	1				3	4				1	0			
3	1	3	2	0	2	3	3	1	1	1	2	2	4	4	1	1	7	0	0	1	1	0
2	2	3	6	4	3		1	3	3	2		1	4	3	7	9	3	0	1	0	0	0
2	3	3	3					2														
1	2	3	0			0		1			1		4					0				
2	3	3	0	0	1	2	1	1	1	1	1	1	5	6	7	9	1	0	0	0	0	1
1	2	3	2	2	5			1	1	3			4	5	4			0	0	0		
2	2	3	0	0	1	0		1	1	1	1		5	5	3	1		0	0	1	1	
2	3	3	0	1	1	2		1	1	1	1		4	7	7			0	0	0		
3	3	3	4	2	3			3	1	2			4	2	2	1		0	1	1	1	
2	2	3	1	1	0	2	0	1	1	1	1	1	6	7	7	8	5	0	0	0	0	0
2	2	3	0	3	1	1	0	1	2	1	1	1	4	2	5		3	0	1	0		0

2	3	3	0	1	0	1		1	1	1	1		6	7	7			0	0	0		
2	2	3	0	1	1	1		1	1	1	1		5	4	7	3		0	0	0	1	
2	3	3	0	1	3	0	2	1	1	2	1	1	6	7	7	5	4	0	0	0	0	0
2	2	3	1	0	0	1	1	1	1	1	1	1	3	4	6	6	3	1	0	0	0	0
2	2	4	2	0	0		0	1	1	1		1	3	3	1		1	1	1	1		1
3	3	3	2	1	1			1	1	1			5	4	7			0	0	0		
2	2	2	2	1				1	1				4	5			4	0	0			0
3	2	3	2		3			1		2			3					1				
2	2	3	0	1	4	4	6	1	1	3	3	3	3	5	7	8	3	1	0	0	0	0
1	1	3	0					1					3					1				
1	2	2	0					1					3					1				
2	2	3	0	1	2	3	1	1	1	1	2	1	4	4	3	2	3	0	0	1	1	0
2	2	3	2	5	4	1		1	3	3	1		5	3	4	1		0	1	0	1	
2	1	3	3	2	2			2	1	1			4	4	6			0	0	0		
2	1	4	4	1	2			3	1	1			2	4	2			1	0	1		
3	1	3	1	1	0	1		1	1	1	1		6	7	7	8		0	0	0	0	
2	2	3	0	1	0	2	0	1	1	1	1	1	5	5	6	8	4	0	0	0	0	0
2	2	3	0					1					4					0				
2	2	3	1	1	2	0	0	1	1	1	1	1	4	4	3	3	6	0	0	1	1	0
2	2	3	1	1	0			1	1	1			5	6	7			0	0	0		
2	1		0	0	0			1	1	1			6	7	7	8		0	0	0	0	
2	2	3	0	4	6			1	3	3			5	4	3			0	0	1		
2	2	3	1	2	1	1		1	1	1	1		3	6	7	5		1	0	0	0	
2	2	3	2	2	3			1	1	2			4	4	7			0	0	0		
2	2	3	3	2	0	1	1	2	1	1	1	1	5	5	7	9	5	0	0	0	0	0
2	1	3	4					3					2					1				
1	2	1	1	0	1			1	1	1			2	4	6			1	0	0		
2	1	3	3					2					5					0				
1	3	3	4		1	1		3		1	1		3		7	9		1		0	0	
1	2	3	0	3	2	2		1	2	1	1		5	6	4	4		0	0	0	0	
2	2	2	2	2	1	3		1	1	1	2		4	3	4	5	7	0	1	0	0	0
2	2	2	1		4	4		1		3	3		3		2	1		1		1	1	
2	3	3	0	1	0	0	1	1	1	1	1	1	4	5	7	7	3	0	0	0	0	0
2	2	3	1	2	0	1	0	1	1	1	1	1	4	7	7	8	6	0	0	0	0	0
2	2	3	3	6	6	4		2	3	3	3		4	2	3			0	1	1		
2	2	3	0		2			1		1			6		3			0		1		
2	1	3	1	4	0			1	3	1			4	4	6			0	0	0		
1	1	2	0	0	0	3		1	1	1	2		3	3	5	2		1	1	0	1	
2	2	3	0	1	0	1	1	1	1	1	1	1	4	4	6	3	4	0	0	0	1	0
2	3	3	1	2	2	2	2	1	1	1	1	1	3	3	4	7	3	1	1	0	0	0
2	1	3	0	2				1	1				3	5				1	0			

1	3	4	5	1	2	2	0	3	1	1	1	1	1	2	2	8	4	1	1	1	0	0
2	1	3	1	1	1	3		1	1	1	2		3	3	6	8		1	1	0	0	
1	2	1	1	2	0	1	0	1	1	1	1	1	2	6	5	5	5	1	0	0	0	0
2	2	3	2					1					3					1				
2	2	3	2	2	2	3		1	1	1	2		4	6	7	8	1	0	0	0	0	1
2	2	3	1	2				1	1				3	6				1	0			
1	2	2	0					1					4					0				
2	3	3	0	0	1	3	1	1	1	1	2	1	6	6	7	9	3	0	0	0	0	0
2	2	3	3	3	4		1	2	2	3		1	4	5	7	8	4	0	0	0	0	0
2	2	3	0	1	0	1	1	1	1	1	1	1	3	5	5	9	3	1	0	0	0	0
2	3	3	0		2			1		1			3		7			1		0		
2	3	3	0	1	0	1		1	1	1	1		4	4	6	6		0	0	0	0	
2	2	3	0					1					4					0				
2	2	2	0					1					3					1				
2	2	3	1	2	2		2	1	1	1		1	3	5	2		2	1	0	1		1
1	2	1	1	2	3	5	1	1	1	2	3	1	2	4	2	3	1	1	0	1	1	1
2	3	3	2	1	3	0	0	1	1	2	1	1	4	7	7	9	7	0	0	0	0	0
2	2	1	1	5	3	1	1	1	3	2	1	1	4	5	6	9	3	0	0	0	0	0
1	2	1	0	2	2	5		1	1	1	3			4	6	8			0	0	0	
2	2	3	1	1	0			1	1	1			4	4	7			0	0	0		
2	1	3	3	0		6		2	1		3		4					0				
2	2	3	1	2	1	2		1	1	1	1		3	5	7	7		1	0	0	0	
1	2	3	1		3	5	3	1		2	3	2	4		3	3	4	0		1	1	0
2	2	3	0	0	0	1		1	1	1	1		6	6	7			0	0	0		
2	2	3	0	3	0	2	2	1	2	1	1	1	3	4	7	4	1	1	0	0	0	1
2	1	3	1	2	3	2		1	1	2	1		3	4	3			1	0	1		
2	1	3	1	4	5			1	3	3			6	2	4	4		0	1	0	0	
2	3	3	2	1	0	1	2	1	1	1	1	1	3	4	5	6	2	1	0	0	0	1
2	3	3	0	1	2	1		1	1	1	1		6	6	7	9		0	0	0	0	
1	2	2	3	2	2	1	0	2	1	1	1	1	3	3	5	7		1	1	0	0	
2	3	3	1	0	0		0	1	1	1		1	4	4	6	9	5	0	0	0	0	0
2	2	3	2					1					3					1				
2	2	3	0	3	2	1		1	2	1	1		4		5	3		0		0	1	
2	2	3	1					1					6					0				
1	1	3	1	0	0			1	1	1			3	4				1	0			
2	2	3	0	1	1		0	1	1	1		1	5	6	5		7	0	0	0		0
2	1	2	1	2	1			1	1	1			3	5	5			1	0	0		
2	3	3	1	0	0	1		1	1	1	1		3	5	7	9	3	1	0	0	0	0
2	2	3	1	1	2	2	0	1	1	1	1	1	4	5	4	1	6	0	0	0	1	0
2	3	3	0	2	1	0	1	1	1	1	1	1	6	4	6	5	7	0	0	0	0	0
3	3	3	0	0	0			1	1	1			4	7	7			0	0	0		

1	2	3	0	1	0	2		1	1	1	1		4	5	6	8		0	0	0	0	
2	2	3	2	2	0	2	4	1	1	1	1	3	5	6	7	9	1	0	0	0	0	1
2	1	3	0	0	0	2	1	1	1	1	1	1	6	4	6	6	3	0	0	0	0	0
2	2	3	0					1					4					0				
2	2	3	0	1	3	2	0	1	1	2	1	1	3	4	4		2	1	0	0		1
1	2	3	0		0	0		1		1	1		4		7	8		0		0	0	
2	2	3	0	0	1			1	1	1			3	3	4	1		1	1	0	1	
1	2	3	1	1				1	1				3	4				1	0			
1	2	2	2		0	0		1		1	1		3		7	8		1		0	0	
2	2	3	4	7	0	0	2	3	3	1	1	1	4	3	7		7	0	1	0		0
2	2	3	1	0	2	3		1	1	1	2		4	5	2	7		0	0	1	0	
2	2	3	2	5	3			1	3	2			3	6	6	1		1	0	0	1	
1	2	2	0					1					4					0				
2	2	3	0					1					4					0				
2	2	3	2	0				1	1				5	3				0	1			
2	2	3	0	2	0	0		1	1	1	1		5	5	6	7		0	0	0	0	
2	2	3	1	0	1	5		1	1	1	3		4	3	1			0	1	1		
3	2	2	1	1				1	1				5	3				0	1			
2	2	3	0					1					6					0				
2	2	1	2					1					2					1				
1	2	3	1	4				1	3				3	7				1	0			
2	2	3	0					1					4					0				
2	3	4	3		5	2	0	2		3	1	1	4		5	7	3	0		0	0	0
2	1		0					1					3					1				
1	3	3	2	3	0			1	2	1			5	3	4			0	1	0		
2	2	3	0	2	3			1	1	2			3	4	4	2		1	0	0	1	
2	2	3	0	1	0	0		1	1	1	1		5	6	7			0	0	0		
2	2	2	1	3	1	1		1	2	1	1		3	4	5	1		1	0	0	1	
2	3	3	1	1	4	2		1	1	3	1		3	4	2	2		1	0	1	1	
2	2	3	1	2	1			1	1	1			5	6	3			0	0	1		
2	1	3	1	1	1	1	3	1	1	1	1	2	5	7	7	7	6	0	0	0	0	0
2	2	3	1	0	1	0	0	1	1	1	1	1	3	4	5	5	4	1	0	0	0	0
2	2	3	0					1					7					0				
2	2	3	0	1	0	4	0	1	1	1	3	1	6	6	7	7	5	0	0	0	0	0
1	2	1	4					3					2					1				
2	2	3	1	1	0	0	0	1	1	1	1	1		4	3		4		0	1		0
2	2	3	7					3					4					0				
2	3	3	1	1	2	1		1	1	1	1		6	4	5	6		0	0	0	0	
2	1	3	1	0	3	1	1	1	1	2	1	1	3	5	2	2	5	1	0	1	1	0
2	2	2	3	0	0			2	1	1			3	5	5			1	0	0		
2	3	3	0	1	1	1	1	1	1	1	1	1	6	7	7	4	2	0	0	0	0	1

1	2	2	0	1				1	1				3	2				1	1			
2	2	3	1	1	0	0	1	1	1	1	1	1		4	6	4	7		0	0	0	0
2	3	3	0	1	1			1	1	1			4	4	6			0	0	0		
2	2	3	2	4	5		0	1	3	3		1	4	2	6	3	6	0	1	0	1	0
1	3	1	0					1					1					1				
2	2	3	1	0	1	0	0	1	1	1	1	1	3	5	6	7	5	1	0	0	0	0
2	2	3	0		0	3		1		1	2		6		5	7		0		0	0	
2	3	3	0	1	1			1	1	1			4	4	2	1		0	0	1	1	
2	2	3	3	1	2		0	2	1	1		1	4	7	7	9	1	0	0	0	0	1
2	2	3	3		2			2		1			3		7			1		0		
2	2	2	2	1	5			1	1	3			4	5	4	1		0	0	0	1	
1	2	2	4	2	1			3	1	1			2	3	7	9		1	1	0	0	
2	3	3	1					1					3					1				
2	2	3	1	3	1	4		1	2	1	3		3	4	7	7	1	1	0	0	0	1
2	2	3	2	3	1			1	2	1			3	7	2			1	0	1		
2	2	3	0	3	1	4	6	1	2	1	3	3	5	5	7	9	3	0	0	0	0	0
2	3	3	1	3	2			1	2	1			6	7	7	7		0	0	0	0	
2	2	3	2	3	1	1		1	2	1	1		3	4	5	3		1	0	0	1	
2	1	1	0					1					2					1				
2	3	3	0					1					4					0				
1	1	3	4	1	3	2		3	1	2	1		3	4	4	7		1	0	0	0	
2	1	3	1			4		1			3		5			1		0			1	
1	2	2	2	2				1	1				3	3				1	1			
2	2	3	2	2	0	2	2	1	1	1	1	1	3	5	7	9	3	1	0	0	0	0
2	2	4	2	1	0	2		1	1	1	1		2	7	6	8		1	0	0	0	
2	2	3	0	0	0	1		1	1	1	1		4	5	7	8		0	0	0	0	
2	2	2	2	0	1			1	1	1			4	4	4			0	0	0		
2	2	3	0	0	3			1	1	2			6	7	6			0	0	0		
1	2	1	4					3					3					1				
2	2	1	0					1					4					0				
1	3	3	1					1					4					0				
2	2	4	2	0				1	1				3	2				1	1			
2	2	3	2	2	0	0	2	1	1	1	1	1	3	7	7	2	5	1	0	0	1	0
2	2	3	2	1	2	3	1	1	1	1	2	1	4	3	3	1	2	0	1	1	1	1
2	2	3	0	0				1	1				4	4				0	0			
2	2	3	1	1	0	2	0	1	1	1	1	1	4	6	7	7	2	0	0	0	0	1
2	2	2	0					1					1			1		1			1	
2	2	1	4	4	0		1	3	3	1		1	2	4	7		4	1	0	0		0
2	2	3	0	1	1	3	0	1	1	1	2	1	3	4	4	7	3	1	0	0	0	0
2	2	3	1	0	1	1	0	1	1	1	1	1	4	5	7	8	5	0	0	0	0	0
2	2	3	1	3	1	2	1	1	2	1	1	1	4	5	7	8	3	0	0	0	0	0

2	2	3	3	2	4	2	0	2	1	3	1	1	4	3	2	9	6	0	1	1	0	0
2	2	3	1					1					5					0				
2	3	3	1	2	1	0	1	1	1	1	1	1	6	7	7	9	5	0	0	0	0	0
2	2	3	0	0	5	6	2	1	1	3	3	1	3	6	4	1	1	1	0	0	1	1
2	2	3	0		1	1	0	1		1	1	1	4		7	8	4	0		0	0	0
1	1	3	0	1	5			1	1	3			6	7	4			0	0	0		
2	2	3	0	2	0	2		1	1	1	1		4	6	7	9		0	0	0	0	
2	1	3	2					1					4					0				
1	2	3	1	2	0	2	0	1	1	1	1	1	4	5	7	8	4	0	0	0	0	0
2	2	3	4	3	1			3	2	1			4	4		3		0	0		1	
2	2	3	0	1	0	3	3	1	1	1	2	2	5	4	3	6	2	0	0	1	0	1
2	2	3	0					1					3					1				
2	2	3	2	3	2	1		1	2	1	1		4	2		3		0	1		1	
2	3	3	1	0	0	1	0	1	1	1	1	1	4	5	7		7	0	0	0		0
2	3		0	2	4	0	0	1	1	3	1	1	6	2	3	5	2	0	1	1	0	1
2	3	3	1	1	4	2		1	1	3	1		7	5	2			0	0	1		
1	2	2	0					1					4					0				
2	2	3	2					1					4					0				
1	2	3	0	1	2			1	1	1			3	4	4			1	0	0		
2	3	3	4					3					5					0				
2	2	3	1	0	0	1	2	1	1	1	1	1	4	5	5	2	7	0	0	0	1	0
2	1	1	5					3					3					1				
3		3	1	1	1	2		1	1	1	1		4	5	4	3		0	0	0	1	
2	1	3	2	1	1	1		1	1	1	1		3	4	4	1		1	0	0	1	
2	2	4	1		0			1		1			2		7			1		0		
2	3	3	1	1	0	2	1	1	1	1	1	1	6	7	7	9	3	0	0	0	0	0
2	1	3	1					1					3					1				
1	1	1	1					1					2					1				
2	2	3	2	1	0			1	1	1			4	4	3			0	0	1		
1	2	1	2	2	1			1	1	1			2	3	5	2		1	1	0	1	
1	2	3	1					1					4					0				
2	2	3	0	1	0	1	1	1	1	1	1	1	4	7	7	8	1	0	0	0	0	1
3	1	4	1	0		2		1	1		1		2	2		1		1	1		1	
2	2	3	0	1	1		1	1	1	1		1	3	3	4		3	1	1	0		0
2	2	3	2					1					4					0				
2	2	3	0	0	1			1	1	1			4	7	7	2		0	0	0	1	
3	1	3	0	1	1			1	1	1			3	2	1			1	1	1		
2	2	3	0	0	1			1	1	1				4					0			
2	2	3	0	2	1	0		1	1	1	1		5	5	5	3		0	0	0	1	
2	2	3	2	2	2	1	0	1	1	1	1	1	4	4	4	5	3	0	0	0	0	0
1	2	1	1					1					4					0				

2	2	3	2	2	2	3	1	1	1	1	2	1	3	2	6	7	4	1	1	0	0	0
1	2	1	0	0	2	2	1	1	1	1	1	1	3	4	3	3	3	1	0	1	1	0
2	2	3	2	1	3			1	1	2			3	3	4	8		1	1	0	0	
2	3	1	3					2					4					0				
2	2	3	0	1	1	0	0	1	1	1	1	1	4	6	7	8	3	0	0	0	0	0
1	2	3	1	1				1	1				3	4				1	0			
2	2	1	1	2				1	1					2					1			
2	1	3	1	2	1	1	6	1	1	1	1	3	3	4	7	3	1	1	0	0	1	1
2	2	3	5					3					3					1				
2	2	3	2	1	2	3	0	1	1	1	2	1	3	3	3	2	4	1	1	1	1	0
2	1	3	4					3					2					1				
2	1	2	0		3	1	0	1		2	1	1	3		4	1		1		0	1	
2	3	3	3	3	2	1	2	2	2	1	1	1	7	7	7	9	4	0	0	0	0	0
1	1	3	2	1	2	1	3	1	1	1	1	2	3		7		7	1		0		0
1	3	1	1					1					2					1				
3	1	3	0	2	0			1	1	1			4	6	4			0	0	0		
2	2	3	0	1	0	1	2	1	1	1	1	1	4	4	5	8	3	0	0	0	0	0
2	2	3	0	4	1	1	3	1	3	1	1	2	6	3	5	4	6	0	1	0	0	0
2	3	3	2	2	1	2		1	1	1	1		3	3	4	6		1	1	0	0	
2	2	3	0					1					5					0				
2	2	3	2	1				1	1				4	5				0	0			
2	2	3	3	1	2	5		2	1	1	3		5	4	5			0	0	0		
2	3	1	3			7		2			3		5					0				
3	1	3	0					1					3					1				
2	3	3	3	2				2	1				2	3				1	1			
2	2	3	1	0	0	2		1	1	1	1		4	4	6	7		0	0	0	0	
2	2	4	7					3					2					1				
1	2	2	1	2	0	2		1	1	1	1		6	5	7	7		0	0	0	0	
1	2	3	0	1	0	2	0	1	1	1	1	1	7	4	7	9	5	0	0	0	0	0
1	2	3	1					1					4					0				
2	3	3	3	1	1	3		2	1	1	2		3	7	7			1	0	0		
2	3	3	0	4	1		2	1	3	1		1	5	4	5	6	3	0	0	0	0	0
2	2	3	2					1					3					1				
2	3	3	1	4	7	2		1	3	3	1		6	7	3	1		0	0	1	1	
2	3	3	2		0	2		1		1	1				2					1		
2	2	2	1					1					3					1				
2	2	3	1	0	1	1	0	1	1	1	1	1	6	5		2	6	0	0		1	0
2	2	3	0	1	1	1	2	1	1	1	1	1	4	3	6	6	1	0	1	0	0	1
2	2	1	3	4	6			2	3	3					2					1		
2	3	3	5	4	1			3	3	1			6	7	7	8		0	0	0	0	
2	3	3	0					1					5					0				

2	2	3	1	0	1	1		1	1	1	1		4	4	5	5		0	0	0	0	
2	2	3	1	0	1			1	1	1			5	5	7			0	0	0		
2	1	2	2					1					4					0				
2	2	3	1		1		2	1		1		1	4		5		1	0		0		1
1	2	3	1	3	4	1	1	1	2	3	1	1	5		3	1	7	0		1	1	0
2	2	3	2	1				1	1				1	3				1	1			
2	2	3	1	2	1			1	1	1			4	4	4	5		0	0	0	0	
1	2	2	0	0	2	2	2	1	1	1	1	1	4		7	3	3	0		0	1	0
2	2	3	1	1	0			1	1	1			4	3	5			0	1	0		
1	3	1	2					1					3					1				
2	3	3	4					3														
1	2	1	1					1					4					0				
2	2	1	3	3	4	2		2	2	3	1			6	4				0	0		
2	1	3	1	1				1	1				3	4				1	0			
2	2	1	1	1	1	5	4	1	1	1	3	3		2	3	6	3		1	1	0	0
2	3	3	1	1	0	0		1	1	1	1		6	7	7	9		0	0	0	0	
2	1	3	0					1					3					1				
2	2	3	0	3	0	1		1	2	1	1		4	5	7	7		0	0	0	0	
2	3	3	1	3	1	1		1	2	1	1		4	5	6			0	0	0		
1	2	3	0		2	3	2	1		1	2	1	7		7	2	5	0		0	1	0
2	3	3	0	0	1	1	1	1	1	1	1	1	5	7	7	5	7	0	0	0	0	0
2	3	1	2		1	3		1		1	2		7		7	8		0		0	0	
2	2	3	0	0	2	0	0	1	1	1	1	1	4	3	6	2	4	0	1	0	1	0
3	3	3	2	1	1	1		1	1	1	1		5	7	7	8		0	0	0	0	
1	2	3	0	1	2	2	0	1	1	1	1	1	3	5	7		3	1	0	0		0
2	2	3	1					1					4					0				
2	2	3	0	1	0	1	0	1	1	1	1	1	3	5	7	3	6	1	0	0	1	0
1	2	3	6					3					3					1				
2	2	3	1	1	1	2	7	1	1	1	1	3	3	6	4	8		1	0	0	0	
2	3	4	3					2					2					1				
2	2	3	0	1	0	0		1	1	1	1		6	7	7	9		0	0	0	0	
3	1	3	4	7	3	0	3	3	3	2	1	2	2	2	1	1	3	1	1	1	1	0
2	2	3	0	1	2	1	0	1	1	1	1	1	3	7	4	7	3	1	0	0	0	0
2	1	3	3	1	4	2	1	2	1	3	1	1	4	5	5	2	1	0	0	0	1	1
2	2	3	0	1	1	2	1	1	1	1	1	1	4	4	6	3	4	0	0	0	1	0
2	1	3	1	1	0	4		1	1	1	3		5	3	4	3		0	1	0	1	
2	2	3	0	3	0			1	2	1			4	6	7			0	0	0		
2	2	4	2					1														
2	2	3	0	1	1	1		1	1	1	1		2	4	5	1		1	0	0	1	
2	3	3	0				0	1				1	5			9	7	0			0	0
2	2	3	0	0	0	0		1	1	1	1		3	4	5	3		1	0	0	1	

2	2	3	3					2					3					1				
2	1	3	3	4	4	2		2	3	3	1		3	5	6	6		1	0	0	0	
1	2	1	3	1				2	1				1	2				1	1			
1	1	4	1	2				1	1				4	5				0	0			
2	2	3	0	1	0		0	1	1	1		1	5	4	6	8	6	0	0	0	0	0
2	2		1		1	1	0	1		1	1	1	7		7	9	7	0		0	0	0
2	3	3	0	0	4	2		1	1	3	1		4		4			0		0		
2	2	3	0					1					3					1				
2	2	2	0	1	1		0	1	1	1		1	4	6	6		2	0	0	0		1
2	1	1	1	1	2			1	1	1			2	2				1	1			
2	2	3	3	1	0			2	1	1			6	5	7			0	0	0		
2	3	3	0	2	2	2	1	1	1	1	1	1	6	7	7	9	2	0	0	0	0	1
2	2	3	0			1		1			1		4					0				
2	2	3	0	1	1			1	1	1			4		7			0		0		
3	2	3	1	1	1	3		1	1	1	2		3	4	2	2		1	0	1	1	
2	3	3	3	2	1	1	0	2	1	1	1	1	4	5	7	8	3	0	0	0	0	0
1	2	2	2	2	0	3	2	1	1	1	2	1	5	5	7	7	1	0	0	0	0	1
2	1	3	1	0				1	1				4	4				0	0			
2	2	3	2					1					3					1				
1	2	3	0		4			1		3			4		3			0		1		
2	2	3	1					1					4					0				
2	2	1	1	0	2			1	1	1			7	2	2	1		0	1	1	1	
2	2	3	1					1					4					0				
2	2	3	0	0	1	4	3	1	1	1	3	2	4	5	7	9	1	0	0	0	0	1
2	2	1	2	2	1			1	1	1			4	7	6			0	0	0		
2	2	3	2	1	2			1	1	1			4	7	7			0	0	0		
2	2	3	0	2	2		2	1	1	1		1	6	4	6		4	0	0	0		0
1	2	3	2	1	1	6		1	1	1	3		3	3	5			1	1	0		
2	2	3	1	2	0			1	1	1			4	5	7		7	0	0	0		0
2	2	3	1	2	1	1	0	1	1	1	1	1	4	2	5	7	3	0	1	0	0	0
2	1	3	2	1	0			1	1	1			5	5	6			0	0	0		
2	2	3	1	1	0	2	1	1	1	1	1	1		6	6	4	1		0	0	0	1
1	3	3	2	3	3	1		1	2	2	1		3	5	4	2		1	0	0	1	
2	1	4	0	3	1	0	0	1	2	1	1	1	3	7	7	8	3	1	0	0	0	0
2	2	3	1	2				1	1				4	5				0	0			
2	1	4	2					1					4					0				
2	2	3	0	1	0	1		1	1	1	1		5	5	5	8		0	0	0	0	
2	2	3	1	1	4	1		1	1	3	1		3	4	1	4		1	0	1	0	
2	2	3	0	0	0	1		1	1	1	1		7	7	7	8		0	0	0	0	
2	2	3	0	3	1	0		1	2	1	1		6	5	7	8		0	0	0	0	
2	3	3	2	0	0			1	1	1			3	4	7			1	0	0		

2	2	3	1					1					5					0				
1	2	3	1					1					3					1				
2	1	2	2		0			1		1			4		5			0		0		
2	3	3	0	3	0			1	2	1			4	5	4	3		0	0	0	1	
2	3	3	2	1		2		1	1		1		4	7		9		0	0		0	
1	3	3	0					1					3					1				
2	3	3	4	5	5	8		3	3	3	3		4	7	7	7		0	0	0	0	
2	2	3	0	1	1	2		1	1	1	1		3	4	4	5		1	0	0	0	
2	2	3		1					1													
2	2	3	0					1					4					0				
2	2	3	0	1	0	1	1	1	1	1	1	1	4	5	7	7	3	0	0	0	0	0
2	2	3	1	2		4		1	1		3		3	2		1		1	1		1	
2	3	3	1	4	4	3	2	1	3	3	2	1	4	4	4	2	6	0	0	0	1	0
2	2	3	1	0	1	1	1	1	1	1	1	1	1	6	3		2	1	0	1		1
2	2	3	3		0			2		1			3		7			1		0		
2	1	3	0	1	0	2	1	1	1	1	1	1	4	7	7	2	7	0	0	0	1	0
2	1	3	1	0	0			1	1	1			3	4	6			1	0	0		
2	2	3	0					1					3					1				
2	2	3	1	1	1	1	1	1	1	1	1	1	4	5	7	9	6	0	0	0	0	0
2	1	3	3					2					3					1				
2	2	3	1	2	3			1	1	2				7	5				0	0		
2	2	1	0					1														
1	1	3	1	2	1			1	1	1			3	3	7	4		1	1	0	0	
2	2	3	0	0	0	1		1	1	1	1		4	4		1		0	0		1	
2	2	2	0					1					3					1				
1	2	3	0		0			1		1			4		7			0		0		
2	2	3	1	2	1			1	1	1			5	6	4			0	0	0		
2	2	3	1	2	0	5	2	1	1	1	3	1	5	7	6	7	2	0	0	0	0	1
2	1	3	0	1	1	0	1	1	1	1	1	1	5	5	6		2	0	0	0		1
2	2	3	0	3				1	2				3	3				1	1			
2	1	3	0	3	5	4		1	2	3	3		6	5	5	4		0	0	0	0	
2	1	3	0	1	1			1	1	1			4	4	3			0	0	1		
2	3	3	1	1	2			1	1	1			4	5	3	7		0	0	1	0	
2	2	1	5	2	5			3	1	3			2	2	2	1		1	1	1	1	
2	2	3	0	1	0		1	1	1	1		1	5	7			2	0	0			1
2	1	3	1					1					3					1				
2	1	3	0					1					3					1				
2	1	3	2					1					3					1				
2	1	3	0					1					3					1				
2	3	3	2					1					3					1				
2	2	3	0	1	1	2		1	1	1	1		3	6	2	1		1	0	1	1	

2	2	4	3					2					3					1				
2	2	2	0					1					4					0				
1	1	3	1	3	3	5	4	1	2	2	3	3	4	3	4	6	2	0	1	0	0	1
2	3	3	1	0	2			1	1	1			4	5	7			0	0	0		
2	2	3	3	2	0	2	2	2	1	1	1	1	4	7	7	8	3	0	0	0	0	0
2	2	3	4	3	3			3	2	2			5	3	3			0	1	1		
2	3	3	5					3					2			8		1			0	
2	2	3	0	0	0		0	1	1	1		1	4	7	7	9	3	0	0	0	0	0
1	2	1	5	1		3		3	1		2		5	2		2		0	1		1	
2	2	3	5	2	1			3	1	1			3	4	3	4		1	0	1	0	
2	2	3	0					1					4					0				
2	2	3	0					1														
2	2	2	2		1	2		1		1	1		4		2	3		0		1	1	
1	1	1	0					1														
2	1	1	0	0				1	1				2	3				1	1			
2	3	3	0	2	2	3		1	1	1	2		6	7	7	9		0	0	0	0	
3	1	2	1	0	0	1	1	1	1	1	1	1	4	7	7	2	3	0	0	0	1	0
2	2	3	0	0	0	1	2	1	1	1	1	1	5	6	7	8	7	0	0	0	0	0
1	2	2	1					1														
2	2	1	0	0	0	1		1	1	1	1		3	4	6	6		1	0	0	0	
2	1	3	3					2					3					1				
2	2	3	4					3					3					1				
2	2	3											3					1				
1	3	3	7	6				3	3				3	3				1	1			
1	2	3	0	2	1	0		1	1	1	1		4	3	5			0	1	0		
2	2	3	2	3	3	2		1	2	2	1			4	7				0	0		
2	2	3	0	3	3	2	0	1	2	2	1	1	5	7	5	3	6	0	0	0	1	0
2	1	3	3		1	0		2		1	1				3	1				1	1	
2	3	3	2	1	1		0	1	1	1		1	4	4	6	3	7	0	0	0	1	0
2	2	3	6		3			3		2			3		4			1		0		
2	2	3	2	0		4	0	1	1		3	1	4	4				0	0			
1	2	1	0	1	0			1	1	1				4	3	3			0	1	1	
2		3			2	1				1	1				3					1		
1	2		0					1					4					0				
2	2	3	1	0	0	1	4	1	1	1	1	3	5	7	7	9	5	0	0	0	0	0
2	2	3	0		0	1	1	1		1	1	1	5		7	8	2	0		0	0	1
2	2	3	0	1	0	6		1	1	1	3		4	3	4	5		0	1	0	0	
1	2	2	0	1				1	1				3	3				1	1			
2	3	3	2	1	1	2		1	1	1	1		5	6	7	9		0	0	0	0	
2	3	3	1	1	0	0	0	1	1	1	1	1	4	4	7	7	6	0	0	0	0	0
2	2	3	3					2					3					1				

2	2	2	2	1				1	1				4	6				0	0			
1	1	1	2	0	1			1	1	1			1	3	6			1	1	0		
1	3	2	3	3	2			2	2	1			2	4	2	8		1	0	1	0	
2	2	3	1	3	3	2	1	1	2	2	1	1	4	6	1	8	3	0	0	1	0	0
2	2	3	0					1					5					0				
2	2	3	0	1	0	2	0	1	1	1	1	1	7	6	7	4	1	0	0	0	0	1
2	2	3	1					1					3					1				
2	2	2	3	1	0		2	2	1	1		1	3	2	4		5	1	1	0		0
2	3	3	1	1	1	1		1	1	1	1		6	7	7	9		0	0	0	0	
2	3	3	2	3	1		1	1	2	1		1	3	5	7		1	1	0	0		1
1	2	1	3	0	0			2	1	1			2	6	7	6		1	0	0	0	
2	2	3	0					1								3					1	
2	2	3	0	0				1	1				4	4				0	0			
2	2	3	1	0	0	0	1	1	1	1	1	1	4	5	7	8	3	0	0	0	0	0
2	1	3	1	0	1			1	1	1			4	6	7	4		0	0	0	0	
2	3	3	0	1				1	1				6	5				0	0			
1	2	3	0	2	4			1	1	3				6	3				0	1		
2	1		1	2		1		1	1		1		3			1		1			1	
2	2	3	1	2				1	1				5	4				0	0			
2	1	3	2	0	2			1	1	1				3	4	1			1	0	1	
2	2	3	0					1					3					1				
2	1	3	2	2	0	4		1	1	1	3		4	3	5	2		0	1	0	1	
2	2	3	4	3	4	2	0	3	2	3	1	1	3	3	2	1	2	1	1	1	1	1
2	2	3	1	0	1	2		1	1	1	1		4	4	7	8		0	0	0	0	
2	2	3	0	0	2	1		1	1	1	1		4	6	3	6		0	0	1	0	
2	1	3	0	2	2	1		1	1	1	1		4	5	7	6		0	0	0	0	
2	2	3	2					1					4					0				
2	2	3	4					3														
3	2	4	2	1			0	1	1			1	3	3		3	4	1	1		1	0
2	3	3	0	0	0	2	0	1	1	1	1	1	6	7	7	8	3	0	0	0	0	0
2	2	3	2	2	2	2	1	1	1	1	1	1	3	3	6	4	5	1	1	0	0	0
2	3	3	0	2	0	1		1	1	1	1		4	6	7	7		0	0	0	0	
2	3	3	2	1	1		0	1	1	1		1	5	4	7	8	6	0	0	0	0	0
1	2	3	0	1	0	3	0	1	1	1	2	1	3		7	2	6	1		0	1	0
1	1	1	1					1					3					1				
2	3	3	3		2		1	2		1		1	3		6	5	3	1		0	0	0
1	2	3	0	1	2	1	1	1	1	1	1	1	4	7	7	8	7	0	0	0	0	0
2	2	3	1	4	2	5	1	1	3	1	3	1	4	2	7	8	6	0	1	0	0	0
2	2	3	2					1														
2	3	3	0		3			1		2												
2	2	2	1					1					3					1				

2	3	3	1	1	1	2	0	1	1	1	1	1	6	5	7	8	1	0	0	0	0	1
2	2	3	1		0	0		1		1	1		4		7	8		0		0	0	
2	1	3	0	1	2	2		1	1	1	1		4	3	5	4		0	1	0	0	
2	2	2	1					1					5					0				
2	2		0		6			1		3			4		4			0		0		
2	2	3	0	1	1	2		1	1	1	1		4	3	7	3		0	1	0	1	
1	2	3	1	2	1	6	2	1	1	1	3	1	4	4	6	7	3	0	0	0	0	0
2	3	3	0					1														
1	2	3	1	3	3	4		1	2	2	3		3	7	4			1	0	0		
2	2	3	1	1	1			1	1	1			4	5	6			0	0	0		
2	1	1	2					1					5					0				
2	2	3	1					1					5					0				
2	1	3	3					2					3					1				
2	2	3	0	2	0	2	0	1	1	1	1	1	4	5	6	8	6	0	0	0	0	0
2	3	3	2	2	0	1		1	1	1	1		4	6	7	9		0	0	0	0	
1	2	1	1		0			1		1					7	9				0	0	
2	2	3	0	0	1	1	0	1	1	1	1	1	3	4	6	2	4	1	0	0	1	0
2	2	3	0	2	1	0		1	1	1	1		4	5	7	5		0	0	0	0	
1	3	1	0					1					2					1				
1	1	2	1	1		1		1	1		1											
2	2	3	0	0	0	1		1	1	1	1		4	4	6	6		0	0	0	0	
2	2	3	0	0	0	0		1	1	1	1		5	7	7	9		0	0	0	0	
2	2	3	2	1	0			1	1	1				7	7	9			0	0	0	
2	2	3	0	1	2	0		1	1	1	1		5	5	5	4		0	0	0	0	
2	2	3	2	3	3	0	0	1	2	2	1	1	3	4	5	5	4	1	0	0	0	0
2	1	3	0	2	1			1	1	1			4	6	7			0	0	0		
3	2	3	2					1					3					1				
2	2	2	1	1	0	4		1	1	1	3		6	4	6	7		0	0	0	0	
2	2	3	0	4	1			1	3	1			5	4	4	2		0	0	0	1	
2	2	3	2	2	1	2	1	1	1	1	1	1	4	5	6	8	3	0	0	0	0	0
1	1	1	2	2	4			1	1	3				2	7				1	0		
1	1	2	2					1					3					1				
2	2	3	0	0	0	0	1	1	1	1	1	1	4	4	7	7	3	0	0	0	0	0
2	2	3	1					1					5					0				
2	2	3	0		0			1		1			3		3			1		1		
2	2	1	2	0				1	1				4	3				0	1			
2	2	3	0	1	0	0	1	1	1	1	1	1	3	5	6	7	7	1	0	0	0	0
2	3	3	0	1	0		1	1	1	1		1	7	3	7		3	0	1	0		0
2	2	3	5	3	3			3	2	2			4	7	6			0	0	0		
1	2	1	4	2	1	1	2	3	1	1	1	1		7	7	8	7		0	0	0	0
2	2	3	0		4	4	1	1		3	3	1	3		1	2	3	1		1	1	0

1	2	3	2	3	1			1	2	1			3	5	6			1	0	0		
2	3	3	0	2		1	0	1	1		1	1	5	4		8	3	0	0		0	0
2	1	3	1	3				1	2				3	4				1	0			
2	2	3	6	1	1	1	4	3	1	1	1	3	3	4	3	2	1	1	0	1	1	1
1	2	2	1					1														
2	3	3	1	3	2	1		1	2	1	1		4	4	3	4		0	0	1	0	
2	1	3	3	2	2	1	0	2	1	1	1	1	4	7	5	6	7	0	0	0	0	0
2	2	3	2	0	1	2		1	1	1	1		3	5	7	7		1	0	0	0	
2	2	3	1	1	0	1		1	1	1	1		5	6	7	7		0	0	0	0	
2	3	3	0	1	1	0		1	1	1	1		4	6	7	8		0	0	0	0	
2	1	2	1	0	1			1	1	1			4	5	4			0	0	0		
2	2	3		1	1	2			1	1	1		4	5	7	9		0	0	0	0	
2	3	3	1	0	1	0		1	1	1	1		6	7	7	9		0	0	0	0	
2	3	3	5	4	2	3		3	3	1	2		3	4	5	4	4	1	0	0	0	0
2	1	1	1					1														
2	2	3	0	1	2	2	1	1	1	1	1	1	3	3	4	3	1	1	1	0	1	1
3	1	3	0	0	0	0	0	1	1	1	1	1	6	7	5	3	4	0	0	0	1	0
2	2	3	0	1	2			1	1	1			4	6	4	4		0	0	0	0	
2	2	2	1	0	2	2	1	1	1	1	1	1	4	2	3	3	7	0	1	1	1	0
2	2	3	5					3					3					1				
2	2	3	0					1					5					0				
1	1	1	3					2					7					0				
2	1	1	1	2				1	1				1	4				1	0			
2	1	2	2	1				1	1					3		2			1		1	
2	3	3	0					1					5					0				
2	2	3	2					1					5					0				
3	2	3	3	1	4	8		2	1	3	3		3	5	4	3		1	0	0	1	
2	2	3	1	0				1	1				6	5				0	0			
2	2	3	1					1					3					1				
3	2	3	1	2	3	2	4	1	1	2	1	3	4	5	2	2	3	0	0	1	1	0
2	2	2	4	0	5			3	1	3			4	4	7			0	0	0		
1	2	1	0	0	0	5	5	1	1	1	3	3		4	6	1	3		0	0	1	0
2	3	3	0	1	0	1	1	1	1	1	1	1	4	6	7	8	7	0	0	0	0	0
2	2	3	1		2	1	3	1		1	1	2	4		7	5	7	0		0	0	0
2	2	3	2					1														
2	3	1	3					2					3					1				
2	2	3	3	4	5			2	3	3			4	3	4			0	1	0		
2	3	3	1	2	5	2		1	1	3	1		5	6	3			0	0	1		
1	2	2	5		2			3		1			1		2			1		1		
2	2	3	0	1				1	1				4	7				0	0			
2	3	3	2	2	1			1	1	1			3	5	4			1	0	0		

2	1	3				1	1				1	1	4			2	4	0			1	0
2	2	3	0	2	1	1		1	1	1	1		4	4	6	2		0	0	0	1	
2	2	3	0	1	1	1	0	1	1	1	1	1	4	5	7	7	4	0	0	0	0	0
2	1	3	0	0	0			1	1	1			5		6			0		0		
2	3	3	1	1	1	2	2	1	1	1	1	1	6	6	3	4	7	0	0	1	0	0
2	2	3	1	1	1	3		1	1	1	2		3	7	6	8		1	0	0	0	
1	2	3	2	3	1	3		1	2	1	2		4	6	7	9		0	0	0	0	
1	2	1	2					1					2					1				
2	3	3	2	1	1	2		1	1	1	1		5	4	4	7		0	0	0	0	
2	2	3	1					1					4					0				
2	1	3	0	0	2			1	1	1			2	3				1	1			
2	3	3	1	1	0	0		1	1	1	1		5	5	7	7		0	0	0	0	
1	3	1	1					1					5					0				
2	2	1	2	0	0	0		1	1	1	1		5		4			0		0		
2	2	3	3	2	2		0	2	1	1		1	6	7	4	8	7	0	0	0	0	0
2	3	3	1	2	5	0		1	1	3	1		5	6	7	8		0	0	0	0	
2	2	3	1	1	1	2		1	1	1	1		5	7	7	7		0	0	0	0	
2	2	3	1	3		4	1	1	2		3	1	7	4		8	4	0	0		0	0
1	1	2	3	2				2	1				4	4				0	0			
2	2	2	1	1	1	1	0	1	1	1	1	1	5	5	7	9	4	0	0	0	0	0
2	3	3	0	1	1	1	0	1	1	1	1	1	5	6	7	4	6	0	0	0	0	0
2	1	3	0		1			1		1			4		2			0		1		
2	2	3	1	0	1			1	1	1			5	4	7	8		0	0	0	0	
2	2	3	0	1	0	0	1	1	1	1	1	1	4	4	7	7	6	0	0	0	0	0
2	2	1		1	0				1	1				2	7	4			1	0	0	
2	1	3	0		1	1	2	1		1	1	1	4		6	7	7	0		0	0	0
2	2	3	0	3	1	2	0	1	2	1	1	1	6	4	7	6	3	0	0	0	0	0
1	2	3	2		3			1		2			4		7	7		0		0	0	
1	3	2	6					3					3					1				
2	2	3	1	2	1	1	0	1	1	1	1	1	5	4	3	5	4	0	0	1	0	0
1	2	3	2	2				1	1				3	2				1	1			
2	2	3	0	1	0			1	1	1			3	3	1			1	1	1		
2	1	3	1	1	0	5		1	1	1	3		5	3	5	4		0	1	0	0	
2	2	3	0					1					3					1				
3	1	3	1	1				1	1				4	3				0	1			
1	2	1	1	1	0	0	0	1	1	1	1	1	3	5	7	8	5	1	0	0	0	0
2	1	3	2		3			1		2			4		1			0		1		
2	2	3	0	1	0			1	1	1			5	5	7	8		0	0	0	0	
2	2	3	3					2					6					0				
2	2	3	1	1	0	0		1	1	1	1		4	5	7	4		0	0	0	0	
2	3	3	1	2	3	2		1	1	2	1		6	7	7	8	7	0	0	0	0	0

1	2	1	2	1	2	1		1	1	1	1		5	4	5			0	0	0		
2		3	1	2	0	2		1	1	1	1		5	7	4	8		0	0	0	0	
1	1	1	1	2	1	2	0	1	1	1	1	1		3	7	8	2		1	0	0	1
1	1	3	0	1	1		3	1	1	1		2	3	5	7		3	1	0	0		0
2	2	3	0	1	0	1	0	1	1	1	1	1		3	3	3	6		1	1	1	0
2	2	3	2		0	1		1		1	1		4		7	9		0		0	0	
2	2	4		1	0	4	0		1	1	3	1	2	2	4	1		1	1	0	1	
2	2	3	1	1	1	1		1	1	1	1		6	5	7	8		0	0	0	0	
2	3	3	1	3	3		0	1	2	2		1	6	7	6	4	7	0	0	0	0	0
2	2	3	0	1	1	3		1	1	1	2		5	6	6	7		0	0	0	0	
2	2	3	0	1	1			1	1	1			7	7	7			0	0	0		
2	1	3	0					1					3					1				
1	1	2	2					1					3					1				
1	2	2	3	2	6	4	6	2	1	3	3	3	3	5	7	4	7	1	0	0	0	0
1	2	2	2	1	1	2		1	1	1	1		5	7	4	9		0	0	0	0	
1	3	2	2					1					4					0				
2	2	3	3	2				2	1				4	3	3			0	1	1		
1	2	3	0	3	2			1	2	1			4	5	5	2		0	0	0	1	
1	2	1	2	6	1			1	3	1			3	2	7			1	1	0		
1	2		6	0		7		3	1		3		1	2				1	1			
2	2	3	1	3	4	3	2	1	2	3	2	1	4	5	5	3	3	0	0	0	1	0
2	2	3	4					3					3					1				
2	1	3	0	0	3			1	1	2			3	5	1			1	0	1		
1	2	2	1					1					4					0				
2	3	3	0					1					4					0				
2	3	3	1	0	0			1	1	1			7	7	7		7	0	0	0		0
2	3	3	0					1					5					0				
1	2	1	6	0				3	1				5	6				0	0			
1	1	2	6		8	2		3		3	1		4		3	1		0		1	1	
2	1	3	2	0				1	1				3	2				1	1			
2	1	3	1		2	4	4	1		1	3	3	4		2	3		0		1	1	
2	2	3	1	1	0			1	1	1			4	4	5	5		0	0	0	0	
2	2	3	1	2	1	2	2	1	1	1	1	1	7	3	2	2	1	0	1	1	1	1
1	3	3	0	2	0	0	0	1	1	1	1	1	2	5	7		3	1	0	0		0
1	2	1	1	0	2	1	0	1	1	1	1	1	7	4	6	4	5	0	0	0	0	0
2	3	3	3	3	1			2	2	1			4	7	5			0	0	0		
2	2	3	0	2	2		3	1	1	1		2	4	5	6		3	0	0	0		0
2	1	4	3	4	0			2	3	1			3	2	3			1	1	1		
2	1	3	0	2	0	3		1	1	1	2		3		5	2		1		0	1	
2	2	2	3	1				2	1				2	6				1	0			
2	2	3	0	0	0	0	2	1	1	1	1	1	6	5	7	8	7	0	0	0	0	0

2	2	3	0	1	0		1	1	1	1		1	5	5	7	3	3	0	0	0	1	0
2	2	3	1	1				1	1				3					1				
2	2	3	3	0	0	0	0	2	1	1	1	1	5	6	7	8	7	0	0	0	0	0
2	2	3	1	2	0	5		1	1	1	3		5	4	7	8		0	0	0	0	
1	2	3	0	2	0	2	0	1	1	1	1	1	7	5	7	8	1	0	0	0	0	1
1	1	3	3					2					3					1				
2	2	3	1	0	1			1	1	1			6	7	7			0	0	0		
2	3	1	0	2	0	3		1	1	1	2		3	5	5	8		1	0	0	0	
2	1	3	4					3					4					0				
2	1	3	2	3	2			1	2	1			4	5	7			0	0	0		
2	2	3	3					2					3					1				
2	2	3	0	2	0	1		1	1	1	1		5	5	6	7		0	0	0	0	
2	2	4	6					3					3					1				
2	3	2	4	1	2	5	2	3	1	1	3	1	4	6	7	8		0	0	0	0	
2	2	2	1	2	0	0	0	1	1	1	1	1	4	2	6	6	6	0	1	0	0	0
2	2	3	0	2	0	2	0	1	1	1	1	1	5	6	7	9	4	0	0	0	0	0
2	3	3	0	2	3	0		1	1	2	1		4	4	5	1		0	0	0	1	
2	2	2	0	3	1		2	1	2	1		1	5	7	7		7	0	0	0		0
2	2	3	1	1	1	0	0	1	1	1	1	1	5	5	7	9	6	0	0	0	0	0
2	2	3	0	1	3		0	1	1	2		1	3	5	6	1	3	1	0	0	1	0
2	2	3	1					1					4					0				
1	3	1	0					1					4					0				
2	1	3	0		0			1		1			3		5			1		0		
1	2	3	1	1	2	1	1	1	1	1	1	1	3	4	5	1	5	1	0	0	1	0
2	2	3	1	4				1	3				4					0				
2	2	3	1	1				1	1				3	7				1	0			
2	2	3	0					1					4					0				
2	1	3	0		1	1	0	1		1	1	1	5		7	4	3	0		0	0	0
2	2	3	0	1	0	0		1	1	1	1		5	3	3	3	6	0	1	1	1	0
2	2	3	2	2	0	2		1	1	1	1		4	4	3	5		0	0	1	0	
2	2	3	0	3	0	1		1	2	1	1		4	2	6	4		0	1	0	0	
2	3	3	0	1	1	2		1	1	1	1		6	7	7	9	1	0	0	0	0	1
2	2	3	1	0	0	2		1	1	1	1		4	6	6	8		0	0	0	0	
2	2	1	3					2					1					1				
2	3	3	4	2	3	4		3	1	2	3		5	5	3	2	5	0	0	1	1	0
2	2	3	4					3					4					0				
2	3	3	2	2	3	0	1	1	1	2	1	1	3	6	6	5	5	1	0	0	0	0
2	1	3	2	1	4	7		1	1	3	3		4	4	7	3		0	0	0	1	
2	1	3	1	1	3	2		1	1	2	1		4	7	5	3		0	0	0	1	
3	1	3	0	2				1	1				4	2				0	1			
2	2	3	1	1	3	4		1	1	2	3		3	4	4			1	0	0		

2	2	3	2	0	0	0	0	1	1	1	1	1	3	6	7	7	7	1	0	0	0	0
2	2	3	0	1	2	1	0	1	1	1	1	1	5	6	5	8	6	0	0	0	0	0
3	1	3	0					1					4					0				
2	2	3	1	1	1	4		1	1	1	3		5	6	7	6	4	0	0	0	0	0
2	2	3	0	1	3	5		1	1	2	3		3	6	2	1		1	0	1	1	
3	2	3	0	1	2	1		1	1	1	1		4	4	2			0	0	1		
2	2	3	1					1					3					1				
2	2	3	1	1	0	4	1	1	1	1	3	1	3	2	4	2	4	1	1	0	1	0
2	3	2	0					1					4					0				
2	1	3	1	3	3			1	2	2			3	3	3			1	1	1		
3	1	3	0		1	6	1	1		1	3	1	3		6	4	5	1		0	0	0
2	2	2	3	5	1			2	3	1			3	2	4			1	1	0		
2	2	3	0	1	0	0	1	1	1	1	1	1	4	6	7	6	6	0	0	0	0	0
1	2	1	0	1	0			1	1	1				3	7				1	0		
2	2	3	0					1														
2	1	3	1	2	1	1	2	1	1	1	1	1	3	3	7	3	5	1	1	0	1	0
2	2	3	0	2		1		1	1		1		3	5				1	0			
2	3	3	2	3	2	3	4	1	2	1	2	3	7	5	7	6	1	0	0	0	0	1
2	2	3	0		5			1		3			6		4			0		0		
2	1	4	4		7			3		3			4		2			0		1		
2	2	3	2					1					3					1				
2	1	2	3					2					4					0				
2	2	3	0	1	0	1		1	1	1	1		6	6	7	7		0	0	0	0	
2	2	3	1	2	4	1	2	1	1	3	1	1	3	3	4	1	1	1	1	0	1	1
2	2	3	0	0	0	1	0	1	1	1	1	1	4	6	7	7	3	0	0	0	0	0
2	2	1	3					2														
2	1	2	4		6			3		3			4					0				
1	2	3	0	1	0	0	1	1	1	1	1	1	7	7	7	9	7	0	0	0	0	0
2	3	3	1	3	2		1	1	2	1		1	4	6	5		3	0	0	0		0
2	2	3	1	0	0	2	3	1	1	1	1	2	4	5	4	6	1	0	0	0	0	1
2	2	2	2					1					7					0				
2	2	2	2	1	2	1	2	1	1	1	1	1	6	4	7	8	7	0	0	0	0	0
2	3	4	5					3					2					1				
1	1	3	2					1					4					0				
2	3	3	1	0	1	4		1	1	1	3		5	5	7	1		0	0	0	1	
2	3	3	1	2	1			1	1	1			4	4	5	7		0	0	0	0	
2	2	3	2	2	2			1	1	1			4	5	6			0	0	0		
2	1	4	2					1					2					1				
1	2	3	0					1					4					0				
2	2	3	0	1	1	4		1	1	1	3		4	6	7	4		0	0	0	0	
2	3	3	1	1	0	2	0	1	1	1	1	1	6	5	7	6	7	0	0	0	0	0

2	2	3	0	1	0			1	1	1			6	7	7			0	0	0		
1	1	1	3		0			2		1			4		4			0		0		
2	3	3	0	1	0	0		1	1	1	1		5	7	7	9		0	0	0	0	
2	1	3	1	0	1	1	0	1	1	1	1	1	4	6	7	7	5	0	0	0	0	0
2	2	3	0	1	0	0		1	1	1	1		4	4	7	8		0	0	0	0	
2	2	3	1	1	0	0	0	1	1	1	1	1	3		5	7	7	1		0	0	0
2	2	3	0	2	2	1	0	1	1	1	1	1	5	3	2	1	5	0	1	1	1	0
2	2	3	1	2	0	1	0	1	1	1	1	1	4	6	7	3	6	0	0	0	1	0
2	1		4					3					1					1				
3	2	3	0	1	2	2	1	1	1	1	1	1	4	3		3	5	0	1		1	0
2	2	3	0	1	0			1	1	1			4	5	6	3		0	0	0	1	
2	1	3	0	1	2	4		1	1	1	3		4	4	3	4		0	0	1	0	
2	2	3	3	1	4	6		2	1	3	3				4					0		
2	2	3	2	1	2	1	0	1	1	1	1	1	5	7	6	8	7	0	0	0	0	0
2	2	3	4					3					3					1				
2	3	3	0	2	0	0		1	1	1	1		6	7	7	3		0	0	0	1	
1	2	3	0					1					3					1				
1	2	3	1	6	1			1	3	1			4	7	5			0	0	0		
2	3	3	1	2	2	0	0	1	1	1	1	1	5	6	4	6	3	0	0	0	0	0
3	3	3	0	0	2	1		1	1	1	1		4	5	4			0	0	0		
3	1	3	1	3	0	2	3	1	2	1	1	2	4	3	2		1	0	1	1		1
2	3	3	0					1														
2	1	3	0	0				1	1				3					1				
2	2	3	0	1	2	0	2	1	1	1	1	1		7	7	8	6		0	0	0	0
1	2	2	1	1	3			1	1	2			4	7	2			0	0	1		
2	2	3	0	0	1	3	0	1	1	1	2	1	4	4	3	3	3	0	0	1	1	0
2	2	2	2					1					6					0				
2	2	3	0	0	2	2		1	1	1	1			4	2				0	1		
2	2	2	1	2	6	1	2	1	1	3	1	1	2	7	5		5	1	0	0		0
2	3	3	1	1	0	1	1	1	1	1	1	1	5	7	7	8	6	0	0	0	0	0
2	2	3	0	0	1			1	1	1			4	6	4	8	7	0	0	0	0	0
2	2	1	1					1					4					0				
2	2	3	1	2	1	1	2	1	1	1	1	1	5	2	6	8	4	0	1	0	0	0
2	1	3	1	0				1	1				6	4		4		0	0		0	
1	2	2	0	0	0	4		1	1	1	3		5	5	7	9		0	0	0	0	
2	3	3	1	6				1	3				5	2				0	1			
1	2	3	1	2	1	5	6	1	1	1	3	3	4	5	7	3	1	0	0	0	1	1
2	1	3	4		7	3		3		3	2		4		7			0		0		
2	3	3	2	2	1	2		1	1	1	1		4	5	4	7		0	0	0	0	
2	2	3	0	1	1	0	3	1	1	1	1	2	5	5	7	8	5	0	0	0	0	0
1	1	1	1	1	0			1	1	1				5	7				0	0		

3	1	3	4					3					3					1				
2	2	2	1					1					7					0				
1	3	3	4					3					3					1				
2	2	3	1	2	1	1	4	1	1	1	1	3	4	4	6	7	2	0	0	0	0	1
2	1	2	3	1	2	1	3	2	1	1	1	2	3	2	3	2	1	1	1	1	1	1
2	1	3	3					2					3					1				
2	2	3	2	2	1	3	1	1	1	1	2	1	3	6	6	7	5	1	0	0	0	0
2	3	3	4	4	0	5		3	3	1	3		5	7	7	9		0	0	0	0	
2	3	3	2					1					4					0				
2	3	2	0	4	2	2	1	1	3	1	1	1	4	7	7		2	0	0	0		1
1	2	3	1					1					3					1				
2	2	3	0	2	0	0		1	1	1	1		3	4	6	8		1	0	0	0	
2	2	3	2	0	0	1	0	1	1	1	1	1	4	6	6	9	7	0	0	0	0	0
2	2	2	1	2	1	2		1	1	1	1		6	5	7	8		0	0	0	0	
2	3	3	1	0				1	1				6	5				0	0			
1	2	1	1	2	0			1	1	1			5	7	7			0	0	0		
3	1	1	0		0		0	1		1		1	3		1	2		1		1	1	
2	2	3	1	2	0	2	0	1	1	1	1	1	6	7	7	8	7	0	0	0	0	0
2	1	2	2		0	0	3	1		1	1	2	4		7	8	1	0		0	0	1
2	2	3	0	0		2		1	1		1		4					0				
2	2		5	1	2			3	1	1			2	4	4			1	0	0		
2	2	3	0		3	1		1		2	1		3		6	1		1		0	1	
3	3	3	1			2		1			1		4			2		0			1	
2	2	2	0	0	1	4		1	1	1	3		3	6	7	6		1	0	0	0	
1	2	1	4	1	1			3	1	1			5	7	3			0	0	1		
2	2	2	4	6				3	3				3	3				1	1			
2	3	3	2					1					4					0				
1	2	3	1	3	4	2	2	1	2	3	1	1	4	4	6	6	6	0	0	0	0	0
2	2	3	1					1					7					0				
1	2	1	0	0	1	0		1	1	1	1		4	4	6	4		0	0	0	0	
1	2	1	1		3	0	0	1		2	1	1	3		2	8	5	1		1	0	0
1	2	1	1					1					4					0				
2	1	3	1	0	1		3	1	1	1		2	3	7	4		2	1	0	0		1
2	3	3	0	1	0	0	8	1	1	1	1	3	5	6	7	8	6	0	0	0	0	0
2	1	2	3	3	4			2	2	3			3	4	3			1	0	1		
2	2	3	0	1	0		1	1	1	1		1	6	5	6	8	3	0	0	0	0	0
2	3	3	0		0			1		1			4		7			0		0		
2	2	3	0	5	1	2		1	3	1	1		3	3	5	7		1	1	0	0	
2	1	3	1	1	1			1	1	1			3	3	3			1	1	1		
1	2	3	1		0	4	1	1		1	3	1	5		7	5	3	0		0	0	0
1	2	1	1	1	0			1	1	1			2	3	7	8		1	1	0	0	

2	3	3	1	1	0	0	0	1	1	1	1	1	5	5	7	8	7	0	0	0	0	0
2	1	3	1	4	2	2	4	1	3	1	1	3	4	3	2	1	1	0	1	1	1	1
1	2	1	0	2	1	1	0	1	1	1	1	1	2	7	7	6	4	1	0	0	0	0
1	1	1	4	1	1			3	1	1			2	5	5			1	0	0		
2	1	1	3	2	2			2	1	1			3	3	3	6		1	1	1	0	
2	2	3	1		0			1		1			5		6			0		0		
1	2	2	1					1					3					1				
2	2	3	1					1					5					0				
2	1	3	0	2	1			1	1	1			3	4	7			1	0	0		
1	3	1	2	2	3	4	3	1	1	2	3	2	4	4	3	5	3	0	0	1	0	0
3	2	3	1		0	1		1		1	1		5		5			0		0		
2	2	3	1	2	4	4		1	1	3	3		4	4		2		0	0		1	
3	1	3	3		0			2		1			2		2			1		1		
2	1	3	1	4				1	3				4	3				0	1			
2	2	4	0	1	1	2		1	1	1	1		3	4	4	4		1	0	0	0	
2	1	3	4	2		3		3	1		2		4	2		1		0	1		1	
2	2	3	4	1	1			3	1	1			3	3	3			1	1	1		
1	2	2	1	2	4			1	1	3			4	2	2			0	1	1		
2	1	3	0	1				1	1				4	2				0	1			
3	1	3	0					1					4					0				
2	1	3	4	2				3	1				3	5		5		1	0		0	
2	2	3	0	1	1		1	1	1	1		1	5	4	6	1	1	0	0	0	1	1
2	2	3	1					1					3					1				
2	2	3	0	0	3	2	1	1	1	2	1	1	4	3	4	2	3	0	1	0	1	0
1		2	3	4	4	1	1	2	3	3	1	1	4	6	7	5	7	0	0	0	0	0
3	1	3	0	1	1	0	0	1	1	1	1	1	5	3	4	2	3	0	1	0	1	0
2	2	3	1					1					4					0				
2	1	3	1	4		1	0	1	3		1	1	5	6		6	3	0	0		0	0
2	2	3	0	0	0	2	1	1	1	1	1	1	5	7	7	8	3	0	0	0	0	0
2	2	3	3				0	2				1	6			1	1	0			1	1
2	2	3	0					1					4					0				
2	3	3	1	2	2	3	2	1	1	1	2	1	4	6	6	9	6	0	0	0	0	0
2	2	3	0					1					3					1				
2	2	3	4	1	4	4	1	3	1	3	3	1	2	5	6	7	3	1	0	0	0	0
2	2	3	0	4	4			1	3	3			4	3	2			0	1	1		
2	2	2	1					1					5					0				
2	2	3	3	2	0	3		2	1	1	2		4	5	7	9		0	0	0	0	
2	3	3	3	0	0	2		2	1	1	1		6	7	7	7		0	0	0	0	
2	2	3	0	0	0			1	1	1			5	5	6	8		0	0	0	0	
2	2	3	1	1	2	1		1	1	1	1		4	5	7	5		0	0	0	0	
2	3	3	0	3	2	3	0	1	2	1	2	1	3	4	6	5	3	1	0	0	0	0

2	2	3	0	1	2			1	1	1			6	4	7			0	0	0		
2	3	3	0	1	0			1	1	1			5	7	7	9		0	0	0	0	
2	2	3	0	1	0	1		1	1	1	1		4	7	7			0	0	0		
2	1	3	0	2	3	4		1	1	2	3		6	3	4	5	1	0	1	0	0	1
2	2	3	3	1	0			2	1	1			5	5	5	7		0	0	0	0	
2	2	3	1	1	3	0	0	1	1	2	1	1	4	6	2	1	7	0	0	1	1	0
2	2	3	3	1	0	2	0	2	1	1	1	1	4	4	6	6	5	0	0	0	0	0
2	1	3	1	1	0	2	0	1	1	1	1	1	5	5	6	5	5	0	0	0	0	0
2	2	3	1	1	0	3		1	1	1	2		5	7	7	9		0	0	0	0	
2	3	2	1	2				1	1				4	3		1		0	1		1	
2	1	3	2					1					3					1				
2	3	3	0	1	0			1	1	1			5	5	5			0	0	0		
2		3	0	0	0	0		1	1	1	1		4	5	5	4		0	0	0	0	
2	3	3	2	1	2			1	1	1			6	7	7	8		0	0	0	0	
2	1	3	0	1	1			1	1	1			5	5	7			0	0	0		
1	2	2	3					2					4					0				
1	1	3	0	0	2		1	1	1	1		1	3	3	2		5	1	1	1		0
3	1	3	0	0	2	2		1	1	1	1		5		3	1		0		1	1	
2	1	3	0		1	6		1		1	3		3		2	3		1		1	1	
2	3	1	5	2	4			3	1	3			5	3	3	1		0	1	1	1	
2	3	3	0	1	1	2	0	1	1	1	1	1	6	7	7	9	4	0	0	0	0	0
2	2	3	4	1	1	2		3	1	1	1		4	6	5	6		0	0	0	0	
2	2	3	0	0				1	1				4	7				0	0			
2	2	3	0	0	0	0	0	1	1	1	1	1	4	5	4		5	0	0	0		0
2	2	2	1					1					4					0				
2	3	3	2	2	1	2	1	1	1	1	1	1	6	7	7	9	7	0	0	0	0	0
2	2	3	0	1	3			1	1	2			3	4	6	8		1	0	0	0	
2	2	3	1	2				1	1				4	6		8		0	0		0	
2	2	3	0	4	2			1	3	1			4	7	3			0	0	1		
1	3	3	1	3	1			1	2	1			7	3	3			0	1	1		
2	3	1	3	2				2	1				4	7				0	0			
2	2	3	4	2	1	2	1	3	1	1	1	1	4	6	6	7	2	0	0	0	0	1
2	2	3	3	0	1	1		2	1	1	1		3	4	4	5		1	0	0	0	
2	1	3	1	0	0			1	1	1			3	4	6			1	0	0		
2	2	3	0		1		2	1		1		1	4		7		3	0		0		0
2	2	3	0	0	1	4	2	1	1	1	3	1	3	6	7	2	3	1	0	0	1	0
1	2	3	2					1					5					0				
1	2	3	0		1	5		1		1	3				3	4				1	0	
2	2	3	0	1	2			1	1	1			4	7		9		0	0		0	
1	2	2	7					3					3					1				
1	2	3	0	0				1	1				4	3				0	1			

1	2	2	0					1					4					0				
2	3	3	2		2	4	1	1		1	3	1	4		5	4	7	0		0	0	0
2	1	3	0	0	0			1	1	1			4	5	6			0	0	0		
2	2	2	4	2	2	2		3	1	1	1		3	5	6	1	3	1	0	0	1	0
2	1	3	0	0	0	2		1	1	1	1		3	5	7	2		1	0	0	1	
2	2	3	1		0	0		1		1	1		3		4	4		1		0	0	
2	3	3	0	3		1		1	2		1		5	5				0	0			
2	3		1	4	0	4	2	1	3	1	3	1	4	5	4		6	0	0	0		0
1	2	2	0	2	1	4		1	1	1	3		4	5	5	3		0	0	0	1	
2	3	3	2	0	0	1	0	1	1	1	1	1	3	4	6	7	5	1	0	0	0	0
2	2	3	1	1	1	2	1	1	1	1	1	1	4	5	7	4	1	0	0	0	0	1
1	2	1	0	2	6	1	5	1	1	3	1	3	5	3	3		3	0	1	1		0
2	3	3	2					1					5					0				
2	1	3	1	1	2	0	0	1	1	1	1	1	5	5	7	8	6	0	0	0	0	0
2	2	3	1	2	2			1	1	1			3	6	6			1	0	0		
2	2	3	1	1	1	1		1	1	1	1		3	5	5	4		1	0	0	0	
2	2	3	2	0	2			1	1	1			3	1	1			1	1	1		
1	1	2	2					1					3					1				
2	2	3	1	1		0		1	1		1		5	4		7		0	0		0	
2	2	3	0	1	0	2	2	1	1	1	1	1	5	3	7	9	1	0	1	0	0	1
2	1	1	5					3					3					1				
2			1					1														
2	2	3	0	2	3			1	1	2			4	7	6	7		0	0	0	0	
2	3	3	3	1	0			2	1	1			3	2	5	3		1	1	0	1	
1	2	1	2	2	3			1	1	2			7	3	5			0	1	0		
2	2	3	2					1					3					1				
2	2	3	1	2	1		2	1	1	1		1	5	6	3	2	1	0	0	1	1	1
2	2	3	1	3	2			1	2	1			4	6	7	9		0	0	0	0	
2	2	3		0	0				1	1				4	3	2			0	1	1	
2	2	3	0	2	1	1		1	1	1	1		4	4	7	7		0	0	0	0	
1	1	3	2	2	1	0	3	1	1	1	1	2	4	3	5	3	3	0	1	0	1	0
2	2	4	5					3														
2	2	3	2					1					5					0				
3	1	4	2	1	1			1	1	1			3	2	7			1	1	0		
2	1	2	3	3	6	4		2	2	3	3		4	4	2	1		0	0	1	1	
2	3	3	0					1					5					0				
2	1	3	1					1					5					0				
2	2	3	0	4	2			1	3	1			4	5	5	3		0	0	0	1	
2	2	3	0	3	1	3		1	2	1	2		4	4	3	5		0	0	1	0	
2	2	3	0	3	1			1	2	1			3	4	6			1	0	0		
2	2	3	3	2	2			2	1	1			4	6	5	9		0	0	0	0	

2	2	3	2	1	0	1		1	1	1	1		4					0				
2	2	3	2	3	2	2	3	1	2	1	1	2	5	6	5	5	1	0	0	0	0	1
2	2	3	0	0	0	0	0	1	1	1	1	1	3	3	6	5	6	1	1	0	0	0
2	1	3	2					1					3					1				
2	1	3	3	2	4	2		2	1	3	1		4	4	2	7		0	0	1	0	
1	1	3	2					1					3					1				
1	3	2	3					2					6					0				
2	3	3	0	0	3	0	0	1	1	2	1	1	4	7	6	9	3	0	0	0	0	0
1	3	3	0	1	0	4	1	1	1	1	3	1	5	5	4	1	4	0	0	0	1	0
2	1	3	1	1	1	3	1	1	1	1	2	1	6	3			7	0	1			0
2	2	3	1		3	2		1		2	1		5		4			0		0		
2	2	2	2	3	3			1	2	2			6	7	7			0	0	0		
2	2	2	0	0			3	1	1			2	4	5		1	1	0	0		1	1
3	2	3	1		2		1	1		1		1	5		3	2	1	0		1	1	1
1	2	1	1	1	2			1	1	1			3	2	2			1	1	1		
2	2	3	3	1	0	0	1	2	1	1	1	1	5	7	7		4	0	0	0		0
2	1	3	0		2	0	3	1		1	1	2	5		2		5	0		1		0
2	2	3	1	1	0	2	0	1	1	1	1	1	7	7	6	7	7	0	0	0	0	0
3	1	3	1	2	0	2		1	1	1	1		3	6	5	5		1	0	0	0	
2	2	3	1	3	2	3	1	1	2	1	2	1	3	2	2	4	7	1	1	1	0	0
2	2	3	4				2	3				1	5				4	0				0
2	2	3	3					2					3					1				
2	2	3	2	0	3			1	1	2			4	5	2	3		0	0	1	1	
2	2	3	1	0	3			1	1	2			3	4	3			1	0	1		
3	2	3	1		2	1		1		1	1		4		4	2		0		0	1	
2	2	3			2		2			1		1	5		2	1		0		1	1	
2	2	3	0	1	0			1	1	1			4	6	6			0	0	0		
2	3	3	2	2	2			1	1	1			4	5	5	5		0	0	0	0	
2	2	3	0		0		1	1		1		1	4		4		5	0		0		0
2	2	3	0					1					4					0				
2	3	3	0	0	0	3		1	1	1	2		3	3	7	7		1	1	0	0	
2	2	2	1	0	3		1	1	1	2		1	6	6	7	4	4	0	0	0	0	0
2	2	2	3	2	2		0	2	1	1		1	5	6	3	6	7	0	0	1	0	0
2	2	3	1	2	3	0	0	1	1	2	1	1	4	5	7	1	6	0	0	0	1	0
3	1	3	1	1	0	0	0	1	1	1	1	1	3	7		5	3	1	0		0	0
2	2	3	0					1					5					0				
2	2	3	0	3	2	3		1	2	1	2		4	5	4	4		0	0	0	0	
2	2	3	0	0	0	1		1	1	1	1		4	6	4	1		0	0	0	1	
2	2	3	0	0	1	1	0	1	1	1	1	1	5	7	5	6	6	0	0	0	0	0
2	1	3	0		1	8		1		1	3		4		2	2		0		1	1	
2	2	3	3					2					4					0				

2	2	3		3	3	4	2		2	2	3	1	4	5	5	8	1	0	0	0	0	1
2	2	3	0	1	1	2		1	1	1	1		6	2	3			0	1	1		
2	2	3	1	0				1	1				4	5				0	0			
2	2	3	3		0			2		1			4		5	2		0		0	1	
3	1	3											3					1				
2	2	3	3	3	0			2	2	1			4	4	6			0	0	0		
2	1	3	3		1	1		2		1	1		4		4	4		0		0	0	
2	2	2	2					1					5					0				
2	2	3	2	2	0	2		1	1	1	1		4	5	7			0	0	0		
2	2	3	0	0	0			1	1	1			5	6	5			0	0	0		
3	2	3	0					1					7					0				
2	2	3	6		0	1		3		1	1		3		7	4		1		0	0	
2	3	3	4		1	2		3		1	1		6		7	8		0		0	0	
2	3	3	3	1				2	1				6	7				0	0			
2	2	2	0	1	2			1	1	1			6	5	7			0	0	0		
2	2	3	0	1	0	0	0	1	1	1	1	1	3	7	7		7	1	0	0		0
2	2	3	1	0				1	1													
2	2	3	0	1				1	1				4	5				0	0			
2	2	3	4					3					3					1				
2	2	3	1	0	0	0	1	1	1	1	1	1	6	6	7	9	6	0	0	0	0	0
3	3	3	0	1	0	0		1	1	1	1		4	4	7	2		0	0	0	1	
2	2	3	1	1	0	1	0	1	1	1	1	1	4		6	6	4	0		0	0	0
2	2	3	1	3	2	3	2	1	2	1	2	1	6	5	4	3	1	0	0	0	1	1
2	1	3	2	2	3			1	1	2			1	5	6			1	0	0		
1	1	3	0		2			1		1			3		2			1		1		
1	2	3	1	1	1			1	1	1			4	4	4	7		0	0	0	0	
2	2	1	1	0	2	1		1	1	1	1		4	4	7	8		0	0	0	0	
2	1	3	0	1				1	1				4	7				0	0			
2	3	3	1	3	2	4		1	2	1	3		3	3	4	2		1	1	0	1	
1	1	3	4	3				3	2				1	3				1	1			
3	2	3	1					1					3					1				
2	2	4	2	1	5	2	0	1	1	3	1	1	2	5	3		7	1	0	1		0
2	2	3	0	1	0			1	1	1			4	5	7			0	0	0		
1	3	1	2					1					6					0				
3	1	2	1		5			1		3			3		4			1		0		
2	3	3	1	1	1	1		1	1	1	1		6	4	6	8		0	0	0	0	
2	1	3	3	2	4			2	1	3			3	3	2			1	1	1		
2	2	3	3					2					4					0				
2	1	3	1	4	4	3		1	3	3	2		4	4	4	7		0	0	0	0	
2	1	2	1		3			1		2					2					1		
2	2	3	0	0	0	1		1	1	1	1		5	7	7	4		0	0	0	0	

1	2	3	1	2	1	6		1	1	1	3		3	4	5	3		1	0	0	1	
2	2	3	2	1	1	1		1	1	1	1		4	5	7	8		0	0	0	0	
1	2	3	2	3	1	2	0	1	2	1	1	1	5	4	6		1	0	0	0		1
2	3	3	2	3	0	2		1	2	1	1		5	5	6	7		0	0	0	0	
1	2	3	4		0			3		1			4		7			0		0		
2	2	3	0					1					4					0				
2	2	1	2	2		4		1	1		3		2	2		6		1	1		0	
2	2	3	0	0				1	1				3	2				1	1			
2	2	3	0					1					4					0				
1	2	3	0	1		2		1	1		1		4	7		6		0	0		0	
2	2	3	3	1	2			2	1	1			4	4	5			0	0	0		
2	2	3	3	1	1	0	0	2	1	1	1	1	5	5	5	5	7	0	0	0	0	0
2	1	3	1	3				1	2				3	5				1	0			
3	2	3	2					1					4					0				
2	2	1	0	4				1	3				3	2			3	1	1			0
2	3	2	5	6	2	2	0	3	3	1	1	1	5	4	4	6	5	0	0	0	0	0
2	1	3	1		1	2		1		1	1		3		2	1		1		1	1	
2	2	3	0		1	2	1	1		1	1	1	5		6	6	6	0		0	0	0
1	3		2	4	0	2		1	3	1	1		4	6	7	1		0	0	0	1	
2	2	3	0	0	1		0	1	1	1		1	4	4	4			0	0	0		
2	2	3	1	1	3			1	1	2			4	4	4			0	0	0		
2	2	3	0					1					4					0				
2	2	3	0			1	2	1			1	1	5			9	2	0			0	1
2	3	3	0	0	0	1	4	1	1	1	1	3	5	4	7	9	5	0	0	0	0	0
2	2	3	0	3	1	1		1	2	1	1		4	3	5	1		0	1	0	1	
2	2	3	0	0	0	2	0	1	1	1	1	1	3	5	7	8	5	1	0	0	0	0
2	2	3	0		1	1	1	1		1	1	1			5	7	7			0	0	0
1	2	3	2	1	1	1		1	1	1	1		4	4	5	5		0	0	0	0	
2	1	3		0	0				1	1			4	5	5	6		0	0	0	0	
2	2	3	0	1	0	2	1	1	1	1	1	1	5	5	3	4	6	0	0	1	0	0
2	3	3	0	3	1	0		1	2	1	1		5		7	7		0		0	0	
2	2	3	1	0	1	2	0	1	1	1	1	1	4	6	7	8	1	0	0	0	0	1
2	1	3	0	0	0	2		1	1	1	1		3	4	5	5		1	0	0	0	
1	1	3	1	1	0	1		1	1	1	1		4	4	7	8		0	0	0	0	
2	1	2	2			3		1			2		4			1		0			1	
2	2	3	0	1				1	1				5	3				0	1			
2	2		0		2			1		1			4		2			0		1		
2	3		7					3					6					0				
2	2	3	1					1					5					0				
1	2	1	1	3	0			1	2	1			4	2	7	5		0	1	0	0	
2	2	3	1	2	1			1	1	1			4	4	5			0	0	0		

2	2	3	1	2	2	1	1	1	1	1	1	1	5		6	7	7	0		0	0	0
1	2	3			4					3					5					0		
1	1	3	2					1					4					0				
2	2	3	2	6	2		7	1	3	1		3	5	5	7		6	0	0	0		0
2	2	1	1			2		1			1											
2	2	3	0	2	1			1	1	1			4	6	3	3		0	0	1	1	
2	2	3	0	2	0			1	1	1			5	5				0	0			
1	2	1	3	1	0	2	3	2	1	1	1	2	4	4		4	1	0	0		0	1
2	2	2	2	0	0	1		1	1	1	1		4	5	7			0	0	0		
2	2	3	0	2	0	1		1	1	1	1		5	2	2	1		0	1	1	1	
2	3	3	0	2	1	0		1	1	1	1		7	6	7	7		0	0	0	0	
2	3	3	0	2	2	0		1	1	1	1		4	6	3	4		0	0	1	0	
2	2	3	4					3					7					0				
2	1	3	0					1					6					0				
1	2	2	5	4	2	1		3	3	1	1		3	4	3			1	0	1		
2		3	1	2	2	0	4	1	1	1	1	3	4	5	7	9	2	0	0	0	0	1
2	3	3	2	1	3			1	1	2			5	7	4			0	0	0		
2	2	3	0		0	1		1		1	1		4		1	1		0		1	1	
2	3	3	1	1	0	2		1	1	1	1		6	7	5	5		0	0	0	0	
2	1	3		2	3	0	1		1	2	1	1	3	3	3	2	5	1	1	1	1	0
2	2	3	0	0	0	3		1	1	1	2		5	6	7	8		0	0	0	0	
2	2	3	1					1					3					1				
2	2	3	0	0	3	1	0	1	1	2	1	1	6	6	4	7	5	0	0	0	0	0
2	1	3	1	3	2			1	2	1			6	3	7			0	1	0		
2	2	3	1	0				1	1				3	5				1	0			
2	3	3	0					1					6					0				
2	1	3	1	0	1	1		1	1	1	1		3	4	4	2		1	0	0	1	
2	2	3	2					1					3					1				
2	3	3	2	3	3	2		1	2	2	1		4	6	6	9		0	0	0	0	
2	3	3	1	0				1	1					4					0			
2	3	3	3					2					5					0				
2	2	2	1	1	0	0	0	1	1	1	1	1	4	4	6	4	5	0	0	0	0	0
2	1	3	1	3	1	2	2	1	2	1	1	1	4	6	7		7	0	0	0		0
2	1	1	2					1					3					1				
2	3	3	1	1	1	3		1	1	1	2		5	3	7	3		0	1	0	1	
2	2	3	3	1	1	2		2	1	1	1		4	4	7	8	4	0	0	0	0	0
2	1	4	2			3		1			2		7			3		0			1	
2	2	2	0	0				1	1				3	3				1	1			
2	1	3	1	0	1	1		1	1	1	1		4	4	5			0	0	0		
2	1	3	0	1	1	0	2	1	1	1	1	1	3	6	7	7	3	1	0	0	0	0
2	2	2	5	3	2			3	2	1			4	6	5			0	0	0		

3	1	3	0	0	0	4	2	1	1	1	3	1		5	7		3		0	0		0
2	2	3	0	3	4	1	0	1	2	3	1	1	4	4	7	8	1	0	0	0	0	1
2	2	3	1					1					4					0				
2	3	3		1	1				1	1			4	5	7	7		0	0	0	0	
2	1	3	0	1	3			1	1	2			4	7	4			0	0	0		
2	2	3	3	3	3		1	2	2	2		1	3	4	3		4	1	0	1		0
2	2	2	0					1					5					0				
2	1	1	3					2					2					1				
2	3	3	1	3	2	0	1	1	2	1	1	1	4	5	7	9	3	0	0	0	0	0
2	2	3	0	1	0	1		1	1	1	1		4	5	7	8		0	0	0	0	
2	2	3	0	1	0	2		1	1	1	1		7	5	6	6		0	0	0	0	
2	1	3	0	0	0	0	0	1	1	1	1	1	4	6	4	8	6	0	0	0	0	0
2	1	3	1					1					3					1				
2	2	3	0	3	3			1	2	2			4	3	4	7		0	1	0	0	
2	2	3	3	2	1	4	3	2	1	1	3	2	3	3	4	7	1	1	1	0	0	1
2	2	1	2	3	4	2		1	2	3	1		2	2	5			1	1	0		
2	2	3	4	5				3	3				4	6				0	0			
2	2	2	1	2	1	4		1	1	1	3		4	3	5	1		0	1	0	1	
2	2	3	0	3	2			1	2	1			5	7	7			0	0	0		
2	3	3	1	0	4	2		1	1	3	1		5		5	2		0		0	1	
2	1	2	0	1	0	2		1	1	1	1		3	4	5	2		1	0	0	1	
2	2	3	1	1	0	1	0	1	1	1	1	1	5	5	3	6	4	0	0	1	0	0
2	2	3	2					1					4					0				
2	1	3	0	0	0			1	1	1			3	4		2		1	0		1	
2	2	3	1	1	1	3	2	1	1	1	2	1	5	7	7	9	1	0	0	0	0	1
2	2	2	4	1				3	1				2	4				1	0			
2	2	3	5	2	3		1	3	1	2		1	4	6	2		1	0	0	1		1
2	3	1	2	4	1	3	0	1	3	1	2	1	3	5	7	8	5	1	0	0	0	0
2	2	2	1	3	1		2	1	2	1		1	5	7	7	9	4	0	0	0	0	0
2	2	3	0	2	1			1	1	1				4	2	8			0	1	0	
2	2	3	0	1	0	2	1	1	1	1	1	1	4	4	2	1	1	0	0	1	1	1
2	2	1	0					1					4					0				
2	3	3	4	2	0	7	2	3	1	1	3	1	5	5	7	9	5	0	0	0	0	0
2	2	3		1	0	0	3		1	1	1	2	3	6	7		3	1	0	0		0
1	2	3	1					1					5					0				
2	3	3	1	1				1	1				4	3				0	1			
2	2	3	0	0	0			1	1	1			5	5	7			0	0	0		
2	2	3	1					1					4					0				
2	1	3		1	0				1	1			3	4	5			1	0	0		
2	2	3	0	4	7		0	1	3	3		1	4	5	3		4	0	0	1		0
2	2	3	1	3	2			1	2	1			3	2	2			1	1	1		

2	1	3	4	2	1	2		3	1	1	1		5		3	3		0		1	1	
2	1	2	0	3	5	3	2	1	2	3	2	1	1	3	3	1	3	1	1	1	1	0
2	2	1	0					1					3					1				
2	2	3	2					1					3					1				
2	3	3	4		1			3		1			4		6			0		0		
2	2	3	0		0			1		1			6		7			0		0		
2	2	3	1	1	0	6		1	1	1	3		4	5	6	7		0	0	0	0	
2	2	2	1	3	1			1	2	1			4	4	3			0	0	1		
2	2	3	2	1	2	0		1	1	1	1		4	3	7	9		0	1	0	0	
2	2	3	1	1	1	0	0	1	1	1	1	1	6	7	7	7	1	0	0	0	0	1
2	3		3	1	0	0	0	2	1	1	1	1	5	6	7	7	4	0	0	0	0	0
2	1	3	0	4	2	1	1	1	3	1	1	1	4	4	7	6	5	0	0	0	0	0
3	2	3	1		0			1		1			3		5			1		0		
2	3	3	2	1	0	1	2	1	1	1	1	1	6	7	7	8	1	0	0	0	0	1
2	2	2	0	0	5	0	0	1	1	3	1	1	3	4	7	8	6	1	0	0	0	0
2	2	3	0	0	2	0		1	1	1	1		4	5	6	4		0	0	0	0	
2	2	3	1	2	3	1	0	1	1	2	1	1	7	7	7	9	3	0	0	0	0	0
2	1	3	1	1	0	0		1	1	1	1		5	6	7	9		0	0	0	0	
2	2	3	1	3	0	2	0	1	2	1	1	1	6	4	7	3	5	0	0	0	1	0
2	3	2	1	3	1	4	1	1	2	1	3	1	5	7	7	3	4	0	0	0	1	0
2	2	2	3				0	2				1					3					0
2	2	3	4					3					5					0				
2	2	3	0	0	0	2		1	1	1	1		5	6	7	8		0	0	0	0	
2	3	3	1	1	3	2	2	1	1	2	1	1	5	6	5	5	4	0	0	0	0	0
2	2	3	1		4	0	1	1		3	1	1	3		7	7	2	1		0	0	1
2	2	4	0					1					4					0				
2	2	3	0		1	2		1		1	1		4		5	7		0		0	0	
2	3	1	4	5	2			3	3	1			3	7	2	2		1	0	1	1	
2	2	4	3					2					2					1				
2	2	1	1	3	0	2	1	1	2	1	1	1	3	5	7	8	2	1	0	0	0	1
2	1	3	0	1				1	1				4	4				0	0			
2	2	3	3	2	4	2	0	2	1	3	1	1		3	4	5	3		1	0	0	0
1	2	2	4					3					3					1				
1	2	2	4					3					3					1				
1	3	2	1	2	1	1	1	1	1	1	1	1	3	4	5	7	3	1	0	0	0	0
2	3	3	2					1					4					0				
2	2	1	2					1					1					1				
2	2	3	1					1					5					0				
2	1	3	0	0	0	2	0	1	1	1	1	1	5	6	7	8	4	0	0	0	0	0
1	1	3	1		1	0	0	1		1	1	1	3		5	3	3	1		0	1	0
2	3	3	1	0	0	1		1	1	1	1		5		6	7		0		0	0	

2	2	3	1					1					3					1				
2	2	2	3	3	3			2	2	2			4	4	4			0	0	0		
2	2	3	2	1	2	1		1	1	1	1		4	4	4	6		0	0	0	0	
2	1	1	1	1	1	0	1	1	1	1	1	1	1	2	3	4	3	1	1	1	0	0
2	2	3	3		0	1		2		1	1		4		5	7	1	0		0	0	1
2	2	3	1					1														
2	2	4	3	1	2			2	1	1			2	5	7	4		1	0	0	0	
2	1	3	0		1			1		1			7		6			0		0		
1	2	3	0		5			1		3			4		2			0		1		
3	1	4	5					3					3					1				
2	3	3	3	3				2	2				3	5				1	0			
2	2	2	1					1					6					0				
2	3	3	0	3				1	2				5	3				0	1			
2	2	3	1	1	0	0	0	1	1	1	1	1	4	5	7	8	3	0	0	0	0	0
2	1	3	1		2	1		1		1	1		7		2	1		0		1	1	
2	2	3	1	1		0		1	1		1		3	4		4		1	0		0	
2	2	3	0	1	1	0	1	1	1	1	1	1	3	7	4	4	7	1	0	0	0	0
2	2	3	0					1					4					0				
2	2	3	2	0	0	2	2	1	1	1	1	1	4	6	6	7	3	0	0	0	0	0
1	2	1	1					1					2					1				
1	2	3	0	1	0	1		1	1	1	1		4	5	7	9		0	0	0	0	
1	2	3	2	1				1	1				4	7				0	0			
2	3	3	0					1					7					0				
2	2	3	0	2	1	0	0	1	1	1	1	1	3	4	7	8	4	1	0	0	0	0
2	3	3	0					1					6					0				
2	3	3	0	0	2	1	0	1	1	1	1	1	4	5	4	5	5	0	0	0	0	0
2	3	2	0	3		3		1	2		2		6	2				0	1			
1	2	3	1		2			1		1			4		7			0		0		
2	1	3	0	1	0	1		1	1	1	1		4	7	7	8		0	0	0	0	
2	2	2	1					1														
2	2	3	1	3	1	1	2	1	2	1	1	1	4	6	7	8	5	0	0	0	0	0
2	3	3	1	0				1	1				3	4				1	0			
2	1	3	0	1	2	1	1	1	1	1	1	1	5	5	6	2	3	0	0	0	1	0
2	2	3	1	1	0	1		1	1	1	1		7	6	7	2		0	0	0	1	
2	2	3	0	0	2		2	1	1	1		1	4	6		1	5	0	0		1	0
2	3	3	0	0	1	0	0	1	1	1	1	1	4	6	6	3	4	0	0	0	1	0
3	2	3	0	2	1	1	0	1	1	1	1	1	4	5	7	5	4	0	0	0	0	0
2	2	3	1	1	0		0	1	1	1		1	4	4	5	9	7	0	0	0	0	0
2	2	3	0	0				1	1				3					1				
2	1	3	1	2	0	2	4	1	1	1	1	3	5	7	7	8	3	0	0	0	0	0
2	2	3	3	4	5	1	1	2	3	3	1	1	4	4	5	2	7	0	0	0	1	0

2	2	3	2	1	1	2		1	1	1	1		4	6	4	3		0	0	0	1	
2	2	2	4	2	1			3	1	1			6	5	3			0	0	1		
2	2	3	3	5	3	1	0	2	3	2	1	1	3	5	6	9	4	1	0	0	0	0
2	3	3	1	2	1	1	0	1	1	1	1	1	4	3	5	3	3	0	1	0	1	0
2	2	3	1	1				1	1				4	4				0	0			
2	1	3	0	0				1	1				5	3				0	1			
2	2	3	0	2	0	3		1	1	1	2		5	4	6	4		0	0	0	0	
2	2	3	1	0	3			1	1	2			3	4	2			1	0	1		
2	2	3	0	1				1	1				4	5		8		0	0		0	
2	2	3	0	1	0	2	2	1	1	1	1	1	5	6	7	9	5	0	0	0	0	0
2	3	3	0	2	3			1	1	2			6	6	7			0	0	0		
2	2	3	2	1	0	1		1	1	1	1		4	7	4	1		0	0	0	1	
2	3	3	0	0	1			1	1	1			4	5	4			0	0	0		
2	2	3	0	3		2		1	2		1		4	4				0	0			
2	2	3	1	1	0	3		1	1	1	2		7	7	7			0	0	0		
2	1	3	4	3	0			3	2	1				3					1			
2	1	3	2	1	1	1		1	1	1	1		4	2	5			0	1	0		
1	2	1	1	2	4			1	1	3				4	7				0	0		
2	3	3	0	1	1	2		1	1	1	1		7	6	7	9		0	0	0	0	
2	3	3	1					1					4					0				
1	1	1	2					1					4					0				
2	1	3	1	2	0			1	1	1			3	4	5	4		1	0	0	0	
2	3	3	1			5		1			3		7			7		0			0	
2	2	3	0	1	0			1	1	1			4	6	6			0	0	0		
2	2	3	0	1	0			1	1	1			7	7	7	8		0	0	0	0	
1	1	3	1		4	7		1		3	3		3		7			1		0		
2	2	3	0	2	3	2	1	1	1	2	1	1	4	5	3	2	1	0	0	1	1	1
3	2	3	0					1					4					0				
1	2	3	3	3	5	3		2	2	3	2		3	4	4	7		1	0	0	0	
2	2	2	0		4		0	1		3		1	5		6	7	3	0		0	0	0
1	3	3	0					1														
3	2	2	1					1								6					0	
2	2	3	4	3	2			3	2	1			3	4	2	3		1	0	1	1	
2	1	3	0	0	0	1	1	1	1	1	1	1	5	6	7	7	4	0	0	0	0	0
2	1	3	0	2	2		0	1	1	1		1	3	4	4		3	1	0	0		0
2	2	1	4		5	4		3		3	3		2		5			1		0		
2	2	3	3	1	2			2	1	1			3	5	5			1	0	0		
2	2	3	1	3	4	3		1	2	3	2		4	2	2	1		0	1	1	1	
2	2	3	1					1					5					0				
2	2	3	0	1				1	1				5	7				0	0			
2	3	3	1					1					6					0				

2	3	3	0	1	0	0		1	1	1	1		3	6	7	8		1	0	0	0	
2	2	3	1	0	1			1	1	1				5					0			
2	2	3	0	0				1	1				6					0				
2	2	3	1	0	0	1	2	1	1	1	1	1	5	5	7	9	5	0	0	0	0	0
2	1	3	1	0	1			1	1	1			4	5	6	8		0	0	0	0	
2	2	3	1	0	1	1	0	1	1	1	1	1			4	4	5			0	0	0
3	1	3	5	2		3		3	1		2		4	4				0	0			
2	2	3	0	0	0	3	0	1	1	1	2	1	6	6	7	8	6	0	0	0	0	0
2	2	2	1					1					5					0				
2	2	3	0	2	0	1	0	1	1	1	1	1	5	5	7	8	4	0	0	0	0	0
2	2	3	0	0	2	1	1	1	1	1	1	1	6	5	5	8	4	0	0	0	0	0
2	2		3					2					3					1				
2	2	3	1					1					6					0				
2	2	3	0	2	2	1	2	1	1	1	1	1	5	6	6	6	7	0	0	0	0	0
2	3	3	2	2	0	0		1	1	1	1		7	6	7	3		0	0	0	1	
2	2	3	2	0	2		2	1	1	1		1	3	4	7	8	6	1	0	0	0	0
2	2	3	2	1	1	1	2	1	1	1	1	1	5	5	6	8	7	0	0	0	0	0
1	2	2	5	2				3	1				3	6				1	0			
2	2	3	0	1	1	2	0	1	1	1	1	1	4	5	5		3	0	0	0		0
1	2	1	1					1					2					1				
2	2	3	1	0	2	2		1	1	1	1		4	4	4	2		0	0	0	1	
2	2	3	2					1					4					0				
2	2	3	0	1	1	0	1	1	1	1	1	1	5	6	7	8	7	0	0	0	0	0
2	2	3	2		0	5	1	1		1	3	1			7	4	6			0	0	0
2	2	2	2	1	1	2		1	1	1	1			2	4	2			1	0	1	
2	2	3	1	0	1			1	1	1			4	3	6			0	1	0		
2	3		3	3				2	2				4	6				0	0			
2	2	3	2		2	1		1		1	1		3		3	2		1		1	1	
2	2	2	5					3					2					1				
2	1	3	2					1					3					1				
1	1	1	6	0	0			3	1	1				6	7				0	0		
2	2	3	1		3			1		2			5		6			0		0		
1	1	2	3	1	0		1	2	1	1		1		3	7		5		1	0		0
2	2	3	0	3	2	2	2	1	2	1	1	1	6	4	6	9	3	0	0	0	0	0
2	2	1	0	2	2			1	1	1			4	4	5	1		0	0	0	1	
2	3	3	1	4	1	0	1	1	3	1	1	1	5	7	6	4	5	0	0	0	0	0
2	2	3	0	0	0		3	1	1	1		2	5	6	7		5	0	0	0		0
2	2	3	1	3	0	1		1	2	1	1		5	4	7	1		0	0	0	1	
2	2	3	1	2		3		1	1		2		7	7		4		0	0		0	
2	2	3	1	4	3	5		1	3	2	3		4	6	4			0	0	0		
2	2	2	1	1	0	2	1	1	1	1	1	1	7	6	7	9	6	0	0	0	0	0

2	2	3	4	1	6	1	0	3	1	3	1	1	4	3	4	5	2	0	1	0	0	1
2	2	3	1	1	0			1	1	1			3	3	6	8		1	1	0	0	
2	1	2	0	0	2	2		1	1	1	1		4	5	3	2		0	0	1	1	
2	3	3	0					1					6					0				
2	2	3	0	0				1	1				5	4				0	0			
2	2	3	0					1					4					0				
2	2	3	1	2	1	5		1	1	1	3		5	5	6			0	0	0		
2	1	3	0					1					4					0				
2	2	3	0	1	2	1		1	1	1	1		4	5	6	7		0	0	0	0	
2	2	3	2	0	0	1		1	1	1	1		4	7	7	3		0	0	0	1	
2	2	3	0	1	1	2		1	1	1	1		3		1	4		1		1	0	
2	2	2	3	2	7			2	1	3				2	2				1	1		
2	2	3	1	3	1	2		1	2	1	1		4	5	7	2		0	0	0	1	
2	2	3	0	0	1	1		1	1	1	1		5	5	5	8		0	0	0	0	
2	3	3	0	1	0	0	1	1	1	1	1	1	6	4	7	4	3	0	0	0	0	0
2	2	3	1	1	0	3	2	1	1	1	2	1	3	4	6	1	3	1	0	0	1	0
2	2	3	0	3	5			1	2	3			4	7	3			0	0	1		
2	1	3	2	2	0	4		1	1	1	3		5	7	7	6		0	0	0	0	
2	2	3	0	2		2		1	1		1		4	5				0	0			
2	2	3	0	2	3	2	1	1	1	2	1	1	5	7	7	2	6	0	0	0	1	0
2	2	1	0					1					7					0				
2	2	2	0	1	0		0	1	1	1		1	6	5	7		7	0	0	0		0
2	2	3	0					1					4					0				
1	2	3	0					1					3					1				
2	2	3	2	2	3	3	2	1	1	2	2	1	4	5	7	7	4	0	0	0	0	0
2	3	3	0	2	0		2	1	1	1		1	4	5	4	5	1	0	0	0	0	1
2	3	3	2	3		2	0	1	2		1	1	3	3		8	2	1	1		0	1
1	2	2	1	2				1	1				4	3				0	1			
1	2	2	0	0	1			1	1	1			7	5	4	6		0	0	0	0	
2	2	3	1	1	0	1	1	1	1	1	1	1	5	5	7	8	2	0	0	0	0	1
2	3	3	0	3	3	0		1	2	2	1		5	3	5	6		0	1	0	0	
2	3	3	1	0	0	0	0	1	1	1	1	1	4	7	7	9	7	0	0	0	0	0
2	2	1	2					1					3					1				
2	1	3	2	1	2		4	1	1	1		3	4	4	7		5	0	0	0		0
2	3	3	1	1	0	1	2	1	1	1	1	1	4	7	7	9	4	0	0	0	0	0
2	2	3	1	1	1			1	1	1			4	4	1			0	0	1		
1	1	2	3	2	0	3	1	2	1	1	2	1	2	3	5	7	5	1	1	0	0	0
2	2	3	0	2				1	1				5	4		9		0	0		0	
1	2	2	1	1	2			1	1	1				5	3				0	1		
3	1	3	0	0	0	0	0	1	1	1	1	1	6	5	6	7	4	0	0	0	0	0
2	2	3	0					1					4					0				

1	2	1	5		3		1	3		2		1	2		4		5	1		0		0
2	3	3	0	0	0			1	1	1			3	4	4			1	0	0		
2	1	3	0	0	0			1	1	1			3	5	7			1	0	0		
2	2	3	0	1	3	2	1	1	1	2	1	1	4	5	5	1	3	0	0	0	1	0
1	2	1	0	3				1	2					3					1			
2	2	3	2	1	1	2	0	1	1	1	1	1	4	6	7	9	6	0	0	0	0	0
2	2	3	1	1	2			1	1	1			6	7	7	7		0	0	0	0	
1	2	2	0					1					2					1				
2	2	1	2	0	0			1	1	1			3	4	4			1	0	0		
2	2	3	1	1	1	0	1	1	1	1	1	1	5	6	7	5	5	0	0	0	0	0
1	2	3	0					1					4					0				
2	2	3	2	0	1	3		1	1	1	2		4	4	4	7		0	0	0	0	
2	2	3	1	2	0		0	1	1	1		1	6	7	7	9	4	0	0	0	0	0
1	2	2	2			6		1			3		7					0				
2	2	1	1					1					2					1				
2	2	3	1					1					5					0				
2	1	2	0	0	0			1	1	1			3	4	1	1		1	0	1	1	
2	2	4	2	2	1			1	1	1			3	5	5			1	0	0		
1	2	3	0	5	0			1	3	1			3	3	5			1	1	0		
2	1	3	0	5	3			1	3	2			4	6	4	3		0	0	0	1	
2	2	2	1	0	0	0		1	1	1	1		3	3	6	5		1	1	0	0	
1	1	1	0					1					4					0				
2	2	3	0	1	0	0	2	1	1	1	1	1	7	5	7	9	7	0	0	0	0	0
2	3	3	1	1	1	0	0	1	1	1	1	1	7	7	7	9	3	0	0	0	0	0
1	2	1	3	2	2			2	1	1			1	3	4			1	1	0		
2	3	3	0	0	2		0	1	1	1		1	6	7	7	9	7	0	0	0	0	0
2	2	2	1	1	0	4		1	1	1	3		1	3	7	3		1	1	0	1	
3		3		0	1	2	1		1	1	1	1		7	6	8	7		0	0	0	0
2	2	3	2	1	1			1	1	1			4	5	5	1		0	0	0	1	
2	2	3	0	1	0	0	0	1	1	1	1	1	6	5	7	9	5	0	0	0	0	0
1	2	2	1		2			1		1			4		3			0		1		
2	1	3	1	2	0			1	1	1			3	2	1	1		1	1	1	1	
2	1	1	2	2				1	1				2					1				
2	2	3	1	2	1	3	3	1	1	1	2	2	5	3	5	4	1	0	1	0	0	1
1	2	2	0					1					3					1				
2	2	3	2	1	4	1		1	1	3	1		4	5	2	2		0	0	1	1	
2	2	3	0	1				1	1				2					1				
2	1	3	2	4	3			1	3	2			1	5	4	7		1	0	0	0	
1	1	3	0	3	2			1	2	1			5	4	3	3		0	0	1	1	
2	2	3	5		3			3		2			4		1			0		1		
2	2	3	3					2					4					0				

2	2	3	1	4	1	2	3	1	3	1	1	2	4	2	2	2	5	0	1	1	1	0
2	2	3	1	0	0			1	1	1			4	7	7			0	0	0		
2	1	3	2	0	2			1	1	1			4	2	2	2		0	1	1	1	
2	2	3	0	1	1	2	0	1	1	1	1	1	5	5	3	4	7	0	0	1	0	0
2	2	3	1	4	1			1	3	1			4	5	6			0	0	0		
1	2		2					1					3					1				
2	3	3	0	0	2	1		1	1	1	1		3	3	4	1		1	1	0	1	
2	3	3	0			3		1			2		5					0				
2	2	3	0	0	0	1		1	1	1	1		7	5	7	9		0	0	0	0	
2	1	3	1	0	0	2	0	1	1	1	1	1	5	6	7	6	6	0	0	0	0	0
2	2		0	2	1	0	3	1	1	1	1	2	4	4	3	3	2	0	0	1	1	1
2	1	3	0		0		0	1		1		1	4				7	0				0
2	2	3	1	0				1	1				5	6				0	0			
2	2	3	1	4				1	3				3	2				1	1			
2	2	3	1	2	0			1	1	1			5	5	6	8		0	0	0	0	
2	2	2		1	1				1	1			3	2	6			1	1	0		
2	2	3	0	2	5	1		1	1	3	1		5	4	5	5		0	0	0	0	
2	1	3	3		0			2		1			4					0				
1	1	1	2	3				1	2				5	4				0	0			
1	2	3	1	2	2			1	1	1			3	4	7			1	0	0		
2	3	3	1	0		0	0	1	1		1	1	4			8	3	0			0	0
2	2	3	4	1	1	4		3	1	1	3		3	5	7			1	0	0		
3	2	3	4	1	0	2	0	3	1	1	1	1	5	4	6	7	4	0	0	0	0	0
2	3	3	1	1	0			1	1	1			4	6	7	7		0	0	0	0	
2	2	4	5	3	1	4	3	3	2	1	3	2	6	3	2	2	1	0	1	1	1	1
2	2	3	1					1					4					0				
1	3	1	1	1	2		1	1	1	1		1	4	4	7	8	2	0	0	0	0	1
3	1	3	2	0				1	1				4	4				0	0			
2	2	3	3					2					3					1				
2	2	1	2	0	0	2	1	1	1	1	1	1	4	2	2	1	5	0	1	1	1	0
2	3	3	2					1					4					0				
2	3	3	1	2	2	3		1	1	1	2		5	7	7	9		0	0	0	0	
2	2	3	1					1					5					0				
1	2	2	0					1														
2	3	4	0	3	2		1	1	2	1		1	5	6	7	8	3	0	0	0	0	0
1	2	3	4	2				3	1				3	4				1	0			
2	2	3	3					2					3					1				
2	2	3	2	1	2	2		1	1	1	1		5	7	5	5		0	0	0	0	
2	2	3	1	3	2	4	1	1	2	1	3	1	6	6	7	9	4	0	0	0	0	0
2	2	3	0			1		1			1		3			8		1			0	
2	2	2	0	3	0	5	1	1	2	1	3	1	3	2	3		1	1	1	1		1

2	2	3	1	0				1	1				4	6				0	0			
2	3	3	1	1	1	0		1	1	1	1		6	5	4	4		0	0	0	0	
2	1	2	0					1					3					1				
1	2	3	0	1	3	2		1	1	2	1		3	4	5	2		1	0	0	1	
2	3	3	0	1	0	2	1	1	1	1	1	1	7	7	7	8	5	0	0	0	0	0
2	1	4	3	1	4			2	1	3			5	3	7			0	1	0		
2	2	3	2	1	0	2		1	1	1	1		5	5	7			0	0	0		
2	2	2	3		3			2		2			2		3			1		1		
2	2	3	1	5	2			1	3	1			4	4	4			0	0	0		
2	2	3	2					1														
1	1	3	6	1	0	3	0	3	1	1	2	1	3	4	5	8	5	1	0	0	0	0
2	2	3	0	1	0	2	0	1	1	1	1	1	4	3	5	7	1	0	1	0	0	1
2	2	3	0	0	1	2		1	1	1	1		5	7	7	8		0	0	0	0	
2	2	3	1					1					5					0				
1	2	1	2					1					3					1				
2	2	3	0	1	1	3	2	1	1	1	2	1	5	5	7	8	7	0	0	0	0	0
2	2	3	1					1					4					0				
2	2	1	2	2				1	1				2					1				
2	2	3	1		3	2	0	1		2	1	1	4		3	8	7	0		1	0	0
2	2	3	2	1	2	5		1	1	1	3		4	6	3	2		0	0	1	1	
2	2	3	1	1	1	1	0	1	1	1	1	1	4	5	4	6	5	0	0	0	0	0
2	1	1	2	1	2	3		1	1	1	2		2	4	5			1	0	0		
3	2	3	2	3	3			1	2	2			4	2	7			0	1	0		
2	2	3	0	1	0	1	1	1	1	1	1	1	7	7	7	9	5	0	0	0	0	0
2	2	3	0	2	0	2	0	1	1	1	1	1	5	6	7	4	4	0	0	0	0	0
2	3	3	1	4	3	3	2	1	3	2	2	1	5	5	7	8	4	0	0	0	0	0
2	2	3	0			2		1			1					1					1	
1	2	1	0					1					4					0				
3	1	4	1		1			1		1			3		3			1		1		
2	2	3	4		1	0	0	3		1	1	1	7		7	7	5	0		0	0	0
2	1	3	0	1	0		1	1	1	1		1	3	4	7		6	1	0	0		0
2	2	3	2					1					4					0				
2	2	3	0	2	0			1	1	1			5	7	7			0	0	0		
3	2	3	0	3	4			1	2	3			4	5	4			0	0	0		
2	2	3	0	0	2	2		1	1	1	1		4	6	4	8		0	0	0	0	
2	2	2	1					1					4					0				
2	2	3	1	0	0	2	1	1	1	1	1	1	4	5	7	8	7	0	0	0	0	0
1	2	1	0	2	1	1		1	1	1	1			3	5				1	0		
2	2	3	1	2	3	2	1	1	1	2	1	1	4	4	3	1	7	0	0	1	1	0
2	3	3	0	1	0	0	1	1	1	1	1	1	7	7	7	9	4	0	0	0	0	0
2	1	2	4					3					4					0				

2	1	3	0	1	0	2		1	1	1	1		4	5	7	1		0	0	0	1	
2	2	2	1	2	3	2		1	1	2	1		4	4	5	4		0	0	0	0	
2	2	3	0	1				1	1				4	4				0	0			
2	1	2	1		3			1		2												
2	3	3	3	5	2			2	3	1			3	4	7			1	0	0		
1	2	2	2		3	2		1		2	1		3		3	2		1		1	1	
3	3	3	0	0				1	1					2					1			
2	2	3	2					1					4					0				
2	1	3	1	1	1	1		1	1	1	1		4	3	5	7	2	0	1	0	0	1
2	2	3	0	3	2	0		1	2	1	1		4	4	3	3		0	0	1	1	
2	2	3	1	3	5	0		1	2	3	1		3	3	2	1		1	1	1	1	
2	2	3	0	2	0	0	1	1	1	1	1	1	6	7	7	9	6	0	0	0	0	0
2	2	3	1	1	1			1	1	1			3	6	7	8		1	0	0	0	
2	2	4	4	2	6	4	3	3	1	3	3	2	4	7	7	8	4	0	0	0	0	0
2	2	3	0	1	0	0		1	1	1	1		6	6	7			0	0	0		
2	2	3	3	2	0	2		2	1	1	1		4	3	6	6		0	1	0	0	
2	3	3	0	2	0	1	1	1	1	1	1	1	6	7	5	6	3	0	0	0	0	0
2	2	3	0	1	2	5		1	1	1	3		6	6	4			0	0	0		
2	2	3	2	1	1			1	1	1			4	5	7	9		0	0	0	0	
2	2	3	0	1				1	1				5	6				0	0			
2	2	3	1	2	2	2		1	1	1	1		4	4	3	3		0	0	1	1	
2	2	3	0	2		2	0	1	1		1	1	4	5		8	4	0	0		0	0
2	3	3	0	0	0	1		1	1	1	1		4	5	7	8		0	0	0	0	
2	3	3	1	2	2	5	3	1	1	1	3	2	5	7	7	9	3	0	0	0	0	0
2	3	3	2	2	2	0		1	1	1	1		3	4	7			1	0	0		
2	2	3	1	1	3	0		1	1	2	1		4	6	4	1		0	0	0	1	
1	1	1	1	0				1	1				1	2				1	1			
2	2	3	2	1	1	2		1	1	1	1		6	7	7			0	0	0		
1	2	1	3	3	2	0	1	2	2	1	1	1	2	6	7	8	6	1	0	0	0	0
1	2	2	3					2					3					1				
2	2	2	3	1	0	1		2	1	1	1			5	7	8			0	0	0	
2	2	3	0	1	1	1	0	1	1	1	1	1	4	5	4	8	7	0	0	0	0	0
2	2	3	0	2	0	4		1	1	1	3		4	5	7	9		0	0	0	0	
2	2	3	0					1					5					0				
2	2	3	0	3	3			1	2	2			5	7	5			0	0	0		
2	2	3	4					3					2					1				
1	1	2	0					1														
2	2	3	1	0	0	4		1	1	1	3		4	4	4	9		0	0	0	0	
2	2	3	0	1	0	0		1	1	1	1		5	7	7	8		0	0	0	0	
3	3	2	4		3			3		2			3		4			1		0		
2	2	3	2					1					3					1				

2	1	3	0	1	1	0	1	1	1	1	1	1	4	5	6	4	4	0	0	0	0	0
2	1	3	4		5			3		3			4		2			0		1		
2	2	3	0					1					3					1				
2	2	3	0	1	0		2	1	1	1		1	4	4	7		4	0	0	0		0
2	2	3	0	1	0			1	1	1			4	3	6			0	1	0		
2	1	3	0	1	2			1	1	1			3	6	4			1	0	0		
2	2	3	0	1	0	2		1	1	1	1			7	7	9			0	0	0	
2	3	3	0	1	2	1	0	1	1	1	1	1	4	7	7	8	5	0	0	0	0	0
1	1	3	4	3	1	2	3	3	2	1	1	2	3	4	6	6	2	1	0	0	0	1
2	1	3	0	1	6			1	1	3			3	5	4			1	0	0		
2	2	3	1	3	3	6	4	1	2	2	3	3	3	3	3	1	1	1	1	1	1	1
3	2	3	0					1					4					0				
2	2	3	2	4	4	3		1	3	3	2		5	2	2	1		0	1	1	1	
2	2	3	4	2	0		0	3	1	1		1	6	6	6		4	0	0	0		0
2	2	3	2		2	6		1		1	3		4		2			0		1		
2	1	3	1	1	0	1	1	1	1	1	1	1	3	4		1	1	1	0		1	1
3	3	4	2	2				1	1				2	2				1	1			
2	2	3	1					1					6					0				
2	2	3	0	2	2	2	0	1	1	1	1	1	3	3	3	2	3	1	1	1	1	0
2	2	2	3	1	2			2	1	1			3	5	6			1	0	0		
2	2	2	3	2	3			2	1	2				4	2	8			0	1	0	
1	1	3	0	2	0	0	2	1	1	1	1	1	3	4	5	6	6	1	0	0	0	0
2	2	3	3		0	1	2	2		1	1	1	4		6	7	7	0		0	0	0
2	1	3	2	1	0		3	1	1	1		2	3	5	7		3	1	0	0		0
2	2	3	1	1	0	1		1	1	1	1		5	4	6	6		0	0	0	0	
3	2	3	3	1	1		1	2	1	1		1	4	5	5	5	3	0	0	0	0	0
1	2	3	3	1	1		1	2	1	1		1	3		5	4	2	1		0	0	1
2	2	2	0					1					7					0				
1	1	2	4	1	3			3	1	2			3	4	4	1		1	0	0	1	
2	1	3	1	1	1			1	1	1			3	2	7	9		1	1	0	0	
2	2	3	1	0	1	0	2	1	1	1	1	1	3	4	6	7	6	1	0	0	0	0
3	3	3	2	1	0	1	0	1	1	1	1	1	5	6	6	8	6	0	0	0	0	0
2	1	3	1	2	5	2	0	1	1	3	1	1	5	5	3	7	7	0	0	1	0	0
2	2	3	0	1	1	2	0	1	1	1	1	1	7	5	7	6	5	0	0	0	0	0
3	3	3	1	0	1	2	1	1	1	1	1	1	3	3	3	4	4	1	1	1	0	0
2	2	3	2					1					5					0				
2	2	3	0	3	3			1	2	2			6	7	7			0	0	0		
2	2	3	1	1	1	3	2	1	1	1	2	1	4	6	7	6	6	0	0	0	0	0
2	2	2	2	1	0			1	1	1			4	3	2			0	1	1		
2	2	3	3	3	4		1	2	2	3		1	4	4	6	4	6	0	0	0	0	0
2	2	3	1	3	2		0	1	2	1		1	4	5	7	6	7	0	0	0	0	0

1	3	3	3	2	3	1		2	1	2	1		4	4	7	8		0	0	0	0	
2	2	3	4	2	5	3	0	3	1	3	2	1	4	3	3	7	4	0	1	1	0	0
1	2	3	2					1					5					0				
2	2	3	2	2	1	4		1	1	1	3		5	4	6	7		0	0	0	0	
2	2	3	0	0	0	0		1	1	1	1		5	4	7	9		0	0	0	0	
2	2	3	1	1	2			1	1	1			6	4	3	2		0	0	1	1	
1	2	3	0		3			1		2			3		3			1		1		
1	2	3	2					1					4					0				
2	2	2	0	2	2	3		1	1	1	2		6	7	6	7		0	0	0	0	
2	2	3	2					1					3					1				
2	2	3	0	2	0	0		1	1	1	1		6	5	5	7		0	0	0	0	
2	2	3	0	1				1	1				6	7				0	0			
1	2	1	0	1	0		1	1	1	1		1	2	6	7		6	1	0	0		0
2	2	2	3					2					3					1				
1	1	1	1					1					4					0				
2	2	3	1		2	2	2	1		1	1	1	6		6	3	4	0		0	1	0
2	1	3		4	1				3	1			3	4	6			1	0	0		
1	1	3	0	1				1	1				4	1				0	1			
1	2	3	5					3														
2	2	3	0	1	0			1	1	1			4	4	7	5		0	0	0	0	
2	1	3	2	2	3	4	3	1	1	2	3	2	4	3	3	1		0	1	1	1	
1	2	1	0	0	1			1	1	1				4					0			
2	1	3	4	4	4			3	3	3			7	7	2			0	0	1		
2	2	3	0					1					3					1				
1	1	4	4	3	1	3		3	2	1	2			4	7	3			0	0	1	
2	2	3	3					2					4					0				
2	2	3	1	0	2	0		1	1	1	1		3	4	5	3		1	0	0	1	
1	1	3	2	0				1	1				4	5				0	0			
2	3	3	0	3	0	0	0	1	2	1	1	1	4	5	7	7	6	0	0	0	0	0
2	2	3	0	1	1	0		1	1	1	1		4	5	2	8		0	0	1	0	
2	2	3	1	0	1			1	1	1			3	4	3			1	0	1		
1	2	3	4	4	2	4	1	3	3	1	3	1	4	4	5	8		0	0	0	0	
2	1	3	0	1	1			1	1	1			4	6	6	4		0	0	0	0	
2	2	2	2	3	2	0		1	2	1	1		4	6	7	7		0	0	0	0	
1	2	3	1		1			1		1			5		3			0		1		
2	2	3	0					1														
2	2	3	2	0	4			1	1	3			4	6	3			0	0	1		
1	2	2	0	1	2			1	1	1			4	5	6			0	0	0		
2	2	3	0	0	2	1	1	1	1	1	1	1	6	5	7	9		0	0	0	0	
2	2	3	2	2		0	1	1	1		1	1	4	5		1	3	0	0		1	0
2	1	2	2	2	5	4	0	1	1	3	3	1	3	3	3	1		1	1	1	1	

2	2	3		0	1	1			1	1	1		4	4	4	5		0	0	0	0	
2	2	3	2	2	1		0	1	1	1		1	4	4	7	8	6	0	0	0	0	0
2	2	3	0	0	1	1	0	1	1	1	1	1	4	5	6	8	4	0	0	0	0	0
2	3	1	5	3	4			3	2	3			3	2	7	1		1	1	0	1	
2	3	4	3					2					3					1				
2	2	3	0					1					4					0				
2	2	3	2					1					4					0				
2	2		0	2	1	3		1	1	1	2			7	4	7			0	0	0	
2	2	3	0	2	3			1	1	2				3	3	3			1	1	1	
2	2	3	2	4	4	2	0	1	3	3	1	1	7	2	4	9	4	0	1	0	0	0
2	1	3	1		0	1		1		1	1		4		5	6		0		0	0	
1	1	1	4		3			3		2			2		3	3	1	1		1	1	1
2	2	1	0	2	2	3		1	1	1	2			2	4	7			1	0	0	
1	2	3	2	1	2			1	1	1			5	2	7			0	1	0		
2	2	3	2					1					3					1				
2	2	3	0					1					4					0				
1	2	2	1	3	1		1	1	2	1		1	1	4	3		5	1	0	1		0
2	1	3	1	0	3	6		1	1	2	3		4	3	5	2		0	1	0	1	
2	2	3	2	2				1	1				4	6				0	0			
2	2	3	1	3	3	4	3	1	2	2	3	2	4	5	7	6	2	0	0	0	0	1
2	1	3	0	0	0	1		1	1	1	1		5	5	7	8		0	0	0	0	
2	1	1	1		1	2	1	1		1	1	1	3		3	1	2	1		1	1	1
2	2	3	0					1					4					0				
2	3	3	1		1			1		1			4		6	8		0		0	0	
1	2	2	3					2					3					1				
2	2	3	3	2	2	2		2	1	1	1		6	3	4	2		0	1	0	1	
1	3	3	4	4	2	5	1	3	3	1	3	1	2	2	3	6	1	1	1	1	0	1
2	2	1	4	3	2	3	0	3	2	1	2	1	2	4	2	5	2	1	0	1	0	1
2	1	3	0		1	2		1		1	1		4		5	6		0		0	0	
2	3	3	0	2	4	1		1	1	3	1		5	6	7	9		0	0	0	0	
2	3	3	1					1					5					0				
2	2	3	0	0	2		0	1	1	1		1	7	5	5		2	0	0	0		1
2	1	1	2					1					2					1				
2	2	3	1	2	0	2	3	1	1	1	1	2	6	7	6	8	3	0	0	0	0	0
2	2	3	0					1					5					0				
2	2	3	1	2	0			1	1	1			4	5	5			0	0	0		
2	2	3	3	2	3	0	1	2	1	2	1	1	5	7	7	8	6	0	0	0	0	0
3	2	3	1					1					5					0				
2	2	3	0	4	7			1	3	3			4	2	4			0	1	0		
2	2	2	2	1	2	2		1	1	1	1			4	4	7			0	0	0	
1	1	2	2					1														

2	1	3	1	1				1	1				4	3				0	1			
2	1	2	0					1					6					0				
2	2	1	3	3	1	2		2	2	1	1			6	2	1			0	1	1	
2	2	3	0	1	1	1		1	1	1	1		6	6	3	1		0	0	1	1	
2	3	3	3	0	0	3	2	2	1	1	2	1	4	4	7	6	5	0	0	0	0	0
2	2	3	0	1	0	0		1	1	1	1		5	7	7	9		0	0	0	0	
2	2	3	0		0	0		1		1	1		4		6	7		0		0	0	
1	2	3	1	5	2		3	1	3	1		2	4	2	2		6	0	1	1		0
2	2	3	1					1					5					0				
3	1	3	0	0	2			1	1	1			3	7	3			1	0	1		
2	2	1	2					1					3					1				
2	2	1	3					2					3					1				
2	2	3	0	0	2	1		1	1	1	1		5	4	3	6		0	0	1	0	
2	2	2	3		5	7	1	2		3	3	1	5		5		4	0		0		0
1	2	3	1	1				1	1				4	5				0	0			
2	3	3		1	0				1	1			6		5		7	0		0		0
3	1	3	0	0	2			1	1	1			4	4	5	1		0	0	0	1	
2	1	4	0	2	1	5		1	1	1	3		4	5	6	4		0	0	0	0	
2	1	3	1					1					5					0				
2	1	3	0	1	1			1	1	1			4	5	5	1	1	0	0	0	1	1
2	2	3	4					3														
1	2	1	1	1	1	0	0	1	1	1	1	1	5	5	4	8	3	0	0	0	0	0
3	1	3	0	2	0	0	2	1	1	1	1	1		7	3	3	6		0	1	1	0
2	1	3	2	2	3	5		1	1	2	3		4		1	1		0		1	1	
2	2	3	0					1														
2	3	3	1	1	2	0		1	1	1	1		3	6	3	4		1	0	1	0	
2	2	3	0	2				1	1				5	6				0	0			
2	2	3	0	1	0			1	1	1			4	5				0	0			
2	2	3	1	3	1	2	0	1	2	1	1	1	4				3	0				0
2	1	3	0					1					2					1				
2	3	3	0	1	1			1	1	1			4	4	6			0	0	0		
2	2	3	0	1	0	3	3	1	1	1	2	2	5	3	7	9	5	0	1	0	0	0
2	2		0	0	0	2	0	1	1	1	1	1	4	6	7	8	6	0	0	0	0	0
2	2	3	1	0	1			1	1	1			3	4	1			1	0	1		
2	3	3	1	0	0			1	1	1			3	4	4			1	0	0		
2	2	3	2	2	1			1	1	1			5	4	2	2		0	0	1	1	
2	2	2	2		2	6		1		1	3		5		2	1		0		1	1	
2	1	4	1	2	1	2	1	1	1	1	1	1	2	4			5	1	0			0
2	2	3	2					1					5					0				
2	3	3	1	4				1	3				6	2				0	1			
2	2	3	0	5	2	1		1	3	1	1		4	3	4	7		0	1	0	0	

2	1	3		4	0				3	1			4	5	3			0	0	1		
2	2	3	0	2	2			1	1	1			4	3	4			0	1	0		
2	2	3	3	1				2	1				4	6				0	0			
1	2	4	1					1					2					1				
2	2	3	0	0	0	3	2	1	1	1	2	1	6	4	7	8	3	0	0	0	0	0
2	1	3	0					1					5					0				
2	2	1	3					2					2					1				
2	2	3	0	1		0		1	1		1		4	6				0	0			
1	1	1	1					1														
2	2	3	2	0	0	1		1	1	1	1		4	7	7	5		0	0	0	0	
2	3	3	2	3	0	0		1	2	1	1		4	5	7	8		0	0	0	0	
2	3	3	0	0	1			1	1	1			5		2			0		1		
2	2	3	2	4				1	3				3	5				1	0			
1	2	1	1	2	2			1	1	1			3	6	4	4		1	0	0	0	
2	2	3	1	2	1	2		1	1	1	1		4	6	6	3		0	0	0	1	
2	3	3	1	3	0	1	0	1	2	1	1	1	7	6	7	9	5	0	0	0	0	0
2	2	3	3	0	2	0	1	2	1	1	1	1	4	4	7	8	5	0	0	0	0	0
2	1	3	0	0	1	2		1	1	1	1		5		7	6		0		0	0	
1	2	3	3					2					3					1				
3	3	3	0	0	4	2		1	1	3	1		7	7	2			0	0	1		
3	1	4	1		5	1	2	1		3	1	1	3		2	1	7	1		1	1	0
1	2	2	0		2			1		1			4		2			0		1		
2	1	3	0	2	1			1	1	1				4	6				0	0		
2	2	3	1	2	2			1	1	1			5	5	5			0	0	0		
2	1	3	1	3	1			1	2	1			3	5	6			1	0	0		
1	2	2	3					2					2					1				
2	3	3	1		2	3		1		1	2		4		5			0		0		
2		2	1	0	2			1	1	1			3	4	2			1	0	1		
2	2	3	1	0				1	1				3	3				1	1			
2	2	3	5	2	2			3	1	1			4	4	3			0	0	1		
2	1	3	0					1					3					1				
1	2	2	1	0	0	1	1	1	1	1	1	1	3	5	7	8	4	1	0	0	0	0
2	2	3	0	0	1			1	1	1			4	4	2			0	0	1		
2	1	3	0	1	1		6	1	1	1		3	4	6	5		2	0	0	0		1
2	1	3	1	2	1	1	0	1	1	1	1	1	4	5	6	7	2	0	0	0	0	1
2	3	3	2			1		1			1		5			3		0			1	
1	2	1	1	1	2	0		1	1	1	1		4	4	7	8		0	0	0	0	
2	2	2	0					1					4					0				
2	2	2	0	0				1	1				4	4				0	0			
2	2	3	5	1		2		3	1		1		4	6		1		0	0		1	
2	2	3	1	1	1	0	0	1	1	1	1	1	5		7	3	5	0		0	1	0

2	2	3	1	1	2	1	1	1	1	1	1	1	5		3	1	4	0		1	1	0
2	2	2	3	2	3	4	2	2	1	2	3	1		3	2		3		1	1		0
2	3	3	2					1					4					0				
3	2	3	2	1	0	6	6	1	1	1	3	3	6	2	4	6	1	0	1	0	0	1
2	1	3	1					1					2					1				
2	2	1	4	4	4	6		3	3	3	3		4	3	5	4		0	1	0	0	
2	1	1	3	0	2			2	1	1			2	2	2	4		1	1	1	0	
1	2	3	5	0	0	4	0	3	1	1	3	1		4	4		1		0	0		1
2	3	3	0	1	0	2	1	1	1	1	1	1	5	7	4	6	7	0	0	0	0	0
2	1	3	1		0	2	1	1		1	1	1	5		7	3	1	0		0	1	1
2	2	3	1	2	0	2	3	1	1	1	1	2	3	5	7	5	3	1	0	0	0	0
2	3	3	0					1					3					1				
2	2	2	1	3				1	2				3	6				1	0			
2	2	3	0					1					4					0				
1	2	2	1	3	2			1	2	1			3	7	7			1	0	0		
1		1	2		5			1		3					5					0		
2			5					3														
2	2	3	0					1					7					0				
2	3	3	5	3	2	1		3	2	1	1		7	7	7	8		0	0	0	0	
2	2	2	2		1	1		1		1	1		4		3	3		0		1	1	
2	2	2	4					3					3					1				
1	2	3	3		3			2		2			4		7			0		0		
2	2	3	2					1					3					1				
3	3	1	4					3					2					1				
2	3	3	0	3	2	1	1	1	2	1	1	1	5	4	7	8	1	0	0	0	0	1
2	2	3	2	1	0	1	2	1	1	1	1	1	3	2	2	4	1	1	1	1	0	1
2	1	3	1	1	1	0		1	1	1	1		4	7	7			0	0	0		
2	2	3	1	3	3	4	0	1	2	2	3	1	3	4	2	6	1	1	0	1	0	1
1	2	3	0	0				1	1				3	6				1	0			
2	2		0					1					2					1				
2	2	2	1					1														
2	3	3	0	0	0			1	1	1			5	7	7			0	0	0		
1	2	3	0					1					4					0				
2	3	2	1		0	1		1		1	1		6		7	9		0		0	0	
2	2	2	1	0	2	4	0	1	1	1	3	1	4	3	2		4	0	1	1		0
2	2	1	4	5	5		4	3	3	3		3	2	4	3	1	4	1	0	1	1	0
2	1	3	0	0	1	1		1	1	1	1		4	5	3	6		0	0	1	0	
1	2	1	2	1	0	6		1	1	1	3		5	3	7	3		0	1	0	1	
2	3	3	0	0	0	1	1	1	1	1	1	1	2	3	7		7	1	1	0		0
2	2	4	1					1					2					1				
2	2	3	0	2	2			1	1	1			5	4	7	8		0	0	0	0	

3	1			2	0				1	1				5	5	1			0	0	1	
2	2	3	1	2	5			1	1	3			4	3	3			0	1	1		
2	2	3	3		4			2		3			3		3			1		1		
2	2	3	3					2					3					1				
2	3	3	2		3			1		2			3		1			1		1		
1	1	3	2	3	3	5	1	1	2	2	3	1	7	3	5	6	4	0	1	0	0	0
3	1	3	2					1					3					1				
2	3	3	3	6	7	2	2	2	3	3	1	1	4	2	6	4	3	0	1	0	0	0
2	1	3	1	2	1	0		1	1	1	1		3	3	7	7		1	1	0	0	
2	2	3	2					1					5					0				
2	2	3	0	2	2		1	1	1	1		1	4	4	6	8	5	0	0	0	0	0
2	3	1	5					3					4					0				
2	2	1											2					1				
2	2	3	1	1	1	1	1	1	1	1	1	1	4	5	4	5	5	0	0	0	0	0
3	1	3	0	0	0	1		1	1	1	1		2	2	7			1	1	0		
2	2	3	0	0	0	1		1	1	1	1		5	3	5	3		0	1	0	1	
2	2	3	1	2	0	2		1	1	1	1		3	3	3	2		1	1	1	1	
3	2		0	0	0	3	2	1	1	1	2	1	6	7	7	9	1	0	0	0	0	1
1	3	1	3	3	2	2	1	2	2	1	1	1	4	7	5	8	1	0	0	0	0	1
2	2	3	1	0	0		3	1	1	1		2	4	4	7		3	0	0	0		0
2	3	3	0	0	0	0		1	1	1	1		3	3	3	3	3	1	1	1	1	0
3	2	3	0	3	3	0		1	2	2	1		5	3	2	3		0	1	1	1	
2	1	2	2					1					3					1				
2	2	3	2	3	3			1	2	2			3	7	6			1	0	0		
2	2	3	1	3	1			1	2	1			5	6	7	1		0	0	0	1	
2	2	3	0	1	1	1		1	1	1	1		5	5	7	6	6	0	0	0	0	0
2	3	3	1					1					5					0				
2	3	3	2	2	3			1	1	2			4		3			0		1		
2	2	3	1	4	5			1	3	3			4	3	2			0	1	1		
2	2	3	1	1	1			1	1	1			4	4	6			0	0	0		
2	3	3	1	0	0	4	2	1	1	1	3	1	3	3	5	8	1	1	1	0	0	1
2	2	3	0	1	0	2		1	1	1	1		4	3	5	7		0	1	0	0	
3	2		1	2	0		1	1	1	1		1	6	7	7		7	0	0	0		0
3	2	3	0	0	2	3	6	1	1	1	2	3	4	7	3	2	1	0	0	1	1	1
2	2	1	5	1	4			3	1	3				3	2				1	1		
2	3	3	0	1	1			1	1	1			5		7			0		0		
2	2	3	1	1				1	1				4	3				0	1			
2	2	3	0	0	0	0		1	1	1	1		5	6	7	2		0	0	0	1	
2	3	3	3	1	5	3		2	1	3	2		3	3	6	5		1	1	0	0	
2	2	3	2	2	2			1	1	1			6	6	7			0	0	0		
3	2	3	3	3	1			2	2	1			3	4	4	1		1	0	0	1	

2	2	3	0					1					4					0				
2	1	3	1	2	2			1	1	1			4	2	3	4		0	1	1	0	
2	2	3	1	1	1		1	1	1	1		1	6	6	7	9	3	0	0	0	0	0
2	2	1	0					1														
2	2	3	1					1					4					0				
2	2	2	1	1	0	0		1	1	1	1		4	5	7	8		0	0	0	0	
2	2	3	2	0	0	0		1	1	1	1		3	6	6	3		1	0	0	1	
2	3	3	0	2	0	2	1	1	1	1	1	1	1	6	7	2	3	1	0	0	1	0
1	1	2	3					2														
1	3	3	2					1					4					0				
2	2	3	0		1			1		1			6		7			0		0		
2	3	3	2	0	0	0		1	1	1	1		5	7	7	9		0	0	0	0	
2	2	3	2		2	1	0	1		1	1	1	3		7	8	7	1		0	0	0
2		2		1	0	0	1		1	1	1	1		5	5	6	7		0	0	0	0
2	2	3	1	1	0		0	1	1	1		1	6	5	7		5	0	0	0		0
2	2	3	1	0				1	1				4	5				0	0			
2	3	3	0	1	1	1	0	1	1	1	1	1	4	5	7	1	2	0	0	0	1	1
1	3	3	3					2					3					1				
3	3	3	1	4	0	0	0	1	3	1	1	1	5	3	6	1	4	0	1	0	1	0
2	2	3	3	0	0			2	1	1			5	6	5			0	0	0		
3	2	3	0	1	1	2		1	1	1	1		6	4	7	8		0	0	0	0	
2	2	3	1	0	2	2		1	1	1	1		4		1			0		1		
2	2	3	0	1	0	1		1	1	1	1		5	3	5	6		0	1	0	0	
2	2	3	0	1	0	4	2	1	1	1	3	1	3	3	4	3	5	1	1	0	1	0
2	2	3	4	4	1	2	2	3	3	1	1	1	4	3	7	3	7	0	1	0	1	0
2	2	3	0	0				1	1				7	7				0	0			
1	1	2	1		0			1		1			3		3			1		1		
2	2	3	2	1	3			1	1	2			4	5	5			0	0	0		
2	1	3	0	4	0		0	1	3	1		1	4	2	3		3	0	1	1		0
2	2	3	0	1	2	2	4	1	1	1	1	3	5	7	4	1	1	0	0	0	1	1
1	1	3	0	0	2		2	1	1	1		1	3	2	4		1	1	1	0		1
3	2	3	0	0	0	0		1	1	1	1		7	6	7	3		0	0	0	1	
2	2	3	1	0	1			1	1	1			4	4	5			0	0	0		
2	2	3	0	2	1			1	1	1			3	5	5			1	0	0		
2	1	3	1	2	3			1	1	2			4	4	5			0	0	0		
2	2	3	2	1	1	0	1	1	1	1	1	1	3	6	7	6	7	1	0	0	0	0
3	3	3	3	3	2	1	0	2	2	1	1	1	4	4	7	8	5	0	0	0	0	0
2	3	3	1	0	0			1	1	1			4	4	5			0	0	0		
2	1	3	1					1														
2	2	3	0	1	1	2		1	1	1	1		5	7	5	5		0	0	0	0	
2	3	3	3					2					5					0				

2	1	2	1	3	3	3		1	2	2	2		3	5	6	5		1	0	0	0	
2	2	3	0	1	0	1	2	1	1	1	1	1	6	6	7	9	4	0	0	0	0	0
2	2	3	4	3	2	2	4	3	2	1	1	3	3	4	4	5	6	1	0	0	0	0
2		3	2	5				1	3													
2	2	3	2	2	1	0		1	1	1	1		4	5	7	8		0	0	0	0	
1	1	2	3					2					3					1				
2	1	2	2	5	5			1	3	3			4	2	2	4		0	1	1	0	
2	3	3	1	2	3	1	1	1	1	2	1	1	4	7	6	5	7	0	0	0	0	0
2	1	3	0	5	3	0	1	1	3	2	1	1	4	3	2	1	6	0	1	1	1	0
3	1	3	1	0				1	1				4	5				0	0			
2	3	3	1	2	1			1	1	1			5		7			0		0		
2	2	3	0	0	0	0	0	1	1	1	1	1	5	7	7	8	5	0	0	0	0	0
2	2	3	0	1	0		0	1	1	1		1	5	5	6	8	1	0	0	0	0	1
1	2	2	3	3	1			2	2	1			7	2	6			0	1	0		
2	3	2	2	4				1	3				3	5				1	0			
2	2	3	2					1					3					1				
2	2	3	0		2	2	2	1		1	1	1	4		6	2		0		0	1	
2	2	1	0	2	3	3	2	1	1	2	2	1	2	4	3	3	1	1	0	1	1	1
2	3	3	0	0	0	2		1	1	1	1			6	7	8			0	0	0	
2	2	3	2	2				1	1				4	7				0	0			
2	2	3	1	1		1		1	1		1		7	3		7		0	1		0	
2	1	3	1	3	3	2	0	1	2	2	1	1	4	3	4	1	3	0	1	0	1	0
2	2	3	0		0		2	1		1		1	3		3		1	1		1		1
2	1		0			3	1	1			2	1	6				1	0				1
2	1	3	1					1					3					1				
1	2	1	0	3	5			1	2	3			3	2	4			1	1	0		
3	3	3	0	1	0	4		1	1	1	3		4	6	4			0	0	0		
2	2	3	1	1	1	2	1	1	1	1	1	1	4		7	8	4	0		0	0	0
1	2	1	1	2	3			1	1	2				4	7				0	0		
2	2	3	0	0	0			1	1	1			4	4	5	2		0	0	0	1	
2	3	3	2	4	5		1	1	3	3		1	5	4	6		6	0	0	0		0
2	3	3	0	2	1			1	1	1			6	4	5			0	0	0		
1	1	2	1		2	1		1		1	1		2			4		1			0	
2	3		1	3				1	2				2	2				1	1			
2	2	3	3	0	1	2	2	2	1	1	1	1	3	3	6	6	2	1	1	0	0	1
2	2	3	0	1	0			1	1	1			3	4	5	3		1	0	0	1	
2	2	3	2	2	0	1		1	1	1	1		4	5	7	9		0	0	0	0	
2	3	3	0	1	1	0	0	1	1	1	1	1	4	4	4	8	2	0	0	0	0	1
2	2	3	0		1	2	0	1		1	1	1	3		6	8	5	1		0	0	0
2	2	2	1	1				1	1				2	6				1	0			
2	2	3	1	2	1	2	0	1	1	1	1	1	4	4	7	8	7	0	0	0	0	0

2	1	3	3	2	0			2	1	1			4	5	6	8		0	0	0	0	
1	2	3	3		2	7		2		1	3		4		7	2		0		0	1	
2	2	3	0	0	0			1	1	1			5	6	6	8		0	0	0	0	
2	2	3	1					1					3					1				
2	1	3	1	1	2	4		1	1	1	3		5	3	6	7		0	1	0	0	
2	2	3	0	0	1	0	2	1	1	1	1	1	5	6	7	8	6	0	0	0	0	0
2	2	3	0	2	0	0	1	1	1	1	1	1	5	6	7	7	3	0	0	0	0	0
2	2	3	2	1	1		0	1	1	1		1	6	7	7		2	0	0	0		1
2	3	1	3	1	1	2		2	1	1	1		2	3	7	7		1	1	0	0	
2	2	3	1	4				1	3				5	5				0	0			
2	2	2	4					3														
2	2	3	1	3	6	0		1	2	3	1		4	6	6	1		0	0	0	1	
2	2	3	0		1			1		1			4		2	3		0		1	1	
2	3	3	0	2	0	2	2	1	1	1	1	1	4	5	7	8	3	0	0	0	0	0
2	2	3	0	2	0	0	0	1	1	1	1	1	6	5	7	9	3	0	0	0	0	0
3	1	2	0	0	2			1	1	1			6	5	3			0	0	1		
2	2	3	0	0	0			1	1	1			4	4	7	8		0	0	0	0	
2	2	3	3	2	3			2	1	2			4	4	5			0	0	0		
2	2	3	0	2	2	1		1	1	1	1		7	3	7	8		0	1	0	0	
2	2	3	2					1					4					0				
2	1	3	3					2					3					1				
2	2	3	2					1					2					1				
1	2	2	3	1	1			2	1	1			3	4	5			1	0	0		
2	2	1	0					1					1					1				
2	2	3	0	2	1	1	0	1	1	1	1	1	4	6	5	6	7	0	0	0	0	0
2	3	3	2	7	3			1	3	2			4	2	2			0	1	1		
2	2	1	4					3					2					1				
2	2	3	3	3	3			2	2	2			4	5	2			0	0	1		
2	2	3	0	1	3	0	2	1	1	2	1	1	7	6	7	9	7	0	0	0	0	0
3	1	3	0	0	3	2	1	1	1	2	1	1	4	3	2		1	0	1	1		1
1	1	1	1	3	0			1	2	1			2	3	7			1	1	0		
2	2	3	0	2	0	0		1	1	1	1		4	4	7	7	4	0	0	0	0	0
2	3	3	2		2	1	0	1		1	1	1	6		7	9	6	0		0	0	0
2	3	3	2	1	0		6	1	1	1		3	7	7	7	9	3	0	0	0	0	0
2	2	3	0	3	0	1	1	1	2	1	1	1	5	3		1	1	0	1		1	1
2	2	3	0					1					5					0				
2	2	3	5	1				3	1				5	4				0	0			
2	2	3	0	0	0		0	1	1	1		1	7	7	5		5	0	0	0		0
2	2	3	0	2	2		1	1	1	1		1	4	6	4		4	0	0	0		0
2	3	3	1	1	2	3	1	1	1	1	2	1	6	6	3	5	5	0	0	1	0	0
2	2	3	0		0	1	2	1		1	1	1	3		7	3	3	1		0	1	0

2	2	3	1	1	0			1	1	1			4	4	5			0	0	0		
2	2	2	2	0	1			1	1	1			2	5	7			1	0	0		
1	1	1	1	1		2		1	1		1		6	2		4		0	1		0	
2		1	1					1														
2	2	3	1		2			1		1			5		7			0		0		
1	2	2	0					1					4					0				
3	2		0	0	0	1	2	1	1	1	1	1	1	7	7		1	1	0	0		1
1	2	3	1	3	2	5		1	2	1	3		3	4	5			1	0	0		
2	2	2	0	1				1	1				4	4				0	0			
2	2	3	0	2	2		2	1	1	1		1	5	2	3	8	6	0	1	1	0	0
2	2	3	0	0	1			1	1	1			4	5	1			0	0	1		
2	3	3	2	2	0	1	0	1	1	1	1	1	6	4	4		5	0	0	0		0
2	3	3	0					1					3					1				
2	2	3	0					1					4					0				
2	2	3	1	0	1		1	1	1	1		1	5	4	6		5	0	0	0		0
2	2	3	0	3	0			1	2	1			3	2	7			1	1	0		
2	2	3	5	3	4	2	1	3	2	3	1	1	4	4	4	3	4	0	0	0	1	0
2	3	3	1	2				1	1				5	6				0	0			
2	2	3	3		2	3		2		1	2		4		5	1		0		0	1	
2	2	3	0					1					4					0				
1	2	4	3					2					2					1				
2	2	3	0	2	0	0		1	1	1	1		5	4	5	4		0	0	0	0	
2	3	3	0	1	1	1	1	1	1	1	1	1	5	5	7	8	6	0	0	0	0	0
2	2	3	0	1	0			1	1	1			4	3	7			0	1	0		
3	3	3	1	1	2			1	1	1			5	3	7			0	1	0		
2	2	3	1		3	2		1		2	1		4		7	3		0		0	1	
2	2	3	0	0	0			1	1	1			3	4	7			1	0	0		
2	2	3	3	3	1			2	2	1			5	5	6	6		0	0	0	0	
3		3	1					1					4					0				
2	2	3	0	1	0	1	2	1	1	1	1	1	5	6	7	2	3	0	0	0	1	0
2	2	3	0	0	2	3		1	1	1	2		4		3	1		0		1	1	
2	2	3	0	4	2	0	0	1	3	1	1	1	5	4	4	3	1	0	0	0	1	1
3	3	3	3					2					6					0				
2	2	3	0	0	0	1	0	1	1	1	1	1	4	3	2	3	2	0	1	1	1	1
2	2	3	0		0	0	0	1		1	1	1	6		6	2	5	0		0	1	0
1	1	1	2					1					2					1				
1	2	1	2	0	3			1	1	2				6	4				0	0		
2	2	3	1	2	4	3		1	1	3	2		3	7	4	2		1	0	0	1	
2	2	3	0					1					5					0				
2	2	3	1	1	1	1	8	1	1	1	1	3	5	7	7	8	7	0	0	0	0	0
2	2	1	4	4	6	3	2	3	3	3	2	1	1	4	3	1	4	1	0	1	1	0

2	2	3	1	1	0			1	1	1			5	6	6		4	0	0	0		0
2	2	3	0	1	2	3	0	1	1	1	2	1	4	5	7	8	6	0	0	0	0	0
2	2	3	0	1	0			1	1	1			4	7	6	5		0	0	0	0	
2	2	3	0	2	0	1	1	1	1	1	1	1	7	6	7	7	5	0	0	0	0	0
2	2	4	1					1					3					1				
2	2	3	0	0	0			1	1	1			7	5	6	3		0	0	0	1	
2	2	3	1	2	1	2	0	1	1	1	1	1	4	7	7	9	7	0	0	0	0	0
2	1		3	0	0	4		2	1	1	3		4	2	2	1		0	1	1	1	
2	2	3	2	4	2		0	1	3	1		1	4	5	6	5	4	0	0	0	0	0
2	2	3	3	2	1	1	2	2	1	1	1	1		5	3	1	4		0	1	1	0
2	3	3	3	2	1			2	1	1			6	7	7			0	0	0		
2	1	3	1	3				1	2				4	3				0	1			
3	2	3	1		1			1		1			4		7			0		0		
2	2	3	0	0	0			1	1	1			4	5	7			0	0	0		
2	2	3	0	1	0	5	1	1	1	1	3	1	5	3	7	8	3	0	1	0	0	0
2	2	3	1	1	4	3	1	1	1	3	2	1	3		6	8	5	1		0	0	0
1		2	1	2	3	4		1	1	2	3		7	7	4	5		0	0	0	0	
2	1	2	1	0	4	3	2	1	1	3	2	1	2	3	6		4	1	1	0		0
1	2	3	0	2				1	1				4	6				0	0			
2	2	3	2	2	1			1	1	1			4	3		7		0	1		0	
1	2	1	1	4	1			1	3	1			3	2	7	6		1	1	0	0	
1	1	1	3		1			2		1			2		4	8		1		0	0	
2	1	1	0					1					2					1				
1	2	1	4	3	2		0	3	2	1		1	2	2	4	3	4	1	1	0	1	0
2	3	3	0	0	3	3	0	1	1	2	2	1	4	5	6	7	4	0	0	0	0	0
2	2	3	0	0	0			1	1	1			5	4	5			0	0	0		
2	2	3	2					1					5					0				
1	2	2	3	0				2	1				3	5				1	0			
2	3	3	0	2	0	4	7	1	1	1	3	3	4	2	3	2	1	0	1	1	1	1
2	3	2	1	0		1		1	1		1			3					1			
1	2	3	4	4	4	4	1	3	3	3	3	1	3	2	6	7	2	1	1	0	0	1
2	3	3	1	0	0	0	0	1	1	1	1	1	5	6	7	5	4	0	0	0	0	0
2	2	3	2					1					4					0				
2	2	3	1			4		1			3		3			9		1			0	
2	2	3	0	1	3	2	2	1	1	2	1	1	5	7	7	2	4	0	0	0	1	0
2	2	3	0					1					5					0				
1	2	2	1					1					5					0				
1	2	3	1		0			1		1			4		7			0		0		
2	3	3	0	1	3	2		1	1	2	1		7	7	7	9	4	0	0	0	0	0
2	1	3	2		1	1		1		1	1		4		5	7		0		0	0	
2	3	3	0	0	1			1	1	1			4	3	3			0	1	1		

2	2	3	6	2	2	2	1	3	1	1	1	1	3	5	7	7	5	1	0	0	0	0
1	2	2	2		0	2		1		1	1		5		7			0		0		
2	2	1	2					1					3					1				
2	2	3	1	1	1	2	1	1	1	1	1	1	5	6	4	5	6	0	0	0	0	0
2	2	3	0	1	1	1	0	1	1	1	1	1	5	5	5	7	4	0	0	0	0	0
2	2	3	0	1	2	4	2	1	1	1	3	1	5	2	3	4	2	0	1	1	0	1
2	2	3	0	0	1			1	1	1			5	5	5			0	0	0		
2	2	3	1	0	3	3	0	1	1	2	2	1	4	4	3	2	3	0	0	1	1	0
2	2	3	0		0	2		1		1	1		4		3	3		0		1	1	
1	2	1	4	3	2	0	0	3	2	1	1	1	4	2	5	6	7	0	1	0	0	0
2	2	3	2					1					5					0				
1	2	2	0	0	1	1		1	1	1	1			2	6	3			1	0	1	
2	2	3	2	3	1		0	1	2	1		1	4	3	4		5	0	1	0		0
2	3	3	1					1														
2	2	3	0	1	1			1	1	1				7	7				0	0		
2	2	2	0	0	1	4	1	1	1	1	3	1	5	5	4	3	1	0	0	0	1	1
1	2	3	2		2		1	1		1		1	3		2		4	1		1		0
1	1	3	1	2	3	1		1	1	2	1		4	3	5	4		0	1	0	0	
2	2	3	1	1	0			1	1	1				3	5				1	0		
2	2	3	0	0	0	2	6	1	1	1	1	3	3		4		4	1		0		0
2	2	4	1	0	3			1	1	2			3	2		7		1	1		0	
2	1	3	3	3	1	6	2	2	2	1	3	1	3	3	4	2	1	1	1	0	1	1
1	2	2	3					2					2					1				
2	2	3	0	0	0	1	2	1	1	1	1	1	5	4	5	5	4	0	0	0	0	0
2	2	3	1	2	0	1		1	1	1	1		3	7	7	7		1	0	0	0	
2	2	3	0	0	1	0		1	1	1	1		4	3	4	4		0	1	0	0	
2	2	3	1	3	1	1		1	2	1	1		4	4	3	4		0	0	1	0	
2	2	3	0	0		0	5	1	1		1	3	4	4		3	3	0	0		1	0
2	2	2	1	1	1		0	1	1	1		1	4	5	5		3	0	0	0		0
2	2	1	2	2	6	2	0	1	1	3	1	1	5	4	7	7	5	0	0	0	0	0
1	2	2	1	1		5	2	1	1		3	1	3	3		2	1	1	1		1	1
2	1	2	4	2	1			3	1	1			4	3	6			0	1	0		
2	2	1	4	4	0			3	3	1			4	7	6	4		0	0	0	0	
2	2	3	0	1	1		1	1	1	1		1	4	5	5	3		0	0	0	1	
2	2	3	3	2	1	1		2	1	1	1		4	4	6	4	4	0	0	0	0	0
2	2	3	1					1					6					0				
2	2	3	0	0	0	0	4	1	1	1	1	3	4	5	6	8	1	0	0	0	0	1
2	3	3	0	2	2	1	0	1	1	1	1	1	5	6	5	7	6	0	0	0	0	0
2	3	3	1	1	3	1	0	1	1	2	1	1	5	6	6	8	5	0	0	0	0	0
2	2	3	0	0	0	2		1	1	1	1		5	3	5	3		0	1	0	1	
2	3	3	1	3	2	1		1	2	1	1		3	5	6	8		1	0	0	0	

2	1	3	0	2	0	0		1	1	1	1		4	4	4	8		0	0	0	0	
2	3	3	0					1					6					0				
2	2	3	1					1					4					0				
2	3	3	2	4	3			1	3	2			4	5	2	3		0	0	1	1	
2	2	3	0	0	0	1	1	1	1	1	1	1	7	5	6	6	2	0	0	0	0	1
2	2	3	1		3			1		2			4		3	4		0		1	0	
2	2	3	0					1					3					1				
2	3	3	0	4	1			1	3	1			4	7	4			0	0	0		
2	2	4	1		1	0		1		1	1		5		5	3		0		0	1	
1	2	2	3		2			2		1			2		3			1		1		
2	2	3	0	1	2			1	1	1			3	4	5			1	0	0		
2	2	3	0	0	0			1	1	1				2	5				1	0		
2	3	3	2					1					3					1				
2	2	3	0	2	0	1		1	1	1	1		6	4	7	5		0	0	0	0	
1	1	3	0	1		4		1	1		3		3	5				1	0			
2	2	1	3	2	1	2		2	1	1	1		6	7	7	8		0	0	0	0	
2	2	3	0	0	2			1	1	1			6	1	3			0	1	1		
2	3	3	3	2				2	1				3	3				1	1			
2	3	3	1	2	1			1	1	1			5	4	6			0	0	0		
2	2	3	0					1					5					0				
2	2	3	5					3					3					1				
1	3	3	0	2	0	3		1	1	1	2		3	6	7	9		1	0	0	0	
3	2	3		1	1				1	1			5	7	7	7		0	0	0	0	
2	2	3	0	1	0			1	1	1			4	5	5	6		0	0	0	0	
2	1	3	0	2	1	2		1	1	1	1		7	3	3	1		0	1	1	1	
2	2	1	0	1	1	2	1	1	1	1	1	1	4	5	7	7	3	0	0	0	0	0
2	2	3	0	1	0	3	1	1	1	1	2	1	6	7	7	9	1	0	0	0	0	1
2	3		2					1					4					0				
2	1	3	0	2	1			1	1	1			5	7	6			0	0	0		
2	2	3	3	5	4	0	1	2	3	3	1	1	3	4	4	1	4	1	0	0	1	0
2	2	2	4	2	4	2		3	1	3	1		4	3	5	4		0	1	0	0	
2	3	3	2	1				1	1				4	7				0	0			
1	2	1	2	1				1	1				2	4				1	0			
2	2	3	0	3	5	1	3	1	2	3	1	2	4	6	4	7	1	0	0	0	0	1
1	2	1	1	2				1	1				2	7				1	0			
2	3	3	3	2	2	0	0	2	1	1	1	1	5	5	7	7	4	0	0	0	0	0
1	2	2	0	3	1	2		1	2	1	1		3	5	7	7	7	1	0	0	0	0
2	2	3	1	0	1	2	0	1	1	1	1	1	6	5	5	2	2	0	0	0	1	1
2	2	3	0	1	0	1		1	1	1	1		6	6	7	7		0	0	0	0	
2	2	3	1	0	1	1		1	1	1	1		4	3	4	4		0	1	0	0	
2	2	4	4	5	4	3	3	3	3	3	2	2	4	4	4	2	1	0	0	0	1	1

2	2	3	2	5	3		1	1	3	2		1	4	4	6		6	0	0	0		0
2	2	3	2	4				1	3				4	7		3		0	0		1	
2	2	3	0	2	3	0		1	1	2	1		6	4	7	6		0	0	0	0	
2	2	1	6	3	1			3	2	1			4	5	4			0	0	0		
3	1	2	0	1	1	2	0	1	1	1	1	1	5	4	2	1	3	0	0	1	1	0
2	3	3	2	2	0	1		1	1	1	1			7	7	6			0	0	0	
2	2	3	0	1	1	0		1	1	1	1		5	7	7	9		0	0	0	0	
2	3	1	2	2		3		1	1		2		4	5		7		0	0		0	
2	3	4	1	0				1	1				1	2				1	1			
2	1	3	0	1	0			1	1	1			5	5	6	4		0	0	0	0	
2	3	3	0	1	1	0	1	1	1	1	1	1	4	4	5	5	6	0	0	0	0	0
2	3	3	3	0		2		2	1		1		1			6		1			0	
2	2	2	1	1	0	1	0	1	1	1	1	1	4	5	4	5	5	0	0	0	0	0
2	2	3	0	0	1	1		1	1	1	1		4	6		1		0	0		1	
2	2	3	1	1	0	1		1	1	1	1		5	6	7	9		0	0	0	0	
2	1	3	1	2	1	1		1	1	1	1		4	4	3	4		0	0	1	0	
2	2	3	0	1	3	2	1	1	1	2	1	1	4	4	4	7	7	0	0	0	0	0
2	2	3	3	3	2			2	2	1			4	4	2			0	0	1		
2	3	3	0	1	1	0	0	1	1	1	1	1	5	5	5	8	7	0	0	0	0	0
2	2	3	0	0				1	1				5	7				0	0			
2	3	3	0					1					4					0				
2	1	3	1	1	1	5	1	1	1	1	3	1	5	6	3	2	3	0	0	1	1	0
2	3	3	3	4	3	1		2	3	2	1		4	4	4	9		0	0	0	0	
3	1	2	0					1					4					0				
1	2	2	0		1	0		1		1	1		3		7	1		1		0	1	
2	3	3	1					1					4					0				
2	2	3	0	1	0	0	1	1	1	1	1	1	4	7	7	8	5	0	0	0	0	0
3	3	3	0	2				1	1				6	7				0	0			
1	2	1	0	2	1			1	1	1			4	7	7			0	0	0		
2	1	3	0					1					5					0				
2	3	3	2	2	0	0	0	1	1	1	1	1	1	6	5	4	5	1	0	0	0	0
2	2	3	2		2			1		1			4		7			0		0		
2	1	3	2	1	0	1	1	1	1	1	1	1	5	4	6	2	3	0	0	0	1	0
2	2	3	0	1	0	1	0	1	1	1	1	1	5	6	6	8	5	0	0	0	0	0
2	2	3	2					1					4					0				
2	3	3	1					1					4					0				
2	2	3	1					1					4					0				
1	1	3	2		1	2		1		1	1		4		3			0		1		
2	1	3	1	2				1	1				4	3				0	1			
2	1	2	1	1				1	1				3	5				1	0			
2	2	2	2					1					4					0				

2	2	3	0	2	4			1	1	3			4		2	2		0		1	1	
2	2	3	0	1	3	3	1	1	1	2	2	1	6	5	5	6	5	0	0	0	0	0
2	2	3	0	1	4			1	1	3			4	7	5			0	0	0		
2	2	2	0			4		1			3		4					0				
2	3	3	1	2	2	0	0	1	1	1	1	1	5	6	5		4	0	0	0		0
2	3	3	0	2	1	2		1	1	1	1		6		7	6	4	0		0	0	0
2	2	3	2	0	2	1	0	1	1	1	1	1	3	3	3	8	3	1	1	1	0	0
2	3	3	1	1	1	1		1	1	1	1		4	6	7	9		0	0	0	0	
2	2	2	1					1					1			1		1			1	
2	2	2	2	1	2	6		1	1	1	3		3	4	4	1		1	0	0	1	
1	2	3	1	1	2	1	0	1	1	1	1	1	4	5	5	4	6	0	0	0	0	0
2	1	3	0	0	0	1		1	1	1	1		4	3	4			0	1	0		
2	2	1	3	2	4	5	3	2	1	3	3	2	6	4	3	4	3	0	0	1	0	0
2	2	3	0	0	0	0		1	1	1	1		4	7	7	8		0	0	0	0	
2	2	3	6	3	1		0	3	2	1		1	2	3	4	2	7	1	1	0	1	0
2	2	3	0		1	2		1		1	1		5		4	3		0		0	1	
2	2	4	2	2	1	4	1	1	1	1	3	1	2	5	5		1	1	0	0		1
1	2	3	0	3	2	1	0	1	2	1	1	1	3	6	7	7	7	1	0	0	0	0
1	2	3	1	3	0			1	2	1			3	4	7			1	0	0		
2	1	3		4	4	4	2		3	3	3	1	4	5	4	4	3	0	0	0	0	0
1	2	1	0					1					6					0				
2	2	3	0	0	0	0	0	1	1	1	1	1	5	7	7	9	7	0	0	0	0	0
2	2	3	0	4	1			1	3	1			4	4	7	3		0	0	0	1	
2	2	3	2	1	0	1	0	1	1	1	1	1	7	5	7	8	1	0	0	0	0	1
2	2	3	3	1	0	1	0	2	1	1	1	1	4	6	3	1	3	0	0	1	1	0
1	2	3	1			1		1			1		3					1				
2	2	1	1	2				1	1				3	4				1	0			
2	1	1	0			3		1			2		2			4		1			0	
2	2	3	0	0	1	3	2	1	1	1	2	1	7	5	7	7	4	0	0	0	0	0
2	2	3	0	0	1			1	1	1			5	4	5			0	0	0		
2	3	3	3	3	4	2	0	2	2	3	1	1	4	7	7	9	7	0	0	0	0	0
2	2	3	2	1	0	0	1	1	1	1	1	1	3	6	7	2	1	1	0	0	1	1
2	2	3	1			2		1			1		3					1				
2	2	3	2	0	0	1	0	1	1	1	1	1	5	6	7		6	0	0	0		0
2	2	3	3					2					3					1				
2	3	3	0	1	0	1	0	1	1	1	1	1	4	5	7	8	2	0	0	0	0	1
2	2	3	0	2	0			1	1	1			5	5	6			0	0	0		
2	2	3	3					2					5					0				
2	2	3	0					1					5					0				
2	2	3	0	2	0	1	8	1	1	1	1	3	7	6	4	6	4	0	0	0	0	0
3	2	3	5					3					6					0				

2	3	3	1			2	1	1			1	1	7			5	3	0			0	0
2	2	3	0	2	4	0		1	1	3	1		4	2	3	1		0	1	1	1	
2	2	3	0		0	1		1		1	1		4		6	7		0		0	0	
2	2		0	2	1	1		1	1	1	1			4	4	4			0	0	0	
2	2	3	2	1	4	3		1	1	3	2		7	7	3	4		0	0	1	0	
2		3	1	4	0		0	1	3	1		1	5	5	4	7	5	0	0	0	0	0
2	2	3	0	1	1			1	1	1			4	3	3			0	1	1		
2	2	3	0	0	1			1	1	1			5	4	4			0	0	0		
2	2	3	1	3	1	3		1	2	1	2		5	3	3	2		0	1	1	1	
2	2	3	0	2	1	2	1	1	1	1	1	1	6	6	7	9	5	0	0	0	0	0
2	2	3	0	1	0	7		1	1	1	3		4	5	7	3		0	0	0	1	
2	2	3	1	1	1	0	1	1	1	1	1	1	7	7	7	8	4	0	0	0	0	0
2	3	3	0	0	1	1	0	1	1	1	1	1	3	7	7	7	7	1	0	0	0	0
2	2	2	1	4	0		0	1	3	1		1	6	3	4	8	4	0	1	0	0	0
2	1	3	1	3	3	2	0	1	2	2	1	1	3	2	5	5	3	1	1	0	0	0
2	2	3	3	2	0	5	0	2	1	1	3	1	3	3	3	6	1	1	1	1	0	1
2	2	3	1	3	1	7		1	2	1	3		4	3	2	2		0	1	1	1	
2	2	3	0	2	1			1	1	1			6	4	4			0	0	0		
2	2	3	1	4	3		6	1	3	2		3	3	6	5	6	1	1	0	0	0	1
2	1	3	1	2	3	3		1	1	2	2			3	1	6			1	1	0	
2	2	3	1	2	0	3	0	1	1	1	2	1	3	4	6	7		1	0	0	0	
2	2	3	3	0	1	1	0	2	1	1	1	1	5	7	7	8	3	0	0	0	0	0
1	2	3	3	4	2			2	3	1			2	4	4			1	0	0		
2	3	3	2	1	2	2	1	1	1	1	1	1	5	5	7		4	0	0	0		0
2	2	2	2	2				1	1				3	4				1	0			
1	2		4	1		5		3	1		3		2	3		1		1	1		1	
2	3	1	0	1	0	1	2	1	1	1	1	1	4	2	5	7	3	0	1	0	0	0
2	2	3	0	2	1	2		1	1	1	1		4	4	7	4		0	0	0	0	
2	1	3	0	0	0	1	0	1	1	1	1	1	5	5	7	5	3	0	0	0	0	0
2	3	3	2					1					4					0				
2	2	3	0	1	0			1	1	1			4	4	7			0	0	0		
2	2	3	1	1				1	1				6	4				0	0			
2	2	3	2	0				1	1					2					1			
1	2	3	4					3					3					1				
2	2	3		1	2	3			1	1	2		4	7				0	0			
2	2	3	2	2	2	2		1	1	1	1		3	7	7	2		1	0	0	1	
2	2	1	3					2					3					1				
2	2	3	1					1					4					0				
3	3	3	1	0	0	1	0	1	1	1	1	1	5	5	4	1	5	0	0	0	1	0
2	2	3	0	2	0			1	1	1			4	3	2			0	1	1		
2	1	3	0	0	0		0	1	1	1		1	4	4	7			0	0	0		

2	3	2	2					1					4					0				
1	2	4	1					1					4					0				
2	2	2	1		0			1		1			6		4			0		0		
2	2	3	0					1					4					0				
1	1	3	1	1	0	0	1	1	1	1	1	1	5	7	7	9	6	0	0	0	0	0
1	3	2	5	2	1			3	1	1			4	7	3			0	0	1		
2	2	3	6	5	3	4	2	3	3	2	3	1	4	5	5	7	1	0	0	0	0	1
2	2	3	1	3	4	3	1	1	2	3	2	1	4	6	5	4	5	0	0	0	0	0
2	3	3	0	3	2	0	0	1	2	1	1	1	6	5	6	8	3	0	0	0	0	0
2	2	3	1		3			1		2			5		5			0		0		
2	2	3	1	1	0	2	1	1	1	1	1	1	4	7	7	8	4	0	0	0	0	0
2	2	3	0	0	0	0	0	1	1	1	1	1	4	7	6	8	2	0	0	0	0	1
2	2	1	3	3	4		1	2	2	3		1	2	4	4	4	5	1	0	0	0	0
2	2	3	2					1					6					0				
2	1	3	0	4	1	2	0	1	3	1	1	1	4	3	4	3	7	0	1	0	1	0
2	2	3	0		0		0	1		1		1	4		7	2	3	0		0	1	0
2	3	3	1	2	1		1	1	1	1		1	4	4	5	4	4	0	0	0	0	0
1	2	1	3					2					3					1				
2	2	3	2	3	2	3	5	1	2	1	2	3	3	1	5	5	3	1	1	0	0	0
2	1	3	1	0	0	0		1	1	1	1		5	3	4	3		0	1	0	1	
2	2	3	0	0	1	2		1	1	1	1		4	4	3	4		0	0	1	0	
2	3	1	1	1	0	3		1	1	1	2		3	5	7	9		1	0	0	0	
1	2	3	0					1					4					0				
2	2	3	2	2	4	2	1	1	1	3	1	1	5	4	3	3	6	0	0	1	1	0
1	2	3	4		5			3		3			3		3			1		1		
2	2	3	0	0	0		4	1	1	1		3	6	4	6		3	0	0	0		0
2	2	2	0	2	3	0		1	1	2	1		3	5	3	4		1	0	1	0	
2	2	3	1		0	1		1		1	1		7		7			0		0		
2	3	3	1		1	1		1		1	1		5		7	8		0		0	0	
2	2	3	2	4	2	1	1	1	3	1	1	1	3	5	4	8	7	1	0	0	0	0
2	2	3	1	2	0			1	1	1			5	5	3			0	0	1		
2	2	3	2		2	3		1		1	2		5		4			0		0		
2	3	3	0	2	1	1	3	1	1	1	1	2	5	4	7	4	6	0	0	0	0	0
2	2	3	1	1	0	0	2	1	1	1	1	1	5	5	7	8	7	0	0	0	0	0
2	2	3	0					1					6					0				
2	2	3	0	1	0	2	0	1	1	1	1	1	4	7	5	9	5	0	0	0	0	0
2	3	3	3	1	0			2	1	1			3	3	3			1	1	1		
1	2	2	5					3					5					0				
1	2	3	2	4	2	2		1	3	1	1		5	4	2	7		0	0	1	0	
2	2	3	1	0	1	0	0	1	1	1	1	1	7	6	7	8	6	0	0	0	0	0
2	2	3	1	3	0	5		1	2	1	3			4	3	4			0	1	0	

2	2	3	0		1	2	0	1		1	1	1	3		7	4	3	1		0	0	0
2	1	3	1	3	0			1	2	1			4	7	7			0	0	0		
1	2	3	0	1	0			1	1	1			4	5	7			0	0	0		
2	2	3	0	0	0	0		1	1	1	1		4	5	7	9		0	0	0	0	
2	2	3	0	0	1			1	1	1			4	4	7		4	0	0	0		0
2	3	3	0	0	0	1	0	1	1	1	1	1	4	7	5	3	4	0	0	0	1	0
2	1	1	2					1					5					0				
2	3	3	1	1	3			1	1	2			7	7	7	9		0	0	0	0	
2	2	1	1					1					4					0				
2	2	3	0	3				1	2				4	3				0	1			
2	2	3	0	1	0			1	1	1			4	5	7			0	0	0		
1	2	1	4	4	5	1		3	3	3	1		1	3	1	3		1	1	1	1	
2	1	3	1	0	0	3	0	1	1	1	2	1	4	3	4	2	1	0	1	0	1	1
1	1	3	4	5	2			3	3	1			2	3	1	2		1	1	1	1	
2	2	3	0	2	0	2		1	1	1	1		4	7	7	8		0	0	0	0	
3	2	2	4					3					4					0				
1	2	2	1					1					3					1				
2	2	3	1					1					3					1				
1	2	1	0		3	2		1		2	1		3			3		1			1	
2	1	2	0	0	0	2		1	1	1	1		4	3	2	1		0	1	1	1	
1	1	1	2	0	4			1	1	3				2	2				1	1		
1	2	3	4					3					5					0				
2	1	3	0	0				1	1				4	5				0	0			
2	3	3	2					1					3					1				
1	3	3	5	4	1	0		3	3	1	1		4	3	7	8		0	1	0	0	
2	2	3	5	0		4		3	1		3		3	4				1	0			
2	1	3	5	5	2	2	1	3	3	1	1	1	3	2	5	6	6	1	1	0	0	0
2	3	3	1	3	4	1	0	1	2	3	1	1	4		3	8	3	0		1	0	0
2	2	3	3	1	3	2	3	2	1	2	1	2	6	7	7	9	5	0	0	0	0	0
2	2	3	4	1	0	1	1	3	1	1	1	1	3	6	7	8	4	1	0	0	0	0
2	1	2	2	2				1	1					2					1			
1	2	3	1	1	0	1	0	1	1	1	1	1	4	7	7	9	7	0	0	0	0	0
2	2	3	1	0	0	0		1	1	1	1		5	4	4	5		0	0	0	0	
2	2	3	0	2	0	1		1	1	1	1		3	3	5	8		1	1	0	0	
1	2	3	2	3	3	0	0	1	2	2	1	1	7	6	5	7	7	0	0	0	0	0
2	2	3	2	1	0	0	4	1	1	1	1	3	4	7	7	7	3	0	0	0	0	0
2	1	3	1	0	0			1	1	1			3	6	7	8		1	0	0	0	
2	1	3	2	1	1			1	1	1			4	4	5			0	0	0		
2	2	3	1	1	1	2		1	1	1	1		5	3	4	2		0	1	0	1	
2	3	3	2	2	0	1	2	1	1	1	1	1	6	5	7	8	7	0	0	0	0	0
2	1	3	2	3	5			1	2	3			4			1		0			1	

1	1	3	2					1					3					1				
2	2	3	1					1					3					1				
2	3	3	0	1	0	0	0	1	1	1	1	1	4	5	5	4	3	0	0	0	0	0
2	1	1	2					1					4					0				
1	2	3	1		4	4		1		3	3		3		3	3		1		1	1	
2	1	2	3	1	2	3	1	2	1	1	2	1	3	6	7	8	3	1	0	0	0	0
1	2	3	0	1				1	1				7	4				0	0			
2	2	3	0	0	0	2	1	1	1	1	1	1	4	7	7	7	5	0	0	0	0	0
2	2	3	0	1	1			1	1	1			4	6	6	4		0	0	0	0	
2	2	2	0	2	2		2	1	1	1		1	4	6	3	3	3	0	0	1	1	0
2	3	3	0	1	0	2		1	1	1	1		4	3	4	3		0	1	0	1	
2	3	3	0	0	0	1	0	1	1	1	1	1	3	4	5	5	6	1	0	0	0	0
1	1	3	1	0	2			1	1	1			3	4	3			1	0	1		
2	2	1	2		0			1		1			4		7			0		0		
3	2	3	1					1					4					0				
2	3	3	1					1														
1	1	1	0	3	2			1	2	1			1	5	5			1	0	0		
3	1	3	1	2	0	1	3	1	1	1	1	2	5	3	1	2	3	0	1	1	1	0
2	2	3	0	0	1	3	3	1	1	1	2	2	4	4	3	2	2	0	0	1	1	1
2	3	2	3					2					6					0				
2	1	3	1	0	2			1	1	1			4	4	4			0	0	0		
3	3	2	0	1	1	0	0	1	1	1	1	1	6	7	7	8	6	0	0	0	0	0
2	3	3	2					1					3					1				
2	3	3	3					2					5					0				
1	1	1	3	4	5	1	1	2	3	3	1	1	6	4	3	2	6	0	0	1	1	0
1	2	4	4		2	3	6	3		1	2	3	4		5	7	1	0		0	0	1
2	2	3	2	3	5			1	2	3			3	3	4	4		1	1	0	0	
2	2	3	0	1	3			1	1	2				7	3				0	1		
2	2	3	1	0				1	1					3					1			
3	2	3	0					1					5					0				
2	2	3	0	0	1	2	0	1	1	1	1	1	5	5	7	8	5	0	0	0	0	0
2	2	3	0	3	0	3		1	2	1	2		5	4	7	8		0	0	0	0	
1	2	3	0	0	0			1	1	1			5	4	5			0	0	0		
2	2	3	1					1					5					0				
1	3	1	0	4				1	3				4					0				
1	2	3	1	1	0	0	2	1	1	1	1	1		6	6	9	3		0	0	0	0
2	2	3	0	1	0	2		1	1	1	1		4	5	7			0	0	0		
2	3	2	0	0	2	1	1	1	1	1	1	1	4	5	7	8	3	0	0	0	0	0
1	2	3	0	0	1	0	0	1	1	1	1	1	3	6	6		4	1	0	0		0
2	3	3	1	3	1			1	2	1			4	7	1	3		0	0	1	1	
2	2	3	0	0				1	1				4	6				0	0			

3	3	3	1	1	1			1	1	1			4	3	3			0	1	1		
2	2	3	0		0	1		1		1	1		4					0				
2	2	3	0	1	0			1	1	1			4	6	7			0	0	0		
2	1	3	1					1					3					1				
2	2	1	0	0	0	0	2	1	1	1	1	1	4	6	7	7	1	0	0	0	0	1
2	1	3	2	2	1			1	1	1			3	4	7			1	0	0		
2	2	2	2	1	4			1	1	3			3	4	6	4		1	0	0	0	
2	2	3	2					1					5					0				
2	1	1	0					1					2					1				
2	3	3	0	2	0	0	0	1	1	1	1	1	6	6	7	5	7	0	0	0	0	0
2	2	3	1	2	1		0	1	1	1		1	5	5	3	2	3	0	0	1	1	0
1	2	1	3	1	5	2	3	2	1	3	1	2	3	2	2		6	1	1	1		0
1	2	3	1		0	1		1		1	1		6		7	9		0		0	0	
2	3	3	2		2	2	4	1		1	1	3	3		5	1	5	1		0	1	0
2	1	3	5	1	0			3	1	1			5	6	7			0	0	0		
1	1	3	3		0	2	2	2		1	1	1	3		6	8	1	1		0	0	1
2	2	2	0					1					4					0				
2	2	2	0	0				1	1				5	2				0	1			
2	2	2	3	4	1	4		2	3	1	3		3	2	5	1		1	1	0	1	
1	1	3	5		8	2		3		3	1		3		4	2		1		0	1	
2	2	3	3	0				2	1				4	4				0	0			
3	2	3	1	1	0	1	0	1	1	1	1	1	4		7	7	1	0		0	0	1
2	2	3	3		4			2		3			4		2			0		1		
2	2	3	1	1				1	1				4	5				0	0			
2	3	3	0	2	4	2		1	1	3	1		4	4	3	3		0	0	1	1	
1	2	2	4	1				3	1				2	6				1	0			
1	2	1	4					3					4					0				
2	2	3	3	2	4	2	1	2	1	3	1	1	7	4	6	6	3	0	0	0	0	0
2	2	3	0	4	0	3		1	3	1	2		4	4	4	5		0	0	0	0	
1	2	3	0	1	1	1		1	1	1	1		4	5	4	2		0	0	0	1	
2	3	2	1	0	0	3		1	1	1	2		3	2	6	5		1	1	0	0	
2	3	3	1	3	1			1	2	1			4	5	5	7		0	0	0	0	
2	2	3	1	1	0	2	0	1	1	1	1	1	4	6	7	9	6	0	0	0	0	0
2	2	3	1	2	0	0	1	1	1	1	1	1	5	6	6	8	3	0	0	0	0	0
2	3	3	1					1					3					1				
2	3	3	4	4	5	1	1	3	3	3	1	1	4	4	5	5	3	0	0	0	0	0
2	2	3	2	4	2			1	3	1			7	5	5	6		0	0	0	0	
2	1	3	0	0	2	0	1	1	1	1	1	1	4	7	7	1	7	0	0	0	1	0
2	3	3	0	2	1	1	5	1	1	1	1	3	4	4	3	3	5	0	0	1	1	0
2	2	3	1	1	1		1	1	1	1		1	3	3	3		1	1	1	1		1
2	2	3	1	1	0			1	1	1				2	7				1	0		

2	2	3	0	1	0		0	1	1	1		1	5	6	7	9	5	0	0	0	0	0
2	2	2	1	4	0			1	3	1			3	4	4			1	0	0		
2	2	2	2					1					3					1				
2	3	3	3		0	0	0	2		1	1	1	4		7	8	6	0		0	0	0
1	2	2	1		1			1		1			3		5	8		1		0	0	
2	3	3	0	2	0		0	1	1	1		1	4	3	7	9	4	0	1	0	0	0
2	2	4	1		2	0	0	1		1	1	1	3		2	2		1		1	1	
2	2	3	1	0	1	0		1	1	1	1		4	6	4	4		0	0	0	0	
3	2		0	0	0			1	1	1			5	4	7			0	0	0		
1	2	2	4	3	1			3	2	1			3		7	1		1		0	1	
2	2	2	0	0	0			1	1	1			3	3	4			1	1	0		
2	3	1	3	1	1	2	1	2	1	1	1	1	4	6	4	9	3	0	0	0	0	0
1	2	2	0		3	4		1		2	3		3		7	3		1		0	1	
2	3	3	0		1	2	3	1		1	1	2	7		7	9	1	0		0	0	1
2	2	3	0	0	0			1	1	1			3	5	7			1	0	0		
2	2	4	3	2	0	2		2	1	1	1		4	6	7	8		0	0	0	0	
1	2	3	1	0	0	0	1	1	1	1	1	1	4	5	6	8	6	0	0	0	0	0
2	2	3	3	3	1	1	1	2	2	1	1	1	2	3	6	5	3	1	1	0	0	0
2	2	3	1	1	2	5		1	1	1	3		5	7	7	8		0	0	0	0	
2	2	3	0	1	0	0	1	1	1	1	1	1	6	6	7	9	5	0	0	0	0	0
2	2	3	2	2				1	1				2	5				1	0			
2	2	2	2	0	0	2		1	1	1	1		4	6	7	8		0	0	0	0	
1	3	1	4					3					2					1				
2	2	3	3		2	1	2	2		1	1	1	5		4	8	2	0		0	0	1
2	1	3	0	1	2			1	1	1			4	3	3			0	1	1		
3	2	3	0	0	0	1		1	1	1	1		4	4	6	2		0	0	0	1	
2	1	3	0	1	1			1	1	1			5	4	4	2		0	0	0	1	
2	2	3	0	0	0	1		1	1	1	1		5	7	7	9		0	0	0	0	
1	2	3	0	4	5			1	3	3				3					1			
2	2	3	1	1	3			1	1	2			5	6	7			0	0	0		
1	1	2	1	2	1	2	5	1	1	1	1	3	1	5	6	3	5	1	0	0	1	0
2	2	3	1	3	4	3	3	1	2	3	2	2	5	6	3	3	1	0	0	1	1	1
2	2	2	2					1					6					0				
2	3	3	0	4	1	0	1	1	3	1	1	1	3	7	7	9	6	1	0	0	0	0
2	3	2	0	3				1	2				3	4	3			1	0	1		
2	2	3	1	0	0	6	0	1	1	1	3	1	4	7	7	8	1	0	0	0	0	1
1	2	3	4		1	1	0	3		1	1	1	4		5	5	3	0		0	0	0
2	2	3	3	3	4	2	3	2	2	3	1	2	3	3	2	4	1	1	1	1	0	1
1	3	1	0	2	0			1	1	1				4	7				0	0		
2	1	3	0		4			1		3			3		5			1		0		
2	1	3	0	2	6	1	1	1	1	3	1	1	5	3	2	1	1	0	1	1	1	1

2	2	3	0	1	2	4		1	1	1	3		3	1	3	3		1	1	1	1	
1	1	1	1	0	1	4	2	1	1	1	3	1	2	6	5	5	3	1	0	0	0	0
2	2	3	0	0	1			1	1	1			5	6	7			0	0	0		
2	2	3	0		0	0		1		1	1		5		6			0		0		
2	1	3	0					1					3					1				
2	2	3	1					1					3					1				
2	2	3	0	3	0			1	2	1			5	7	7	8		0	0	0	0	
1	2	2	2	3	3			1	2	2			3	4	5	6		1	0	0	0	
2	3	3	0	2	1	1		1	1	1	1		5	5	3	5		0	0	1	0	
1	2	3	3	1	0	1	0	2	1	1	1	1		4	7	9	5		0	0	0	0
1	1	1	1					1					1					1				
2	2	3	0	1	2	2	2	1	1	1	1	1		6	6		5		0	0		0
2	1	3	0					1					6					0				
2	2	3	0	0	0	0		1	1	1	1											
3		2	2		1			1		1			3		4			1		0		
1	3	1	0	2	2		3	1	1	1		2	4	4	7		1	0	0	0		1
2	2	3	2	3	0	3		1	2	1	2		4	5	6	8		0	0	0	0	
3	2	3	0					1					5					0				
2	2	3	0					1					5					0				
2	2	3	1	1	2	0	0	1	1	1	1	1	6	6	7	7	4	0	0	0	0	0
2	2	3	1	2	1			1	1	1			4	5	4	5		0	0	0	0	
2	2	3	0	1	0	2		1	1	1	1		6	4	7	8		0	0	0	0	
2	2	3	0					1					4					0				
1	1	1	5	3	2	2		3	2	1	1		3	3	5	5		1	1	0	0	
2	2	3	0	3	2			1	2	1			4	6	2			0	0	1		
2	3	3	0	2	1	0	4	1	1	1	1	3	6	6	6	8	7	0	0	0	0	0
1	1	2	4					3					7					0				
2	2	3	0	1	1		0	1	1	1		1	4	5	7		6	0	0	0		0
2	2	3	0	2	1	2	2	1	1	1	1	1	4	5	7	6	5	0	0	0	0	0
2	3	3	0	2	1	2		1	1	1	1		6	4	6	8		0	0	0	0	
2		3	0		0	2		1		1	1		4		7	8		0		0	0	
2	2	3	0	0	0	0		1	1	1	1		4	3	5	8		0	1	0	0	
2	2	3	0	1	0			1	1	1			3	4	5			1	0	0		
2	1	3	0	1	0			1	1	1			4	4	7			0	0	0		
2	1	3	2					1					4					0				
2	2	3	1	5	2	2	6	1	3	1	1	3	4	4	6	7	3	0	0	0	0	0
1	2	2	2					1					3					1				
2	2	3	0	1	1	2		1	1	1	1		4	5	4	5		0	0	0	0	
2	2	3	0			1		1			1		3			4		1			0	
1	1	1	2	1	0			1	1	1			4	7	7	9		0	0	0	0	
2	2	3	2	3	2			1	2	1			4	3	6			0	1	0		

2	3	3	1	0	0	2	1	1	1	1	1	1	4	7	7	7	4	0	0	0	0	0
2	2	3	0	0	0			1	1	1			4	5	6			0	0	0		
2	2	3	0	0	2	1	7	1	1	1	1	3	4	7	6	8	6	0	0	0	0	0
2	1	3	3	3				2	2				3					1				
2	2	3	2					1					4					0				
1	1	2	0					1														
1	2	3	3		4			2		3			3		2			1		1		
2	1	3	0	1	0	1	1	1	1	1	1	1	4	4	5	6	3	0	0	0	0	0
1	2	2	0					1					5					0				
2	1	3	3	1	0	3	0	2	1	1	2	1	4	4	7	8	7	0	0	0	0	0
2	3	3	0					1					7					0				
2	2	3	1	0	1	2		1	1	1	1			4	7	8			0	0	0	
2	2	3	3	2	2	1		2	1	1	1		4	4	5	3		0	0	0	1	
2	3	3	1	2				1	1				5					0				
2	2	3	2	3	3	3		1	2	2	2		4		2	2		0		1	1	
1	2	3	1	0				1	1				5	4		7		0	0		0	
2	2	3	0	1	0	2		1	1	1	1		4	4	6	9		0	0	0	0	
2	2	3	1	1				1	1				6					0				
2	2	3	0	1	1	1	0	1	1	1	1	1	5	7	7	8	6	0	0	0	0	0
1	2	3	5	1	2	1		3	1	1	1			6	6	4			0	0	0	
2	2	2	0					1														
2	2	3	0	1	1	1	1	1	1	1	1	1	5	4	5	2	3	0	0	0	1	0
2			2		1	3	2	1		1	2	1			4	3	3			0	1	0
2	2	1	0	0	1	1	1	1	1	1	1	1	4	3	4	2	4	0	1	0	1	0
3	3	3	0	0	1	1	3	1	1	1	1	2	6	7	7	9	2	0	0	0	0	1
2	2	3	2	0	0	2	2	1	1	1	1	1	4	7	7	4	6	0	0	0	0	0
2	2	3	2		4			1		3			4		3			0		1		
2	2	3	0	0				1	1				5	4				0	0			
2	3	3	1	0				1	1				5	5				0	0			
2	2	3	1	5	1	0		1	3	1	1		3	4	4	8		1	0	0	0	
2	3	3	0	1	2	0	1	1	1	1	1	1	5	3	3	3	3	0	1	1	1	0
2	3	3	0					1					7					0				
1	2	3	1	0	0	1		1	1	1	1		4	4	5	6		0	0	0	0	
1	1	1	0	3				1	2				3	5				1	0			
2	2	3	1	1	0	1	2	1	1	1	1	1	6	6	2	3	6	0	0	1	1	0
2	2	3	3	3				2	2				4	5				0	0			
2	2	3	5	0	3			3	1	2			4	5	2			0	0	1		
2	3	3	4	5	5	3		3	3	3	2		3	5	7	8		1	0	0	0	
2	3	2	0	1	0	2	3	1	1	1	1	2	7	7	7	9	3	0	0	0	0	0
2	1	2	2					1					4					0				
2	2	3	1		1	2	2	1		1	1	1	7		5	3	6	0		0	1	0

2	2	4	4	0	0	4	2	3	1	1	3	1	6	2	4	4	3	0	1	0	0	0
2	2	1	2	3	5			1	2	3			4	3	4			0	1	0		
2	2	3	2	4	3			1	3	2			4	7	5			0	0	0		
3	2	3	0		0	2	3	1		1	1	2	4		7	4	4	0		0	0	0
2	2		0	4	0			1	3	1			4	6	7	8		0	0	0	0	
2	2	3	4	3	1	0	1	3	2	1	1	1	3	7	7	2	4	1	0	0	1	0
2	2	3	0					1					4					0				
2	2	3	4	5	5			3	3	3			5	7	7			0	0	0		
2	2	3	1		0	1	0	1		1	1	1	3		6	3	6	1		0	1	0
2	1	1	3	3				2	2				3	2				1	1			
1	2	1	0	1	0	1	0	1	1	1	1	1	4	6	7	8	4	0	0	0	0	0
1	1	3	1		0			1		1			4		7	9		0		0	0	
2	1	3	0	1	1			1	1	1			4	6	7	2		0	0	0	1	
1	2	3	1	0	0			1	1	1			6	4	7			0	0	0		
2	2	3	1	2	0	2	0	1	1	1	1	1		6	7	3	1		0	0	1	1
2	2	3	0	1	0	0	1	1	1	1	1	1	5	7	7	8	4	0	0	0	0	0
2	2	2	1	0	0	0	0	1	1	1	1	1	4	6	6	9	5	0	0	0	0	0
2	2	3	1					1					3					1				
2	2	2	2	3	1	1	0	1	2	1	1	1	4	5	6	5	3	0	0	0	0	0
2	2	3	0	1	1	0		1	1	1	1		7	6	7			0	0	0		
2	2	1	6					3					3					1				
2	2	3	0	0				1	1					6					0			
2	2	3	0	3	5	2		1	2	3	1		4	5	3	8		0	0	1	0	
3	2	3	0	1	2			1	1	1			4	5	5			0	0	0		
2	2	3	2	1	0		0	1	1	1		1	7	5	7		6	0	0	0		0
2	3	3	1	2	0			1	1	1			5	1	7	1		0	1	0	1	
2	1	3	0					1					5					0				
2	1	3	3	0	0			2	1	1			4	5	5	6		0	0	0	0	
2	2	3	4	4	1	1	0	3	3	1	1	1	2	3	7	9	6	1	1	0	0	0
2	2	3	2					1					4					0				
2	2	3	1	2	0			1	1	1			3	4	6	6		1	0	0	0	
2	3	1	2	4	2	4		1	3	1	3		5	2	2	3		0	1	1	1	
2	2	3	0					1					4					0				
2	2	3	0	3		0	0	1	2		1	1	7	7		6	7	0	0		0	0
2	2	3	0	2	1	1	0	1	1	1	1	1		6	7	7	5		0	0	0	0
2	2	3	1	1	1	1		1	1	1	1		4	4	2	6		0	0	1	0	
1	1	2	1					1					5					0				
2	1	3	0	0	0		0	1	1	1		1	4	3	7		6	0	1	0		0
1	2	3	2	1				1	1				5	5				0	0			
2	2	3	2	2	1	0	0	1	1	1	1	1	4	7	7	8	5	0	0	0	0	0
3	1	3	0					1					3					1				

2	2	3	0	1	3	4	1	1	1	2	3	1	4	6	5	3	4	0	0	0	1	0
2	2	3	0		0	0	0	1		1	1	1	6		7	8	7	0		0	0	0
1	2	3	1	2	2			1	1	1			5	7	7			0	0	0		
2	2	1	3	1	1	1	0	2	1	1	1	1	2	3	3	4	6	1	1	1	0	0
1	2	2	0	3				1	2				5	4				0	0			
1	2	1	0					1					2					1				
1	1	1	0	1	0	3		1	1	1	2		5	5	6	8		0	0	0	0	
2	2	3	2	1	0			1	1	1			4	7	6			0	0	0		
2	2	3	0	1	1	0	0	1	1	1	1	1	6	6	7	1	4	0	0	0	1	0
2	2	3	0	0	1	0	0	1	1	1	1	1	4	5	7	6	7	0	0	0	0	0
2	3	3	0					1					4					0				
2	2	3	0	1	1			1	1	1			5	3	3	2	5	0	1	1	1	0
2	2		0	1	0			1	1	1				6	5	2			0	0	1	
2	1	3	4	0	3	5		3	1	2	3		4	5	3			0	0	1		
2	3	3	2	4	3	3		1	3	2	2			5					0			
2	3	3	0	1	2			1	1	1			5	6	7			0	0	0		
1	2	3	3	2			1	2	1			1	3	4			5	1	0			0
2	2	3	0					1					4					0				
2	2	1	0		0			1		1			2		6			1		0		
2	3	3	0	3	0	1	0	1	2	1	1	1	4	5	7	8	7	0	0	0	0	0
2	2	3	2	3	3		1	1	2	2		1	4	5	7	6	7	0	0	0	0	0
2	2	3	0	3	1			1	2	1			5	5	6			0	0	0		
2	2	3	3	1	4	1		2	1	3	1		5	4	5	4		0	0	0	0	
2	2	3	1		0			1		1			3		5			1		0		
2	2	3	2	1	0	0	0	1	1	1	1	1	3	4	5	3	6	1	0	0	1	0
2	2	3	0	1	0	3		1	1	1	2		4	5	7	8		0	0	0	0	
2	2	3	2	2	5	2	1	1	1	3	1	1	5	4	3	7	4	0	0	1	0	0
2	2	3	2					1					4					0				
2	1	3	3	1				2	1				3	4				1	0			
2	2	3	2	1	3	3	0	1	1	2	2	1	6	6	7	8		0	0	0	0	
2	2	3	0	3	1	2	0	1	2	1	1	1	5	5	6	6	1	0	0	0	0	1
2	2	3	0	3				1	2				4	3				0	1			
2	2	3	2	1				1	1				4	3				0	1			
2	3	3	0	3	0	3	1	1	2	1	2	1	4	6	7	7	3	0	0	0	0	0
2	2	2	2	2	2	2	0	1	1	1	1	1	4	6	4		6	0	0	0		0
2	2	3	3	4	3		2	2	3	2		1	4	5	7		5	0	0	0		0
2	2	3	0	0	2	4	0	1	1	1	3	1		3	3		6		1	1		0
1	2	3	2					1					3					1				
2	2	3	0	0				1	1				4	4				0	0			
2	2	3	1	1	1	0	1	1	1	1	1	1	5	5	6	8	4	0	0	0	0	0
2	2	3	1		6	3		1		3	2		3		1	3		1		1	1	

2	2	3	1	1	0	0	0	1	1	1	1	1	5	5	7	4	3	0	0	0	0	0
1	2	1	1	0	2	1		1	1	1	1		5	5	7	9		0	0	0	0	
2	2	3	2	1	3			1	1	2			4	6	7			0	0	0		
2	2	3	0	0	0			1	1	1			4	5	5			0	0	0		
2	2	3	0	0	1			1	1	1			6	6	4			0	0	0		
2	2	3	2	2	3	2		1	1	2	1		4	3	3	2		0	1	1	1	
2	1	4	0					1					2					1				
2	3	3	1	0	3	4	3	1	1	2	3	2	6	6	7	8	1	0	0	0	0	1
2	2	3	1	2	0			1	1	1			4	4	7	8		0	0	0	0	
2	2	3	0	1	0			1	1	1			3	5	7			1	0	0		
2	2	3	0	2	3	1	0	1	1	2	1	1	6	5	7	2	4	0	0	0	1	0
2	3	3	3	1				2	1				3	4				1	0			
2	2	3	1	5	3	0	0	1	3	2	1	1		5	5	3	5		0	0	1	0
2	1	1	1	0	2	3		1	1	1	2		2	3	3	7		1	1	1	0	
2	2	3	0	0				1	1				4	4	3			0	0	1		
1	2	3	1	3	2			1	2	1			4	5	4			0	0	0		
2	2	2	1	1	0	7	0	1	1	1	3	1	3	3	6	1	1	1	1	0	1	1
2	2	3	4	3	1	2	0	3	2	1	1	1	3	2	7	6	3	1	1	0	0	0
2	2	3	0	2	0	1		1	1	1	1		4	3	3	1		0	1	1	1	
1	2	1	2	2	5	4	0	1	1	3	3	1	3	3	7	4	3	1	1	0	0	0
2	2	3	0	2	0			1	1	1			4	6	7			0	0	0		
1	2	2	1	3	5			1	2	3			3	4	4			1	0	0		
2	2	1	2	2	5		0	1	1	3		1	3		3	6	3	1		1	0	0
2	2	2	0		1	1		1		1	1		7		7	9		0		0	0	
2	1	1	3	7	4			2	3	3			3	5	1			1	0	1		
2	2	3	1	1	1	0		1	1	1	1		7	7	7	9		0	0	0	0	
2	2	3	1	1	1			1	1	1			6	6	7			0	0	0		
2	2	1	1	3				1	2					2					1			
2	1	3	1	2	1	2		1	1	1	1		3	2	3	3		1	1	1	1	
2	2	3	3	1	0			2	1	1			3	6	7			1	0	0		
2	3	3	5					3					7					0				
2	1	4	0					1														
3	1	3	1		0			1		1			3		3			1		1		
1	2	3	1	1	0	3	2	1	1	1	2	1	3	4	7	3	6	1	0	0	1	0
3	2	2	0					1					6					0				
1	2	2	0					1					3					1				
2	2	3	0	2	1			1	1	1			4	5	7	8		0	0	0	0	
2	2	3	0		1	2		1		1	1		4		7	9		0		0	0	
2	1	3	0	0	0	1	4	1	1	1	1	3	7	5	7	8	7	0	0	0	0	0
3	1	3	2	5	1	5		1	3	1	3		6	5	4			0	0	0		
2	2	2	0					1					5					0				

2	3	3	1					1					6					0				
2	3	3	2	0	0			1	1	1			5	7	7	8		0	0	0	0	
2	2	3	1	1	3			1	1	2			5	5	4			0	0	0		
2	2	2	0		1	3		1		1	2		2		4	8		1		0	0	
2	2	3	0	1	0	0	1	1	1	1	1	1	6	4	7	9	4	0	0	0	0	0
2	1	3	2	2	3	2	0	1	1	2	1	1	3	4	4	6	3	1	0	0	0	0
2	1	3	3					2					3					1				
2	2	3	0	0	0			1	1	1				6	6				0	0		
2	2	3	1	3	3		1	1	2	2		1	7	5	7	2	5	0	0	0	1	0
1	2	3	3		4			2		3					3	5				1	0	
2	2	3	0	0	1	0	0	1	1	1	1	1	5	5	6	8	4	0	0	0	0	0
2	2	3	0	1	1			1	1	1			4	3	7	8		0	1	0	0	
2	2	2	1	2	1	2		1	1	1	1		7	7	7	7		0	0	0	0	
1	2	1	4					3														
2	2	3	1	1	3			1	1	2			4	4	7	4		0	0	0	0	
1	1	1	0	3				1	2				1	2				1	1			
1	1	4	0		2			1		1												
1	2	1	0		3			1		2					4					0		
2	3	3	1	1	0	2	1	1	1	1	1	1	6	6	7	7	6	0	0	0	0	0
1	2	3	3		4	4		2		3	3		3		5	6		1		0	0	
2	3	3	2		0			1		1			4		4			0		0		
2	2	3	2	1	4			1	1	3			3	5	5			1	0	0		
2	2	3	0					1					5					0				
2	2	3	1	0	0			1	1	1			5	4	6			0	0	0		
2	2	3	0	2	1			1	1	1			4	5	4			0	0	0		
2	2	3	1					1					5					0				
2	2	3	0					1					3					1				
2	3	2	4	2	2	5		3	1	1	3		6	5	2	1		0	0	1	1	
2	2	3	0	1	2	2		1	1	1	1		6	7	6	6		0	0	0	0	
2	2	2	0	6	3			1	3	2			5	4	3			0	0	1		
2	2		3	1	1	3	0	2	1	1	2	1	4	5	3	1	1	0	0	1	1	1
2	2	3	0	0	1	0	0	1	1	1	1	1	3	4	4	5	3	1	0	0	0	0
1	2	3	1	2	4	2		1	1	3	1		4	5	6	3		0	0	0	1	
3	2	3	0	0	0			1	1	1			6	7	7			0	0	0		
2	2	3	1	0	1	1	0	1	1	1	1	1	5	6	7	8	4	0	0	0	0	0
1	2	2	0					1					7					0				
2	2	2	3	2				2	1				2	2				1	1			
2	2	3	2					1					3					1				
3	2	3	3	1	1	0		2	1	1	1			2	2	1			1	1	1	
2	1	3	1	2	0	2		1	1	1	1			7	7	8			0	0	0	
1	2	3	1	5	4	1		1	3	3	1		3	4	6	3		1	0	0	1	

2	1	3	2	1				1	1				3	5				1	0			
2	2	3	1					1					5					0				
2	1	3	0	1	2	1	3	1	1	1	1	2	6	3	3	4	4	0	1	1	0	0
1	3	1	0	1	3			1	1	2			3	3	4	3		1	1	0	1	
2	3	3	1	1	1		0	1	1	1		1	3	5	7	9	5	1	0	0	0	0
2	3	3	1	1	0	0	1	1	1	1	1	1	7	7	7	9	6	0	0	0	0	0
2	1	2	1					1														
2	3	3	1	1				1	1					4		3			0		1	
2	2	1	1	0	0	0	1	1	1	1	1	1	5	5	7	8	4	0	0	0	0	0
2	2	2	1					1					5					0				
2	2	3	1					1					3					1				
2	3	3	0	1	7	1		1	1	3	1		4	7	7	6		0	0	0	0	
2	2	3	2	2	2	1		1	1	1	1		4	5	4	4		0	0	0	0	
1	2	2	1					1					4					0				
2	2	2	3	5	0			2	3	1			5	5		4		0	0		0	
1	2	2	2		1	3		1		1	2		7		7	9		0		0	0	
2	3	3	0	1	0	1	0	1	1	1	1	1	7	7	7	8	6	0	0	0	0	0
1	2	3	3					2					3					1				
1	2	4	2	0	4		2	1	1	3		1	2	2	3	3	7	1	1	1	1	0
3		3	2		1			1		1			4		1	1		0		1	1	
2	2	3	1					1														
2	2	3	0	1	0	0	1	1	1	1	1	1	5	5	7	7	6	0	0	0	0	0
2	1	3	3	2	1			2	1	1			5	4	7	6		0	0	0	0	
2	2	3	0					1					5					0				
1	2	3	0	1	3	1	3	1	1	2	1	2	4	7	2	1	7	0	0	1	1	0
1	2	2	1	2				1	1				2	5				1	0			
2	3	3	1	2	1			1	1	1			5	7	7	8		0	0	0	0	
2	2	3	0	1	0	1	2	1	1	1	1	1	4	6	6	8	2	0	0	0	0	1
2	2	3	0					1					5					0				
1	2	3	0	0	1	1		1	1	1	1		4	6	7	9		0	0	0	0	
2	2	3	1					1					3					1				
2	1	3	1	4	0	2	0	1	3	1	1	1	4	7	7	9	6	0	0	0	0	0
1	2	2	1					1														
2	2	3	3					2					4					0				
2	3	3	0	0	0	2	3	1	1	1	1	2	4	5	7	8	5	0	0	0	0	0
2	1	3	0	2	2	1	1	1	1	1	1	1	3	5	6	3	4	1	0	0	1	0
2	3	2	2	2	5	0	0	1	1	3	1	1	3	6	5	7	6	1	0	0	0	0
2	3	3	0	1	0		0	1	1	1		1	6	7	7		5	0	0	0		0
2	1	2	0	0	1			1	1	1			2	5	4	6		1	0	0	0	
2	2	3	3					2					4					0				
2	2	2	1		1	1		1		1	1		3		3	2		1		1	1	

2	2	3	4		1			3		1			4		7	2		0		0	1	
2	3	2	4		1			3		1			2		3			1		1		
1	2	2	1	3				1	2				3	3				1	1			
1	2	2	3	1	2	0		2	1	1	1		4	3	5	4		0	1	0	0	
2	2	3	0	0	1	0	0	1	1	1	1	1	5	5	5	3	6	0	0	0	1	0
2	1	1	1	6		8		1	3		3		3	2		1		1	1		1	
1	1	1	0	2	3	0		1	1	2	1		4	3	6			0	1	0		
2	2	3	1	1	0			1	1	1			5	6	7			0	0	0		
1	2	2	4	4	2			3	3	1			6	4	4	1		0	0	0	1	
2	2	1	1		0	1		1		1	1					8					0	
1	1	3	4					3					3					1				
2	2	3	0	0				1	1				4	4				0	0			
2	2	3	3	1	3	1		2	1	2	1		5	7	7	9		0	0	0	0	
2	2	1	2	1	1		2	1	1	1		1	3	5	3	2	1	1	0	1	1	1
2	2	3	1	0	1	1		1	1	1	1		3	6	5	6		1	0	0	0	
2	2	2	1					1					4					0				
2	2	3	2	0	0	1	0	1	1	1	1	1	3	5	6	6	3	1	0	0	0	0
2	2	1	0					1					4					0				
2	1	3	4	3	4			3	2	3			3	3	3			1	1	1		
2	2	3	4	4	3			3	3	2			4	5	7	5		0	0	0	0	
2	2	3	1		0			1		1			7					0				
2	2	3	0	1	0			1	1	1			4	5	7			0	0	0		
2	2	1	2					1					4					0				
2	3	3	0	2				1	1				4	2				0	1			
2	1	3	1	2	1	1	0	1	1	1	1	1	4	3	5	6	2	0	1	0	0	1
2	3	3	0	0	0	1		1	1	1	1		6		7	9		0		0	0	
2	2	3	2	1	0	1	1	1	1	1	1	1	4	5	7	8	2	0	0	0	0	1
2	2	3	0	1	2		0	1	1	1		1	7	7	7	9	4	0	0	0	0	0
2	2	3	0	0	0	1		1	1	1	1		5	7	7	6		0	0	0	0	
2	2	3	0	3	1		1	1	2	1		1	4	5	7	9	6	0	0	0	0	0
2	3	3	3	2	3	3		2	1	2	2		3	3	2	3		1	1	1	1	
2	2	3	0					1					4					0				
2	1	3	0	2	0	4		1	1	1	3		4	3	3			0	1	1		
2	2	2	0	2	2			1	1	1			6	7	7			0	0	0		
2	2	3	0		1			1		1			5		7			0		0		
2	1	3	0	1	0			1	1	1			4	7	7	8		0	0	0	0	
2	3	3	2	0	1	5		1	1	1	3		5	5	6	8		0	0	0	0	
2	2	3	0	1	0	0	0	1	1	1	1	1	6	5	7	2	5	0	0	0	1	0
2	2	3	0	1	0	2	0	1	1	1	1	1	6	7	7	9	3	0	0	0	0	0
2	1	2	3	1				2	1					2					1			
2	2	3	0	2	0	0		1	1	1	1		4	7	7	9	5	0	0	0	0	0

3	2	3	4	3	3	0		3	2	2	1		4	2	3	5		0	1	1	0	
2	3	3	0	1				1	1				4	6				0	0			
2	2	1	1		2			1		1			3		3			1		1		
2	2	3	2					1					6					0				
1	1	1	2	1	2	3	1	1	1	1	2	1		2	4		7		1	0		0
1	2	1	1	2	1	2		1	1	1	1			4	5	2			0	0	1	
2	2	3	0	2	0	2	1	1	1	1	1	1	6	5	7	7	5	0	0	0	0	0
2	2	3	0	0	0	2	1	1	1	1	1	1	4	4	7	8	2	0	0	0	0	1
1	2	2	1					1					7					0				
2	2	3	2					1					3					1				
2	3	3	2					1														
2	1	3	1	0	3			1	1	2			6	4	7			0	0	0		
3	1	3	2	1	1	0	1	1	1	1	1	1	4	4	4	2	3	0	0	0	1	0
2	1	3	0	1	0	0	2	1	1	1	1	1	3	3	3	3	5	1	1	1	1	0
1	2	1	1		2	1	1	1		1	1	1			4	2	7			0	1	0
2	2	3	2			0	3	1			1	2	3				1	1				1
2	1	3	1	0	3	3		1	1	2	2			5	4	2	1		0	0	1	1
2	2	3	0	1	1			1	1	1			6		3			0		1		
1	2	1	1	1	1			1	1	1			3	3	7	6		1	1	0	0	
2		3	2		3			1		2			4		3			0		1		
2	2		0	1	0		0	1	1	1		1	4	5	7		6	0	0	0		0
2	1	1	1					1														
2	1	3	1					1					3					1				
2	2	3	2	2	2			1	1	1			4	7	3	3		0	0	1	1	
2	2	3	2					1					3					1				
2	2	3	3	2				2	1				3	5				1	0			
2	2	3	5	1	1	2		3	1	1	1		4	4	7	8		0	0	0	0	
2	2	3	0	2	0	0		1	1	1	1		5	3	4	6	4	0	1	0	0	0
2	2	3	0	1	0	1	0	1	1	1	1	1	4	4	6	7	3	0	0	0	0	0
2	2	3	0	1	1	1	1	1	1	1	1	1	4	5	5	7	6	0	0	0	0	0
1	1	1	1	0	4	2		1	1	3	1		1	7	1	6		1	0	1	0	
1	1	1	1					1														
2	2	3	0	3	1	2	2	1	2	1	1	1	7	7	6	7	3	0	0	0	0	0
2	3	3	0	1	1			1	1	1			6	6	7	9		0	0	0	0	
2	2	3	4					3					3					1				
2	2	3	2		0	3		1		1	2		5		6	7		0		0	0	
2	2	3	0	1	1		0	1	1	1		1	5	4	7		4	0	0	0		0
2	1	3	1	3	2			1	2	1			3	6	6			1	0	0		
2	2	3	1					1					4					0				
2	2	4	4	1	1			3	1	1			2	2	6	2		1	1	0	1	
2	2	2	1	1	1			1	1	1			4	3	2			0	1	1		

3	1	3	0	0	1	7		1	1	1	3		6	6	2	1		0	0	1	1	
2	2	3	1					1					4					0				
1	1	1	2					1					5					0				
1	2	1	4	2	3			3	1	2			4	4	5			0	0	0		
2	1	3	0					1					7					0				
2	3	3	0	1	0	3	1	1	1	1	2	1	7	7	7	9	1	0	0	0	0	1
2	2	3	2	4	3	1		1	3	2	1		4	6	3	6		0	0	1	0	
2	1	3	1					1					4					0				
1	2	2	4					3					2					1				
2	2	3	0	2	2	1	0	1	1	1	1	1	5	6	4		3	0	0	0		0
2	2	2	0		2			1		1			5		7		1	0		0		1
2	3	3	2	1	0	0	1	1	1	1	1	1	6	5	7		7	0	0	0		0
3	3	3	4	1	0	2	1	3	1	1	1	1	3	3	2	4	1	1	1	1	0	1
1	1	3	4					3					3					1				
2	3	3	0					1					3					1				
2	1	3	0	1	0	1	1	1	1	1	1	1	4	5	5	5	1	0	0	0	0	1
2	2	3	0	1	0	0	1	1	1	1	1	1	7	5	5	6	3	0	0	0	0	0
2	3	3	0	4	2	2	1	1	3	1	1	1	6	4	7	6	5	0	0	0	0	0
2	2	3	2	1	0	0	0	1	1	1	1	1	1	5	7			1	0	0		
2	2	3	1	2				1	1				4	3				0	1			
2	3	3	2	2	1			1	1	1			5	7	7	7		0	0	0	0	
1	2		1		1			1		1			7		2			0		1		
2	1	3	0					1					3					1				
1	2	2	0					1					4					0				
1	2	2	1	3	0			1	2	1				5	7	7			0	0	0	
1	2	2	0	2	0	0	1	1	1	1	1	1	3	3	5	7	4	1	1	0	0	0
2	2	2	1					1					3					1				
2	2	4	4		3		0	3		2		1	3		7		6	1		0		0
2	3	3	0	2				1	1				6	5				0	0			
2	2	3	0	3	3	1	0	1	2	2	1	1	5	4	4		2	0	0	0		1
1	2	3	2					1					3					1				
2	1	3	0	1		0		1	1		1		4	3				0	1			
2	2	3	0		2	2	1	1		1	1	1	4		7	2	5	0		0	1	0
2	2	3	0	1	2	2	2	1	1	1	1	1	4	3	3	1	1	0	1	1	1	1
2	1	3	0	0	0	1		1	1	1	1		4	5	6	2		0	0	0	1	
2	2	3	2	3	2	2		1	2	1	1		4	2	7	8		0	1	0	0	
2	3	3	0	1		2	2	1	1		1	1	6	7		9	5	0	0		0	0
1	2	1	3	2	4	2	1	2	1	3	1	1	4	6	4	3	1	0	0	0	1	1
2	2	3	3	1	2		2	2	1	1		1	2	6	4		3	1	0	0		0
2	2	3	0	1	0	3	5	1	1	1	2	3	5	7	7	8	3	0	0	0	0	0
2	2	3	3	0	0			2	1	1			3	5	6	2		1	0	0	1	

2	2	2	4					3					3					1				
1	3	1	5	0	2			3	1	1			3	3	3			1	1	1		
1	3	3	0	1	3	1		1	1	2	1		5	4		5		0	0		0	
1	2	3	2		2			1		1			4		3			0		1		
2	1	3	1					1					4					0				
1	2	2	1					1					4					0				
2	2	3	0	1				1	1				3	7				1	0			
2	3	3	1	1	1	1	0	1	1	1	1	1	5	7	7	9	5	0	0	0	0	0
2	2	3	1	1	0	1	2	1	1	1	1	1	5	7	7	9	5	0	0	0	0	0
3	2	3	1	0	0	2	3	1	1	1	1	2	4	5	6	1	3	0	0	0	1	0
2	1	3	0	0	1	1		1	1	1	1		5	7	7	8		0	0	0	0	
1	1	3	4					3					5					0				
2	2	3	1					1					4					0				
1	2	1	1	4	6	7	2	1	3	3	3	1	5	3	6	4	3	0	1	0	0	0
2	2	3	1	3	3	4	0	1	2	2	3	1	3	5	4	2	6	1	0	0	1	0
2	2	3	2	0	1	3	2	1	1	1	2	1	4	6	7	8	3	0	0	0	0	0
2	3	3	2	2	1	3		1	1	1	2		4	5	5	6		0	0	0	0	
2	2	3	1	1	0		1	1	1	1		1	5	6	7	8	3	0	0	0	0	0
2	2	1	1					1					4					0				
2	2	3	3					2					2					1				
2	2	3	1	2	2	1		1	1	1	1		5	4	7			0	0	0		
2	3	3	0	1	0	2	1	1	1	1	1	1	6	7	7	9	4	0	0	0	0	0
1	2	3	1	2	0			1	1	1			3	5	3			1	0	1		
2	2	3	0	2	0	2		1	1	1	1		4	7	6		5	0	0	0		0
2	1	4	5					3					2					1				
1	1	1	0					1					6					0				
2	2	3	2					1														
2	2	1	0	1				1	1				3					1				
2	1	1	2	3	2	2	0	1	2	1	1	1	3	3	5	7	5	1	1	0	0	0
2	3	3	0	3	1	1	0	1	2	1	1	1	4	4	5	8	5	0	0	0	0	0
2	1	3	1			2		1			1		4			7		0			0	
2	2	3	1	1	0	1	1	1	1	1	1	1	7	6	7		5	0	0	0		0
2	3	3	0	0	0	0		1	1	1	1		6	6	7	8		0	0	0	0	
2	2	3	1	3	1			1	2	1			6	7	3	3		0	0	1	1	
2	2	3	0	0				1	1				4	5				0	0			
2	1	3	0					1					4					0				
3	2	3	3	2	1	4	2	2	1	1	3	1	6	5	4	6	7	0	0	0	0	0
2	1	3	3	3				2	2				4	7				0	0			
2	2	3	1	1				1	1				6	3				0	1			
2	2	3	0	2	0			1	1	1				3	7				1	0		
2		2	1	0	1	0	0	1	1	1	1	1	5	5	7	8	4	0	0	0	0	0

1	2	2	1	1				1	1				3	4				1	0			
2	2	3	2	1				1	1				3	3				1	1			
2	2	3	3					2					4					0				
2	3	3	1	0	1	1		1	1	1	1		4	5	7	8		0	0	0	0	
2	2	1	1	2	0	2	1	1	1	1	1	1	5	2	5	7	4	0	1	0	0	0
2	2	3	0					1					4					0				
2	2	3	2	1	0			1	1	1			6	6	7			0	0	0		
3	2	3	3	1	1	1	0	2	1	1	1	1	1	7	2	2	5	1	0	1	1	0
2	3		1	3	4			1	2	3			5	4	7			0	0	0		
2	2	3	0	0		0		1	1		1		4	4				0	0			
2	2	3	0	1	0	0	1	1	1	1	1	1	5	3	2	7	3	0	1	1	0	0
2	2	3	4	3	1	6		3	2	1	3		4	5	1	2		0	0	1	1	
2	3	3	0	0	3			1	1	2			4	5	7			0	0	0		
1	3	3	2	4	1			1	3	1			3	3	3			1	1	1		
2	2	3	1	1	1	1	1	1	1	1	1	1	3		5	6	6	1		0	0	0
2	2	3	0	4				1	3				5	5				0	0			
2	2	3	4		2			3		1			3		5			1		0		
2	3	3	1	0				1	1				5	4		3		0	0		1	
2	2	3	2					1					4					0				
1	2	1	1	0				1	1				1	2				1	1			
1	2	3	1	2	0			1	1	1			5	6	6			0	0	0		
2	2			2	0	3	3		1	1	2	2	5	4	5	5	6	0	0	0	0	0
1	1	2	1	1				1	1				4	6				0	0			
2	2	3	0	1	0	1	0	1	1	1	1	1	4	5	5	4	3	0	0	0	0	0
2	2	3	0	1	2	1		1	1	1	1		4	5	4			0	0	0		
1	3	1	4	1	1	1		3	1	1	1		4	4	6			0	0	0		
2	2	2	3		3	3	1	2		2	2	1	2		3	1	5	1		1	1	0
1	2	1	0		0	3	0	1		1	2	1	2		7	9	3	1		0	0	0
3	1	1	1	0	2			1	1	1			2	2	2			1	1	1		
2	2	3	0	0	0			1	1	1			6	5	7	7		0	0	0	0	
2	2	3	0	1	2	5	0	1	1	1	3	1	5	5	7		3	0	0	0		0
2	3	3	2					1					2					1				
2	1	2	0					1					4					0				
1	2	2	6					3					2					1				
2	2	3	0	2	1	1	1	1	1	1	1	1	4	5	5	8	3	0	0	0	0	0
2	2	3	1	3	1			1	2	1			5	4	5	4		0	0	0	0	
2	2	3	0	1	1	1	0	1	1	1	1	1	4	4	3	4	7	0	0	1	0	0
1	2	3	2					1					2					1				
2	2	3	0	0	1			1	1	1			4	5	6			0	0	0		
2	1	3	2		5	3	0	1		3	2	1	7		7	8	5	0		0	0	0
2	2	3	0	2	2			1	1	1			7	7	7	1	3	0	0	0	1	0

2	3	3	0	0	0			1	1	1			5	6	6	8		0	0	0	0	
2	2	3	1	1	0	3	1	1	1	1	2	1	3	5	6		7	1	0	0		0
2	2	3	0		1	2		1		1	1		4		7	4		0		0	0	
2	2	2	5					3					3					1				
2	3	2	2		1			1		1			2					1				
2	2	2	0	1	0	1	0	1	1	1	1	1	5	7	7	9	6	0	0	0	0	0
2	2	3	0	3		0		1	2		1		4	7		2		0	0		1	
1	2		2					1														
3	1	3	1	1	2	0	5	1	1	1	1	3	4	7	2	1	1	0	0	1	1	1
2	3	3	0	1	0	1	0	1	1	1	1	1	7	7	7	8	5	0	0	0	0	0
2	3	3	0					1					4					0				
2	2	3	1					1					5					0				
2	2	2	0					1					3					1				
2	2	3	0	2	1	3	2	1	1	1	2	1	5	5	7	9	6	0	0	0	0	0
1	2	1	2			1		1			1		1			6		1			0	
2	3	3	0	1	0	1	0	1	1	1	1	1	4	4	7	9	5	0	0	0	0	0
2	3	3	1	1	0	2	0	1	1	1	1	1	4	5	5	1		0	0	0	1	
2	2	3	3	4	3		1	2	3	2		1	4	6	6		1	0	0	0		1
2	2	3	3	1	1		2	2	1	1		1	3	4	3		7	1	0	1		0
2	2	1	1		2	4	4	1		1	3	3	4		3	3	3	0		1	1	0
2	2	3	1	2	1	2	1	1	1	1	1	1	7	7	7	9	6	0	0	0	0	0
2	1	1	1		3	4		1		2	3		2		5		3	1		0		0
2	3	3	1	2	6			1	1	3			4	2	6			0	1	0		
2	2	3	2					1					4					0				
1	2	1	1					1					2					1				
1	1	1	4	2	1	0	3	3	1	1	1	2		4	4	1	5		0	0	1	0
1	2	3	0	1	1			1	1	1			4	3	7			0	1	0		
2	2	3	0	1	3		0	1	1	2		1	4	4	5	7	3	0	0	0	0	0
2	3	3	1	1	1	0	2	1	1	1	1	1	4	7	7	9	4	0	0	0	0	0
3	2	3	1	2	4	2	0	1	1	3	1	1	4	7	4	4	6	0	0	0	0	0
2	2	3	0					1					6					0				
2	1	3	3	4	1	1	0	2	3	1	1	1	3	3	6		6	1	1	0		0
2	3	3	1	5	3			1	3	2			6	4	3	8		0	0	1	0	
2	1	3	0	2	2	3		1	1	1	2		4	4		8		0	0		0	
2	2	2	0					1					4					0				
2	1	3	5	3				3	2				4	5				0	0			
2	2		4	3				3	2				3	2				1	1			
2	3	3	0		1	0		1		1	1		3					1				
1	1	2	2	2		3		1	1		2					2					1	
2	2	3	1					1					5					0				
2	2	3	0	1	0			1	1	1			5		6			0		0		

1	3	3	5	3	3		1	3	2	2		1	3	3	4	3	1	1	1	0	1	1
2	2	3	0	2	1	2	1	1	1	1	1	1	6	2	3	3	1	0	1	1	1	1
2	2	3	3	2	4	3	1	2	1	3	2	1	4	5	7	9	4	0	0	0	0	0
1	2	1	2	1	1		0	1	1	1		1	4	7	7	8	3	0	0	0	0	0
2	2	3	0	0	3	0	2	1	1	2	1	1	5	4	6	6	4	0	0	0	0	0
2	3	3	0	1	0	1	5	1	1	1	1	3	6	7	7	9	3	0	0	0	0	0
2	2	3	2	2	1	0	2	1	1	1	1	1	4	7	7	7	7	0	0	0	0	0
2	1	1	2	0	0	1	3	1	1	1	1	2	3	4	3	1	1	1	0	1	1	1
1	2	2	4					3					4					0				
2	2	3	0					1					4			4		0			0	
2	1	3	0	1				1	1				3	4				1	0			
2	3	3	3					2					5					0				
3	1	3	3	3	4	2	3	2	2	3	1	2	3	5	3	1	4	1	0	1	1	0
2	2	3	0	4	1	4		1	3	1	3		3	2	3	4	4	1	1	1	0	0
3	2	3	0	0	0	4	0	1	1	1	3	1	4	4	4	1	3	0	0	0	1	0
2	2	1	1	3	2			1	2	1			3	3	6	6		1	1	0	0	
3	2	3	2	2	1	1	0	1	1	1	1	1	5	4	4	2	7	0	0	0	1	0
2	2	3	4	2	4			3	1	3			3	3	7	3		1	1	0	1	
1	2	1	2					1					3					1				
2	3	4	2		0	1		1		1	1		3		7	8		1		0	0	
2	3	3	3	2	2	2	3	2	1	1	1	2	5	5	3	4	2	0	0	1	0	1
2	2	3	2					1					2					1				
3	1	3	0	1	0	0	0	1	1	1	1	1	3	3	6	1	5	1	1	0	1	0
2	2	3	1	3		0		1	2		1		4	6				0	0			
1	2	1	3					2														
2	2	1	1					1					6					0				
2	3	3	1	1	1		2	1	1	1		1	6	7	7		7	0	0	0		0
1	2	1	2	3	1	2	2	1	2	1	1	1		2	7	6	4		1	0	0	0
2	1	3	3	1				2	1					2					1			
2	2	3	0	0	0			1	1	1			6	7	7			0	0	0		
2	2	3	2		0	6		1		1	3		7		7	7		0		0	0	
2	2	1	5	1	5			3	1	3			3	7	7			1	0	0		
1	2	3	2	3	1	7		1	2	1	3		4	2	3	9	4	0	1	1	0	0
1	2	3	3		1		8	2		1		3	3		7		2	1		0		1
2	2	3	1	1	1	6		1	1	1	3		4	5	6			0	0	0		
2	2	3	5	1				3	1				5					0				
2	2	3	0	2	1	3	2	1	1	1	2	1	7	7	7	8	7	0	0	0	0	0
2	1	3	1	3	1	2		1	2	1	1		4	4	7	1		0	0	0	1	
2	2	3	0	4	1	3	2	1	3	1	2	1	5	4	5	6	2	0	0	0	0	1
2	2	3	2	0	1	1		1	1	1	1			6	7	8			0	0	0	
2	2	2	2					1					3					1				

2	2	3	0	2	1	1	4	1	1	1	1	3	3	1	3	1	7	1	1	1	1	0
2	2	2	4		6			3		3			3		2			1		1		
2	2	3	2	1	0	1		1	1	1	1		4	2	4	4		0	1	0	0	
1	2	3	0					1					4					0				
1	3	3	1	1	0	1	0	1	1	1	1	1	3	4	5	8	7	1	0	0	0	0
2	1	3	2	1	0	2	2	1	1	1	1	1	5	4	7	7	5	0	0	0	0	0
2	2	3	1	1	1	1	0	1	1	1	1	1	4	5			7	0	0			0
2	2	3	2	1		2		1	1		1			4		8			0		0	
1	2	3	1					1					4					0				
2	3	3	0					1					4					0				
2	2	3	4					3					6					0				
2	2	3	0	4	3	1	4	1	3	2	1	3	3	4	1	1	2	1	0	1	1	1
2	1		1		0			1		1			3		4			1		0		
2	2	3	0	0	2	0	0	1	1	1	1	1	4	5	7	7	6	0	0	0	0	0
2	2	2	3					2					5					0				
2	2	3	0					1					4					0				
2	1	2	0					1					2					1				
2	2	4	3					2					3					1				
3	3	3	0	0	0		2	1	1	1		1	5	6	7		6	0	0	0		0
2	2	3	1					1					5					0				
2	2	3	2	0	0		0	1	1	1		1	5	6	7		3	0	0	0		0
1		1	0					1					1					1				
1	2	1	2					1														
2	2	3	2	0				1	1				5	4				0	0			
2	2	3	1		1			1		1			3		2			1		1		
2	2	2	1	1	2	1	3	1	1	1	1	2	3	4	4	6	4	1	0	0	0	0
2	3	3	2	2	1			1	1	1			3	4	7			1	0	0		
2	2	3	1	2	1	1	0	1	1	1	1	1	7	7	2	2	5	0	0	1	1	0
2	3	3	3					2					3					1				
2	2	2	2					1					3					1				
2	1	3	0					1														
2	3	3	0	1	1			1	1	1			7	7	7			0	0	0		
2	1	3	1	1	0			1	1	1			4	7	7			0	0	0		
2	3	3	1	1	0	3	2	1	1	1	2	1	5	3	4	7	1	0	1	0	0	1
3	2	4	0	2				1	1				3	3				1	1			
2	2	3	1					1					4					0				
1	2	3	0	0				1	1				5	5				0	0			
1		1	3	0				2	1				2	3				1	1			
2	1	3	0	1	0	1		1	1	1	1		3	3	4	1		1	1	0	1	
3	3	3	3	4	4	3		2	3	3	2		3	7	4	2		1	0	0	1	
2	2	2	2		3	3	4	1		2	2	3	4		3	1	2	0		1	1	1

2	2	3	1	3	3	3		1	2	2	2		4	4	6	4		0	0	0	0	
2	2	3	3	2	0		1	2	1	1		1	5	7	7	9	4	0	0	0	0	0
2	2	3	0	1	2			1	1	1			3	4	4	3		1	0	0	1	
2	2	1	4	1	1	3		3	1	1	2		3	2	3	4		1	1	1	0	
2	2	3	1		1			1		1			5		4			0		0		
2	1	1	0					1					3					1				
2	2	3	0					1					5					0				
2	2	3	1	3	2	3		1	2	1	2			7	3	4			0	1	0	
2	3	3	3					2					5					0				
2	2	3	0	3	4	0	0	1	2	3	1	1	4	5	3	2	4	0	0	1	1	0
2	2	3	1	2	3			1	1	2			5	5	7			0	0	0		
2	2	2	2	2				1	1				3	4				1	0			
2	2	3	2	4	3			1	3	2					1	1				1	1	
3	2	2	1	0	1			1	1	1			3	3	3	1		1	1	1	1	
2	2	2	3	0				2	1				2	3				1	1			
2	2	2	0	1	0	4	0	1	1	1	3	1	6	2	4	5	4	0	1	0	0	0
2	2	3	0	2				1	1				5					0				
2	2	3	3	1	1	2	0	2	1	1	1	1	5	3	2	2	7	0	1	1	1	0
1	2	2	0					1					3					1				
1	1	3	2	0	0			1	1	1			3	2	4	3		1	1	0	1	
2	1	3	0	0				1	1				4	5				0	0			
1	2	1	4		4	1	1	3		3	1	1	3		6	1	5	1		0	1	0
3	2	3	2					1					3					1				
2	1	3	0		3	2		1		2	1		4		3	6		0		1	0	
2	2	3	1					1					4					0				
2	2	3	1	1	0	1	2	1	1	1	1	1	6	7	7	9	4	0	0	0	0	0
1	1	2	0	5	0			1	3	1			4	6	7			0	0	0		
2	3	3	0	1	3	3		1	1	2	2		4	3	5	4		0	1	0	0	
2	2	3	0					1					4					0				
2	2	3	0	0	1			1	1	1			5	5	6	7		0	0	0	0	
2	2	3	4	3	2	0	0	3	2	1	1	1	5	4	7	6	3	0	0	0	0	0
2	2	3	1			1	3	1			1	2	4			7		0			0	
2	1	2	1	0	1	3	3	1	1	1	2	2	4	4	5	4	1	0	0	0	0	1
1	2	2	1		4	1		1		3	1		3		7	8		1		0	0	
2	2	3	0	2	2	5		1	1	1	3		4	4	6	1		0	0	0	1	
2	3	3	1	2	2	4		1	1	1	3		3	7	3			1	0	1		
2	3	3	2	3				1	2				4	4				0	0			
2	2	3	1	2	1	1	0	1	1	1	1	1	4	3	6	6	3	0	1	0	0	0
2	2	3	0	2	2	0	0	1	1	1	1	1	4	3	4	6	4	0	1	0	0	0
2	2	3	1					1					4			6		0			0	
2	2	1	1					1					3					1				

2	3	2	3		1	5	0	2		1	3	1	5		3	2	2	0		1	1	1
3	2	3	3					2					2					1				
2	2	3	1					1					5					0				
2	2	3	0					1					4					0				
1	2	1	0	0				1	1				3	2				1	1			
2	1	3	0					1					5					0				
2	1	1	5					3														
2	2	3	1	0	1	4		1	1	1	3		5	7	5	1		0	0	0	1	
2	2	3	0	2	5			1	1	3			5	5	3			0	0	1		
2	1	4	1					1					3					1				
2	2		1	0	3		0	1	1	2		1	6	7	7	8	4	0	0	0	0	0
2	1	3		3	0				2	1			3	6	3			1	0	1		
2	2	3	1	5	1	0	0	1	3	1	1	1	4	2		3	3	0	1		1	0
2	2	3	1	3		0		1	2		1		5	7		8		0	0		0	
3	1	3	0	3	2	0	0	1	2	1	1	1	4	3	3	3	7	0	1	1	1	0
2	2	3	0	0	2	1		1	1	1	1		4	5	3	3		0	0	1	1	
1	1	2	2	2	5	1	0	1	1	3	1	1	4	2	2	4	3	0	1	1	0	0
3	2	3	0	0	0	0	0	1	1	1	1	1	4	5	6	1	2	0	0	0	1	1
2	3	2	5	7	4	3	1	3	3	3	2	1	3	7	7	8	6	1	0	0	0	0
2	3	3	2	1	2	1	1	1	1	1	1	1	4	5	6	4	3	0	0	0	0	0
2	2	3	1	2				1	1				4	1				0	1			
2	2	3	0	1	1	0	2	1	1	1	1	1	5	7	7	9	2	0	0	0	0	1
2	2	3	2	2	0	3		1	1	1	2		4	5	6	2		0	0	0	1	
2	2	3	0	2	0			1	1	1			4	5	6			0	0	0		
2	2	3	0	3	1	1		1	2	1	1		4	4	1	1		0	0	1	1	
2	3	3	3	0	0			2	1	1			3	5	7	9		1	0	0	0	
3	2	3	2	2	4	2	1	1	1	3	1	1	3	3	2	1	6	1	1	1	1	0
2	2	4	1					1														
1	3	1	2		1			1		1			2		7			1		0		
2	2	3	0	1	0	1	1	1	1	1	1	1	5	5	7	8	4	0	0	0	0	0
2	2	3	0	0	0	0	1	1	1	1	1	1	4	5	7	7	6	0	0	0	0	0
2	1	3	0	0				1	1				3	4				1	0			
1	2	2	0	2	0	1		1	1	1	1		3	6	5	7		1	0	0	0	
2	2	3	0	3	0	2	0	1	2	1	1	1	3	7	7		5	1	0	0		0
2	1	3	0	0	1	0		1	1	1	1		4	3	7	3		0	1	0	1	
2	2	3	5					3					3					1				
2	1	3	1					1					3					1				
2	3	3	2		3	0		1		2	1		5		7	8		0		0	0	
2	2	3	2	0	0	2	0	1	1	1	1	1	4	5	4		4	0	0	0		0
2	2	3	0	0	5	1	0	1	1	3	1	1	4		3	1	7	0		1	1	0
2	1	3	0	0	1	2	2	1	1	1	1	1	6	7	4	6	7	0	0	0	0	0

1	2	1	0		1			1		1			5		3			0		1		
1	3	1	0		0	0	0	1		1	1	1	3		7		6	1		0		0
2	1	3	1	1	2	3		1	1	1	2			5	5	1			0	0	1	
2	2	3	0	1	1	1		1	1	1	1		5		6	7		0		0	0	
2	2	3	0	2	1	0	1	1	1	1	1	1	3	5	6	6	6	1	0	0	0	0
1	2	3	1	5				1	3				4	2				0	1			
2	2	2	0					1					3					1				
1	1	2	0					1					3					1				
2	2	3	1					1					4					0				
2	1	3	4					3					4					0				
1	2	3	1		0	2	0	1		1	1	1	4		4	4	3	0		0	0	0
2	3	3		1	2		2		1	1		1			7	8	6			0	0	0
2	2	3	1	0	1			1	1	1			4	6	7			0	0	0		
1	2	2	3					2					4					0				
2	2	3	2	1	1	3		1	1	1	2		6	7	7	8		0	0	0	0	
2	2	3	3	3	3		0	2	2	2		1	4	6	7	9	3	0	0	0	0	0
2	2	3	0	2	0			1	1	1			6	2	3			0	1	1		
2	2	3	0	1	1			1	1	1			5	7	7			0	0	0		
2	2	2	0		0	0	0	1		1	1	1	6		6	2	4	0		0	1	0
3	3	3	2	1	0	2		1	1	1	1		3	5	6	5		1	0	0	0	
2	2	3	0	1	0	3		1	1	1	2		4	5	7	8		0	0	0	0	
1	2	3	1		4		2	1		3		1	3		3		3	1		1		0
2	2	3	2	4	1	4		1	3	1	3		4	7	5			0	0	0		
2	2	3	1	5	5	2	1	1	3	3	1	1	4	4	7		3	0	0	0		0
3	3	3	0					1					5					0				
2	1	3	0	1		2		1	1		1		6	7				0	0			
2	2	3	2	1	1	1	8	1	1	1	1	3	7	5	6	7	3	0	0	0	0	0
1	2	3	2	2			1	1	1			1		7		8			0		0	
2	2	3	3	3	1			2	2	1			3	4	1			1	0	1		
3	2	3	1					1					3					1				
2	3	3	1	1	1	0	0	1	1	1	1	1	4	5	6	9	6	0	0	0	0	0
1	2	1	1		2	2		1		1	1		2		4	8		1		0	0	
2	2	3	0	1	0			1	1	1			4	3	6			0	1	0		
2	3	3	3	2	0	1		2	1	1	1		2	4	7			1	0	0		
1	1	3	5	0	4	3		3	1	3	2		2	4	5			1	0	0		
2	2	3	0	5	3	1	1	1	3	2	1	1	2	2	5	3	3	1	1	0	1	0
3	1		0	1	0			1	1	1			3	4	3	1		1	0	1	1	
2	3	3	1	4	2	1		1	3	1	1			5	7	9			0	0	0	
2	3	3	1					1					2					1				
2	1	3	0	0	1			1	1	1			4	3	1			0	1	1		
2	2	4	5					3					2					1				

2	2	3	0	0	1	2		1	1	1	1		5	5	6			0	0	0		
2	2	3	3	1				2	1					4					0			
2	2	3	1	1	0			1	1	1			5	5	6	6		0	0	0	0	
2	2	3	2					1					3					1				
2	1	3	0	3	1	2	0	1	2	1	1	1	3	6	7	9	3	1	0	0	0	0
2	2	3	0	0	0			1	1	1			6	7	7			0	0	0		
2	3	3	1	0	1	0	1	1	1	1	1	1	4	6	6	9	6	0	0	0	0	0
2	1	1	0					1					2					1				
2	1	3	2	2				1	1				3	6				1	0			
1	3	3	1	2	1	1		1	1	1	1		3	5	6	8		1	0	0	0	
2	2	3	1	1	1		4	1	1	1		3	3	7	7		1	1	0	0		1
2	2	3	2	2	1	4	2	1	1	1	3	1	4	5	6	7	1	0	0	0	0	1
2	2	3	2	4	5	0		1	3	3	1		2	5	1	1		1	0	1	1	
2	3	3	0	1	0	0		1	1	1	1		4	5	6	6	6	0	0	0	0	0
2	2	4	1	1	6			1	1	3			2	3	3			1	1	1		
3	1	3	1					1					3					1				
2	2	3	0	1	0	1	0	1	1	1	1	1	5	6	5	7	5	0	0	0	0	0
2	2	3	0					1					5					0				
2	3	3	0	0	0	3	2	1	1	1	2	1	6	7	7	7	2	0	0	0	0	1
2	2	3	1	0	1	1	1	1	1	1	1	1	5	7		2	1	0	0		1	1
2	2	3	2	1	0			1	1	1			4	4	7			0	0	0		
2	2	3	4	2	2	2	1	3	1	1	1	1	4	7	5	8	3	0	0	0	0	0
2	2	3	2		0			1		1			6		7			0		0		
2	2	3	0	1	3	2	0	1	1	2	1	1	4	5	3	1	2	0	0	1	1	1
2	2	3	0	1	1	1		1	1	1	1		4	4		9		0	0		0	
2	2	3	5					3					4					0				
2	3	3	0	2				1	1				4	3				0	1			
2	3	3	3		2	4		2		1	3		4		7	8		0		0	0	
2	2	3	1					1					4					0				
2	3	3	0	2	1	2		1	1	1	1		4	3	7			0	1	0		
2	2	3	0	0	0		0	1	1	1		1	5	3	5		3	0	1	0		0
2	2	3	1	4	2			1	3	1			4	4	5	7		0	0	0	0	
2	1	3	0		0			1		1			7		7			0		0		
1	1	2	3					2					2					1				
2	2	3	0		0			1		1			5		6			0		0		
2	2	3	3	2				2	1				3	4				1	0			
3	2	3	0	1	4	2		1	1	3	1		5	6	3	7		0	0	1	0	
2	3	3	0	1	1			1	1	1			5	6	4			0	0	0		
2	2	2	0					1					4					0				
2	1	3	3		0			2		1			4		6			0		0		
2	1	3	0		0			1		1			4		2			0		1		

2	3	3	0	1	1			1	1	1					5	4				0	0	
2	2	3	0	1	0			1	1	1			5	5	6	7		0	0	0	0	
2	2	3	0					1					5					0				
2	2	3	2	1	3	0	0	1	1	2	1	1	4	7	7	2	6	0	0	0	1	0
2	2	3	0	1	1	2		1	1	1	1		4	5	7	9		0	0	0	0	
2		1			1					1					6					0		
3	1	3			3	2				2	1		4		6	6	1	0		0	0	1
2	2	3	0	2				1	1				4	6				0	0			
2		3		1	2				1	1			4	4	4			0	0	0		
2	1	3	0	1	0	3		1	1	1	2		5	5	6			0	0	0		
2	2	3	0	0	0	1	2	1	1	1	1	1	4	5	7	8	3	0	0	0	0	0
2	2	3	0	1	0	1	1	1	1	1	1	1	4	6	7	8	5	0	0	0	0	0
2	3	3	1	1	1	2	0	1	1	1	1	1	3	4	4	1	5	1	0	0	1	0
2	2	2	2	0	1	3	1	1	1	1	2	1	4	5	5		4	0	0	0		0
2	1	3	2					1					4					0				
2	2	3	1	2	2	0		1	1	1	1		7	3	7	4		0	1	0	0	
2	2	3	1	4	1	2		1	3	1	1		4	2	6	9		0	1	0	0	
2	2	3	1	3	2	2		1	2	1	1		4	7	7			0	0	0		
1	3	3	0	3	2	3	0	1	2	1	2	1	3	5	3	1	3	1	0	1	1	0
1	3	3	4	0		2		3	1		1		2	4		4		1	0		0	
2	1	2	2		6			1		3			3		3	1		1		1	1	
2	2	3	0	1	1	2	3	1	1	1	1	2	5	7	7		1	0	0	0		1
2	2	3	1	1	1			1	1	1			5	7	6			0	0	0		
1	2	2	0	3	5			1	2	3			5	3	3	5		0	1	1	0	
2	2	3	0	0	1	0	0	1	1	1	1	1	4	6	7	1	3	0	0	0	1	0
2	1	3	0	0	1			1	1	1			4	6	7	7		0	0	0	0	
2	3	4	3	1	0			2	1	1			3	2	1			1	1	1		
3	3	3	1			3		1			2		7			9	6	0			0	0
2	2	1	5	2	2			3	1	1			4	2	3			0	1	1		
2		3	1	0	2			1	1	1				6	7				0	0		
2	1	3	1	1	4			1	1	3			4	2	1	1		0	1	1	1	
2	2	3	1	2	3	1	0	1	1	2	1	1	4	2	7	8	5	0	1	0	0	0
2	2	3	1	3	1			1	2	1			4	6	5	4		0	0	0	0	
2	2	3	2	3	1	1	1	1	2	1	1	1	4	4	6	5	3	0	0	0	0	0
2	3	3	0	3	0	1		1	2	1	1			7	7	9			0	0	0	
1	2	1	1	3	0			1	2	1			4	5	7	9		0	0	0	0	
1	2	2	2					1					4					0				
2	2	3	1		2		4	1		1		3	3		4	8	3	1		0	0	0
2	2	3	0		1	0		1		1	1		4		7	6		0		0	0	
2	2	2	3					2														
2	3	3	0	0	0			1	1	1			6		7			0		0		

2	2	3	1		1			1		1			4		6			0		0		
2	3	3	1	1	0	3	2	1	1	1	2	1	3	5	7	9	4	1	0	0	0	0
2	3	3	2	5			2	1	3			1	7	3			3	0	1			0
1	1		2	0	2	1		1	1	1	1		3	4	2	3		1	0	1	1	
1	2	2	2					1					3					1				
2	3	3	0	0	2			1	1	1			5	5	2	3	7	0	0	1	1	0
2	2	3	1			3		1			2		3			1		1			1	
3	2	3	5	2	1			3	1	1			3					1				
2	2	3	1	2	2	2		1	1	1	1		4	4	3	1		0	0	1	1	
2	3	3	1	2	1	1	0	1	1	1	1	1	4	6	7		3	0	0	0		0
2	1	3	0	2	1	0	0	1	1	1	1	1	3	4	5	4	6	1	0	0	0	0
2	3	3	1	1	0	1	2	1	1	1	1	1	6	7	7	8	7	0	0	0	0	0
2	2	3	1	0	0	2		1	1	1	1		4	4	7	3		0	0	0	1	
1	2	1	2	2	4	3		1	1	3	2		3	1	2	3		1	1	1	1	
2	2	3	0	2	0	2	1	1	1	1	1	1	4	5	6	8	3	0	0	0	0	0
2	3	3	1					1					7					0				
2	2	1	3					2					3					1				
1	1	1	1					1														
1	1	3	0	2		0		1	1		1		4	5		6		0	0		0	
2	3	3	3	0	1			2	1	1			6	6	7			0	0	0		
1	2	3	1	0	0	1	0	1	1	1	1	1	3	4	4	6	5	1	0	0	0	0
3	1	3	2	1	3			1	1	2			3	4	3	1		1	0	1	1	
2	2	3	0	0	0	0	0	1	1	1	1	1	4	5	5	9	4	0	0	0	0	0
2	2	3	0	1	0	1		1	1	1	1		3	5	7	8		1	0	0	0	
2	2	3	0	2	0	2	2	1	1	1	1	1	4	6	6	8	3	0	0	0	0	0
2	3	3	2	1	0	0	0	1	1	1	1	1	4	3	7	8	7	0	1	0	0	0
2	2	3	3	2	1	2	0	2	1	1	1	1	4	4	5		1	0	0	0		1
2	2	3	3	2	2	2	0	2	1	1	1	1	4	3	4	3	6	0	1	0	1	0
2	2	3	3					2					4					0				
2	1	3	6					3					3					1				
3	2	3	0					1					5					0				
1	2	1	6			5		3			3					5					0	
2	2	3	2					1					3					1				
2	2	2	0	2	3			1	1	2			3	7	6			1	0	0		
2	3	3	2	1				1	1				3	4				1	0			
1	3	2	1					1					4					0				
2	2	3	1	3				1	2				4	4				0	0			
2	2	3	1	0	2			1	1	1			3	6	4			1	0	0		
2	2	3	2	4	5			1	3	3				3	5				1	0		
2	3	3	0	0	1	0	0	1	1	1	1	1	4	6		9	5	0	0		0	0
2	3	3	1	3	2	5	1	1	2	1	3	1	7	7	1	1	7	0	0	1	1	0

3	2	3	0	0	1			1	1	1			6	7	7	8		0	0	0	0	
2	2	3	3	1	2	0	2	2	1	1	1	1	4	5	6	1	6	0	0	0	1	0
2	1	2	6	3	3	4		3	2	2	3		4	5	3	1	1	0	0	1	1	1
2	3	4	7					3					3					1				
2	2	3	3	1	0			2	1	1			4	7	7			0	0	0		
2	3	2	1	1	0			1	1	1			4	5	7			0	0	0		
2	2	3	1		7			1		3			4		7			0		0		
2	1	3	0	1		5		1	1		3		4	6		9		0	0		0	
1	3	1	2					1					4					0				
2	2	3	2	3	1	1	1	1	2	1	1	1	6	5	7	8	6	0	0	0	0	0
2	3	3	2					1														
2	1	3	0	2	2	2	0	1	1	1	1	1	5	6	4	3	3	0	0	0	1	0
2	2	3	0	0	2	0	1	1	1	1	1	1	5	7	7	8	5	0	0	0	0	0
2	2	3	3	1	1	1		2	1	1	1		3	6	7	6		1	0	0	0	
2	1	2	3					2					4					0				
2	1	3	1	2				1	1				3					1				
2	3	3	1	2				1	1					3					1			
2	3	3	2	5	2	0		1	3	1	1		4	3	5	9		0	1	0	0	
2	2	3	3					2					3					1				
2	2	3	0	0	1	2		1	1	1	1		4	4	2	8		0	0	1	0	
2	2	3	0	1	0	3		1	1	1	2		4	3	7	7		0	1	0	0	
1	2	3	0					1					4					0				
2	2	4	3	3	1	0		2	2	1	1		2	4	5	5		1	0	0	0	
2	2	4	3	0	1			2	1	1			5	2	7	9		0	1	0	0	
2	2	2	1		0			1		1			3		5			1		0		
2	1	3	0	0				1	1				4	7				0	0			
2	2	3	2	2	1	3		1	1	1	2		4	4	7	7		0	0	0	0	
1	1	1	0					1					3					1				
2	2	3	2	0	0	3		1	1	1	2		5	5	6	5		0	0	0	0	
2	2	3	0	0	0	0		1	1	1	1		5	6	7	8		0	0	0	0	
1	2	3	0	0	0		1	1	1	1		1	3	6	4	4	4	1	0	0	0	0
2	1	2	0					1					3					1				
2	2	1	3					2														
1	2	2	2					1					3					1				
1	1	2	2	2				1	1				3	3		2		1	1		1	
2	2	3	1		0	3	0	1		1	2	1	4		7	9	3	0		0	0	0
2	2	3	0	0	0			1	1	1			4	6	5			0	0	0		
2	2	3	0					1														
2	3	3	1	2	3	1	4	1	1	2	1	3	5	6	7	5	1	0	0	0	0	1
2	2	3	1	1	3	4		1	1	2	3		5	7	4	9		0	0	0	0	
2	3	3	2	3	0	1	0	1	2	1	1	1	7	5	7	3	2	0	0	0	1	1

2	2	3	3	3	3	2		2	2	2	1		5			4		0			0	
1	2	3	2	4	1	1		1	3	1	1		4	4	5	6		0	0	0	0	
2	1	3	0					1					3					1				
2	2	3	1	4	1	2		1	3	1	1		3		7	8		1		0	0	
2	2	3	0	1				1	1					4					0			
2	2	3	4					3					3					1				
2	3	3	0	2				1	1				4	5				0	0			
2	2	3	1	2	3			1	1	2			4	5	6			0	0	0		
2	2	3	1	1	2	6		1	1	1	3		3	4	5	7		1	0	0	0	
2	3	3	0					1					3					1				
1	2	2	2					1					4					0				
2	3	3	1	1	1	2		1	1	1	1		4	4	6			0	0	0		
2	2	3	2	2	2		2	1	1	1		1	3	3	7	6	4	1	1	0	0	0
2	2	2	3	2	2			2	1	1			4	6	5	8		0	0	0	0	
2	2	3	1	4	2	4		1	3	1	3		4	5	7	8		0	0	0	0	
2	2	1	0	0				1	1													
3	2	3	0	0	0	3		1	1	1	2		4	4	6	4		0	0	0	0	
2	2	3	0	0	0	2		1	1	1	1		6	6	6	6		0	0	0	0	
2	2	3	0	1	0	2	1	1	1	1	1	1	4	6	7	8	6	0	0	0	0	0
2	2	3	0	0	2			1	1	1			3	4	2	1		1	0	1	1	
2	3	3	3	1	0		1	2	1	1		1	5	5	7		5	0	0	0		0
2	2	2	2	3	2	6		1	2	1	3			5	2	1			0	1	1	
3	2	3	4					3					3					1				
2	1	3	1					1														
2	3	3	0	3	1		0	1	2	1		1	4	4	4		3	0	0	0		0
2	2	3	2					1					4					0				
2	2	3	1	1	0	0	2	1	1	1	1	1	3	6	6	1	1	1	0	0	1	1
2	2	3	0	0	0	4	2	1	1	1	3	1	7	7	7	7	7	0	0	0	0	0
2	2	3	1	3	2	1	1	1	2	1	1	1	4	6	7	7	7	0	0	0	0	0
2	2	3	0	1	0	2		1	1	1	1			4	5	2			0	0	1	
2	2	1	0	0	0	1	2	1	1	1	1	1	4	5	3	7	3	0	0	1	0	0
2	3	3	2	1	3	1	2	1	1	2	1	1	3	3	5	5	4	1	1	0	0	0
2	2	3	0	1	2	2	0	1	1	1	1	1	4	7	3		4	0	0	1		0
2	3	3	1	2	0	1		1	1	1	1		5	7	7	9		0	0	0	0	
2	2	3	2	1	1	1		1	1	1	1		4	7	7	9		0	0	0	0	
2	3	3	1	2	2	4		1	1	1	3		4	6	1	8		0	0	1	0	
2	2	3	4	2	4	0		3	1	3	1		3	6	4	7		1	0	0	0	
2	2	3	3			4	2	2			3	1	4			3	3	0			1	0
2	3	3	0	1	0	1		1	1	1	1		5	5	5	6		0	0	0	0	
2	2	3	2					1					4					0				
3	1	3	2	4		3		1	3		2		3			1	4	1			1	0

2	2	3	2	2	2	1	5	1	1	1	1	3	5	5	7	3	1	0	0	0	1	1
2	2	3	0	0	0	0	0	1	1	1	1	1	5	6	6	7	3	0	0	0	0	0
2	2	3	1			2		1			1		6			5		0			0	
2	2	3	0	0		0		1	1		1		4			1		0			1	
2	2	2	0	1	2			1	1	1			5	3	4			0	1	0		
2	2	3	2					1					4					0				
1	2	1	4		4			3		3			2		7			1		0		
2	2	3	1	1		3	0	1	1		2	1	5	7			1	0	0			1
2	3	3	1		1	1	2	1		1	1	1	7		7	9	2	0		0	0	1
2	2	1	0					1					3					1				
2	2	3	1	2	0	2	0	1	1	1	1	1	4	4	5	5	7	0	0	0	0	0
2	2	3	1	0	0			1	1	1			4	4	7			0	0	0		
2	2	3	1	0	0	1		1	1	1	1		6	7	7	8		0	0	0	0	
2	2	3	0	0	0	2		1	1	1	1			5	4	6			0	0	0	
2	1	3	4		3	2		3		2	1		2		3			1		1		
2	2	3	0	1				1	1				3	7				1	0			
3	3	3	3	1	2	2	0	2	1	1	1	1		3	4	3	5		1	0	1	0
2	1	2	0					1					5					0				
2	2	3	0	1	0			1	1	1			5	7	7			0	0	0		
2	2	2	1					1					4					0				
1	1	1			1	4	1			1	3	1	3		4	3	4	1		0	1	0
2	1	3	1	1	1			1	1	1			4	7	7			0	0	0		
2	3	3	1	2	0			1	1	1			2	5	7	8		1	0	0	0	
2	2	3	4	2	3	3		3	1	2	2		3	7	7	6		1	0	0	0	
2	3	3	1		2	3		1		1	2		3		5	4		1		0	0	
2	2	3	0	3	2	2		1	2	1	1		4	7	7	1		0	0	0	1	
2	3	3	3	1	2		2	2	1	1		1	5	6	3	2	1	0	0	1	1	1
2	2	3	0	1	1			1	1	1			3	4	5			1	0	0		
2	2	3	1	1	1			1	1	1			4	4		6		0	0		0	
2	2	3	0	0	0	0	2	1	1	1	1	1	5	7	7	8	3	0	0	0	0	0
2	2	3	0	1	2		1	1	1	1		1	3	6	4		6	1	0	0		0
2	2	3	0					1														
2	2	3	2	2	1		5	1	1	1		3		5	5	4	6		0	0	0	0
2	2	3	0	3	1	1	1	1	2	1	1	1	4	3	6	8	3	0	1	0	0	0
2	2	2	2	2	1	2	5	1	1	1	1	3	3	3	5	4	1	1	1	0	0	1
2	2	3	1					1					5					0				
2	2	3	1	0	1	3		1	1	1	2			6	7				0	0		
2	3	3	0					1					4					0				
2	3	2	4	6	4			3	3	3			3	4	5	3		1	0	0	1	
1	2	1	2					1					4					0				
2	2	3	0					1					5					0				

2	3	2	1					1					5					0				
2	2	1	3					2					2					1				
2	2	3	2	1	2	5	1	1	1	1	3	1	6	7	7	6	6	0	0	0	0	0
2	2	3	2	1	3	1	2	1	1	2	1	1	5	4	3	3	4	0	0	1	1	0
1	3	3	2	1	1			1	1	1			3	7	7	8		1	0	0	0	
2	2	3	3	1	4			2	1	3			6	7	5	9		0	0	0	0	
2	2	2	1	0	1	2		1	1	1	1		3	3	6			1	1	0		
2	3	3	2					1					5					0				
2	3	3	0	2				1	1				4	5				0	0			
2	2	3	1	3	0	5	5	1	2	1	3	3	3	3	3	1	3	1	1	1	1	0
2	2		1	2	2			1	1	1			4	5	2			0	0	1		
2	1	2	0	2	1			1	1	1			5		6			0		0		
2	2	3	0	2	0	1	1	1	1	1	1	1	6	7	7		3	0	0	0		0
2	2	3	1		1			1		1			6		4			0		0		
2	1	3	0	2	3	0		1	1	2	1		4	3	4	3		0	1	0	1	
1	1	1	6					3					2					1				
2	2	1	3					2					3					1				
1	2	1	1					1					4					0				
2	2	3	2	1	0			1	1	1			6	6	6			0	0	0		
2	2	3	0					1					4					0				
2	2	1	3	4		5		2	3		3		3	3		2		1	1		1	
2	1	3	0	1	0	1	0	1	1	1	1	1	4	3	7	8	6	0	1	0	0	0
2	1	3	0	1	0			1	1	1			4	7	7			0	0	0		
1	3	3	0	2	3	1		1	1	2	1		4	6	4	2		0	0	0	1	
2	2	3	1	4	2	0	0	1	3	1	1	1	5	4	7	4	7	0	0	0	0	0
2	2	3	0	2	1	3		1	1	1	2		5	7	7	9	7	0	0	0	0	0
1	2	2	0	2		3		1	1		2		6	7		5		0	0		0	
3	2	3	2	1	2		0	1	1	1		1	5	6	7		3	0	0	0		0
2	2	3	0	1	0	1	1	1	1	1	1	1	4	3	5	3	4	0	1	0	1	0
2	2	1	4					3														
2	2	3	1	2	1	1		1	1	1	1				5	1				0	1	
2	2	3	1	3	2	3	0	1	2	1	2	1	5	4	7	5	4	0	0	0	0	0
2	2	3	0	0	0			1	1	1			4	5	4			0	0	0		
2	2	4	4	2	3			3	1	2			3	3	3	2		1	1	1	1	
2	2	3	0	0	2	2	1	1	1	1	1	1	4	4	3	3	4	0	0	1	1	0
2	2	3	0	1	1	2		1	1	1	1		4	4	3	3		0	0	1	1	
2	2	3	2		3	2	2	1		2	1	1	4		4	8	5	0		0	0	0
2	2	3	0	0	0	1	0	1	1	1	1	1	2	4	4	3	6	1	0	0	1	0
2	2	3	0	0	0	2		1	1	1	1		4	4	7			0	0	0		
2	2	3	0					1					3					1				
2	2	3	2	1	2			1	1	1			3	3	3			1	1	1		

1	1	3	0	3	1			1	2	1			4	7	6		3	0	0	0		0
2	2	3	3	1	3		0	2	1	2		1	5	2	7	6	4	0	1	0	0	0
1	2	3	0		0		1	1		1		1	4		5			0		0		
2	2	3	0	2	0		3	1	1	1		2	4	3	3		2	0	1	1		1
2	2	3	0	1	0	1	0	1	1	1	1	1		6	7	2	4		0	0	1	0
1	2	3	2	3	0		1	1	2	1		1	3	5	6	4	7	1	0	0	0	0
2	3		1	2	0	0	0	1	1	1	1	1	6	6	7	8	6	0	0	0	0	0
2	2	3	1	0	1	0	0	1	1	1	1	1	7	7	5	8	6	0	0	0	0	0
2	2	3	2		2			1		1			6		7	7		0		0	0	
2	2	2	0	1	2	0	0	1	1	1	1	1	3	3	4	5	3	1	1	0	0	0
2	2	3	1	3	3		0	1	2	2		1	3	5	7	8	3	1	0	0	0	0
2	3	3	1	1	0			1	1	1			4	6	7			0	0	0		
1	1	1	1	2				1	1				1	3				1	1			
2	3	3	0					1					7					0				
2	2	1	2	1	3	5		1	1	2	3		5	2	4			0	1	0		
2	2	3	0	0	0	1	0	1	1	1	1	1	4	5	5	6	2	0	0	0	0	1
2	2	2	4					3					3					1				
2	2	3	0	1		2		1	1		1		5	4				0	0			
1	3	3	2					1					4					0				
2	2	3	1		1	3	1	1		1	2	1	4		4	4	5	0		0	0	0
2	3	3	0	0				1	1				4	5				0	0			
2	2	3	0					1														
1	1		1	0	2	1		1	1	1	1			3	6	4			1	0	0	
2	2	3	0					1					4					0				
2	1	3	3	1	2			2	1	1			3	3	2			1	1	1		
2	2	3	3	1	2	5		2	1	1	3		5	3	2	1		0	1	1	1	
2	2	3	2	2	3	1		1	1	2	1		5	7	7	1		0	0	0	1	
1	2	1	0	1	0	0		1	1	1	1		3	5	7	8		1	0	0	0	
2	3	3	0	1	0	0	0	1	1	1	1	1	5	4	5	5	2	0	0	0	0	1
2	2	3	1	1	2		1	1	1	1		1	4	5	2		3	0	0	1		0
2	1	1	3	2		3		2	1		2		2	6				1	0			
2	2	3	0	0	0	0		1	1	1	1		4		6	3		0		0	1	
2	3	3	2					1					4					0				
2	1	2	3		1	3		2		1	2				6	8	5			0	0	0
2	1	3	2	2	5	6		1	1	3	3		4	3	7			0	1	0		
2	2	3	0					1					4					0				
2	3	3	0	2	2		0	1	1	1		1	4	5	5	6	3	0	0	0	0	0
2	2	3	2	2	5	2	1	1	1	3	1	1	4	5	3	4	7	0	0	1	0	0
2	2	3	0	0	1	6		1	1	1	3		3	6	5	5		1	0	0	0	
2	2	3	0	2	4	1	0	1	1	3	1	1	5	5	4	3	3	0	0	0	1	0
2	2	3	2	1	2	2	0	1	1	1	1	1	3	5	7	3	7	1	0	0	1	0

2	2	3	0	1	2	1		1	1	1	1		4	4	7	6		0	0	0	0	
2	2	3	1	1				1	1				2	4				1	0			
2	2	3	4	2	1			3	1	1			3	4	4			1	0	0		
2	1	3	0	0	0	1		1	1	1	1		6	5	6	9		0	0	0	0	
2	3	3	0	3	2	2	2	1	2	1	1	1	2	3	4	3	3	1	1	0	1	0
2	2	3	2	1	0	1	0	1	1	1	1	1	6	5	6	8	7	0	0	0	0	0
3	3	3	1	2	0			1	1	1			5	3	3	2		0	1	1	1	
2	1	3	3	0				2	1					2					1			
2	3	3	3	2	3	1	1	2	1	2	1	1	4	3	6	7	3	0	1	0	0	0
2	2	3	4					3					4					0				
2	2	3	1			1		1			1		4			8		0			0	
2	2	3	0	1	1	4	4	1	1	1	3	3	7	6	6	8		0	0	0	0	
2	2	3	0	1	1	1		1	1	1	1		4	6	3	3		0	0	1	1	
3	1	3	1					1					6			3		0			1	
2	2	3	0	0	0	1	1	1	1	1	1	1	6	7	7	8	6	0	0	0	0	0
2	2	3	3	2	1			2	1	1			5	7	7			0	0	0		
2	3	3	0		0	0	1	1		1	1	1	6		4		7	0		0		0
3	3	3		3	1				2	1			4		7			0		0		
2	2	2	1					1					4					0				
2	2	1	1					1					3					1				
2	2	3	0	2	4			1	1	3			4	2	7			0	1	0		
1	2	2	0	2	2	3		1	1	1	2		5	4	7	3		0	0	0	1	
2	2	3	1	1	1			1	1	1			4	6	6	4		0	0	0	0	
2	2	3	1	0	1			1	1	1			3	3	3			1	1	1		
2	3	3	0	1				1	1					3					1			
2	2	3	0	0	2			1	1	1			4	4	3			0	0	1		
1	2	3	6	3	4			3	2	3			2	4	3			1	0	1		
2	3	1	0	2	0			1	1	1			4	4	6			0	0	0		
1	3	3	2					1					3					1				
3	2	3	0	0				1	1				4	5				0	0			
2	2	3	0	0	0	0	1	1	1	1	1	1	4	4	7	1	3	0	0	0	1	0
2	3	3	1	1	2			1	1	1			5	5	5	5		0	0	0	0	
2	2	1	0	2	3	3	1	1	1	2	2	1		4	6	2	3		0	0	1	0
2	2	3	4					3					3					1				
2	1	3	0	0	1	2	1	1	1	1	1	1	2	3	5	2	3	1	1	0	1	0
2	3	3	4	0	0			3	1	1			4	4	7			0	0	0		
2	3	3	0	0	1	4	3	1	1	1	3	2	6	7	5	6	1	0	0	0	0	1
2	2	3	0	1	3			1	1	2			5	4	3	4		0	0	1	0	
2	3	3	2	1	0	1	2	1	1	1	1	1	4	7	7	9	5	0	0	0	0	0
1	1	3	0					1														
2	3	3	2	2	2	1	0	1	1	1	1	1	3	4	7	8	7	1	0	0	0	0

1	1	1	4					3														
2	2	3	1					1					4					0				
2	1	3	2					1					4					0				
2	2	1	1					1					3			2		1			1	
2	2	3	2	1	0	2	1	1	1	1	1	1	5	6	7	7	3	0	0	0	0	0
2	2	3	1	6	3			1	3	2			3	3	4			1	1	0		
2	1	3	1	2	1	2		1	1	1	1		5	7	7	8		0	0	0	0	
1	1	1	3					2					3					1				
2	2	3	4	5	2			3	3	1			4	3	6	6		0	1	0	0	
2	2	2	1					1					4					0				
2	3	2	0	2	1		1	1	1	1		1	6	3	7		3	0	1	0		0
2	3	3	2	2	5		3	1	1	3		2	5	6	4	4	5	0	0	0	0	0
2	1	3	1		0			1		1			4					0				
2	2	3	3	1	2	1	2	2	1	1	1	1	3	4	5	5	6	1	0	0	0	0
1	1	1	1	5	2	6		1	3	1	3		3	4	2	3		1	0	1	1	
2	2	3	2	4	1		1	1	3	1		1	3	7	7		3	1	0	0		0
1	2	1	1					1					6					0				
2	2	2	4	1	1	1	1	3	1	1	1	1	5	6	7	4	5	0	0	0	0	0
2	1	3	0	0	1	1		1	1	1	1		7	7	6	5		0	0	0	0	
1	1	3	2	1				1	1				4	7				0	0			
2	2	3	1	0	0	0	0	1	1	1	1	1	4	5	7	4	7	0	0	0	0	0
2	2	3	0	3	0	1		1	2	1	1		4	5	7	9		0	0	0	0	
2	2	3	1	1	2	2	1	1	1	1	1	1	5	5	7	3	1	0	0	0	1	1
2	2	3	0	0	0	1	2	1	1	1	1	1	4	4	5	8	2	0	0	0	0	1
2	2	3	1	2				1	1				3	2		3		1	1		1	
2	2	3	1					1														
2	2	3	1					1					7					0				
1	1	3	5					3					3					1				
2	1	3	2	1				1	1				4	5		2		0	0		1	
1	1	1	1	2	1	0	1	1	1	1	1	1	2	6	6	5	5	1	0	0	0	0
2	1	3	0	1				1	1				4					0				
2	2	3	1	1	1	1	1	1	1	1	1	1	5	7	7	6	6	0	0	0	0	0
2	2	3	3	1				2	1				4	6				0	0			
2	1	3	0					1					3					1				
3	3	3	1	0	1		1	1	1	1		1	3	6	2		3	1	0	1		0
1	2	2	4					3					3					1				
1	2	1	4	4	4			3	3	3				4	3				0	1		
1	1	1	1					1					6					0				
1	1	2	1					1					5					0				
2	2	3	0	3	3			1	2	2			5	6	4			0	0	0		
2	2	3	1	1				1	1				4	5				0	0			

2	2	3	1	1	4	1	0	1	1	3	1	1	3	2	5	7	7	1	1	0	0	0
2	1	3	0	0	0	2	0	1	1	1	1	1	4	5	7	7	4	0	0	0	0	0
2	2	1	1					1					6					0				
1	2	3	5			2		3			1		3			6		1			0	
2	2	3	2					1					3					1				
2	2	2	0	1	2			1	1	1			5	7	7			0	0	0		
2	2	3	3	0				2	1				3	2				1	1			
2	2	2	3		2			2		1			3		7			1		0		
2	2	3	0	2	2	2	0	1	1	1	1	1	6	3	4	5	4	0	1	0	0	0
3	1	3	2	4	5	2	0	1	3	3	1	1		3	3		2		1	1		1
2	2	1	2	2	4	1		1	1	3	1			3	1	1			1	1	1	
2	1	3	4					3					4					0				
2	1		4	6	3	0		3	3	2	1		3		1	1		1		1	1	
2	2	3	2	0	0			1	1	1				5	4				0	0		
2	2	3	1	1	1	0		1	1	1	1		5	4	7	8		0	0	0	0	
2	3	3	1		2	0	1	1		1	1	1	5		7	9	7	0		0	0	0
2	2	3	0	2	3	1	2	1	1	2	1	1	6	7	7	7	7	0	0	0	0	0
2	1	4	2					1					4					0				
2	2	3	3	2				2	1				3	4				1	0			
2	2	2	2		1		0	1		1		1	4		4	7	3	0		0	0	0
2	1	3	1	2	0	2	0	1	1	1	1	1	4	6	7	8	6	0	0	0	0	0
2	3	3	3	2	3	1		2	1	2	1		4	3	6	4		0	1	0	0	
1	2	2	3					2					3					1				
2	2	3	3	2	1		0	2	1	1		1	3	5	6		3	1	0	0		0
2	2	3	0	1		3		1	1		2		6	7		9		0	0		0	
2	1	3	0					1					5					0				
1	2	3	2	2	2	0		1	1	1	1		3	5	5	4		1	0	0	0	
2	2	3	2	1				1	1													
2	2	3	3	2				2	1													
2	1	4	2					1					2					1				
2	2	3	1	3	0			1	2	1			5	7	7			0	0	0		
2	3	3	0					1					4					0				
2	3	3	1	3	2	2	2	1	2	1	1	1	7		7	7	3	0		0	0	0
3	3	3	0	0	0	2	0	1	1	1	1	1	4	4	5	9	6	0	0	0	0	0
2	2	3	1	1	1			1	1	1			3	6	6	8		1	0	0	0	
2	3	3	0	0	0			1	1	1			5	5	7			0	0	0		
2	1	3	1	0	1			1	1	1			4	5	6			0	0	0		
1	1	1	5					3					1					1				
2	2	3	0	0	0	6		1	1	1	3		6	7	7	9		0	0	0	0	
2	2	3	1	0	2	0	0	1	1	1	1	1	4	4	1	3	3	0	0	1	1	0
2	1	3	1		0			1		1			5		4		4	0		0		0

2	2	3	0	0	0	0		1	1	1	1		7	7	7	8		0	0	0	0	
2	2	2	0					1														
1	2	3	1					1					4			9		0			0	
2	3	2	1	0	3	3		1	1	2	2		3	3	3	4		1	1	1	0	
2		3																				
2	1	3	3	0	1			2	1	1			4	7	3			0	0	1		
2	2	4	2					1					4					0				
2	3	3	0	1	1	1	1	1	1	1	1	1	5	6	5	8		0	0	0	0	
2	2	3	0	1	0	0		1	1	1	1		5	6	7	4		0	0	0	0	
2	2	3	0	1	2		1	1	1	1		1	3	4	5		6	1	0	0		0
1	2	3	3					2					3					1				
2	2	3	0	0	2			1	1	1			4	3	5	7		0	1	0	0	
1	2	1	1					1					4					0				
2	3	3	0	0	0		1	1	1	1		1	5	6	7	9	7	0	0	0	0	0
2	2	3	4	4	6	6		3	3	3	3				6					0		
1	1	3	0					1					4					0				
2	1	3		1	0	2			1	1	1		6	7	7	7		0	0	0	0	
1	2	1	2	3				1	2				2	4				1	0			
2	1	3	1	1				1	1				5	4				0	0			
2	3	3	0		0	2	0	1		1	1	1	4		7		5	0		0		0
1	1	1	2	3	0	0	0	1	2	1	1	1	5	4	7	7	4	0	0	0	0	0
2	2	3	0					1					4					0				
2	1	2	1					1					4					0				
2	2	1	1	0	2		1	1	1	1		1		2	6		6		1	0		0
2	2	3	3					2					3					1				
2	3	3	1		1	1	2	1		1	1	1	5		7	5	3	0		0	0	0
1	3	2	2		1		1	1		1		1			3		6			1		0
2	2	3	0	2	1	1	1	1	1	1	1	1	4	4	6	6	4	0	0	0	0	0
2	2	3	0	0	0	3	1	1	1	1	2	1	5	5	7		1	0	0	0		1
1	2	2	1					1					3					1				
2	3	3	2	3	2	1	1	1	2	1	1	1	5	6	7	8	4	0	0	0	0	0
2	2	2	1	0	3	0	1	1	1	2	1	1		5	5	8	7		0	0	0	0
2	2	3	0	0	0	3		1	1	1	2		7	5	6	7		0	0	0	0	
2	2	3	2	0	0	1	2	1	1	1	1	1	3	5	7	8	4	1	0	0	0	0
2	1	3	1	2	1			1	1	1			4	3	3			0	1	1		
2	2	3	1	0	0			1	1	1			4	4	6			0	0	0		
2	1	3	0					1					3					1				
2	2	3	1	2	1	2	0	1	1	1	1	1	4	4	5	5	3	0	0	0	0	0
1	2	1	0	2	0			1	1	1			2	7	7			1	0	0		
1	1		1					1					4					0				
2	2	3	1	1	1	1	2	1	1	1	1	1	3	7	5	9	4	1	0	0	0	0

2	2	3		0	1	2	0		1	1	1	1		4	7		4		0	0		0
2	3	3	0	1	1	1	2	1	1	1	1	1	6	4	6	2	5	0	0	0	1	0
2	1	3	0	1	0	2		1	1	1	1		2	1	1	1		1	1	1	1	
2	1	3	3		2			2		1			3		2			1		1		
2	2	3	2	3	3	1	0	1	2	2	1	1	6	7	7	8	7	0	0	0	0	0
2	2	3	1					1					5					0				
2	2	1	3					2					4					0				
1	2	2	3					2					3					1				
2	2		0		4		0	1		3		1	7		5		7	0		0		0
2	3	3	1	1	1		1	1	1	1		1	4		7		3	0		0		0
2	2	3	1					1					3					1				
2	2	3	0	0	0	0		1	1	1	1		5	7	7	3		0	0	0	1	
2	3	3	0	1	0	4		1	1	1	3		6	5	4	7		0	0	0	0	
1	3	3	4	1	2	3	4	3	1	1	2	3	3	5	5		2	1	0	0		1
2	2	3	0	2	1	3		1	1	1	2		7	7	3	8		0	0	1	0	
2	2	3	1	0				1	1				4	5				0	0			
2	3	2	3		4	5		2		3	3		6		2	7		0		1	0	
2	2	3	2	1	0	0	1	1	1	1	1	1	1	4	5	5	3	1	0	0	0	0
2	3	3	0	4	8	4	0	1	3	3	3	1	7	7	3	1	3	0	0	1	1	0
2	2	3	1	2	0	4	2	1	1	1	3	1	4	6	4	5	5	0	0	0	0	0
2	1	3	0	1	0	2	0	1	1	1	1	1	6	5	7	4	6	0	0	0	0	0
2	2	3		2	1				1	1			6	7	7			0	0	0		
2	2	3	0	1	1	3	2	1	1	1	2	1		5	7	6	2		0	0	0	1
2	2	2	0	2		2		1	1		1		4	4		2		0	0		1	
2	2	3	0	0	1	2	0	1	1	1	1	1	6	6	5	4	1	0	0	0	0	1
1		2	4	5	2	2	2	3	3	1	1	1	4	3	4	1		0	1	0	1	
1	1	3	0					1					3					1				
2	2	1	2		0			1		1			4		7	9		0		0	0	
2	2	3	0	1	0	1		1	1	1	1		6	7	7	9		0	0	0	0	
2	1	3	0					1					3					1				
2	2	3	0					1					4					0				
2	2	3	1	1	1	1		1	1	1	1		7	7	7	8	5	0	0	0	0	0
1	2	3	0	1	0			1	1	1			4	4	4			0	0	0		
3	2	3	1	1	2			1	1	1			5	3	5			0	1	0		
2	2	1	1	2	1	1		1	1	1	1		3	2	3	2		1	1	1	1	
2	2	3	0					1														
2	2	3	0	1	0	1	1	1	1	1	1	1	5	7	7	8	7	0	0	0	0	0
2	1	3	0	1	0	1		1	1	1	1			6	2	5			0	1	0	
3	2		0	1				1	1				5	3				0	1			
2	3	3	0	1	2	1	2	1	1	1	1	1	3	5	7	7	3	1	0	0	0	0
2	2	3	1	2	1			1	1	1			4	4	6	1		0	0	0	1	

2	1	3	5					3					3					1				
2	2	3	0					1					5					0				
2	2	3	1	0	0	0	2	1	1	1	1	1	4	5	7	8	5	0	0	0	0	0
2	2	3	3	3	2		1	2	2	1		1	5	6	7		3	0	0	0		0
2	2	1	1	2		5		1	1		3			3		9			1		0	
3	1	3	2	0	2			1	1	1			3	5				1	0			
3	2	3	4				1	3				1	4			1	1	0			1	1
2	2	3	1	3	0			1	2	1			4	3	7		3	0	1	0		0
1	2	3	1	2	0			1	1	1				3	4				1	0		
2	1	3	0	0				1	1					4					0			
2	3	3	0	2				1	1				4	5				0	0			
2		3	0	0	1	3		1	1	1	2		5	6	7	9		0	0	0	0	
2	1	3	0	5				1	3				4	2				0	1			
2	1	3	1	1	0		0	1	1	1		1	4	3	3	4	7	0	1	1	0	0
2	2	3	1	1	1			1	1	1			4	5	5			0	0	0		
2	2	3	1	0	3	0		1	1	2	1		3	3	4			1	1	0		
1	1	1	2	3	1			1	2	1				6	3				0	1		
2		3	1					1														
1	2	3	1	3	3			1	2	2			4	7	1			0	0	1		
2	2	2	0					1					4					0				
2	2	3	1	0	0	3		1	1	1	2		4	5	6	6	7	0	0	0	0	0
2	2	3	2		0			1		1			4		5			0		0		
2	2	3	2		5			1		3			6		4			0		0		
2	2	3	0	1	0			1	1	1			3	3	5			1	1	0		
2	1	3	1	3				1	2					3					1			
2	3	3	1	3	0			1	2	1			6	6	7			0	0	0		
2	2	4	2	2	1	3		1	1	1	2		4	5	7	8		0	0	0	0	
2	2	3	0	1				1	1				5	7				0	0			
1	1	1	4					3														
2	2	2	0					1					3					1				
2	3	3	0	1	2	0	1	1	1	1	1	1	6	4	5	7	2	0	0	0	0	1
2	2	3	2	1	0	0		1	1	1	1			7	7	9			0	0	0	
2	1	2	2	3	1	2	0	1	2	1	1	1	4	3	6	4	1	0	1	0	0	1
2	2	3	1	3				1	2				4	3				0	1			
2	2	3	1	1	0	1		1	1	1	1		5	6	7	9		0	0	0	0	
1	1	2	0	1	1			1	1	1			3	5	2			1	0	1		
1	2	3	3	1	0	2	3	2	1	1	1	2	4	6	4	4	4	0	0	0	0	0
2	1	3	2	2	1	2	0	1	1	1	1	1	4	5	5	4	2	0	0	0	0	1
1	2	4	0	2	3			1	1	2			3	2	2			1	1	1		
2	2		1	0	0	3		1	1	1	2						6					0
2	2	3	2					1					5					0				

3	1	3	0	2	2	2		1	1	1	1		5	5	4	3		0	0	0	1	
2	2	3	1					1					4					0				
2	2	2	1	1	0			1	1	1			7	6	6			0	0	0		
2	2	2	0		2			1		1			5		7			0		0		
2	3	3	0	2	0	1	0	1	1	1	1	1	4	4	6	7	4	0	0	0	0	0
2	2	1	1	3	3	4	1	1	2	2	3	1	3	3	6	6	1	1	1	0	0	1
2	2	3	0	0	1	3		1	1	1	2		5	6	7	8		0	0	0	0	
3	2	3	0	1	1	0	3	1	1	1	1	2	3	7	7			1	0	0		
2	2	3	1	1	5	4	2	1	1	3	3	1	5		2	9	3	0		1	0	0
2	2	3	1	1	3		3	1	1	2		2	5	6	5	6	4	0	0	0	0	0
2	2	3	0	1	2	2		1	1	1	1		3	7	3			1	0	1		
1	2		2	3		2		1	2		1		3	4		6		1	0		0	
2	2	1	1					1														
2	3	3	1	1	2	1	0	1	1	1	1	1	7	7	7	9	7	0	0	0	0	0
2	2	3	0	1	0	3	0	1	1	1	2	1	5	3	7	8	6	0	1	0	0	0
2	2	3	0	0	0	3	2	1	1	1	2	1	3	5	7	8	7	1	0	0	0	0
2	2	3	3	3	3	2		2	2	2	1		7	7	7	9		0	0	0	0	
2	3	3	2					1					5					0				
2	2	3	0	1	0			1	1	1			6		7			0		0		
1	1	1	0					1					4					0				
2	2	3	2	2				1	1				5	5				0	0			
2	2	3	0	0	1	3	1	1	1	1	2	1	4	3	5	3	3	0	1	0	1	0
2	2	3	2	1	0			1	1	1			4	5	5			0	0	0		
2	2	3	2	1	0	1		1	1	1	1		4	2	7	9		0	1	0	0	
2	2	3	1	0				1	1				5	4				0	0			
2	1	3	1	2				1	1				5	5				0	0			
2	2	3	1	1	1	1	1	1	1	1	1	1	7	5	6	6	5	0	0	0	0	0
2	3	2	2					1					4					0				
1	2	3	0	3	0	4		1	2	1	3		3	2	2	2		1	1	1	1	
2	2	3	1	1	0	2		1	1	1	1			6	6				0	0		
2	2	4	3					2					4					0				
2	2	3	0	0	0	1	1	1	1	1	1	1		6	5	6	6		0	0	0	0
2	2	3		3	1				2	1			4	3	3			0	1	1		
2	1	1	4	1	3			3	1	2			3	5	4			1	0	0		
2	2	3	1					1					5					0				
2	3	3	4	4	1			3	3	1			5	6	6			0	0	0		
1	2	2	2					1					5					0				
2	1	2	1	1	0			1	1	1			4	4				0	0			
2	2	3	2	1				1	1				5	6				0	0			
2	2	3	3	1	0	1		2	1	1	1		5	5	6	8		0	0	0	0	
1	1	3	0					1					4					0				

2	2	3	0	1	0	0		1	1	1	1		5	7	7	9		0	0	0	0	
2	2	4	5	6				3	3				3	2				1	1			
2	2	3	0	0	0	0	2	1	1	1	1	1	2	7	7		5	1	0	0		0
2	2	3	1					1					5					0				
2	2	3	4	5	1	0	0	3	3	1	1	1	4	7	6	4	4	0	0	0	0	0
2	2	1	4	1	1		2	3	1	1		1	4	7	7		7	0	0	0		0
2	1	3	2	2	1			1	1	1			3	4	6	6		1	0	0	0	
1	2	1	1					1					5			1		0			1	
1	1	3	1					1					3					1				
2	2	3	0	0	0	1		1	1	1	1		4	4	6	8		0	0	0	0	
2	2	3	1	0	1	2		1	1	1	1		3	4	6	4		1	0	0	0	
2	2	3	0	2	2			1	1	1			5		6			0		0		
1	1	1	4	1				3	1				2	3				1	1			
2	2	3	1		2	4		1		1	3		4		6	5		0		0	0	
3	2	3	0	1	0	1	0	1	1	1	1	1	4	4	5	6	7	0	0	0	0	0
2	2	3	3	4	5			2	3	3			3	2	2	6		1	1	1	0	
2	2	3	0		1			1		1			4		4			0		0		
2	3	2	2	2				1	1				4	5				0	0			
1	2	2	1		0	3		1		1	2		4		7	7		0		0	0	
2		3	5		5	2	0	3		3	1	1	3		4	5	7	1		0	0	0
2	2	3	0	3	2			1	2	1			4	3	4	7		0	1	0	0	
2	3	3	4					3					5					0				
2	3	3	1					1					4					0				
2	3	2	6	7	3	3		3	3	2	2		7	6		9		0	0		0	
2	2	3	0	2	6	1		1	1	3	1		5	7	6	8		0	0	0	0	
2	2	3	1	0	0	0	2	1	1	1	1	1	5	4	5	7	1	0	0	0	0	1
2	2	3	0	1	0			1	1	1			4	5	7			0	0	0		
2	3	2	3	4	6	2		2	3	3	1		3	6	2	4		1	0	1	0	
2	3	3	0	0	0	0	0	1	1	1	1	1		7	7	8			0	0	0	
2	3	3	0	1	3			1	1	2			6	7	5			0	0	0		
2	1	3	2	4	2	1		1	3	1	1		5	5	6	4		0	0	0	0	
3	2	3	1		1	1		1		1	1		4		6	4		0		0	0	
2	1		4					3														
2	2	3	1	2	3			1	1	2			5	5	3			0	0	1		
1	1	2	3					2					4					0				
2	1	3	3	4	1	2		2	3	1	1		4	5	7	8		0	0	0	0	
2	2	3	2	1	0	1		1	1	1	1		4	4	4	8		0	0	0	0	
2	2	3	0	1	0			1	1	1			4	6	7	8		0	0	0	0	
2	3	3	0	5	5	2		1	3	3	1		7	7	7	1		0	0	0	1	
1	1	3	0	1	1			1	1	1			5	7	7			0	0	0		
3	1	3	3	3	1	3	1	2	2	1	2	1	5		7	4	6	0		0	0	0

2	2	3	0	1	3	2		1	1	2	1		4	4	6	1		0	0	0	1	
2	2	3	0	1				1	1													
2	2	3	1	0	2			1	1	1			6	6				0	0			
2	2	3	0	0	0			1	1	1			4	5	6			0	0	0		
2	2	1	3		2			2		1			2					1				
2	2	3	1	1	0			1	1	1			5	7	7			0	0	0		
2	2	3	0					1					4					0				
2	2	3	0					1					5					0				
2	2	3	0					1														
2	2	1	0	1	4			1	1	3			5	3	7	4		0	1	0	0	
2	2	3	2	2	1	3		1	1	1	2		4	6	7	7		0	0	0	0	
2	3	3	2	0	3	0		1	1	2	1		4	4	5	6		0	0	0	0	
2	2	3	2					1					6					0				
1	2	2	0		0			1		1			3		3			1		1		
2	1	3	0	1	0			1	1	1			4	4	7			0	0	0		
2	2	1	3	2	1			2	1	1			2	3	7	7		1	1	0	0	
2	1	3	0	0	3	1		1	1	2	1		4	3	7	8		0	1	0	0	
2	2	3	0	2	2	2	1	1	1	1	1	1	6	7	3		5	0	0	1		0
2	2	3	1	2	4			1	1	3			4	4	4	4		0	0	0	0	
2	2	3	1	0	1	1	0	1	1	1	1	1		4	4	1	7		0	0	1	0
2	2	2	0					1					4					0				
3	1	2	0	3				1	2					3					1			
2	2	3	1	4	3	2		1	3	2	1		4	4	4	8	1	0	0	0	0	1
2	1	2	0					1					3					1				
2	2	3	0	2	1	2	0	1	1	1	1	1	4	6	7	8	1	0	0	0	0	1
2	2	3	0	3	2	6		1	2	1	3		4	4	7	9		0	0	0	0	
2	2	3	3	0				2	1				4	6				0	0			
2	3	3	2	2	0	2		1	1	1	1		4	5	5	7		0	0	0	0	
2	2	3	0	1	2	2		1	1	1	1		6	7	7	9		0	0	0	0	
2	3	1	3	3	3			2	2	2			2	2	3			1	1	1		
2	2	3	1	3	3	0	0	1	2	2	1	1	4	4	2	2	3	0	0	1	1	0
2	2	3	1	1	0	1	0	1	1	1	1	1	7	7	7	9	6	0	0	0	0	0
1	2	1	1	1				1	1				6	5				0	0			
1	2	2	0	2	2			1	1	1			3	2	2			1	1	1		
1	2	1	3	3	4			2	2	3			3	3	5	4		1	1	0	0	
2	2	3	0	1	0			1	1	1			5	7	7			0	0	0		
2	1	3	0	2	0			1	1	1			5	5	7	8		0	0	0	0	
1	1	1	4		1	1		3		1	1		2					1				
2	3	2	2	4	5			1	3	3			2	3	3			1	1	1		
2	2	3	3	2	1			2	1	1			7	7	7			0	0	0		
1	3	3	2					1					4					0				

2	2	4	6	2	2	5	1	3	1	1	3	1	2	3	2	4	3	1	1	1	0	0
1	1	3	4					3					2					1				
2	3	3	0	0	1			1	1	1			4	5	7			0	0	0		
2	2	1	1	1	0			1	1	1			3	2				1	1			
2	3	3	2	0		1		1	1		1		3	6		8		1	0		0	
2	2	3	0	0				1	1				4	6				0	0			
2	2	3	0		1			1		1			4		7	2		0		0	1	
2	2	4	2					1					2					1				
2	2	2	0		2	2	0	1		1	1	1	4		3	3	5	0		1	1	0
2	2	3	2	4	3	4		1	3	2	3		5	6	5			0	0	0		
2	1	3	2	2	1		0	1	1	1		1	4	3	2		6	0	1	1		0
1	1	2	0					1					5					0				
2	2	3	0					1					3					1				
2	3	3	1					1														
1	3	1	1	1	0	2		1	1	1	1		3	5	7			1	0	0		
2	2	3	1	2	2			1	1	1			3	3	6			1	1	0		
2	2	3	0					1					4					0				
2	2	3	1	1	4			1	1	3			4	7	2			0	0	1		
1	1	2											4					0				
2	2	3	0	2				1	1				3	3				1	1			
2	2	3	1					1					3					1				
2	2	3	2	3				1	2				6	4				0	0			
2	2	3	0	1	0			1	1	1			7	7	7			0	0	0		
2	1	3	1	1				1	1					3					1			
2	3	3	1					1					5					0				
2	1	3	0	1				1	1				3	4				1	0			
1	2	3	3	0				2	1				3	3				1	1			
2	3	2	1					1					3					1				
2	2	3	0	1	1		2	1	1	1		1	7	4	6		3	0	0	0		0
2	2	3	0		0	3		1		1	2				6					0		
2	2	2	2					1					7					0				
2	2	3	0					1					4					0				
2	3	3	1			1	0	1			1	1	6			8	2	0			0	1
2	2	3	3					2					6					0				
2	2	3	0	0	0			1	1	1			4	2	7			0	1	0		
2	2	3	2	1	1	1	2	1	1	1	1	1	5	6	5	6	3	0	0	0	0	0
2	2	2	4	2	3	0		3	1	2	1		4	6	3	1		0	0	1	1	
2	3	3	3	3	2	3		2	2	1	2		4	6	7	7		0	0	0	0	
2	2	3	0					1					6					0				
2	2	3	4	5		0		3	3		1		5	5		8		0	0		0	
2	3	3	3	3	1	2		2	2	1	1		4	5	3	2		0	0	1	1	

2	1	2	1					1					3					1				
2	3	3	2	2	1	0	1	1	1	1	1	1	3	7	4	8	3	1	0	0	0	0
2	2	3	0	3	0	3	8	1	2	1	2	3	6	4	5	4	7	0	0	0	0	0
2	1	3	0	1				1	1				3	3		3		1	1		1	
2	2	3	1	1				1	1					7					0			
1	2	3	1	5				1	3				3	4				1	0			
2	2	3	0	0				1	1				4	5				0	0			
2	2	3	0	1	2			1	1	1			3	4	7			1	0	0		
2		3	3	0	1	1	1	2	1	1	1	1		6	5	7	3		0	0	0	0
2	2	2	0	1	3	1		1	1	2	1		6	4		4		0	0		0	
2	2	3	0					1														
2	2	2	1	1	1	1		1	1	1	1			4	6	7			0	0	0	
2	2	3	1	1	0			1	1	1			4	3	7			0	1	0		
2	2	3	1					1					5					0				
2	2	3	2					1					5					0				
2	2	3	1	0	2	1		1	1	1	1		3	5	4	3		1	0	0	1	
1	2	1	0		0			1		1					7					0		
2	2	3	0					1					3					1				
2	2	3	1	1	5	0		1	1	3	1		4	4	4	9		0	0	0	0	
2	3	3	1	1	1	5		1	1	1	3		5	7	7	4		0	0	0	0	
2	1	3	0	1	3	4	0	1	1	2	3	1	4	3	7	8	1	0	1	0	0	1
2	2	3	0	0	2		2	1	1	1		1	4	6	2	2	6	0	0	1	1	0
2	2	3	0	2				1	1				4	5		6		0	0		0	
1	3	3	2		3			1		2			4		4			0		0		
2	2	3	3		0			2		1			4		5			0		0		
2	2	4	1	2	3			1	1	2			4	4	3			0	0	1		
2	3	3	0	0	0	2		1	1	1	1		4	3	3	2		0	1	1	1	
2	2	3	0	0	1	3		1	1	1	2		6	5	6	6		0	0	0	0	
1	2	3	2	2		5		1	1		3		4	3		2		0	1		1	
2	1	3	0	1	1	1	0	1	1	1	1	1	5	6	6	7	5	0	0	0	0	0
2	2	3	0	1	2	2		1	1	1	1		5	7	7	2		0	0	0	1	
2	2	3	0	1	0		2	1	1	1		1	4	7	5		5	0	0	0		0
3	2	3	2	2	0	0		1	1	1	1		3	3	2	1		1	1	1	1	
2	1	1	0					1														
2	3	3	1	1	0	1		1	1	1	1		4		7	8		0		0	0	
2	2	3	1					1					4					0				
2	2	3	1					1														
2	2	3	1	1	2		0	1	1	1		1	4	5	3		7	0	0	1		0
2	2	3	0	1	0	0	2	1	1	1	1	1	4	5	7	1	4	0	0	0	1	0
2	2	1	7					3					3					1				
2	3	3	2	3	5	2		1	2	3	1		4	7	7	8		0	0	0	0	

2	3	3	0	0	2	0	0	1	1	1	1	1	5	6	4	3	7	0	0	0	1	0
2	2	3	3	4	2		1	2	3	1		1	3	4	3		6	1	0	1		0
2	3	3	0	1	2	1	1	1	1	1	1	1	5	6	7		7	0	0	0		0
2	2	3	2	1	2	0		1	1	1	1		5	6	7	8	7	0	0	0	0	0
3	2	3	0		0	1		1		1	1		3		7	4		1		0	0	
2	2	3	0	2	1			1	1	1			6	7	7	9		0	0	0	0	
2	1	4	0					1					2					1				
2	2	3	3					2					4					0				
2	3	3	0	1	3	2		1	1	2	1		4	6	5	1		0	0	0	1	
2	3	3	0	1	0	0		1	1	1	1		6	5	7			0	0	0		
2	2	3	0	2	2	0	1	1	1	1	1	1	4	2	5	4	5	0	1	0	0	0
2	3	3	2	5	4	2	0	1	3	3	1	1	5	7	7	7	6	0	0	0	0	0
2	1	2	1					1					3					1				
1	2	3	1		3	2	0	1		2	1	1	4		4	3	1	0		0	1	1
2	2	3	0	1	0	2		1	1	1	1		7		6	4		0		0	0	
1	2	1	1	2	1			1	1	1			5	6	3			0	0	1		
2	1	3	1	0	0	0		1	1	1	1		5	4	7	8		0	0	0	0	
1	2	1	0					1					3					1				
2	2	3	0	2	0		4	1	1	1		3	5	6	7		5	0	0	0		0
2		3			0					1					7					0		
2	3	1	2	2	1			1	1	1			2	7	7			1	0	0		
2	3	3	2					1					3					1				
3	3	3	1	2	2	0	0	1	1	1	1	1	7	7	6	8	7	0	0	0	0	0
2	1	4	3	2	4			2	1	3			7		4			0		0		
2	3	3	0	0	0	1		1	1	1	1		5	5				0	0			
2	3	3	2	1	0			1	1	1			6	6	7		4	0	0	0		0
2	3	2	0					1					4					0				
1	2	1	3	3	3			2	2	2				5	3				0	1		
2	1	3	2	2	1	1	2	1	1	1	1	1	4	4	7	2	1	0	0	0	1	1
2	3	3	0		1			1		1					1					1		
2	2	3	0	3	1			1	2	1			5	4	7			0	0	0		
2	2	3	0	0		1		1	1		1		4	5				0	0			
2	2	3	1	0	2	1	3	1	1	1	1	2	5	6	4	6	3	0	0	0	0	0
2	1	3	3	3	1	0		2	2	1	1		4	4	7	7		0	0	0	0	
2	1	3	2	3	1			1	2	1			4	6	5			0	0	0		
2	1	3	0	0	0			1	1	1			4	5	6			0	0	0		
3	2	3	1	1	2		2	1	1	1		1	3	4	4		4	1	0	0		0
2	2	3	1	1	0			1	1	1			3	2	6			1	1	0		
2	3	3	0	3	1	2	2	1	2	1	1	1	7	7	7	9	7	0	0	0	0	0
2	1	3	2	0	2	2	1	1	1	1	1	1	3	3	4	1	1	1	1	0	1	1
2	3	3	1					1					6					0				

2	3	3	3	3	2	0	2	2	2	1	1	1	3	5	7	9	2	1	0	0	0	1
2	2	1	3	0	0	2		2	1	1	1		3	2	5	5	5	1	1	0	0	0
2	2	2	2	1	1			1	1	1			4	2	3			0	1	1		
2	2	3	0	1	0			1	1	1			4	4	7			0	0	0		
1	2	2	0	2				1	1				4	7				0	0			
2	2	3	2	3	3			1	2	2			4	4	7	8		0	0	0	0	
1	2	2	2	3	5	1	0	1	2	3	1	1	3	3	2	7	4	1	1	1	0	0
2	2	3	0	0	0	1	0	1	1	1	1	1	7	7	7	9	6	0	0	0	0	0
2	2	3	3					2					4					0				
1	2	3	2	1	2	2	2	1	1	1	1	1	3	6	5	7	3	1	0	0	0	0
2	3	3	2	2	0			1	1	1			5	6	7	8		0	0	0	0	
2	2	2	2	4	4	2		1	3	3	1		4	5	3	8		0	0	1	0	
2	1	3	2					1					3					1				
2	3	3	1	6	3			1	3	2			6	7	7	9		0	0	0	0	
2	2	3	3	1	3	0	0	2	1	2	1	1	4	5	3	8	6	0	0	1	0	0
2	3	3	0	2	1	1	1	1	1	1	1	1	5	5		8	4	0	0		0	0
1	2	3	0	2	4	4		1	1	3	3			4		3			0		1	
2	2	3	1	2	0	3	0	1	1	1	2	1	5	6	6	7	7	0	0	0	0	0
2	2	4	4			2		3			1											
1	2	3	3	2	6	1	0	2	1	3	1	1	3	6	5		3	1	0	0		0
2	2	3	0	3	0	1	0	1	2	1	1	1	5	5	4	3	6	0	0	0	1	0
2	3	4	3					2					5					0				
2	2	3	1	2	2	3		1	1	1	2		5	5	4	8		0	0	0	0	
2	2	3	3	4	3	0		2	3	2	1		3	2	5	3		1	1	0	1	
1	2	3	3		2			2		1			3		7			1		0		
2	2	3	2	2				1	1					4					0			
2	2	3	0	2	1			1	1	1			5	6	4			0	0	0		
2	1	3	0	0	0	2		1	1	1	1		4	5	4	7		0	0	0	0	
2	2	3	3					2					4					0				
2	1	3	2	3	3	1	0	1	2	2	1	1		5	1	2	5		0	1	1	0
2	2	3	0	2	0	4	0	1	1	1	3	1	4	6	4	5	3	0	0	0	0	0
2	3	3	4	5	6			3	3	3			4	5	3			0	0	1		
2	3	3	0	1	1	0	0	1	1	1	1	1	4	6	7	8	6	0	0	0	0	0
1	2	3	2	3	4	4	1	1	2	3	3	1	4	5	3	4	1	0	0	1	0	1
2	2	3	2	2				1	1				5	3				0	1			
2	1	3	0	0	1	1	0	1	1	1	1	1	6	6	7	7	5	0	0	0	0	0
2		2	1			3		1			2		4			3		0			1	
2	2	3	1					1														
2	2	3	1		2	3		1		1	2		5		4	4		0		0	0	
2	2	3	1	1	0		0	1	1	1		1	5	5	7		5	0	0	0		0
2	2	3	1					1					4					0				

2	2	3	2	1				1	1				5	5				0	0			
2	1	2	0	1	0	3	3	1	1	1	2	2	7	6	7	8	7	0	0	0	0	0
2	2	3	0	1	1	2	1	1	1	1	1	1	4	7	7	9	4	0	0	0	0	0
2	3	3	0	3				1	2					5					0			
2	2	3	2	1	1	0	0	1	1	1	1	1	5	5	5	7	4	0	0	0	0	0
2	3	3	2	1	1	0	1	1	1	1	1	1	6	5	4	9	3	0	0	0	0	0
1	2	3	0	0	2		1	1	1	1		1	4	5	7	8	6	0	0	0	0	0
1	2	1	2	2	3			1	1	2			3	4	7			1	0	0		
2	2	3	0		1			1		1			5		7	1		0		0	1	
2	3	3	0		2	0	1	1		1	1	1	7		7	8	5	0		0	0	0
2	2	3	0	0	0	1	2	1	1	1	1	1	4	5	5	5	5	0	0	0	0	0
2	2	3	1	1	0			1	1	1				7	7	6			0	0	0	
2	2	3	1					1														
2	2	3	2	0	0	1		1	1	1	1		4	5	6	6		0	0	0	0	
2	3	3	0	3	0			1	2	1			5	7	7	9		0	0	0	0	
1	3	1	4	2	6	3		3	1	3	2		2	4	5			1	0	0		
2	2	3	0	0	0			1	1	1			4	4	6	7	5	0	0	0	0	0
1	2	2	2	1				1	1					5		1	1		0		1	1
2	3	2	2					1					6					0				
2	2	3	2					1														
2	3	3	0	1	1	1		1	1	1	1		7	5	7	9		0	0	0	0	
2	2	3	1					1					3					1				
1	2	3	3					2					3					1				
2	2	3	0	1	2	5		1	1	1	3		5	3	7	9	5	0	1	0	0	0
2	3	2	1					1					3					1				
2	2	3	0	1	0			1	1	1			4	4	4	7		0	0	0	0	
2	2	1	0	0	1	2	1	1	1	1	1	1	3	3	2	1	6	1	1	1	1	0
2	1	3	1	1	3	3		1	1	2	2			5					0			
2	2	3	0		1	4	0	1		1	3	1	3		7		4	1		0		0
2	1	3	0	4	2	2	3	1	3	1	1	2	4	2		1	2	0	1		1	1
2	2	3	4	3	3	2		3	2	2	1			4	5	2			0	0	1	
2	3	3	3	2	3	1	1	2	1	2	1	1	4	7	3	3	7	0	0	1	1	0
1	1	2	0					1					1					1				
2	2	3	0	1	0			1	1	1			4		6			0		0		
2	2	3	1	1	0	2	1	1	1	1	1	1	4	7	5	3	4	0	0	0	1	0
2	2	3	2	1	0	1		1	1	1	1		5	5	7	7		0	0	0	0	
1	2	2	2					1					2					1				
2	2	3	1	3	0	5	5	1	2	1	3	3		2	5		1		1	0		1
2	1	3	0	1	1			1	1	1				5	7				0	0		
3	2	3	0	0	0	1	1	1	1	1	1	1	5	6	7	6	4	0	0	0	0	0
2	2	3	1	2	0	2		1	1	1	1		4	3	6	8		0	1	0	0	

2	2	3	2			0	3	1			1	2	5			1	5	0			1	0
1	2	3	4	1	2	1	0	3	1	1	1	1	3	4	4	8	4	1	0	0	0	0
3	1	3	1	0	0			1	1	1			4	4	6			0	0	0		
2	3	3		1	1	0	0		1	1	1	1	2	4	4	1	5	1	0	0	1	0
2	2	3	0	4	4	2	0	1	3	3	1	1	5	4	2	1		0	0	1	1	
1	2	4	2					1					2					1				
2	2	3	0	1	1	1	0	1	1	1	1	1	4	6	6		3	0	0	0		0
2	1	3	0	1	0			1	1	1			3	3	4	4		1	1	0	0	
2	1	2	2					1					2					1				
2	2	3	3	4				2	3				4	5				0	0			
2	1	3	4	2				3	1				5	2				0	1			
2	2	3	0	2	0			1	1	1				3	7				1	0		
2	2	3	0		1	0	0	1		1	1	1	4		7	5	5	0		0	0	0
2	1	3	4	0				3	1				3	4				1	0			
2	1	1	5					3					3					1				
1	3	1	5	3	2	0	2	3	2	1	1	1	2	1	1		5	1	1	1		0
2	1	3	1		4			1		3			5		3	3		0		1	1	
2	2	3	2	2	1	5		1	1	1	3		4	3	2	3		0	1	1	1	
1	3	1	1					1					3					1				
2	2	3	0	1	0			1	1	1			6	6	7			0	0	0		
2	3	3	1	1	0	1	1	1	1	1	1	1	5	5	7	8	3	0	0	0	0	0
2	2	3	1	1				1	1					5					0			
2	2	3	0	0	0	0	1	1	1	1	1	1	7	7	1	9	3	0	0	1	0	0
2	3	3	2	1	0	1		1	1	1	1		3	3	7	9		1	1	0	0	
1	1	2	1					1					2					1				
2	2	3	4	0				3	1				3	5				1	0			
1	3	2	5		1	0		3		1	1		3		5	7		1		0	0	
2	2	3	0	0				1	1				6	6				0	0			
2	2	3	1	2	5	4		1	1	3	3		5		5	1		0		0	1	
2	3	3	0	0	2	1	1	1	1	1	1	1	4	4	5	3	3	0	0	0	1	0
2	2	3	0	2	1	0	1	1	1	1	1	1	4		7	8	7	0		0	0	0
2	2	1	0	2	2	1		1	1	1	1		6	7	7	7		0	0	0	0	
2	2	3	2	3				1	2				3	5				1	0			
2	2	3	0	2	1	0	2	1	1	1	1	1	4	3	2	1	7	0	1	1	1	0
2	3	3	2	1	0			1	1	1			5	4	7	9		0	0	0	0	
2	3	3	0	1	1	1		1	1	1	1		7	5	7	7		0	0	0	0	
2	2	3	0	3	1	1	0	1	2	1	1	1	3	4	4	3	3	1	0	0	1	0
2	2	3	0		1	0	2	1		1	1	1	7		7	8	5	0		0	0	0
1	2	3	1	2	2	3	1	1	1	1	2	1	5	3	4	5	4	0	1	0	0	0
2	2	3	1					1					4					0				
2	2	2	2		3			1		2			3		7			1		0		

2	1	4	2	3	3	0	1	1	2	2	1	1	3	4	7	7	4	1	0	0	0	0
3	1	3	1	0	0	2	0	1	1	1	1	1	5	5	7	8	7	0	0	0	0	0
1	2	2	1	1	0	0		1	1	1	1		4	3	6	3		0	1	0	1	
2	3	3	4					3					2					1				
2	2	3	2	5				1	3				3	3				1	1			
2	2	3	1			0		1			1		4			4		0			0	
2	2	3	1	1	2	1		1	1	1	1		6	7	7	3		0	0	0	1	
2	2	3	0	0	4			1	1	3			5	5	3			0	0	1		
2	2	3	0	0	0	0		1	1	1	1		6	7	7			0	0	0		
2	3	3	1	0	1			1	1	1			6	6	7	8		0	0	0	0	
2	2	3	0	1	4			1	1	3			4	4	7	8		0	0	0	0	
2	2	3	0	2	0			1	1	1			5	3	7	6		0	1	0	0	
2	2	1	2	0		2	0	1	1		1	1	7	7		7		0	0		0	
2	2	3	1	1	1		0	1	1	1		1	5	5	3		3	0	0	1		0
2	2	3	0	1	0		0	1	1	1		1	6	7	7		5	0	0	0		0
2	2	3	0		0			1		1			4		4			0		0		
2	3	3	1		1			1		1			6		6			0		0		
1	1	2	2	1	0	0	0	1	1	1	1	1	3	7	6		2	1	0	0		1
2	2	3	3	2	2	0	4	2	1	1	1	3	4	6	3	4	3	0	0	1	0	0
2	1	3	0					1					6					0				
2	3	3	1					1					4					0				
2	3	3	1		4	2		1		3	1		4		4	7		0		0	0	
2	1	2	2	4	3	1		1	3	2	1		4		3	1		0		1	1	
2	2	3	3	4				2	3				4	6				0	0			
2	2	3	2					1					5					0				
2	2	2	1	1	1	1		1	1	1	1		7	6	7	5		0	0	0	0	
2	3	2	2	5	4	2		1	3	3	1		7	7	4	8		0	0	0	0	
1	2	1	3	2				2	1				3	5				1	0			
2	3	3	2	2	3	0		1	1	2	1		5		3			0		1		
1	2	1	1	2	0			1	1	1			1	5	7			1	0	0		
2	3	3	1	0	0	0	0	1	1	1	1	1	4	5	6	6	6	0	0	0	0	0
2	2	3	5					3					5					0				
2	2	2	1					1					5					0				
3	2	2	5	2	4	3	1	3	1	3	2	1	4	7	4	8	6	0	0	0	0	0
2	2	3	2	3	1			1	2	1			3	5	7			1	0	0		
2	2	3	1	3	3	3		1	2	2	2		4	2	7			0	1	0		
2	2	3	2	3	2	2	0	1	2	1	1	1	3	6	7	9		1	0	0	0	
2	2	3	0	2	1	1	2	1	1	1	1	1	4	4	4	6	6	0	0	0	0	0
2		3	0	3	1	1		1	2	1	1			6	7	2			0	0	1	
1	2	3	0					1					5					0				
2	1	3	3	4	6	2		2	3	3	1		4	4	3	1		0	0	1	1	

1	3	2	2	1				1	1					4					0			
2	2	3	2					1					5					0				
2	2	3	1	3	1	2	2	1	2	1	1	1					5					0
2	2	3	0	0	1	1	0	1	1	1	1	1	4	4	7	7	4	0	0	0	0	0
1	2	2	0					1					3					1				
2	1	3	1	2	2			1	1	1				4	3				0	1		
2	2	2	1	3	2		1	1	2	1		1	4	6	3	8	3	0	0	1	0	0
1	2	2	1					1														
2	2	3	2	2	3	3	3	1	1	2	2	2	4	2		1	4	0	1		1	0
2	2	3	0	1	0	0		1	1	1	1		4	4	6	7		0	0	0	0	
2	2	4	2		4			1		3			3					1				
2	3	4	2	3	1		1	1	2	1		1	3	3	7		3	1	1	0		0
2	3	3	0	0	0	0	0	1	1	1	1	1	5	5	7		5	0	0	0		0
2	2	3	3	5	1	5		2	3	1	3		5	7	2	4		0	0	1	0	
2	1	3	0	0	0	2	0	1	1	1	1	1	4	7	7	5	6	0	0	0	0	0
2	2	4	0	1	2			1	1	1				5	3				0	1		
2	3	3	0	0	0	0	0	1	1	1	1	1	5	6	7	8	6	0	0	0	0	0
2	2	3	0	0	0			1	1	1			7	7	7	8		0	0	0	0	
1	2	2	0	1	2			1	1	1			5	5	7	8		0	0	0	0	
2	2	3	0	2	1	4	2	1	1	1	3	1		6	5	2	2		0	0	1	1
2	2	3	0	2	0			1	1	1			5	7	5	4		0	0	0	0	
2	3	3	1	2	0	1	1	1	1	1	1	1	4	3	2	4	5	0	1	1	0	0
3	2	3	1	1	2	3		1	1	1	2		4	5	5	1		0	0	0	1	
2	2	3	1					1					4					0				
1	2	2	0					1					6					0				
2	2	3	1	1	1			1	1	1			7	6	7			0	0	0		
2		4	1					1														
2	2	3	1		2			1		1			4		6			0		0		
2	2	3	0		4			1		3			4		1	1		0		1	1	
2	2	3	2					1					4					0				
1	3	1	1	1				1	1				4	4				0	0			
2	2	3	1					1														
1	2	1	0	3				1	2				4	6				0	0			
2	2	3	0	0	0			1	1	1			5	5	7			0	0	0		
2	3	3	0	1	2	3		1	1	1	2			6	4	2			0	0	1	
2	2	3	1	2	1	7	2	1	1	1	3	1	6	5	7	8	1	0	0	0	0	1
2	3	3	0	0	1	1	0	1	1	1	1	1	6	4	3	7	2	0	0	1	0	1
2	3	3	2	0	1			1	1	1			6	6	7			0	0	0		
2	2	3	0	0	1	2		1	1	1	1		4	6	7	6		0	0	0	0	
2	2	3	3	3	1			2	2	1			5	5	5	2		0	0	0	1	
1	2	3	0	2	2	1		1	1	1	1		3	7	7	7		1	0	0	0	

1	2	3	4		1	2	0	3		1	1	1	3		5	8	3	1		0	0	0
2	2	3	0	1	0		1	1	1	1		1	5	5	7	6	6	0	0	0	0	0
2	2	3	1	3	1			1	2	1			4	4	7	7		0	0	0	0	
2	2	3	0	1	0	1	5	1	1	1	1	3	4	7	7	8	1	0	0	0	0	1
2	2	3	0	5	1	4		1	3	1	3		6	5	6	4		0	0	0	0	
2	2	3	0	1	1	2		1	1	1	1		5	3	5	1		0	1	0	1	
2	2	3	0					1					4					0				
2	1	3	0	0	0	4	4	1	1	1	3	3	4	5	6	2	1	0	0	0	1	1
2	2	3	0					1					4					0				
2	2	2	1		1	2		1		1	1		3		4	4		1		0	0	
2	2	3	2	1	0		2	1	1	1		1	5	6	5	7	7	0	0	0	0	0
1	2	3	0					1					5					0				
2	2	3	1	1	4	2		1	1	3	1		4	4	3	3		0	0	1	1	
2	2	3	0	0	1			1	1	1			5	4	5			0	0	0		
2	2	2	3					2					4					0				
3	1	3	0	0	1		3	1	1	1		2	4				5	0				0
2	2	3	0	0	0		0	1	1	1		1	5	7	7	9	4	0	0	0	0	0
2	2	3	3	0	0			2	1	1			4	4	6			0	0	0		
2	2	3	0	2	1	2	4	1	1	1	1	3	3	4	7	9	2	1	0	0	0	1
2	3	3	1	3	2	4		1	2	1	3		4	4	6	7		0	0	0	0	
2	1	3	2			2		1			1		5					0				
2	3	3	0	0	0		3	1	1	1		2	5	6	7		2	0	0	0		1
2	2	3	0		1			1		1			3		2			1		1		
2	3	3	4	1				3	1				3	3				1	1			
1	2	2	2	4	1	2	1	1	3	1	1	1	3	7	7	6	5	1	0	0	0	0
2	3	3	0	2	2			1	1	1			6	5	7			0	0	0		
2	2	2	4		6			3		3			5		3			0		1		
2	2	3	4	1	2			3	1	1			5	7	7			0	0	0		
2	2	3	0		2			1		1			4		6			0		0		
2	2	3	0	1	0	1		1	1	1	1		5	5	7			0	0	0		
2	1	3	2	1				1	1				5	7				0	0			
2	2	2	1		0			1		1			4		7	8		0		0	0	
3	3	3	0	0	2	4		1	1	1	3		5	7	4	2		0	0	0	1	
2	2	3	2	3	3			1	2	2			5	2	7			0	1	0		
2	2	3	0	1	2	0	1	1	1	1	1	1	5	4	5	6	5	0	0	0	0	0
2	3	2	5	4	1	3	4	3	3	1	2	3	7	4	7		3	0	0	0		0
2	3	1	4	1	5			3	1	3			4	2	3	3		0	1	1	1	
2	1	3	4	1	2			3	1	1			3	4				1	0			
2	2	3	0	0	1			1	1	1			4	5	3			0	0	1		
2	1	4	5	0				3	1				3	3				1	1			
2	3	3	0	1	1		0	1	1	1		1	4	7	7		4	0	0	0		0

1	2	2	0	0		0		1	1		1		3	4		3		1	0		1	
2	2		3					2					3					1				
1	2	3	0	1	1	1	8	1	1	1	1	3	4	4	5	7	2	0	0	0	0	1
2	1	2	5	0	0	1	0	3	1	1	1	1	2	6	7	8	4	1	0	0	0	0
2	3	3	1			1		1			1		7					0				
2	2	3	1	3	1		0	1	2	1		1	4	4	7	6	3	0	0	0	0	0
2	2	3	1	0	1	2	1	1	1	1	1	1	4	6	3	7	5	0	0	1	0	0
2	2	3	4					3					4					0				
2	3	3	3	2	1	1		2	1	1	1		6	7	7	8		0	0	0	0	
2	2	3	0		5	4		1		3	3		4		5	1		0		0	1	
2	2	2	0					1					3					1				
2	3		1	0	0	0	1	1	1	1	1	1	4	7	7	5	7	0	0	0	0	0
2	2	3	1					1					3					1				
2	2	3	1			3		1			2					3					1	
2	1	3	1		1	0		1		1	1		3		3			1		1		
2	2	3	0					1					3					1				
2	2	3	6		5		1	3		3		1	3		4		4	1		0		0
2	3	2	2			4		1			3		7					0				
2	3	3	0	0				1	1					3					1			
2	1	3	2	3	2	2	2	1	2	1	1	1	3	4	3	8	4	1	0	1	0	0
2	2	3	1	0	2	0	0	1	1	1	1	1	5	5	7	8	3	0	0	0	0	0
1	2	3	3	2	2			2	1	1			3	4	7	7		1	0	0	0	
2	1	3	1	1	0	3	0	1	1	1	2	1	4	4	7	4	3	0	0	0	0	0
2	2	3	2					1					3					1				
2	3	3	2	1	0			1	1	1			5	4	7			0	0	0		
2	2	3	0	1				1	1				4	7				0	0			
2	3	3	0					1					3					1				
2	2	4	2	1	0	5	1	1	1	1	3	1	3	2	7	8	1	1	1	0	0	1
2	3	3	6		2	1		3		1	1		3		4	1		1		0	1	
2	2	3	1	1				1	1				4	7				0	0			
3	2	3	2			0		1			1											
2	1	4	3	0	4			2	1	3				5	4				0	0		
2	2	3	1	0	0			1	1	1			5	3	2	1		0	1	1	1	
3	3	3	0	1	0	1		1	1	1	1		6	7	7	9		0	0	0	0	
1	1	3	0	1	0			1	1	1			5	3	7			0	1	0		
3	2	3	1	0	0	0	0	1	1	1	1	1	6	7	7	8	5	0	0	0	0	0
2	3	3	0	2				1	1				5	7				0	0			
2	2	1	0					1														
2	2	3	1	1				1	1				4					0				
2	2	3	1	3	3			1	2	2			5	7	7	9		0	0	0	0	
2	2	3	0	1	1			1	1	1			4	4	4	1		0	0	0	1	

2	2	3	0	2	0			1	1	1				1	2				1	1		
2	3	3	1	1	1	4	1	1	1	1	3	1	7	4	3		4	0	0	1		0
1	2	3	1		2			1		1			4					0				
2	2	1	3	5	6	1	6	2	3	3	1	3	3	6	7	9	5	1	0	0	0	0
2	2		0	1	3			1	1	2			6	5	3			0	0	1		
1	2	3	0		1			1		1			4		5			0		0		
2	2	3	0					1					6					0				
1	2	3	2					1					4					0				
1	1	2	5	4				3	3				4	5				0	0			
2	2	3	0	0	1	1	1	1	1	1	1	1	6	7	7	9	4	0	0	0	0	0
2	2	3	1		0	0	0	1		1	1	1			5	6	1			0	0	1
2	2	2	4	2				3	1				3	5				1	0			
2	3	3	1	1				1	1				3	6				1	0			
2	1	3	0	2	2			1	1	1			3	5	2			1	0	1		
2	1		0	1	0			1	1	1			4	7	6			0	0	0		
2	3	3	0	2	1	1	2	1	1	1	1	1	6	7	7	8	3	0	0	0	0	0
2	1	3	1		1		2	1		1		1	4		3		3	0		1		0
2	2	3	3	2				2	1				4	7		9		0	0		0	
2	2	3	0					1					3					1				
2	2	3	1	1	1			1	1	1			6	5	7			0	0	0		
1	1	3	0					1					3					1				
2	1	3	4					3														
2	1	3	1	0	0			1	1	1			6	7	5			0	0	0		
2	2	3	2					1					4					0				
2	1	3	0	2	1	3		1	1	1	2		4	5	5	3		0	0	0	1	
2	1	1	1	0				1	1				2	2				1	1			
2	2	3	0	0	0			1	1	1			5	6	7			0	0	0		
1	2	3	2					1					3					1				
2	1	3	2	2	1			1	1	1			4	3	4			0	1	0		
1	2	3	1	1	6	7		1	1	3	3		3	4	3	1		1	0	1	1	
2	3	3	1	2	1	1		1	1	1	1		5	4				0	0			
2	3	3	0	1	0	1		1	1	1	1		5	5	7	8		0	0	0	0	
2	2	3	3	1	1			2	1	1			3	4	7	7		1	0	0	0	
2	2	3	2	2	1			1	1	1			5	5	7			0	0	0		
2	2	3	2	1				1	1				3	6				1	0			
2	2	2	0					1					4					0				
2	2	1	2	0				1	1				4	4		1		0	0		1	
2	2	3	0	2				1	1				5	5				0	0			
2	3	1	4	2	2			3	1	1			2	4	4			1	0	0		
1	1	2	1					1					4					0				
2	1	3	0		2			1		1			4		7			0		0		

2	2	3	0	0	0			1	1	1			4	4	6			0	0	0		
2	1	3	1	1	0	0		1	1	1	1			4	5	4			0	0	0	
2	2	3	0		2		0	1		1		1	4		7		4	0		0		0
2	2	3	0	0	1			1	1	1			4	4	7			0	0	0		
2	2	4	2					1					4					0				
3	3	3	1	0	0	3	2	1	1	1	2	1	5	5	2	2	3	0	0	1	1	0
2	3	2	2		4			1		3			7		6	3		0		0	1	
2	2	3	3	2	1	1		2	1	1	1		3	2	3			1	1	1		
